# Supplementary material for: Impact of the local valley splitting on the coherence of conveyor-belt spin shuttling in 28Si/SiGe
Source: Nat Commun. 2026 Jun 19;17:5448. doi: 10.1038/s41467-026-74382-5 (PMC13282485; doi:10.1038/s41467-026-74382-5)
Supplement: Supplementary file 1 — Supplementary Information [file 41467_2026_74382_MOESM1_ESM.pdf]

Supplementary Material to
Impact of the local valley splitting on the coherence of conveyor-belt spin shuttling in ^{28}Si/SiGe

Mats Volmer,[1] Tom Struck,[1] Arnau Sala,[1] Jhih-Sian Tu,[2] Stefan Trellenkamp,[2] Davide Degli Esposti,[3] Giordano Scappucci,[3] Łukasz Cywiński,[4] Hendrik Bluhm,[1] and Lars R. Schreiber[1,*]

[1] *JARA-FIT Institute for Quantum Information, Forschungszentrum Jülich GmbH and RWTH Aachen University, Aachen, Germany*
[2] *Helmholtz Nano Facility (HNF), Forschungszentrum Jülich, Jülich, Germany*
[3] *QuTech and Kavli Institute of Nanoscience, Delft University of Technology, Lorentzweg 1, 2628 CJ Delft, The Netherlands*
[4] *Institute of Physics, Polish Academy of Sciences, Warsaw, Poland*

CONTENTS

SUPPLEMENTARY NOTE I. Valley splitting detection vs. initialized valley state 2

SUPPLEMENTARY NOTE II. Optimal visibility of the spin-valley anticrossings 5

SUPPLEMENTARY NOTE III. Raw data for the valley splitting map 5

SUPPLEMENTARY NOTE IV. The time-dependent Hamiltonian for spin and valley degrees of freedom of the shuttled dot 5

SUPPLEMENTARY NOTE V. Unedited version of Figure 3 13

SUPPLEMENTARY NOTE VI. Nonadiabatic valley excitation 14

SUPPLEMENTARY NOTE VII. Dynamics due to shuttling through a spin-valley resonance: the spin-valley flip-flop 16

SUPPLEMENTARY NOTE VIII. Mechanisms of decoherence during the shuttling 18
- A. Dephasing in absence of spin-valley resonances and valley excitations 18
- B. Dephasing due to valley splitting fluctuations activated by the spin-valley flip-flop 19
- C. Spin relaxation near the spin-valley relaxation resonance 21
- D. Valley excitation after shuttling through the valley splitting minimum 21

SUPPLEMENTARY NOTE IX. Discussion of experimental results: effect of single there-and-back shuttling through the spin-valley resonance 22

SUPPLEMENTARY NOTE X. Discussion of experimental results: Multiple passages through valley splitting minimum and spin-valley resonances 23
- A. Passages through a spin-valley resonance 24
- B. Passages through a valley splitting minimum 25

References 29

* lars.schreiber@physik.rwth-aachen.de

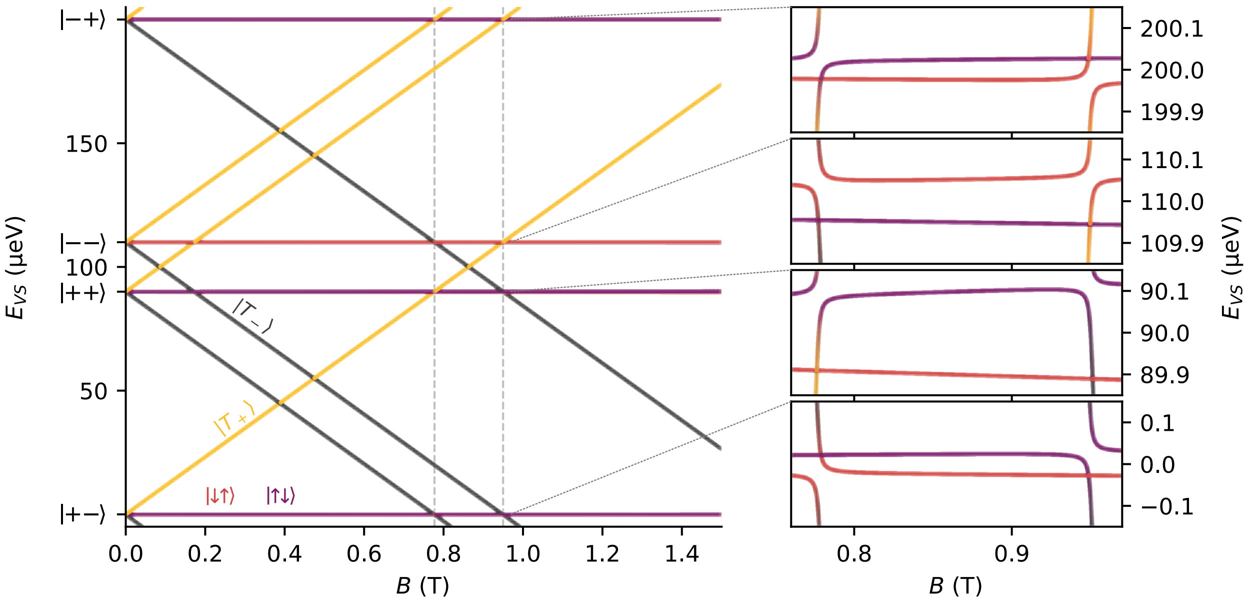

Supplementary Figure 1. Simulated eigenenergies of the full spin and valley degrees of freedom as a function of magnetic field. Insets are included in the vicinity of the anticrossings. Valley states are labelled at the y-axis and spin states are encoded in color.

SUPPLEMENTARY NOTE I: VALLEY SPLITTING DETECTION VS. INITIALIZED VALLEY STATE

In the main text we state that we cannot identify into which specific valley state we initialize the (3,1) charge state in the DQD. For slow enough sweep of interdot detuning, charge transfer from $(4,0)$ state (with both valleys in the L dot fully occupied) to $(3,1)$ state should lead to transfer of one of electrons from the excited valley state in L dot, $|+\rangle_L$, to the ground state in the R dot, $|-\rangle_R$, i.e. creation of S_{+-} singlet with $(3,1)$ charge occupation. This, however, requires the dynamics to be adiabatic with respect to inter-valley tunnel coupling t_{+-} between the respective valley states in the two dots. If this coupling is small, and the intra-valley tunnel couplings, t_{++} and t_{--}, are larger than the inter-valley one, for given detuning sweep rate the charge transfer between $|+\rangle_L$ and $|-\rangle_R$ states might fail (completely, or partially, i.e. the S_{+-} state will be created with proability < 1), while creation of either S_{++} or S_{--} state will occur with high probability due to adiabaticity of dynamics involving the intra-valley tunneling. Full discussion of implications of such nonadiabatic charge and valley dynamics during $(4,0) \rightarrow (3,1)$ transition is given in [1]. Here we focus on showing that the key physical effect underpinning the method of valley splitting mapping used in the main manuscript - a strong renormalizaiton of singlet-triple presession frequency near spin-valley resonances in both dots - occurs independently of the valley occupation pattern of the initialized singlet (or a statistical mixture of singlets).

We focus on $(3,1)$ states, $|v_L v_R \sigma_L \sigma_R\rangle$, labeling them with $v_L = \pm$ (single-occupied valley state in dot L), $v_R = \pm$ (valley state in dot R occupied by the signle electron in that dot), and $\sigma_D =\uparrow / \downarrow$ labeling the projections on the quantization axis of the spins of the electrons in the singly-occupied valley states. The Hamiltonian is defined on a 16-dimensional Hilbert space spanned by the basis $\mathcal{B}$, ordered energetically into four distinct valley manifolds:

$$\begin{aligned}\mathcal{B} &= \{|v_L v_R\rangle \otimes |s_L s_R\rangle\} \\ &= \{\underbrace{|+-\downarrow\downarrow\rangle,\ldots,}_{\text{Ground }(H_{+-})}\ \underbrace{|++\downarrow\downarrow\rangle,\ldots,}_{\text{R-Excited }(H_{++})}\ \underbrace{|--\downarrow\downarrow\rangle,\ldots,}_{\text{L-Excited }(H_{--})}\ \underbrace{|-+\downarrow\downarrow\rangle,\ldots}_{\text{Dual-Excited }(H_{-+})}\}^T\end{aligned} \tag{S1}$$

Within each valley block, the spin basis follows the order $\{\downarrow\downarrow, \downarrow\uparrow, \uparrow\downarrow, \uparrow\uparrow\}$. The full Hamiltonian H is represented in

block-matrix form as:

$$H = \begin{pmatrix} H_{+-} & T_R & T_L & 0 \\ T_R^\dagger & H_{++} & 0 & T_L' \\ T_L^\dagger & 0 & H_{--} & T_R' \\ 0 & {T_L'}^\dagger & {T_R'}^\dagger & H_{-+} \end{pmatrix} \tag{S2}$$

The diagonal blocks describe the magnetic field-dependent Zeeman energy and the static valley splitting offsets. For a specific block with valley configuration (v_L, v_R), the Hamiltonian is diagonal in the spin subspace:

$$H_{v_L v_R} = \text{diag}\left(E_{\downarrow\downarrow}, E_{\downarrow\uparrow}, E_{\uparrow\downarrow}, E_{\uparrow\uparrow}\right) + E_{\text{offset}} \cdot \mathbb{I}_4 \tag{S3}$$

The spin energies are given by $E_{s_L s_R} = \frac{\mu_B B}{2}(g_L s_L + g_R s_R)$. The valley offsets are defined relative to the ground state: zero for H_{+-}, $E_{VS,r}$ for H_{++}, $E_{VS,l}$ for H_{--}, and $E_{VS,l} + E_{VS,r}$ for H_{-+}.

The off-diagonal matrices describe the spin-valley mixing only on the same electron. We exclude cases where a spin flip on the left electron coincides with a valley flip on the right electron. We formally distinguish between transitions originating from the ground valley state (T) and those originating from an already excited valley state (T').

The right-dot coupling T_R connects the ground state ($|+-\rangle$) to the right-excited state ($|++\rangle$). The matrix T_R' describes the same right-dot transition but starting from the left-excited configuration ($|--\rangle \leftrightarrow |-+\rangle$). In this model, we assume $T_R = T_R'$, with the matrix form:

$$T_R = \begin{pmatrix} 0 & 0 & 0 & 0 \\ \Delta_{sv,R} & 0 & 0 & 0 \\ 0 & 0 & 0 & 0 \\ 0 & 0 & \Delta_{sv,R} & 0 \end{pmatrix} \tag{S4}$$

This interaction strictly preserves the left spin state while flipping the right spin and valley indices.

Similarly, T_L and T_L' describe the left-dot valley transition. T_L couples the ground state to the left-excited state ($|+-\rangle \leftrightarrow |--\rangle$), while T_L' couples the right-excited state to the dual-excited state ($|++\rangle \leftrightarrow |-+\rangle$). Assuming $T_L = T_L'$, the coupling matrix is:

$$T_L = \begin{pmatrix} 0 & 0 & 0 & 0 \\ 0 & 0 & 0 & 0 \\ \Delta_{sv,L} & 0 & 0 & 0 \\ 0 & \Delta_{sv,L} & 0 & 0 \end{pmatrix} \tag{S5}$$

This interaction preserves the right spin state while coupling the left spin and valley indices. Using realistic parameters [2] in Supplementary table I, we arrive at the eigenenergies plotted in Fig. 1. We find that independent of which valley state was initialized, the anticrossings with other valley states always appear at the same magnetic fields (see insets in Supplementary Fig. 1) which correspond to the valley splitting energies. The positions are precise up to the relative variation of the g-factors, so the systematic uncertainty is in the permille order of magnitude.

Supplementary Table I. Parameters utilized in the numerical diagonalization. The g-factors $g_{l,0}$ and $g_{r,0}$ correspond to the ground state valley configuration $(L+, R-)$.

Parameter	Symbol	Value	Unit
Valley Splitting			
Left QD	$E_{VS,l}$	110	μeV
Right QD	$E_{VS,r}$	90	μeV
g-factors			
Left (Ground / Excited)	$g_{l,0}$ / $g_{l,1}$	2.002 / 1.999	-
Right (Ground / Excited)	$g_{r,0}$ / $g_{r,1}$	2.001 / 1.998	-
Spin-Valley Coupling			
Spin-Valley Mixing (R)	$\Delta_{sv,R}$	100	neV
Spin-Valley Mixing (L)	$\Delta_{sv,L}$	100	neV

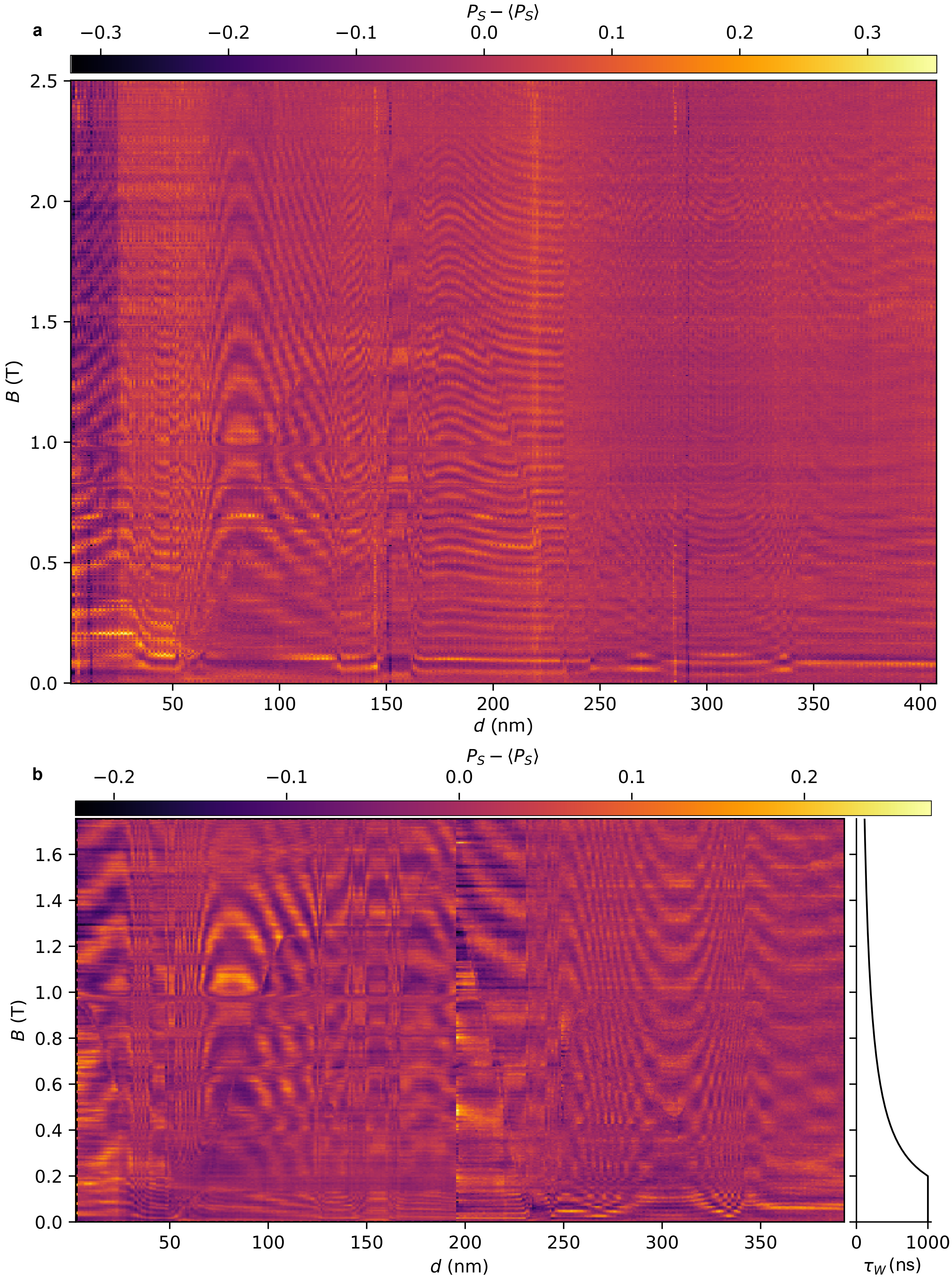

Supplementary Figure 2. Optimal visibility of the spin-valley anticrossings. (a) Valley Splitting map with a constant wait time of 300 ns within the shuttler. (b) Valley Splitting map with varying wait time as a function of magnetic field.

SUPPLEMENTARY NOTE II: OPTIMAL VISIBILITY OF THE SPIN-VALLEY ANTICROSSINGS

In the main text we state that the the wait time is adapted to the external magnetic field in order to identify the spin-valley resonance for valley-splitting mapping. Here, we support this statement by comparing the valley splitting map recorded with a constant wait time of 300 ns (Supplementary Fig. 2a) and one recorded with wait time partially being anti-proportional to the magnetic field (Supplementary Fig.2b). The phase accumulated during the wait time scales linearly with the magnetic field, hence we choose a wait time function that scales with the inverse magnetic field. A function that allowed sensible values across the full range of magnetic fields is

$$\tau_W(B) = \min(1000\,\mathrm{ns}, 200\,\mathrm{ns\,T}/B). \tag{S6}$$

In order to avoid the divergence at zero field, we limit the wait time to 1000 ns. Thus, the wait time below 200 mT is constant so that the background oscillations remain. Everywhere else, the oscillations are damped such that the valley splitting line is more clearly isolated. For some traces, this reduces the background so much that there is almost no oscillation left.

SUPPLEMENTARY NOTE III: RAW DATA FOR THE VALLEY SPLITTING MAP

In this section, we display the raw data for the valley splitting map. In more than thirty high resolution measurements, we captured this complete valley splitting map. Here, we display all of them with the following optimization of the visibility. We always subtract the linewise mean to align the data and subsequently cut the color scale from the 5% to the 95% quantile in order to eliminate outliers that distract the color scale. Further improvements of the visibility are due to the diverse background scenarios unattainable. All figures firstly display a full plot of the trace with dashed areas indicating the individual measurements. The subsequent two plots are the large left half and right half of the measurement. Subsequently, we provide the detailed plot in ascending order of d. All plots are equipped with manual guides to the eye for the found valley splitting curve. These plots are shown in Supplementary Fig. 3 through Fig. 9

SUPPLEMENTARY NOTE IV: THE TIME-DEPENDENT HAMILTONIAN FOR SPIN AND VALLEY DEGREES OF FREEDOM OF THE SHUTTLED DOT

The Hamiltonian describing the coupling between the $|\pm k_z\rangle$ valleys for an electron strongly confined in the z-direction, the spatial wavefunction of which is time-dependent is given by

$$\hat{H}_v(t) = \begin{pmatrix} 0 & \Delta^*(t) \\ \Delta(t) & 0 \end{pmatrix} = |\Delta(t)| \left[\cos\phi(t)\hat{\tau}_x + \sin\phi(t)\hat{\tau}_y\right] \;, \tag{S7}$$

in which we have written the real and imaginary part of the valley coupling $\Delta(t)$ as $\Delta_R(t) = |\Delta(t)|\cos\phi(t)$ and $\Delta_I(t) = |\Delta(t)|\sin\phi(t)$. The time dependence of the valley coupling $\Delta(t)$ follows from the time dependence of the envelope wavefunction of the electron. Under an assumption that the shuttling of the electron leads to a shift of the mean position of the electron, $x(t)$, with possible deformations of the shape of the envelope wavefunction neglected, the time dependence of this coupling simply follows from spatial dependence of the coupling when a QD of fixed shape is positioned at $x(t)$, i.e. $\Delta(t) = \Delta[x(t)]$, and the wavefunction is overlapping with a particular realization of atomic disorder (positions of Si and Ge atoms) at the Si/SiGe interface. Examples of trajectories of $\Delta(x)$ can be found e.g. in [3, 4], and in Sec. SUPPLEMENTARY NOTE VI.

In order to see how time dependence of $\Delta[x(t)]$ activated by shuttling of the dot leads to valley excitations we use the basis of instantaneous valley eigenstates $|\pm(t)\rangle$ that fulfil $\hat{H}_v(t)\,|\pm(t)\rangle = \pm|\Delta(t)|\,|\pm(t)\rangle$. We assume that at the initial time t_0 the electron is in one of the valley eigenstates, $|\pm(t_0)\rangle$. After introducing the unitary transformation between the eigenstates at initial time t_0 and later time t defined by $|\pm(t)\rangle = \hat{U}(t,t_0)\,|\pm(t_0)\rangle$ we arrive at the effective Hamiltonian $\tilde{H}_v(t) = \hat{U}\hat{H}(t)\hat{U}^\dagger + i\hbar(\mathrm{d}\hat{U}/\mathrm{d}t)\hat{U}^\dagger$ that in the basis of $\{|-(t)\rangle\,, |+(t)\rangle\}$ instantaneous valley eigenstates reads

$$\tilde{H}_v(t) = \begin{pmatrix} -|\Delta(t)| & -\frac{\hbar}{2}\dot{\phi}(t) \\ -\frac{\hbar}{2}\dot{\phi}(t) & |\Delta(t)| \end{pmatrix} \;, \tag{S8}$$

where $\dot{\phi}(t) = \mathrm{d}\phi(t)/\mathrm{d}t$. Transitions between the instantaneous eigenstates become significant when $\dot{\phi}$ becomes comparable to $|\Delta|$: if we assume a constant $\dot{\phi} \equiv \omega$, and a constant $|\Delta|$ (so that the trajectory of $\Delta(t)$ corresponds to a circle

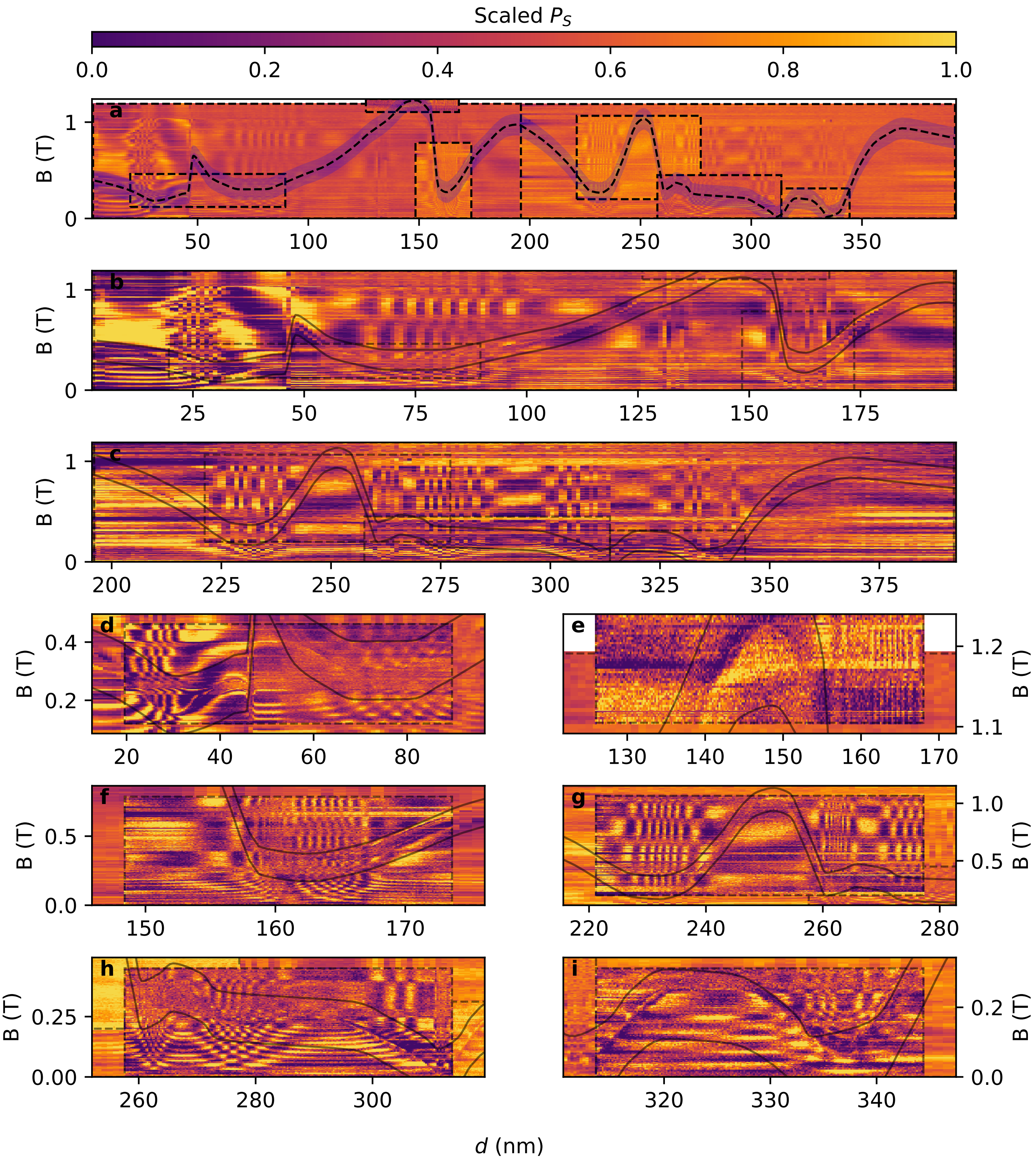

Supplementary Figure 3. Raw data for applying a voltage of $V_{\mathrm{ST}} = 0.3\,\mathrm{V}$ and $V_{\mathrm{SB}} = 0\,\mathrm{V}$ leading to a coordinate of $y = 18\,\mathrm{nm}$ with native contrast. Guides to the eye are added at $\pm 100\,\mathrm{mT}$ with respect to the spline position in panels b-i. (a) Complete Map composed of eight different measurements. (b) Broad measurement of the first 196 nm. (c) Broad measurement of the second 196 nm. (d-i) Fine measurements ordered from left to right. Two offset copies of the spline fit are added as a guide to the eye.

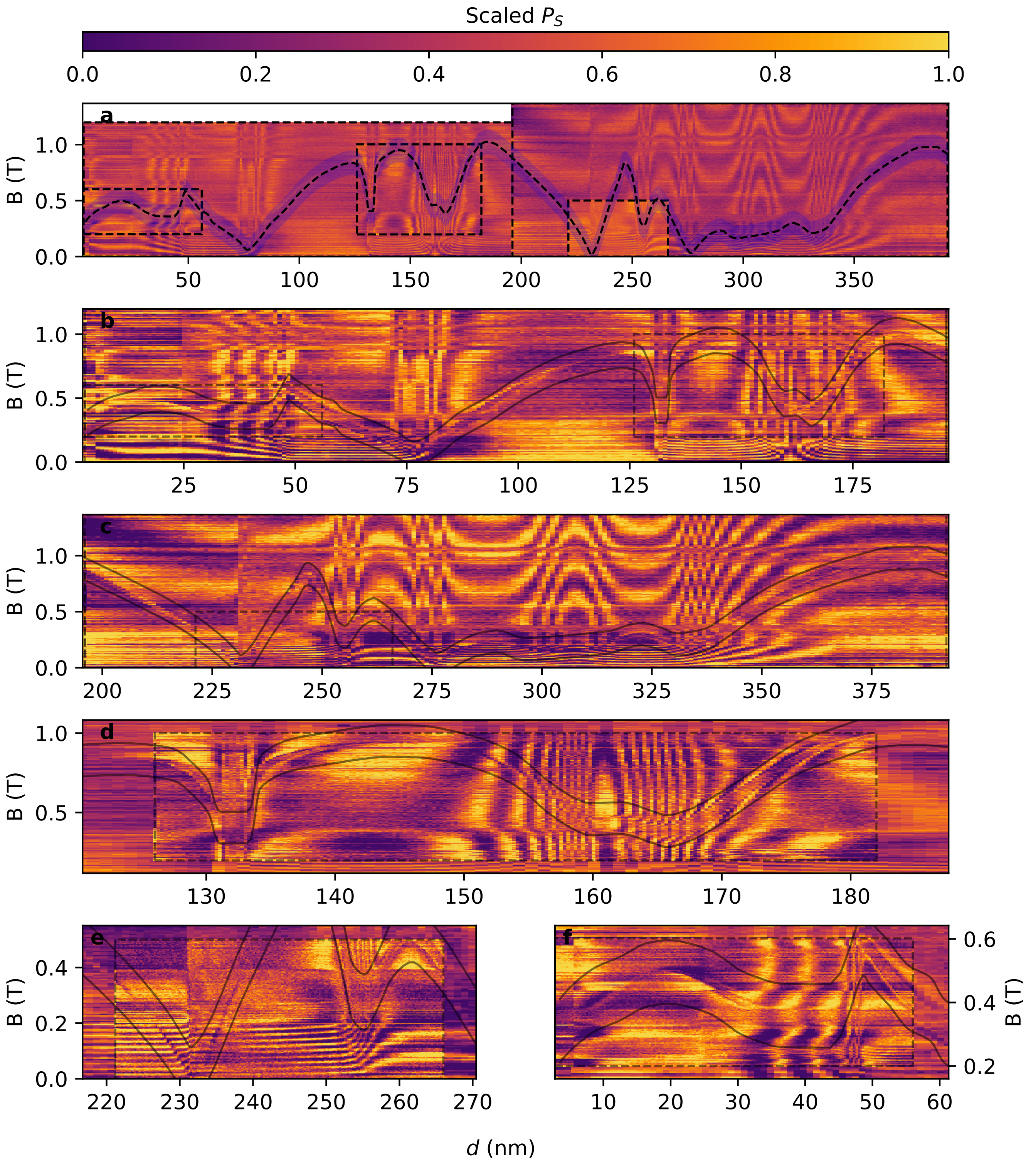

Supplementary Figure 4. Raw data for applying a voltage of $V_{\mathrm{ST}} = 0.25\,\mathrm{V}$ and $V_{\mathrm{SB}} = 0.05\,\mathrm{V}$ leading to a coordinate of $y = 12\,\mathrm{nm}$ with native contrast. Guides to the eye are added at $\pm 100\,\mathrm{mT}$ with respect to the spline position in panels b-f. (a) Complete Map composed of five different measurements. (b) Broad measurement of the first 196 nm. (c) Broad measurement of the second 196 nm. (d) Largest fine measurement at around 150 nm. (e-f) Fine measurements ordered from left to right. Two offset copies of the spline fit are added as a guide to the eye.

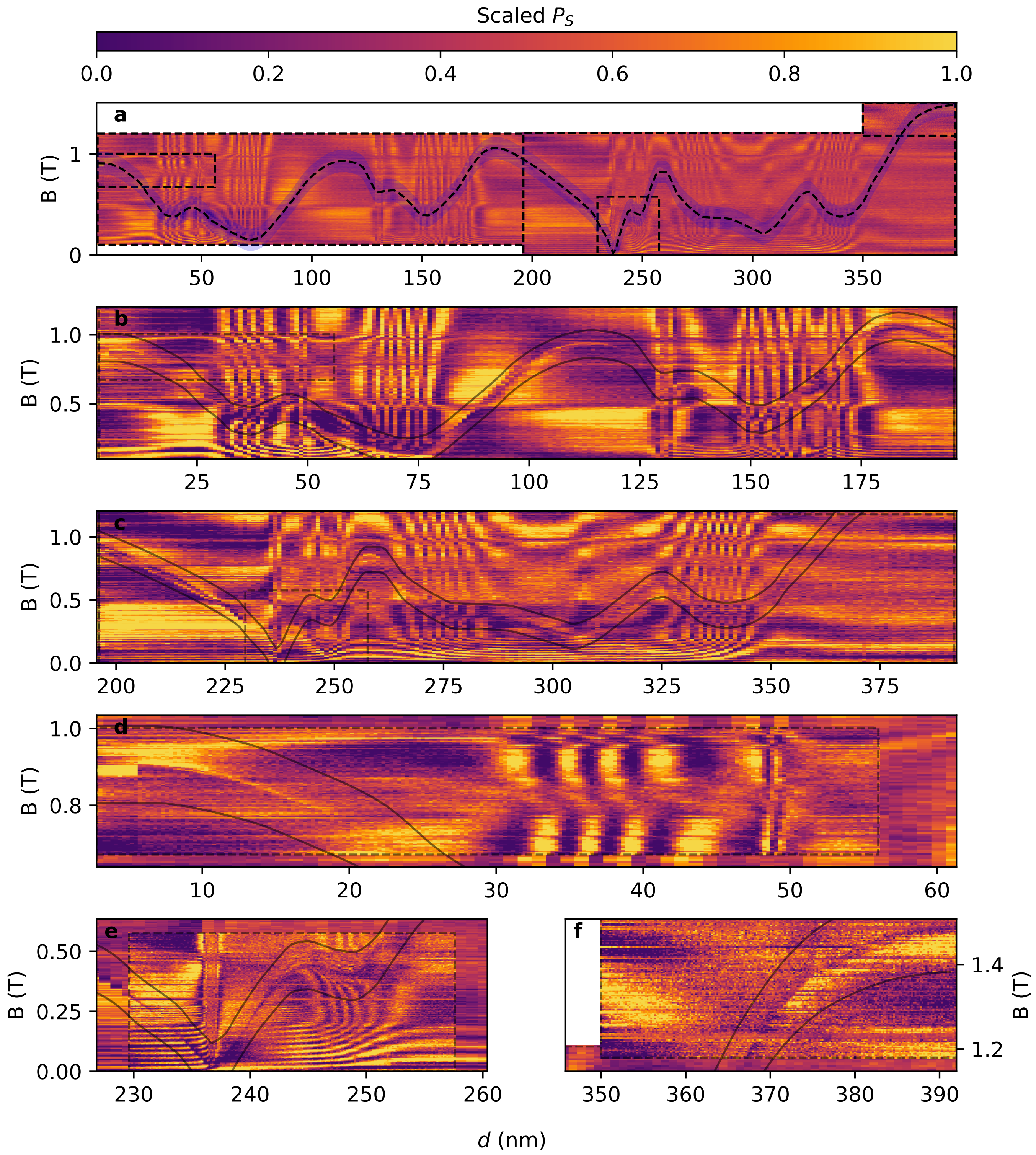

Supplementary Figure 5. Raw data for applying a voltage of $V_{\mathrm{ST}} = 0.2\,\mathrm{V}$ and $V_{\mathrm{SB}} = 0.1\,\mathrm{V}$ leading to a coordinate of $y = 6\,\mathrm{nm}$ with native contrast. Guides to the eye are added at $\pm 100\,\mathrm{mT}$ with respect to the spline position in panels b-f. (a) Complete Map composed of five different measurements. (b) Broad measurement of the first 196 nm. (c) Broad measurement of the second 196 nm. (d) Largest fine measurement at around 30 nm. (e-f) Fine measurements ordered from left to right. Two offset copies of the spline fit are added as a guide to the eye.

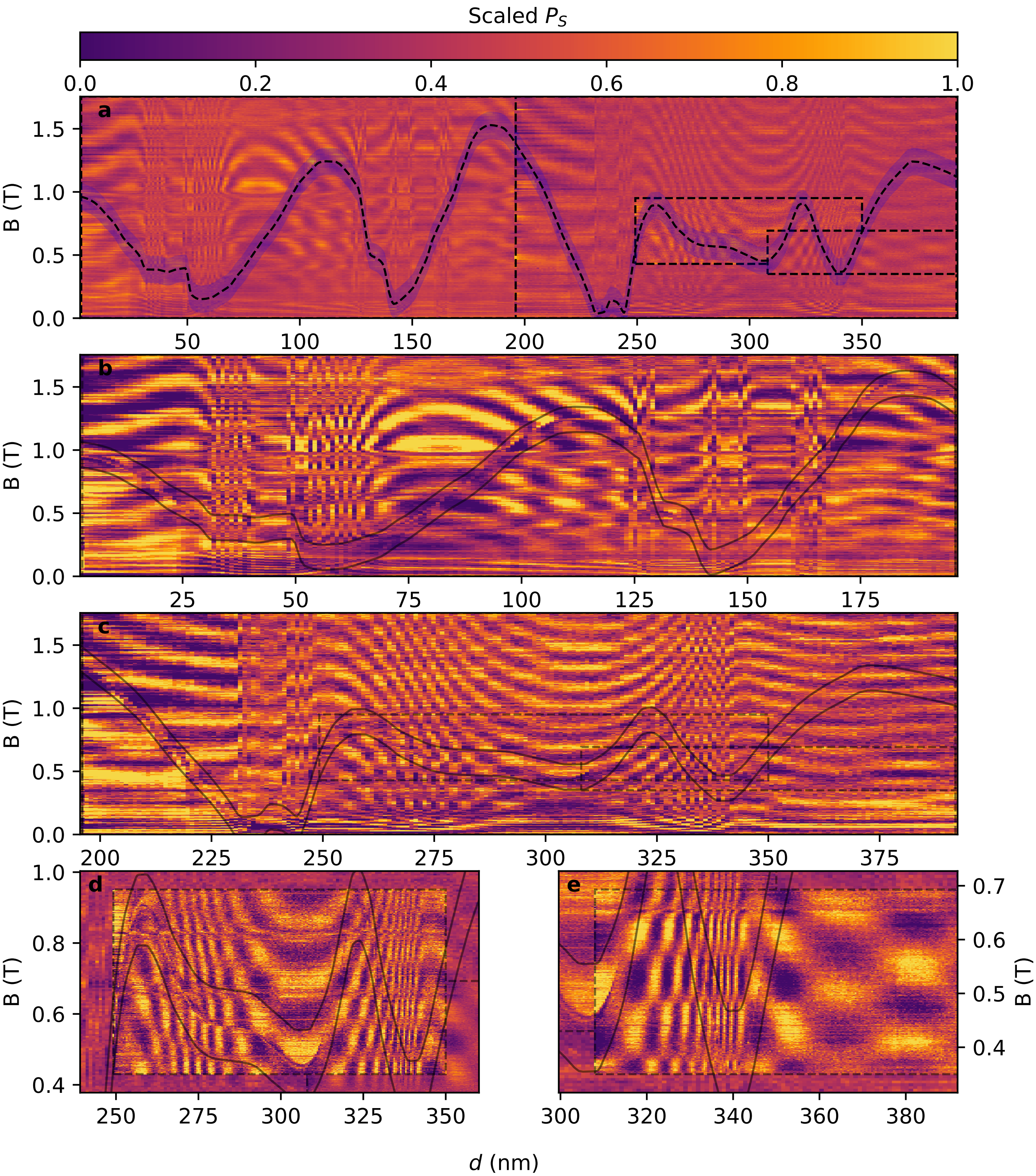

Supplementary Figure 6. Raw data for applying a voltage of $V_{\mathrm{ST}} = 0.15\,\mathrm{V}$ and $V_{\mathrm{SB}} = 0.15\,\mathrm{V}$ leading to a coordinate of $y = 0\,\mathrm{nm}$ with native contrast. Guides to the eye are added at $\pm 100\,\mathrm{mT}$ with respect to the spline position in panels b-e. (a) Complete Map composed of four different measurements. (b) Broad measurement of the first 196 nm. (c) Broad measurement of the second 196 nm. (d-e) Fine measurements ordered from left to right. Two offset copies of the spline fit are added as a guide to the eye.

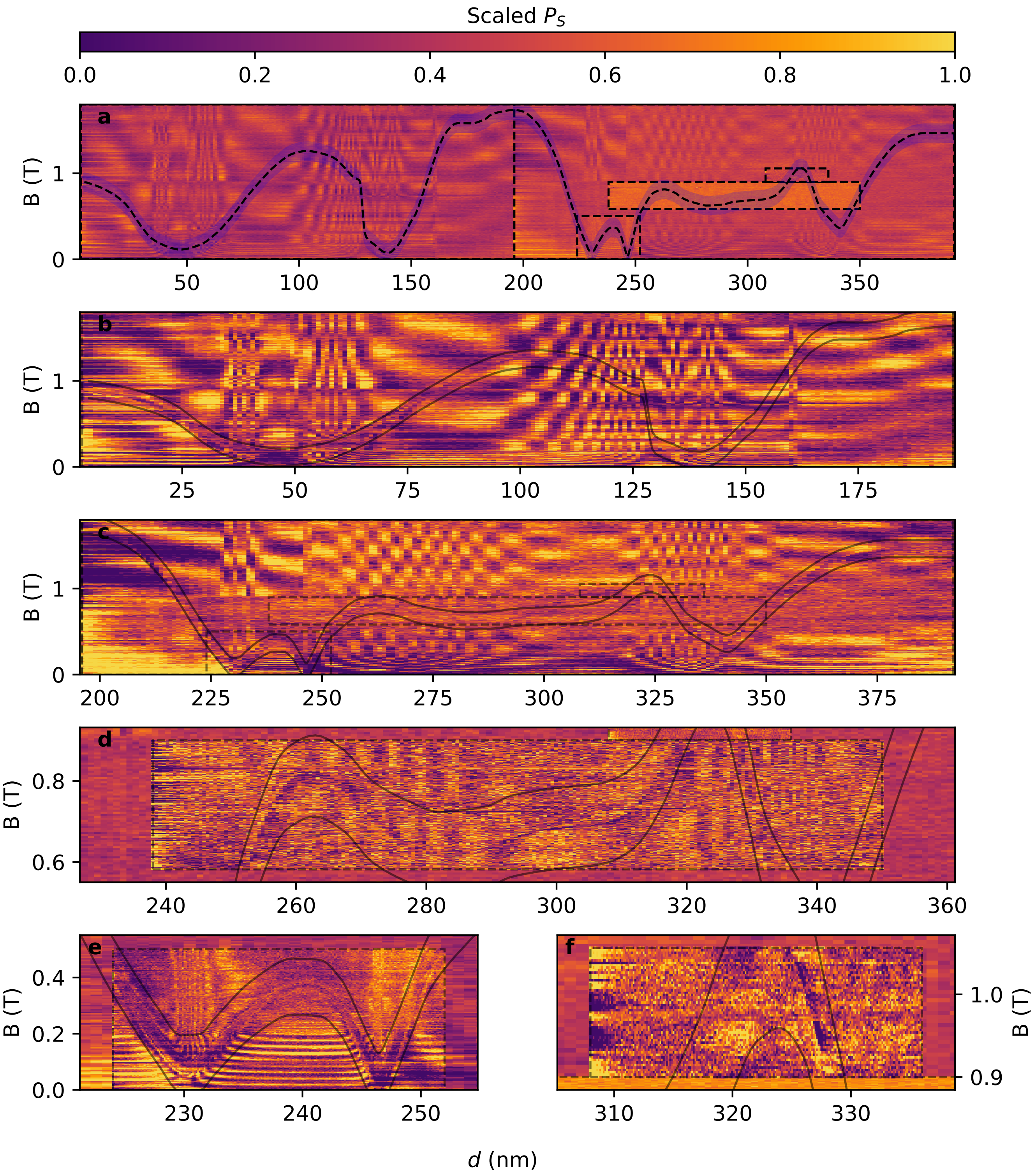

Supplementary Figure 7. Raw data for applying a voltage of $V_{\mathrm{ST}} = 0.1\,\mathrm{V}$ and $V_{\mathrm{SB}} = 0.2\,\mathrm{V}$ leading to a coordinate of $y = -6\,\mathrm{nm}$ with native contrast. Guides to the eye are added at $\pm 100\,\mathrm{mT}$ with respect to the spline position in panels b-f. (a) Complete Map composed of five different measurements. (b) Broad measurement of the first 196 nm. (c) Broad measurement of the second 196 nm. (d) Largest fine measurement at around 300 nm. (e-f) Fine measurements ordered from left to right. Two offset copies of the spline fit are added as a guide to the eye.

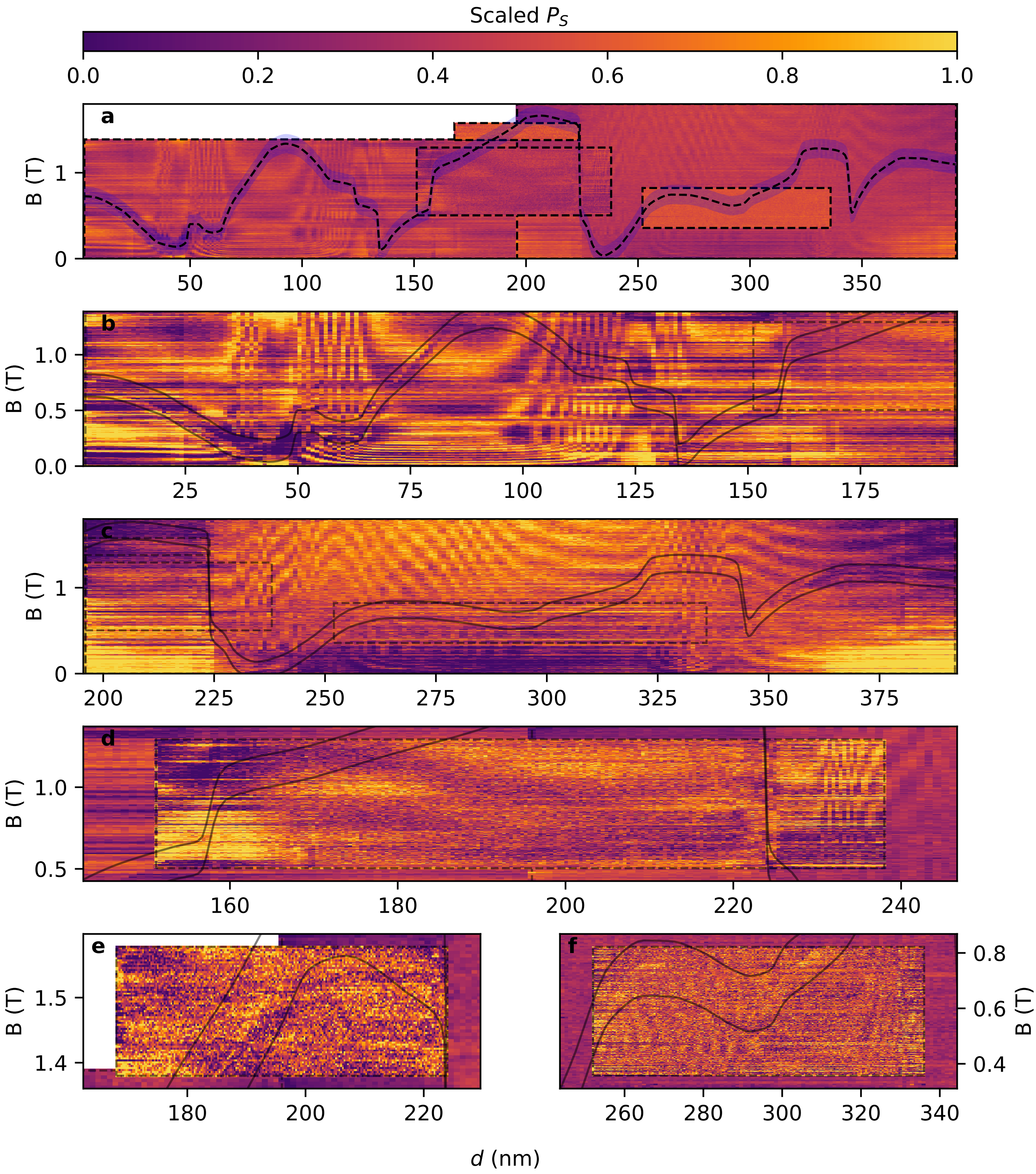

Supplementary Figure 8. Raw data for applying a voltage of $V_{\mathrm{ST}} = 0.05\,\mathrm{V}$ and $V_{\mathrm{SB}} = 0.25\,\mathrm{V}$ leading to a coordinate of $y = -12\,\mathrm{nm}$ with native contrast. Guides to the eye are added at $\pm 100\,\mathrm{mT}$ with respect to the spline position in panels b-f. (a) Complete Map composed of five different measurements. (b) Broad measurement of the first 196 nm. (c) Broad measurement of the second 196 nm. (d) Largest fine measurement at around 200 nm. (e-f) Fine measurements ordered from left to right. Two offset copies of the spline fit are added as a guide to the eye.

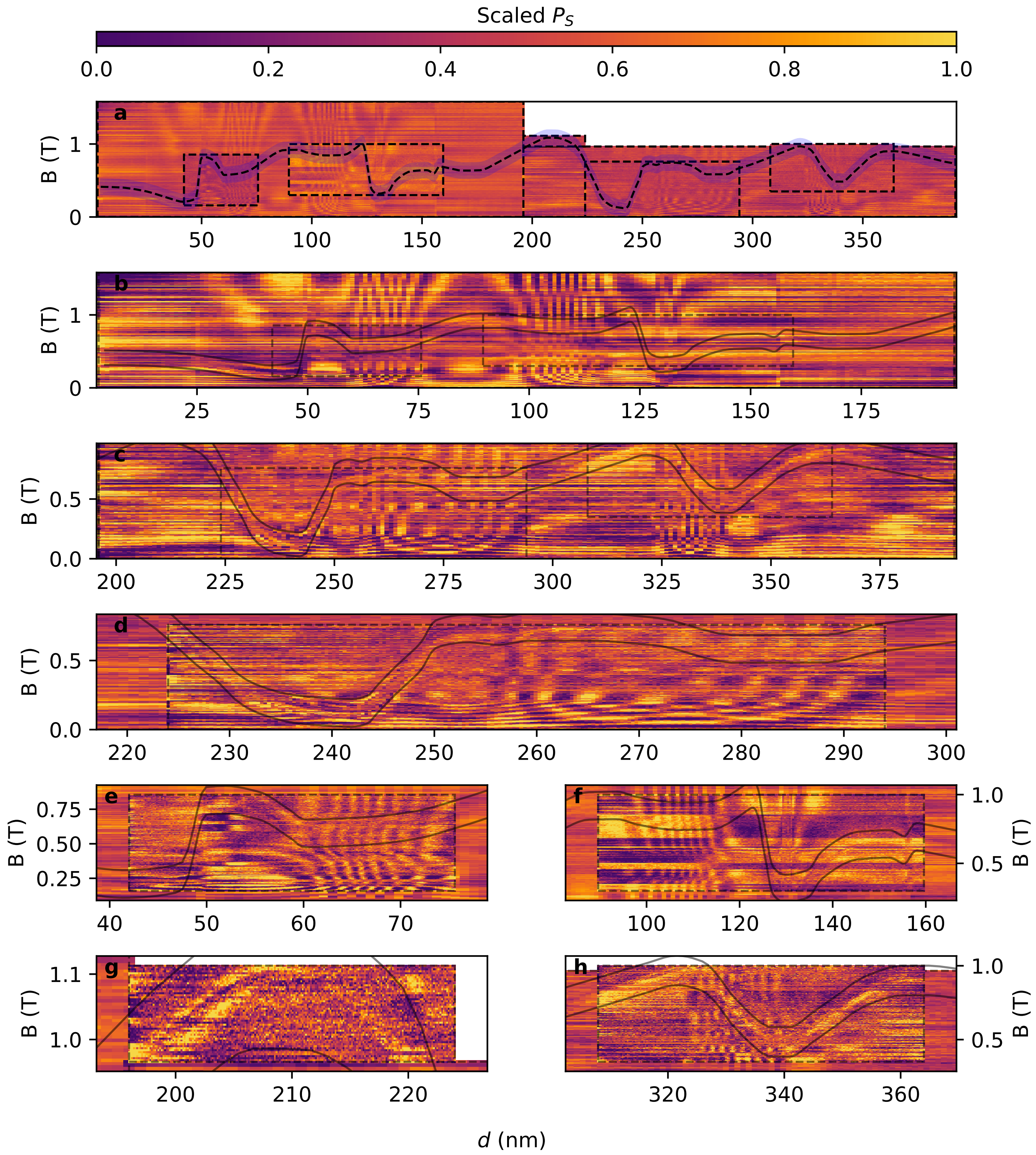

Supplementary Figure 9. Raw data for applying a voltage of $V_{\mathrm{ST}} = 0\,\mathrm{V}$ and $V_{\mathrm{SB}} = 0.3\,\mathrm{V}$ leading to a coordinate of $y = -18\,\mathrm{nm}$ with native contrast. Guides to the eye are added at $\pm 100\,\mathrm{mT}$ with respect to the spline position in panels b-h. (a) Complete Map composed of seven different measurements. (b) Broad measurement of the first 196 nm. (c) Broad measurement of the second 196 nm. (d) Largest fine measurement at around 260 nm. (e-h) Fine measurements ordered from left to right. Two offset copies of the spline fit are added as a guide to the eye.

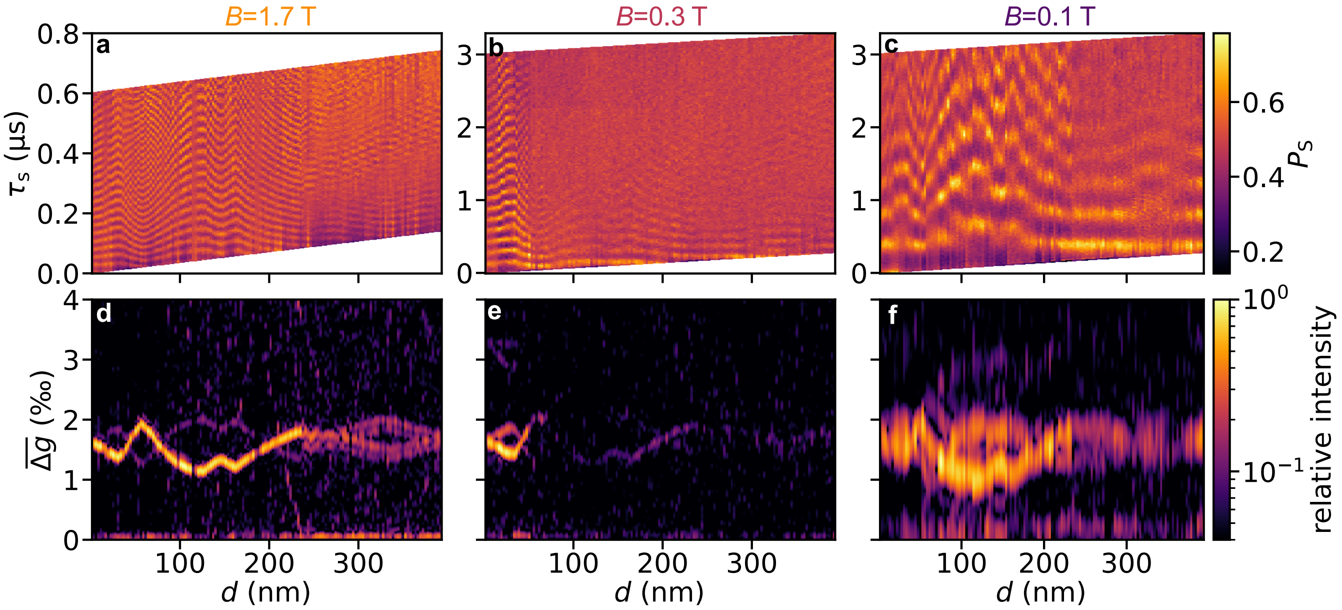

Supplementary Figure 10. Figure 3 from the main text without guides to the eye. Panels (a-f) relate to panels (b-g) of Figure 3 from the main text.

in the complex plane), the maximum occupation of the valley state that is not adiabatically connected to the initial one is

$$Q_{v,max} = \frac{(\hbar\omega)^2}{4|\Delta|^2 + (\hbar\omega)^2} \ , \tag{S9}$$

which is $\approx |\hbar\omega/2\Delta|^2$ when $|\Delta| \gg \hbar\omega$.

It is well established that interplay of interface roughness and spin-orbit interaction causes the g-factor of electron's spin to depend on the valley Hamiltonian eigenstate [5, 6], and it leads to an appearance of finite spin-valley coupling matrix elements connecting $|-\uparrow\rangle$ to $|+\downarrow\rangle$ and $|+\uparrow\rangle$ to $|-\downarrow\rangle$ states [7, 8]. After including these terms we arrive at the following time-dependent Hamiltonian acting in the four-dimensional subspace of lowest energy spin/valley states of the shuttled dot, $\{|-\uparrow(t)\rangle, |-\downarrow(t)\rangle, |+\uparrow(t)\rangle, |+\downarrow(t)\rangle\}$,

$$\tilde{H}(t) = \begin{pmatrix} \frac{1}{2}[E_Z^- - E_{\mathrm{VS}}] & 0 & -\frac{\hbar}{2}\dot{\phi} & \Delta_{sv} \\ 0 & \frac{1}{2}[-E_Z^- - E_{\mathrm{VS}}] & \Delta'_{sv} & -\frac{\hbar}{2}\dot{\phi} \\ -\frac{\hbar}{2}\dot{\phi} & \Delta'^*_{sv} & \frac{1}{2}[E_Z^+ + E_{\mathrm{VS}}] & 0 \\ \Delta^*_{sv} & -\frac{\hbar}{2}\dot{\phi} & 0 & \frac{1}{2}[-E_Z^+ + E_{\mathrm{VS}}] \end{pmatrix} \ . \tag{S10}$$

In the above Hamiltonian the Zeeman splitting in $\pm$ valley, $E_Z^{\pm} = g_{\pm}\mu_B B$, the valley splitting $E_{\mathrm{VS}} = 2|\Delta|$, spin-valley couplings Δ_{sv} and Δ'_{sv}, and valley phase ϕ are all implicitly time-dependent.

The shuttled electron could be initialized in either $|-\rangle$ or $|+\rangle$ valley state. For simplicity of presentation let us assume that it is initialized in the lower energy valley state, $|+\rangle$, as most of discussion below applies after changing the initial state to $|-\rangle$. Whenever the initial valley state of the electron matters for the dynamics, we will make an appropriate remark.

SUPPLEMENTARY NOTE V: UNEDITED VERSION OF FIGURE 3

In this section, we show a plot of an unedited version of Figure 3 from the main text without arrows and lines (Supplementary Figure 10)

SUPPLEMENTARY NOTE VI: NONADIABATIC VALLEY EXCITATION

When spin-valley coupling is negligible, or the Zeeman splitting and $E_{\mathrm{VS}}(x)$ profile along the shuttling path are such that no spin-valley resonances are encountered, or v_S is large enough to passage through these resonances is very close to being diabatic, the Hamiltonian (S10) can be treated as sum of two Hamiltonians, $\hat{H}_{\uparrow}(t)+\hat{H}_{\downarrow}(t)$, each acting in subspace of fixed spin:

$$\hat{H}_{\uparrow}(t) = \begin{pmatrix} \frac{1}{2}E_Z^-(t) & 0 \\ 0 & \frac{1}{2}E_Z^+(t) \end{pmatrix} + \tilde{H}_v(t) \ ,$$
$$\hat{H}_{\downarrow}(t) = \begin{pmatrix} -\frac{1}{2}E_Z^-(t) & 0 \\ 0 & -\frac{1}{2}E_Z^+(t) \end{pmatrix} + \tilde{H}_v(t) \ , \tag{S11}$$

in which $\hat{H}_v$ is given by Eq. (S8). Let us define the difference of Zeeman splittings for the shuttled electron in the two valley states:

$$\delta E_{Z,R} \equiv E_Z^+ - E_Z^- \ . \tag{S12}$$

When $E_{\mathrm{VS}} \gg \frac{1}{2}|\delta E_{Z,R}|$, we can neglect the difference of Zeeman splittings in two valleys when considering nontrivial (i.e. valley-changing) dynamics due to $\hat{H}_v(t)$. Note that at highest considered magnetic fields of about 1 Tesla, and for a large value of intervalley g-factor difference, $\delta g \equiv g_+ - g_- \approx 10^{-3}$, we have $|\delta E_{Z,R}| \approx 0.1$ μeV, which is an order of magnitude smaller than the lowest measured E_{VS}. We can thus safely assume that even at points of low E_{VS}, at which $\dot{\phi}$ could cause intervalley transitions, the dynamics caused by this term is to a very good approximation the same for both spin directions. We will also assume that the valley dynamics caused by $\tilde{H}_v(t)$ occurs in windows of time (narrow regions of x) that are narrow enough to allow for treating the dynamics as impulse-like from the point of view of spin evolution, i.e. the passage of the qubit through a region of small E_{VS} and large $\dot{\phi}$ amounts to the following state transformation:

$$\frac{1}{\sqrt{2}}(|-\uparrow\rangle + e^{i\alpha}|-\downarrow\rangle) \longrightarrow \frac{1}{\sqrt{2}}(a_-|-\rangle + a_+|+\rangle) \otimes (|\uparrow\rangle + e^{i\alpha}|\downarrow\rangle) \ , \tag{S13}$$

in which we have assumed, without any loss of generality, that an equal spin superposition is being shuttled, and α is the relative phase between two amplitudes of the spin qubit at the moment of entering the narrow region of valley excitation. The probability of valley excitation is then given by $Q_v \equiv |a_+|^2$.

Let us estimate when the valley excitations probability can become significant. According to the current understanding of influence of atomistic disorder at Si/SiGe interface on the valley coupling, the real and imaginary components of the coupling are independent Gaussian random fields [3, 9–11], each having autocorrelation length of the order of the spatial extent of the orbital ground state wavefunction, and statistical properties of values E_{VS} encountered over a large enough area are very well captured by the Rice probability distribution, $P(E_{\mathrm{VS}}) = \frac{E_{\mathrm{VS}}}{\sigma^2}\exp\left(-(E_{\mathrm{VS}}^2+\gamma^2)/2\sigma^2\right) I_0(E_{\mathrm{VS}}\gamma/\sigma^2)$, where $I_0(z)$ is the modified Bessel function of the first kind. As discussed in the main text, in the measured sample we have $\sigma \approx 61$ μeV and $\gamma < 1$ μeV, so we are in the "disordered" regime in which $\sigma \gg \gamma$. The typical change of magnitude of both real and imaginary component of Δ when the dot is shuttled by a distance larger than twice the in-plane confinement length (about 30 nm) is then $\approx \sqrt{\pi}\sigma$ [10]. Using the data from Fig. 2 in the main text we arrive at an estimate that a typical distance $|\Delta(x_1) - \Delta(x_2)|$ in the complex plane of valley coupling for $|x_2 - x_1| \approx 30$ nm is ≈ 100 μeV, and consequently an estimate for typical magnitudes of derivatives $v_\Delta \equiv |\mathrm{d}\Delta_{R/I}/\mathrm{d}x|$ is $v_\Delta \sim 3$ μeV/nm.

For given $|\Delta|$ the maximal value of $\dot{\phi}$ is obtained when the $\Delta(x)$ trajectory locally corresponds to motion in a direction that is tangent to a circle of constant $|\Delta|$. Using the v_Δ rate of change of Δ due to change of x introduced above, we see that $|\Delta||\dot{\phi}| \sim v_\Delta v_S$, where v_S is the shuttling velocity, and consequently $|\dot{\phi}| \sim v_\Delta v_S/|\Delta|$. Small values of $|\Delta|$ are thus necessary for sizable nonadiabaticity of evolution described by Hamiltonian (S8). For a given trajectory of $\Delta(t)$ in the complex plane, the valley excitations will occur most probably when the shuttled dot passes through local minima of $E_{\mathrm{VS}}(x) = 2|\Delta(x)|$. Using $v_\Delta \approx 3$ μeV/nm and $v_S \approx 1$ m/s, we can expect significant effect of valley excitation only when the minimal $|\Delta| < 10$ μeV.

In order to verify the accuracy of the above estimates, and to gain more insight into valley excitation probability caused by fast shuttling through E_{VS} minima, we have numerically generated landscape of $\Delta(x)$ using the statistical description for random fields $\mathrm{Re}\Delta(x)$ and $\mathrm{Im}\Delta(x)$ given in [3, 10]. in Supplementary Fig. 11 we show an example of trajectory of $\Delta(x)$ for $x \in [0, 700]$ nm, and the corresponding $E_{\mathrm{VS}}(x)$. Six minima of valley splitting with $E_{\mathrm{VS}} < 25$ μeV are marked with boxes. The $\Delta(x)$ trajectories near each minimum are shown in Supplementary Fig. 12.

Before we discuss the calculation of probability of valley excitation during shuttling through these minima, let us comment on the relationship between the dynamics following from $\hat{H}_v(t)$, and the one described by the Landau-Zener

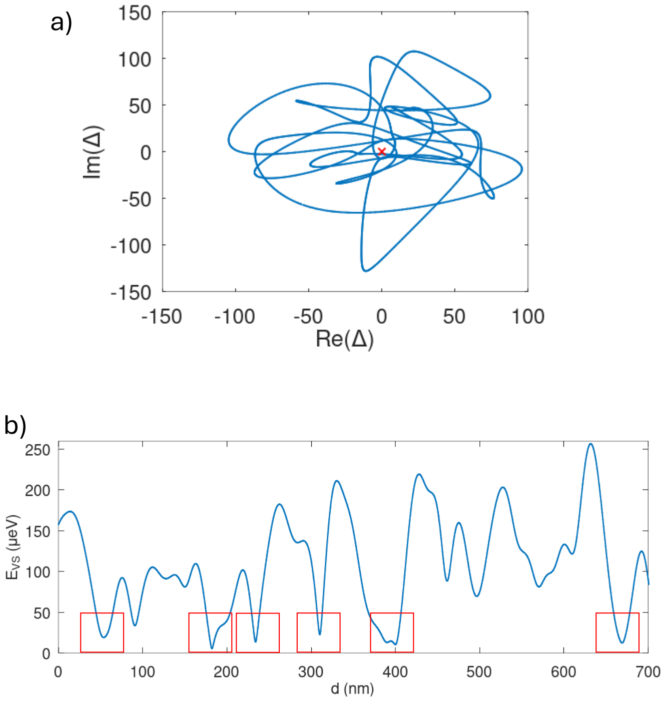

Supplementary Figure 11. a) Simulated trajectory of valley coupling parameter $\Delta(d)$ for $d \in [0, 700]$ nm. b) The corresponding d dependence of valley splitting $E_{\rm VS}(f) = 2|\Delta(d)|$. Six regions containing local minima with $E_{\rm VS} < 25$ μeV are marked with red squares. The values of $E_{\rm VS}$ at the consecutive minima are 19.1, 5.3, 13.6, 22.5, 10.4, and 12.5 μeV.

model. This model corresponds to $\Delta(x)$ trajectory being a straight line in the complex plane [4] that is traversed with constant velocity when $\mathrm{d}x/\mathrm{d}t \equiv v_S$ is constant. For any trajectory of $\Delta(x)$, the Landau-Zener approximation is obtained by finding the point on the trajectory of minimum value of $|\Delta|$, denoted as $|\Delta_0|$, and finding the tangent to the trajectory at this point and the value of $v_\Delta = \sqrt{(\mathrm{d}\Delta_R/\mathrm{d}x)^2 + (\mathrm{d}\Delta_I/\mathrm{d}x)^2}$, with derivatives evaluated at $x = x_0$ at which local minimum of valley splitting occurs, i.e. $E_{\rm VS}(x_0) = 2|\Delta_0|$. Valley dynamics due to shuttling along the tangent is described (after an appropriate choice of basis) by the Landau-Zener Hamiltonian

$$\hat{H}_{LZ} = \begin{pmatrix} v_\Delta v_S t & |\Delta_0| \\ |\Delta_0| & -v_\Delta v_S t \end{pmatrix} , \tag{S14}$$

which gives the valley excitation probability

$$Q_{LZ} = \exp\left(-\pi |\Delta_0|^2 / \hbar v_\Delta v_S\right) . \tag{S15}$$

Let us now see how well the dynamics near the $E_{\rm VS}$ minima can be approximated by the Landau-Zener model. in Supplementary Fig. 12 we plot the $\Delta(x)$ trajectories near the 6 valley splitting minima marked in Supplementary Fig. 11. For each of the trajectories, we calculate the probability of valley excitation assuming that the state at initial x is the lower-energy valley eigenstate, evolving it with time-dependent Hamiltonian $\hat{H}_v(t)$ (in which the time dependence comes from $\Delta(x)$ with $x = v_S t$), and calculating the modulus squared of the overlap of the final state with the higher-energy eigenstate of $\hat{H}_v$ in which $\Delta = \Delta_f$ is the valley coupling at the end of the given trajectory. The resulting final occupation of the excited valley state, Q_v, is plotted as a function of shuttling velocity v_S, and compared with the result of the Landau-Zener approximation calculated as discussed above.

The obtained results confirm the expectation that the Landau-Zener model does not necessarily accurately reproduce the exact Q_v, even if the knowledge of $\Delta(x)$ trajectory is used to choose the effective parameters for this model. This is most apparent for the 1st and 5th minimum. It should be noted that the discrepancy between the exact Q_v and Q_{LZ} in the latter case becomes more prominent at v_S larger than shown in the Figure. In both cases $\Delta(x)$ makes a partial or full loop around $\Delta = 0$. Let us note that for the 5th trajectory, this results in the presence of two closely spaced minima in $E_{\rm VS}(x)$.

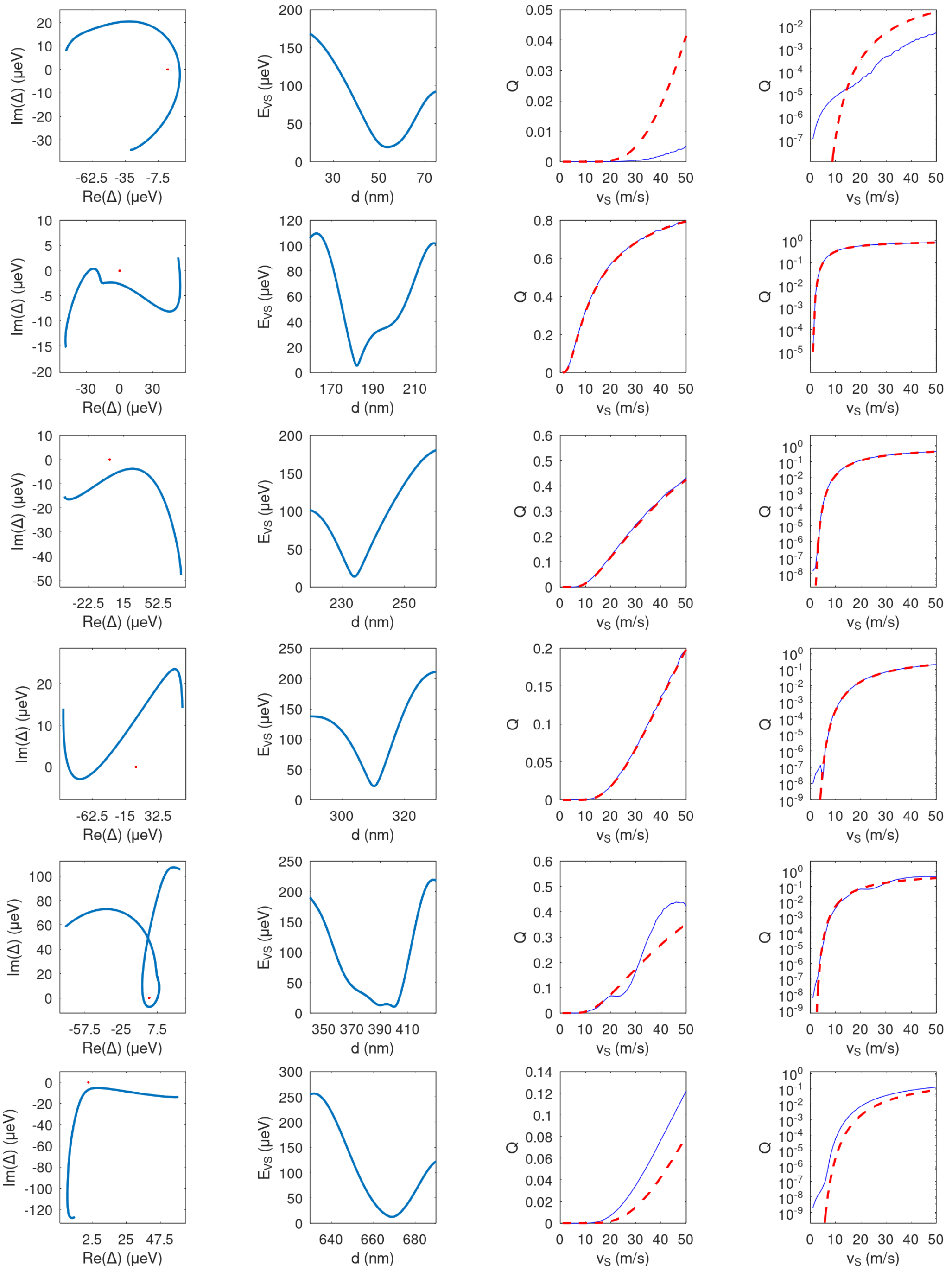

Supplementary Figure 12. Rows correspond to consecutive minima of E_{VS} marked in Supplementary Fig. 11. 1st column: trajectory of $\Delta(x)$. 2nd column: the corresponding $E_{\mathrm{VS}}(x) = 2|\Delta(x)|$. 3rd column: Blue solid line is the probability of excitation out of initial lower energy valley state, Q_v, when shuttling over the distance corresponding to range of x for the plot from the 2nd column; red dashed line: Landau-Zener approximation Q_{LZ} from Eq. (S15); 4th column: the same in the logarithmic scale.

SUPPLEMENTARY NOTE VII: DYNAMICS DUE TO SHUTTLING THROUGH A SPIN-VALLEY RESONANCE: THE SPIN-VALLEY FLIP-FLOP

As we have by definition $E_{\mathrm{VS}} \geq 0$ and $E_Z^{\pm} > 0$, only $|-\uparrow\rangle$ and $|+\downarrow\rangle$ states can get close in energy, so only the Δ_{sv} spin-valley coupling can be relevant for dynamics, and it is in fact relevant only when $|\frac{1}{2}(E_Z^- + E_Z^+) - E_{\mathrm{VS}}| \lesssim 2|\Delta_{sv}|$.

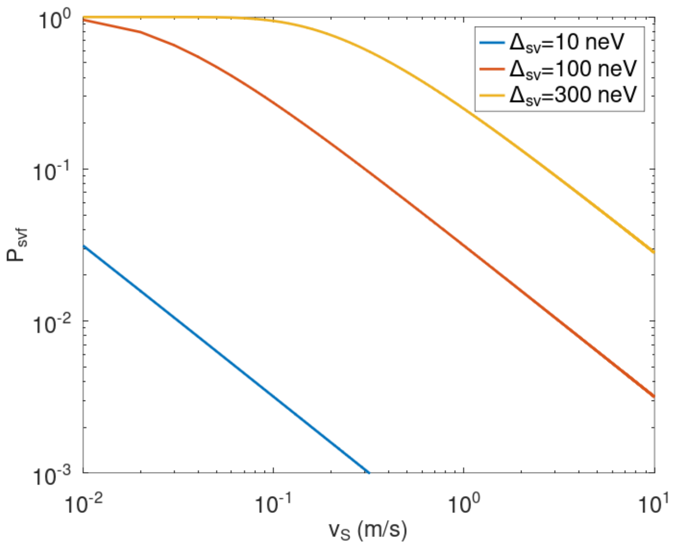

Supplementary Figure 13. Probability of adiabatic spin-valley flip-flop due to shuttling through the spin-valley resonance, Eq. (S17), plotted as a function of shuttling velocity v_S for three values of spin-valley coupling Δ_{sv}. We have used $|\mathrm{d}E_{\mathrm{VS}}/\mathrm{d}x| = 3$ μeV/nm, which is the average value from Fig. 3a in the main text.

This is the region close to a spin-valley resonance defined by $E_z = E_{\mathrm{VS}}$ with $E_Z \approx E_Z^- \approx E_Z^+$. The measured values of Δ_{sv} range from $\approx$ 20 neV [7], through ≈ 50 neV [2] to values in $100 - 150$ neV range measured here and in [8]. It is important to note that due to smallness of these couplings, they are relevant only in very narrow ranges of values of dot positions/shuttling times. Using the $E_{\mathrm{VS}}(d)$ map from Fig. 3a from the main text we obtain an average value of $|\mathrm{d}E_{\mathrm{VS}}/\mathrm{d}x| \approx 3$ μeV/nm, with maximal values measured $\approx$ 15 μeV/nm. The typical range of dot positions across which E_{VS} varies by ≈ 300 neV is thus $\Delta x \approx 0.1$ nm. We can safely neglect the spatial dependence of all the parameters in Hamiltonian from Eq. (S10) except for that of valley splitting, which can be very well approximated by $E_{\mathrm{VS}}(x) = \mathrm{const.} + v_\delta t$, where $v_\delta \equiv (\mathrm{d}E_{\mathrm{VS}}/\mathrm{d}x) \cdot v_S$. We arrive then at the spin-valley coupling Hamiltonian of the Landau-Zener form written in the $\{|-\uparrow\rangle, |+\downarrow\rangle\}$ basis:

$$\hat{H}_{sv} = \frac{1}{4}(E_Z^- - E_Z^+)\mathbb{1} + \begin{pmatrix} \frac{1}{2}v_\delta t & \Delta_{sv} \\ \Delta_{sv}^* & -\frac{1}{2}v_\delta t \end{pmatrix} . \tag{S16}$$

For evolution starting in $|-\uparrow(-t_i)\rangle$ eigenstate at time $-t_i$ fulfilling $|v_\delta t_i| \gg v$, and ending at t_f that also fulfills $|v_\delta t_f| \gg v$, the final occupation of the state with spin and valley indices changed, i.e. the state reached during an **adiabatic** evolution, follows from the Landau-Zener formula

$$P_{svf} = 1 - \exp\left(-2\pi\Delta_{sv}^2/\hbar v_\delta\right) , \tag{S17}$$

where the svf subscript stands for "spin-valley flipped". More precisely, the qubit traversing through a spin-valley resonance evolves as

$$\frac{1}{\sqrt{2}}(|-\uparrow\rangle + e^{i\alpha}|-\downarrow\rangle) \longrightarrow \frac{1}{\sqrt{2}}(a_{svf}|+\downarrow\rangle + b_{svf}|-\uparrow\rangle + e^{i\alpha}|-\downarrow\rangle) , \tag{S18}$$

where a_{svf} and b_{svf} follow from the solution of the Landau-Zener model, i.e $|a_{svf}|^2 = P_{svf}$ and $|b_{svf}|^2 = 1 - P_{svf}$. When $\hbar v_\delta \ll 2\pi\Delta_{sv}^2$ at a given resonance, the evolution is adiabatic, and the final state is given by

$$\frac{1}{\sqrt{2}}\left(|+\rangle + e^{i\alpha}|-\rangle\right) \otimes |\downarrow\rangle , \tag{S19}$$

showing that an adiabatic passage through a spin-valley anticrossing converts the spin superposition into the valley superposition, as noted in [12]. An analogous effect occurs if the incoming electron is in $|+\rangle$ valley state: then the $|+\uparrow\rangle + e^{i\alpha}|+\downarrow\rangle$ state is converted by adiabatic passage through the resonance to $(|+\rangle + e^{i\alpha}|-\rangle) \otimes |\uparrow\rangle$.

in Supplementary Fig. 13, we show how P_{svf} given by Eq. (S17) varies as a function of shuttling velocity for values of spin-valley coupling Δ_{sv} spanning the range of these couplings seen in experiments. The key notion is

that for $\Delta_{sv} \gtrsim 100\,\text{neV}$ the transition between adiabatic and diabatic dynamics upon shuttling across a spin-valley resonance happens for $v_S \sim 1$ m/s. For significantly lower $|\Delta_{sv}|$ the dynamics is expected to be diabatic (i.e. with small probability of spin-valley flip-flop) for $v_S > 0.01$ m/s.

SUPPLEMENTARY NOTE VIII: MECHANISMS OF DECOHERENCE DURING THE SHUTTLING

A: Dephasing in absence of spin-valley resonances and valley excitations

Let us draw attention to an important feature of the dynamics of spin and valley degrees of freedom of the shuttled electron that follow from Hamiltonian (S10). As the shuttling velocity v_S is lowered, off-diagonal terms driving the spin-conserving inter-valley transitions, $\hbar\dot{\phi}$, tend to zero. On the other hand, the spin-valley coupling terms, Δ_{sv}, are independent of shuttling velocity. Consequently, when the shuttled electron encounters the spin-valley resonance, then at the lowest velocities at which the evolution is fully adiabatic, the electron undergoes a spin-valley flip at each resonance. Later we will discuss how such a spin-valley flip changes the nature of dephasing of the qubit, but first let us discuss the dephasing of the qubit that undergoes the evolution that is adiabatic with respect to the valley-changing terms, and no spin-valley resonances are present. Alternatively, if the resonances are present, we assume now that dynamics during passage through them is as close to diabatic as possible, so that the state of the electron is completely unaffected by passage through them.

The dynamics following from Hamiltonian (S10) is then rather trivial: the qubit remains in the instantaneous valley eigenstate, while the spin splitting $E^{-}_{Z,R}(t)$ varies due to electron motion and spatial dependence of g-factor of the qubit (given by $g_{-}(x(t)$ when the electron is in the lower-energy valley state). In this case of purely adiabatic valley dynamics and no influence of spin-valley resonances on the shuttling path, the spin qubit shuttled for time $\tau_S/2$ acquires the relative phase between its two states given by

$$\phi(\tau_S/2) = \int_0^{\tau_S/2} E^{-}_{Z,R}(x(t))\mathrm{d}t \tag{S20}$$

where $E^{-}_{Z,R}(x)$ is the Zeeman splitting for qubit is a dot localized at x. In the experiments in this paper a spin singlet state of the shuttled qubit and an electron left behind in half-filled valley in the stationary L dot is created, and the coherence measurement is done after shuttling the qubit back and performing the Pauli spin blockade readout. Consequently, the phase acquired by the singlet-triplet superposition during back-and forth shuttling that takes the total time τ_S is given by an analogous expression in which $E^{-}_{Z,R}$ is replaced by $\Delta E_Z \equiv E_{Z,L} - E^{-}_{Z,R}$, and shuttling in both directions contributes to the phase. Let us now assume that the electron motion can be well approximated as having constant velocity v_S, with the sign of v_S simply changed on the way back. This leads to an expression equivalent to Eq. (3) in the main text:

$$\phi(\tau_S) = 2\frac{1}{\hbar}\int_0^{\tau_S/2} \Delta E_Z(vt)\mathrm{d}t = \left[\frac{1}{d}\int_0^{d}\frac{1}{\hbar}\Delta E_Z(d)\mathrm{d}x\right]\tau_S \equiv \bar{\omega}(d)\tau_S \equiv 2\pi\bar{f}(d)\tau_S \tag{S21}$$

in which $d = v_S\tau_S/2$, and $\bar{f}(d)$ is the precession frequency averaged over the shuttling distance d.

Hyperfine coupling to very slowly fluctuating nuclei , and low-frequency charge noise leading to slow fluctuations of electron g-factors cause quasi-static (i.e. static at the few microsecond timescale of single shuttling, but changing over the whole time of data acquisition) shifts of Zeeman splittings in the two dots. For the shuttled R dot these shifts are of course dependent on the dot position. Averaging over a Gaussian distribution of these shifts for the static dot contributes a constant $\sigma^2_L/\hbar^2$ to variance of $\tilde{\omega}(d)$. On the other hand, the contribution from the moving dot depends on the shuttling distance d (for derivation see e.g. the supplementary online information of [13]). Assuming that the shift for the shuttled dot is a Gaussian random field with autocorrelation length l_c and Gaussian-shaped autocorrelation function (which applies to the case of $E_{Z,R}$ fluctuations due to nuclear Overhauser fields, see [13]), the resulting variance of the distance-averaged frequency is given by

$$\frac{1}{\hbar^2}\sigma^2(d) = \frac{1}{\hbar^2}\sigma^2_L + \frac{1}{\hbar^2}\sigma^2_S(d;\sigma_R,l_c)\ , \tag{S22}$$

with the variance contributed by the shuttled spin, $\sigma^2_S(d)$, given by

$$\sigma^2_S(d;\sigma_R,l_c) = \sigma^2_R\left[\frac{2l_c^2}{d^2}\left(e^{-d^2/2l_c^2}-1\right) + \frac{\sqrt{2\pi}l_c}{d}\mathrm{Erf}(d/\sqrt{2}l_c)\right]\ , \tag{S23}$$

where σ_R^2 is the typical variance of the spin splitting for the dot positioned within the shuttling channel. Note that for $d \gg l_c$ we have $\sigma_S^2(d) \approx \sqrt{2\pi}\sigma_R^2 l_c/d$. For nuclear Overhauser field noise and Gaussian approximation for the shape of electron's envelope wavefunction l_c is the confinement length of electrons' wavefunction along the shuttling direction, and $l_c \approx 20$ nm. For contribution due to g-factor fluctuations caused by low frequency charge noise we can suspect that the relevant autocorrelation length will be the electric field noise autocorrelation length (estimated to be ~ 100 nm in [14]). The key feature of this result (which requires only that the noise is quasi-static, and is independent of assumptions on the shape of the spatial autocorrelation function of the noise) is however that the decay of the singlet return probability signal oscillation is in this case Gaussian:

$$P_S(\tau_S) = \frac{1}{2} + \frac{1}{2}\cos\left[(\bar{\omega}(d)\tau_S\right]\exp\left[-\left(\frac{\tau_S}{T_{2,L}^*}\right)^2\right]\exp\left[-\left(\frac{\tau_S}{T_2^*(d)}\right)^2\right] \;, \tag{S24}$$

where $T_2^*(d) \equiv \hbar\sqrt{2}/\sigma_S(d)$, and the static dot dephasing time is $T_{2,L}^* \equiv \hbar\sqrt{2}/\sigma_L$. Note that $T_2^*(d)$ depends **only** on the shuttling distance d - it has no dependence on the shuttling velocity v_S with which the path to d and back is traversed. It also does not depend on the number of repetitions of the shuttling, if the cycle of going to d and back is repeated n_{rep} times: in such a case we obtain the above formula with τ_S simply replaced by the total shuttling time given by $\tau \equiv n_{\text{rep}}\tau_S$. Results from shuttling experiments in [13] were well fit with the above formulas, and characteristic dependence $T_2^*(d) \propto \sqrt{d/l_c}$ due to motional narrowing of influence of quasi-static random fields was observed there.

When other processes (which we discuss below) additionally contribute to the decoherence of the shuttled spin, the decay term in Eq. (S24) describing the dephasing of the spin in the static L dot stays the same, while the second Gaussian decay term becomes modified (with changed $T_2^*(d)$) or replaced by a qualitatively distinct term, e.g. exponential decay $\propto \exp(-\tau/T)$.

B: Dephasing due to valley splitting fluctuations activated by the spin-valley flip-flop

Let us focus now on dephasing of the valley superposition state from Eq. (S19). For simplicity we assume that a fully adiabatic dynamics leads to a spin-valley flip-flop that occurs at a single resonance at $x_{sv} < d$, where d is the distance to which the qubit is shuttled, and then the qubit is shuttled back to its starting position while passing through the resonance again. Of course, during the backward shuttling the qubit's $|+\downarrow\rangle$ state get adiabatically mapped back onto $|-\uparrow\rangle$ state.

The relative phase of two states of the valley qubit that exists during shuttling from x_{sv} to d and then back to x_{sv} is sensitive to fluctuations of valley splitting. Furthermore, in the relevant here case of a system initialized in an entangled state involving an electron in valley ν on the L dot and the shuttled electron in the $-$ valley state in the moving R dot, the state existing at $x < x_{sv}$ is given by

$$|\Phi_{in}\rangle = \frac{1}{\sqrt{2}}(|\nu\uparrow,-\downarrow\rangle + e^{i\alpha}|\nu\downarrow,-\uparrow\rangle) \tag{S25}$$

and upon crossing through the spin-valley resonance it is converted to

$$|\Phi_{out}\rangle = \frac{1}{\sqrt{2}}(|\nu\uparrow,-\downarrow\rangle + e^{i\alpha'}|\nu\downarrow,+\downarrow\rangle) \tag{S26}$$

and the energy splitting between the two states is then given by

$$\Delta E = \frac{1}{2}(\nu E_{VL} + E_{Z,L} - E_{VR} - E_{ZR} - \nu E_{VL} + E_{ZL} - E_{VR} + E_{ZR}) = E_{ZL} - E_{VR} \;, \tag{S27}$$

so that the phase superposition state after spin-valley flip-flop of the moving dot is sensitive to fluctuations of the spin splitting in the L dot and of the valley splitting of the R dot.

The phase picked up by the superposition during the shuttling to d and back is then given by

$$\phi(\tau_S) = \frac{1}{\hbar}\left[E_{ZL} - \left(\frac{1}{x_{sv}}\int_0^{x_{sv}} E_{ZR}(x)\mathrm{d}x\right)\cdot\frac{x_{sv}}{d} + \left(\frac{1}{d-x_{sv}}\int_{x_{sv}}^{d} E_{VR}(x)\mathrm{d}x\right)\cdot\frac{d-x_{sv}}{d}\right]\tau_S \equiv \bar{\omega}(x_{sv},d)\tau_S \tag{S28}$$

The crucial thing to note here is that since $E_{VR} \in [1,100]$ μeV, while $|E_{ZL} - E_{ZR}|$ is at most ~ 0.1 μeV at highest considered B fields, the contribution of the integral over E_{VR} to the frequency of singlet-triplet oscillation $\bar{\omega}(x_{sv}, d)$

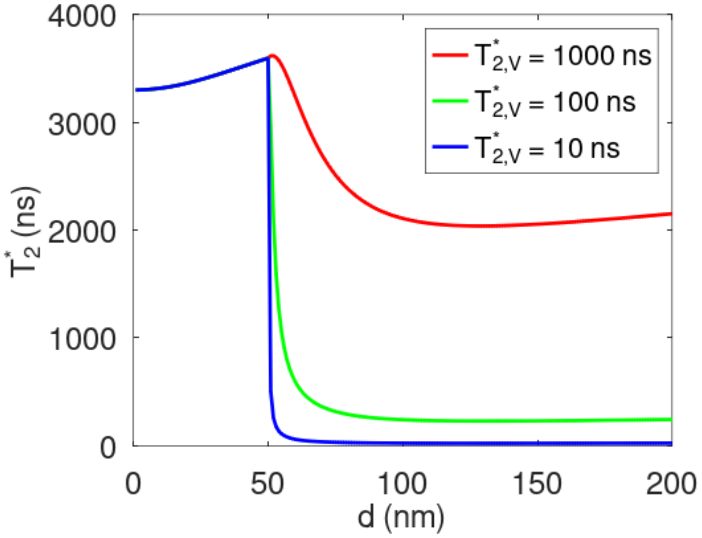

Supplementary Figure 14. Dephasing time T_2^* following from Eqs. (S30) and (S29) characterizing the decay of singlet return probability when the shuttled qubit passes *adiabatically* through a spin-valley resonance at $x_{sv} = 50$ nm when shuttled across distance d and back. The values of rms of spin splitting in both the stationary and the shuttled dot are $\sigma_L = \sigma_R = 0.2$ neV, resulting in $T_2^* \approx 3.3$ μs for a singlet initialized in a static double quantum dot. The superposition of spin states of the shuttled qubit, Eq. (S25), is adiabatically converted into the superposition of valley states, Eq. (S26), upon crossing the spin-valley resonance at x_{sv}, and it then undergoes dephasing due to valley splitting fluctuations until the qubit is shuttled again through the spin-valley resonance on its way back. Three values of rms of valley splitting, σ_V, are considered, corresponding to dephasing times of valley superposition in a static dot given by $T^*_{2,V} = \hbar\sqrt{2}/\sigma_V = 10$, 100, 1000 ns.

strongly dominates over the contribution from integrals over Zeeman splitting as $d - x_{sv}$ distance is increased. When $(d - x_{sv})/d$ becomes larger than the ratio of $|E_{ZL} - E_{ZR}|/E_{VR}$ that is typically $\ll 0.1$, the frequency $\bar{\omega}(x_{sv}, d)$ increases by at at least an order of magnitude. In order to see such a large increase of oscillation frequency when d becomes larger than x_{sv} the $P_S(\tau_S)$ signal should be sampled with frequency orders of magnitude higher than the one used to reconstruct much slower oscillations in the $10 - 100$ MHz range due to Zeeman splitting differences between L and R dots. A signal sampled with too large time delay between mesurement times will look as white noise.

Let us consider now the dephasing of the oscillations. In presence of noise in spin and valley splittings, $\delta E_{Z,L(R)}$ and δE_{VR}, respectively, the calculation of variance of this phase follows the derivation from [13]. We make a natural assumption that $\delta E_{VR}(x)$ is uncorrelated with $\delta E_{ZR}(x)$. Fluctuations of valley splitting in a stationary dot are expected to be caused by low-frequency electric field noise. Noise in out-of-plane electric field affects the overlap between the electron's wavefunction and the Si/SiGe interface (which obviously leads to fluctuations of E_{VS}), while noisy in-plane electric field shifts the dot, leading to random change in valley splitting due to dot-position dependence of E_{VS}. For the dot moving along the x axis the effect of electric fields in z and y direction on valley splitting will be the same, while fluctuating electric fields in x direction will lead to randomness in velocity fluctuations. It is thus reasonable to assume that the valley splitting fluctuations experienced by the moving dot are quite similar to fluctuations for stationary dots. Largest reported dephasing times of valley qubits in such stationary dots are of the order of at most a microsecond [15, 16], and much shorter valley dephasing times were extracted from measurements in Si/SiGe structures from [13] and those used here [1].

While the charge-noise induced rms of valey splitting for a dot at position x, $\sigma_V(x)$, is expected to depend on x, for simplicity we will calculate the valley-splitting-fluctuation induced dephasing by using a single number, σ_V, as a measure of typical magnitude of δE_{VS} fluctuations along the shuttling path. We will also assume that the spatial auto-correlation function of valley splitting fluctuations has a Gaussian form, $\langle \delta E_{\mathrm{VS}}(x) \delta E_{\mathrm{VS}}(x') \rangle \propto \exp\big(-(x - x')^2/2l^2_{c,v}\big)$, where $l_{c,v}$ is the autocorrelation length of valley splitting fluctuations. With these assumptions we can reuse the results of calculation leading to Eq. (S23) for the case of dephasing of valley superposition. We are now dealing with a qubit the phase of which is affected by Overhauser field fluctuations during shuttling from $x = 0$ to $x = x_{sv}$, then by E_V fluctuations during shuttling from $x = x_{sv}$ to $x = d$ and back to $x = x_{sv}$, and finally again by Overhauser field fluctuations during shuttling from $x = x_{sv}$ to $x = 0$. Calcualtion analogous to one leading to Eq. (S23) leads to the

following expression for variance of $\hbar\bar{\omega}(x_{sv}, d)$:

$$\sigma^2(x_{sv}, d) = \sigma_L^2 + \left(\frac{x_{sv}}{d}\right)^2 \sigma_S^2(x_{sv}; \sigma_R, l_c) + \left(\frac{d - x_{sv}}{d}\right)^2 \sigma_S^2(d - x_{sv}; \sigma_V, l_{c,v}) \ , \tag{S29}$$

where $\sigma_S^2(x; \sigma_{R(V)}, l_c(l_{c,v}))$ is given by Eq. (S23). In Supplementary Fig. 14 we plot the resulting dephasing time

$$T_2^*(x_{sv}, d) = \frac{\hbar\sqrt{2}}{\sigma(x_{sv}, d)} \ , \tag{S30}$$

for $\sigma_L = \sigma_R = 0.2$ neV, which corresponds to $T_2^* \approx 3$ μs for a spin singlet in absence of shuttling, and for values of σ_V corresponding to valley superposition dephasing times $T_{2,V}^* \approx 10$ ns, 100 ns, and 1 μs. The value of $x_{cv} = 50$ nm and range of d are chosen to match the experiment at $B = 0.3$ T. For correlation lengths we use $l_c = 20$ nm (as expected based on simulated size of the shuttled dot [13]) and choose $l_{c,v} = l_c$ for simplicity (note that we do not expect these two correlation lengths to have distinct orders of magnitude). The key thing to note is that when $\sigma_V \gg \sigma_{L(R)}$, an adiabatic spin-valley flip-flop leads to very strong dephasing of the singlet return probability.

C: Spin relaxation near the spin-valley relaxation resonance

Spin-valley coupling Δ_{sv} leads to strong mixing of $|-\uparrow\rangle$ and $|+\downarrow\rangle$ states when $|E_{\rm VS} - E_Z| \lesssim 2|\Delta_{sv}|$. This mixing results in enhanced probability of spin relaxation from $\widetilde{|-\uparrow\rangle} = a\,|-\uparrow\rangle + b\,|+\downarrow\rangle$ (with $|a|^2 \approx |b|^2 \approx 1/2$ close to the resonance) eigenstate that is adiabatically connected to $|-\uparrow\rangle$ away from the spin-valley resonance [7, 17]. The spin relaxation rate at such a relaxation hotspot, Γ_s, becomes equal to half of valley relaxation rate, Γ_v, when $|E_{\rm VS} - E_Z| \ll |\Delta_{sv}$, while for $|E_{\rm VS} - E_Z| \gg |\Delta_{sv}|$ we have $\Gamma_s \propto \Gamma_v |\Delta_{sv}|^2/(E_{\rm VS} - E_Z)^2 \ll \Gamma_v$. Consequently, as the value of $E_{\rm VS}$ of the shuttled dot is swept through the spin-valley resonance with rate v_δ, see Eq. (S16), the total probability of spin relaxation at the resonance, p_{sr}, can be upper bounded by $\Gamma_v \Delta t$, where $\Delta t = 4|\Delta_{sv}|/v_\delta$ is the time spent in the vicinity of the relaxation hotspot. As $v_\delta \propto v_S$, the probability of spin relaxation increases with decreasing shuttling velocity v_S.

D: Valley excitation after shuttling through the valley splitting minimum

As discussed in Sec. SUPPLEMENTARY NOTE VI, shuttling through the $E_{\rm VS}$ minimum puts the shuttled electron in a superposition of valley eigenstates (see Eq. S13), while maintaining the superposition state of the spin degree of freedom. Let us note that rather fast (compared to timescales of shuttling) dephasing of valley superpositions, discussed in Sec. SUPPLEMENTARY NOTE VIII B, quickly turns the valley superposition into incoherent statistical mixture of qubit being in the initial valley state (before passage through the $E_{\rm VS}$ minimum) with probability of $1 - Q_v$, and in the other valley eigenstate with probability Q_v, see Sec. SUPPLEMENTARY NOTE VI for discussion of expected magnitude and velocity-dependence of Q_v. During subsequent evolution the spin states occupying the two valleys will undergo precession with distinct frequencies due to finite difference of g-factors for the shuttled electron in two valley eigenstantes resulting in finite difference of Zeeman splittings, $\delta E_{Z,R}(x)$. Shuttling across the valley-splitting minimum acts then as an incoherent beam splitter for the qubit, and the spin phases acquired during subsequent evolution through the two "paths" are distinct. As discussed in [12], if valley relaxation occurs with non-negligible probability during shuttling, it leads to randomization of qubit phase and results in the spin qubit arriving in a partially dephased state to its destination. The presence in the measured signals of multiple frequencies, associated with occupation of both valley states in the stationary and the shuttled dot, means that the valley relaxation is not very efficient on timescales of up to ~ 10 μs that are of relevance for the discussed experiments. This means that for the qubit shuttled across distance d and then subjected to measurement that is not valley selective (as the Pauli Spin Blockade measurement used in the experiment here clearly is), spin precession consisting of contributions with two frequencies would be obtained. While arrival of a qubit in each of two valley states with nonzero probability poses challenges for subsequent processing of quantum information encoded in the spin degree of freedom, this process does not really qualify as spin dephasing. However, when describing the experiments involving multiple there-and-back shuttle cycles before the measurement is done, we need to average over many paths in valley space (due to processes of valley state change occurring with finite probability at multiple times of passages through $E_{\rm VS}$ minima), and leads to averaging over many frequencies, resulting in true dephasing of the signal. This will be discussed in detail in Sec. SUPPLEMENTARY NOTE X B.

SUPPLEMENTARY NOTE IX: DISCUSSION OF EXPERIMENTAL RESULTS: EFFECT OF SINGLE THERE-AND-BACK SHUTTLING THROUGH THE SPIN-VALLEY RESONANCE

In Fig. 3c,f of the main text we see how passage through the spin-valley resonance present at $x_{sv} \approx 50$ nm when $B = 0.3$ T suppresses the amplitude of the singlet-triplet oscillations. Also, as one can most clearly see by focusing on the lower-left corner of Fig. 3c, this suppression of oscillations becomes stronger as the velocity with which the resonance is traversed decreases, with $v_S \sim 0.1$ m/s roughly delineating the boundary between retaining the same order of magnitude of visibility of oscillations and strong suppression of this visibility. Above we have discussed two mechanism that could explain this effect: (1) transition between diabatic and adiabatic passage through the spin-valley resonance as v_S is lowered, with adiabatic passage turning the spin qubit into a valley qubit and making it sensitive to valley splitting fluctuations, and (2) spin relaxation rate enhancement in the vicinity of the spin-valley resonance (the relaxation hotspot effect), leading to spin relaxation probability which scales with time spent in this vicinity, and thus with inverse of shuttling velocity v_S. Let us now discuss the expected significance of these two mechanisms while taking into account the parameters of the system inferred from experiments.

First we consider spin relaxation at the spin-valley resonance. As discussed above, the probability of relaxation is $p_{sr} < 4\Gamma_v |\Delta_{sv}|/v_\delta$, where $v_\delta = |\mathrm{d}E_V(x)/\mathrm{d}x| \cdot v_S$. Using the data on $E_V(x)$ from Fig. 3a of the main text we estimate $|\mathrm{d}E_V(c)/\mathrm{d}x| \approx 10$ μeV/nm at $x_{sv} = 50$ nm. Taking $\Delta_{sv} = 300$ neV as the estimate of maximal spin-valley coupling expected in Si/SiGe structures, we obtain that p_{sr} can become ~ 0.1 at $v_S \approx 0.1$ m/s if the valley relaxation rate is $\Gamma_v \geq \mathrm{ns}^{-1}$, i.e. the valley relaxation time is < 1 ns. This value is orders of magnitude shorter than the valley relaxation times (or spin-valley relaxation times near the hotspots) reported in literature [7, 17, 18]

Let us probe the issue of minimal expected valley relaxation time a bit deeper. The valley relaxation rate, Γ_v, strongly depends on E_V, as $\Gamma_v \propto E_V^7$ [12], and also on the interface roughness at a given dot position, as this roughness activates the valley-orbit coupling [19] that is necessary for valley relaxation. It is thus impossible to give even an order-of-magnitude estimate of Γ_v in a sample in which E_V spans almost two orders of magnitude. Let us however note that calculations done for a particular model of interface roughness (a single monoatomic step) gave valley relaxation times $\tau_v = 1/\Gamma_v > 1$ ms for $E_V < 100$ μeV [12]. Since at $x_{sv} = 50$ nm we have $E_V(x_{sv}) \approx 35$ μeV, it seems that it is rather improbable that valley relaxation time at this x_{sv} could be 9 orders of magnitude larger than the one calculated in [12] for any possible realization of atomic disorder at the interface.

Finally, let us note that the presence of multiple frequencies of singlet return probability oscillations in Figs. 3e-g strongly suggest that excited valley states in both the stationary and the shuttled dot are appreciably occupied during the whole shuttling time that reaches ~ 1 μs. This clearly shows that sub-microsecond valley relaxation times are not expected anywhere along the shuttling channel. Having $\tau_v \sim 1$ ns at $x = x_{sv}$, so that the spin relaxation at this point becomes fast enough to bring a large change in singlet return probability signal when the neighbourhood of x_{sv} is visited for $\Delta t \sim 1$ ns, is thus highly improbable.

This leaves us with the mechanism of diabatic-to-adiabatic change of character of electron spin and valley dynamics during shuttling though the spin-valley resonance, as an explanation of suppression of singlet-triplet coherence when v_S drops below ~ 0.1 m/s. As discussed in Sec. SUPPLEMENTARY NOTE VIII B, adiabatic passage through the resonance converts a spin superposition into a valley superposition, and exposes the coherence of the superposition to valley splitting fluctuations.

In Sec. SUPPLEMENTARY NOTE VIII B we have discussed the dephasing for shuttling to $d > x_{sv}$ and back for adiabatic dynamics thorugh the resonance: the spin qubit was converted into a valley qubit at x_{sv}, propagated as such to d and back to x_{sv}, and then was adiabatically converted back to a spin qubit. In order to capture the behavior shown in Fig. 3c,f of the main text we have to account for the degree to which the spin-valley dynamics is adiabatic at given v_S. This means that the state of the spin qubit approaching the spin-valley resonance for the first time transforms upon the passage as in Eq. (S19) where the amplitudes a_{svf} and b_{svf} are obtained by solving the Landau-Zener model from Eq. (S16) for given value of Δ_{sv} and valley splitting sweep rate v_δ that is related to the real space shuttling velocity by $v_\delta \equiv (\mathrm{d}E_{\mathrm{VS}}/\mathrm{d}x) \cdot v_S$. The state after the passage experiences dephasing due to spin splitting and valley splitting fluctuations, and it undergoes the second Landau-Zener evolution during the backwards shuttling through the spin-valley resonance. The full theory of spin-valley dynamics and dephasing should thus account for possibility of Landau-Zener-Stueckelberg-Majorana (LZSM) interference due to two coherent "beam splitting" events, with partial dephasing of superposition of three relevant states happening between them. In order to simplify the model to capture the key effect expected when dephasing of valley superposition is much stronger than that of spin superpositions, we assume that any intervalley coherence is simply lost during shuttling from x_{sv} to d and back to x_{sv}. This means that after the full shuttling cycle, the oscillations of singlet return probability are due to events in which a diabatic path was taken through the anticrossing on way both "there" and "back" (which happen with probability $|b_{svf}|^4$), with the dephasing of these oscillations caused by Zeeman splitting fluctuations only. All the other paths (adiabatic on way "there", diabatic on way "back", etc) result in either an arrival of a polarized tripled, or an arrival of completely dephased superposition of a singlet an unpolarized triplet. Consequently, the oscillating part of the

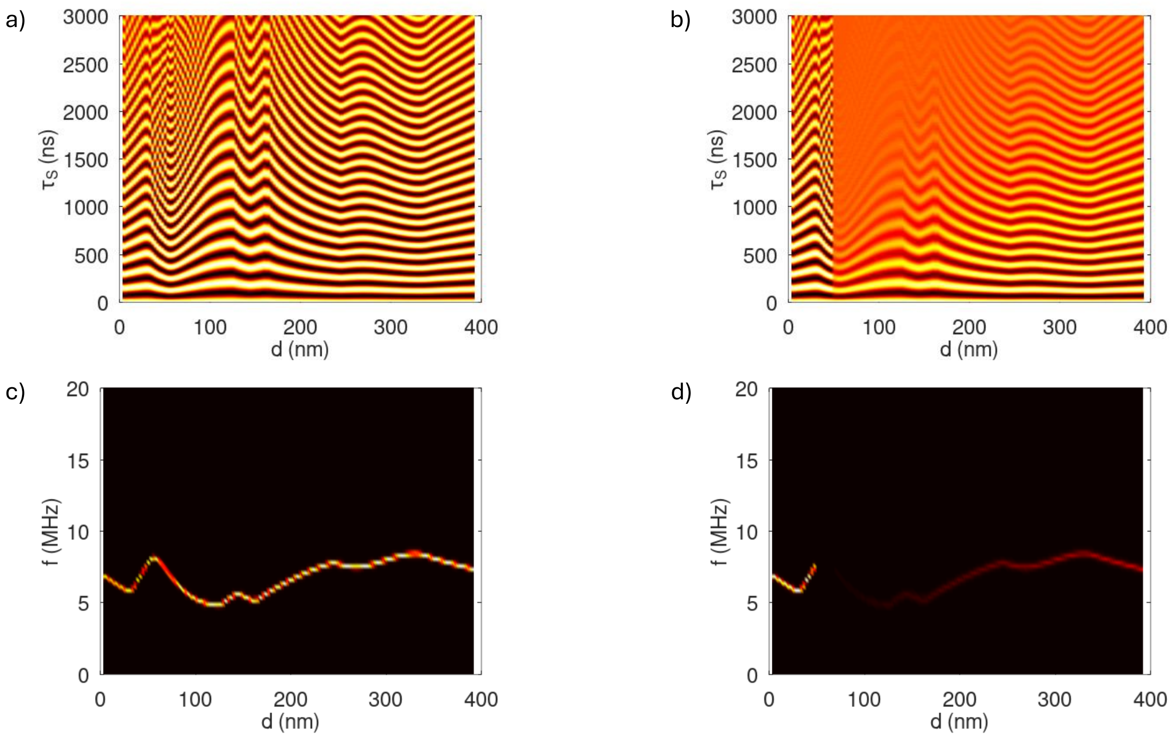

Supplementary Figure 15. (a) Simulation of $P_S(d, \tau_S)$ signal at $B = 0.3$ T using Eqs. (S24) and (S23), with $\delta g(x)$ data extracted from experiments, and $T_2^*(d)$ time following from assuming static single spin qubit $T_2^* = 4.66$ μs in each of the dots (due to hyperfine coupling to nuclei and g-factor fluctuations due to low-frequency electric field noise). (b) An analogous simulation, but taking into account the effect of passage through spin-valley resonance at $x_{sv} = 50$ nm, assuming $\Delta_{sv} = 300$ neV and very fast dephasing of valley superpositions. (c,d) Corresponding Fourier transforms with respect to τ_S. Note the similarity between panels (b) and d here and corresponding panels in Fig. 3 in the main text. Note that the second frequency visible in the signal in Fig. 3f is not included in the model used here, as we focus on effects of passage through a spin-valley resonance for any of the singlets that are occupied in the experiment.

singlet return probability is given by

$$P_S(\tau_S) \propto |b_{svf}|^4 \cos\left[(\bar{\omega}(d)\tau_S\right] \; \exp\left[-\left(\frac{\tau_S}{T_2^*(d)}\right)^2\right] \tag{S31}$$

with $\bar{\omega}(d)$ and $T_2^*(d)$ given in Sec. SUPPLEMENTARY NOTE VIII A, and

$$|b_{svf}|^2 = \exp\left(-2\pi\Delta_{sv}^2/\hbar v_\delta\right) \; . \tag{S32}$$

In Supplementary Fig. 15b we plot the signal given by the above formula assuming $\Delta_{sv} = 300$ neV, and $|\mathrm{d}E_{\mathrm{VS}}/\mathrm{d}x| = 10$ μeV/nm. in Supplementary Fig. 15d we show the Fourier transform of this result with respect to τ_S, and we see a clear qualitative similarity to results shown in Fig. 3f in the main text.

SUPPLEMENTARY NOTE X: DISCUSSION OF EXPERIMENTAL RESULTS: MULTIPLE PASSAGES THROUGH VALLEY SPLITTING MINIMUM AND SPIN-VALLEY RESONANCES

Let us discuss now the decoherence caused by shuttling the qubit n times over distance λ and back, while passing through a valley splitting minimum and/or a spin-valley resonance. The data from Fig. 4a and Table I of the main paper show that when no valley splitting minima with $E_{\mathrm{VS}}^{\mathrm{min}} \lesssim 10$ μeV, and no spin-valley resonances are encountered on the shuttling path, the spin dephasing follows Gaussian decay with no significant v_S dependence of $T_2^*(\lambda)$ time, as previously reported [13]. This is the expected result when dephasing is due to quasi-static fluctuations of spin splitting, see [13] and Sec. SUPPLEMENTARY NOTE VIII A above. On the other hand, as the other panels of Fig. 4

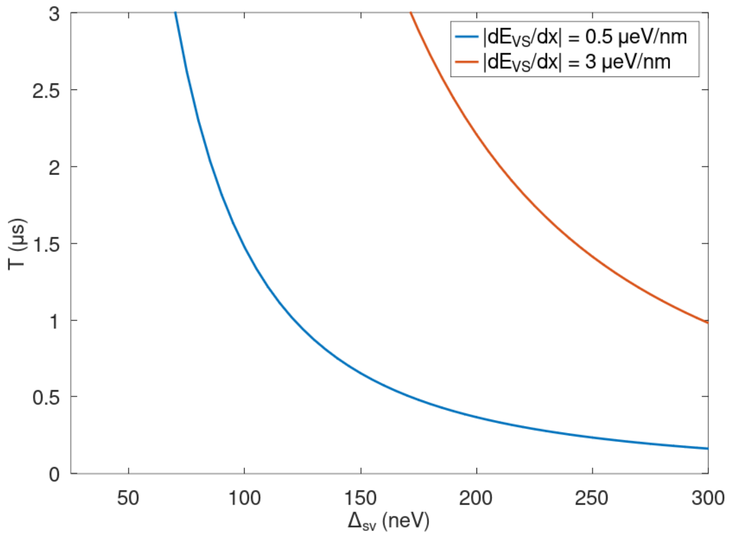

Supplementary Figure 16. Decoherence time T from Eq. (S37) due to multiple passages through a spin-valley resonance, plotted as a function of the spin-valley coupling Δ_{sv} for two values of valley splitting gradient at the resonance.

and Table I from the main paper show, shuttling through two valley splitting minima with $E^{\min}_{\mathrm{VS}} \lesssim 10$ μeV at $d \approx 232$ and 245 nm (and through two spin-valley resonances at $B = 40$ mT) leads to coherence decay that is better fit by an exponential, i.e. having the envelope of the oscillations damped as $e^{-\tau/T}$, with the decoherence time T decreasing with shuttling velocity v_S and magnetic field B increasing. Let us analyze now how multiple passages through the valley splitting mimum and/or the spin-valley resonance can lead to exponential decay of coherence, and what coherence times can be expected taking into account the typical parameters in the measured samples.

A: Passages through a spin-valley resonance

First, we generalize the model from Sec. SUPPLEMENTARY NOTE VIII B describing qubit dephasing due to shuttling across the spin-valley resonance, to the case of multiple there-and-back passages. We begin by noting that for $v_S \geq 5.6$ m/s used in there-and-back shuttling experiments, the probability of spin-valley flip-flop, P_{svf} given by Eq. (S17), is $\ll 1$ for $\Delta_{sv} \leq 300$ neV, and for $|\mathrm{d}E_{\mathrm{VS}}/\mathrm{d}x| > 0.5$ μeV/nm. As the average of $|\mathrm{d}E_{\mathrm{VS}}/\mathrm{d}x|$ shown in Fig. 3a of the main text is ≈ 3 μeV/nm, we can expect that shuttling across the spin-valley resonance with v_S above a few m/s results in mostly diabatic spin-valley dynamics, with events of spin-valley flip-flop occuring with $P_{svf} \propto 1/v_S \ll$. More specifically, for the two spin-valley resonances encountered at $B = 40$ mT and $d \in [230, 240]$ (see Fig. 4e of the main text), we have $|\mathrm{d}E_{\mathrm{VS}}/\mathrm{d}x| \approx 3$ and 0.5 μeV/nm, so the assumption of $P_{svf} \ll 1$ almost certainly holds for these resonances.

As above we assume that each event of spin-valley flip-flop leads to complete loss of coherence of the qubit. The decay of the envelope of the oscillating part of the singlet return probability signal, for n there-and-back passages is then given by

$$W_n = (1 - P_{svf})^{2n} = \exp\left(2n \ln[1 - P_{svf}]\right) \approx e^{-2nP_{svf}} \ , \tag{S33}$$

where we have used $\ln(1-x) \approx -x + O(x^2)$ for $x \ll 1$. Rewriting W_n as a function of total shuttling time $\tau = n\tau_S$, where $\tau_S = 2\lambda/v_S$ is the time of shuttling to λ and back, we obtain

$$W(\tau) = e^{-\tau/T} \ , \tag{S34}$$

where the decoherence time is

$$T = \frac{\tau_S}{2P_{svf}} = \frac{\lambda}{v_S P_{svf}} \ , \tag{S35}$$

and after using

$$P_{svf} \approx \frac{2\pi\Delta_{sv}^2}{\hbar v_S |\mathrm{d}E_{\mathrm{VS}}/\mathrm{d}x|} \ , \tag{S36}$$

we finally arrive as

$$T \approx \frac{\hbar\lambda |\mathrm{d}E_{\mathrm{VS}}/\mathrm{d}x|}{2\pi\Delta_{sv}^2} \ . \tag{S37}$$

The key things to note is the lack of dependence of this T on v_S, and a weak dependence of B field, as long as the resonance is actually present (i.e. $2\mu_B B$ is larger than E_{VS}^{min}). The first result follows from applicability of Landau-Zener model to the description of the spin-valley flip-flop, resulting in $P_{svf} \sim 1/v_S$, and consequently an approximate cancellation of v_S dependence from the denominator of Eq. (S35). As for the second, the implicit dependence of T on B comes from dependence of $|\mathrm{d}E_{\mathrm{VS}}/\mathrm{d}x|$ and Δ_{sv} on the value of valley splitting at which the spin-valley resonance, occurs, $E_{\mathrm{VS}} = 2\mu_B B$.

in Supplementary Fig. 16, we show the coherence times T due to this mechanism plotted as function of Δ_{sv} for two values of the E_{VS} gradient, corresponding to the two resonances that occur at $d \approx 232$ nm when $B = 40$ mT. For the resonance at which the gradient is smaller, we obtain $T < 1$ μs for $\Delta_{sv} > 100$ neV. While this shows that the spin-valley flip-flop could explain the observed decrease of coherence time of the shuttled dot from >4 μs to ≈ 2.6 μs at the B field is raised from 10 to 40 mT at $v_S = 5.6$ m/s, the fact that T at 40 mT exhibits visible v_S dependence, suggestes that the spin-valley flip-flop is not the process that dominates the observed decoherence. The fact that the T fitted to the experimental results also visibly vary between $B = 10$ and 20 mT (i.e. in the field range in which there is no spin-valley resonance, and no contribution to T from the above-discussed mechanism), additionally suggests that the spin-valley related mechanism, while in principle relevant in range of Δ_{sv}, v_S, and $|\mathrm{d}E_{\mathrm{VS}}/\mathrm{d}x|$ encountered in experiments, does not explain the magnetic field and shuttling velocity dependence of the observed T.

B: Passages through a valley splitting minimum

Let us thus move on to the mechanism associated with intervalley excitation - and the resulting change of g-factor of the shuttled spin - that occurs when a valley splitting minimum is traversed with large enough v_S.

As discussed in Sec. SUPPLEMENTARY NOTE VI, shuttling through a minimum of $E_{\mathrm{VS}}(x)$ results in finite probability Q_v of valley excitation. Example calculations from that Section have shown that when $E_{\mathrm{VS}}^{min} < 25$ μeV, we can expect $Q_v \sim 0.01 - 0.1$ for $v_S \sim 10$ m/s. Furthermore, when the shape of $E_{\mathrm{VS}}(x)$ near the minimum at x_m is symmetric, and the dependence of $E_{\mathrm{VS}}(x)$ is close to a linear one in wide range of x not too close to x_m, then we can count that with sizable probability the Landau-Zener model calculation to give a good approximation to Q_v (especially at lowest values of E_{VS}^{min}, say below 10 μeV). Note however that is observation, based on simulations of ~ 10 trajectories of $\Delta(x)$, should be taken with a grain of salt, as it is known that trajectories of Δ can lead shapes of $E_{\mathrm{VS}}(x)$ in perfect agreement with the L-Z model, while giving results for $Q_v(v_S)$ that are in qualitative disagreement with this model [4]. However we can set this issue aside when focusing on data shown in Fig. 4 of the main text, as the measured $E_{\mathrm{VS}}(x)$ for $x \in [220, 250]$ nm shown there exhibits two minima separated by ≈ 15 nm (i.e. a distance similar to the autocorrelation length of valley coupling). As illustrated in the fifth row from of Fig. 12, such a shape of $E_{\mathrm{VS}}(x) = 2|\Delta(x)|$ is most probably caused by a trajectory of $\Delta(x)$ in complex plane that makes a loop, or at least a sizable fraction of a loop, around zero. As shown in that figure, this can result in $Q_v(v_S)$ dependence that shows qualitative deviations (e.g. plateaus, possibly even oscillations) from the Landau-Zener result for $Q_v(v_S)$. More investigations into the statistical properties of $Q_v(v_S)$ curves for many realizations of valley coupling disorder would be necessary to discuss more quantitatively the probabilities that a given feature in $E_{\mathrm{VS}}(x)$ corresponds to a particular $Q_v(v_S)$ dependence. Here we will take a "black box" approach to the valley excitation probability at a double minimum of $E_{\mathrm{VS}}(x)$: we will do a model calculation of the spin dephasing due to multiple passages through a region in which the valley dynamics is expected to be partially nonadiabatic, and we will check if we can reproduce the observed decoherence time that changes from more that 4 μs at $B = 10$ to ≈ 2.6 μs at 40 mT when $v_S = 5.6$ m/s, and then comment on the suppression of this time down to ≈ 1.5 μs when v_S is raised to 22.4 m/s.

We consider repeated shuttling n_{rep} times over distance λ and back, with time of one there-and-back shuttle given by $\tau_S = 2\lambda/v_S$, and total shuttling time $\tau \equiv n_{rep}\tau_S$. When the shuttled qubit is in valley ν (with $\nu = \pm 1$, and $\nu = -1$ denoting the ground valley state), the spin phase picked up during one cycle of shuttling is given by Eq. (S21) with valley-specific $\bar{\omega}_\nu(\lambda)$, i.e. this phase is $\Delta\phi_\nu = \bar{\omega}_\nu(\lambda)\tau_S$. We focus now on a single minimum of $E_{\mathrm{VS}}(d)$ at $d = d_e$, at which a change of valley state that results in change of precession frequency of the shuttled qubit, occurs with probability Q_v. We assume that $Q_v \ll 1$, so that after a valley state change the qubit spends typically $\ll 1$ cycles in the new state. In this limit we can ignore the fact that valley flipping events happen at discrete times (of passage through d_e either on the way to λ, or on the way back), and set up a model in which the valley flipping occurs with rate

$$\gamma \equiv \frac{2Q_v}{\tau_S} = \frac{Q_v v_S}{\lambda} \ . \tag{S38}$$

Let us write the valley-dependent shuttling frequency $\bar{\omega}_\nu$ as

$$\bar{\omega}_\nu = \bar{\omega}_0 + \delta\bar{\omega}_\nu \ , \tag{S39}$$

and observe that the phase acquired during one realization of the initialization-shuttling-readout experiment can be approximated by

$$\phi(\tau) = (\bar{\omega}_0 + \bar{\omega}_{av})\tau + \delta\bar{\omega}\int_0^\tau \xi(t)\mathrm{d}t \ , \tag{S40}$$

where

$$\bar{\omega}_{av} = \frac{1}{2}(\delta\bar{\omega}_+ + \delta\bar{\omega}_-) \ , \tag{S41}$$

$$\delta\bar{\omega} = \frac{1}{2}(\delta\bar{\omega}_+ - \delta\bar{\omega}_-) \equiv \overline{\delta g}\mu_B B \ , \tag{S42}$$

where $\overline{\delta g}$ is is the difference of g-factors in two valleys averaged over the distance λ, and $\xi(t)$ is a realization of Random Telegraph Noise process, with ξ switching between $\xi = 1$ and $\xi = -1$ with rate γ. Averaging of this phase over realizations of the stochastic process $\xi(t)$ is thus equivalent to calculation of dephasing of the qubit the splitting of which is affected by random telegraphic noise (RTN). This problem has a known solution [20–22] that reads

$$W(\tau) \equiv \langle e^{-i\phi(\tau)}\rangle = e^{-i(\bar{\omega}_0+\bar{\omega}_{av})\tau}e^{-\gamma\tau}\left(\cosh\gamma\mu\tau + \frac{\gamma - ip\delta\bar{\omega}}{\gamma\mu}\sinh\gamma\mu\tau\right) \tag{S43}$$

where $\langle ...\rangle$ denotes the average over the realizations of noise ξ, $p = -1$ $(+1)$ if the evolution starts with the shuttled qubit in $-$ $(+)$ valley state, and $\mu \equiv \sqrt{1 - \delta\bar{\omega}^2/\gamma^2}$.

The key features of the above expression for dephasing factor $W(\tau)$ are the following: When $\gamma \ll \delta\bar{\omega}$ we are in the so-called strong coupling regime, in which the typical random phase acquired after a single valley-flipping event is $\gg 1$, and its variance is of the same order of magnitude. The characteristic decay timescale is then simply γ^{-1}: the envelope of decay of is approximately exponential, $|W(\tau)| \approx \exp(-\tau/T_S)$, with the strong-coupling decay time $T_S = \gamma^{-1}$. Note that this was the limit in which the valley-excitation-induced dephasing of the shuttled spin was considered in [12]. On the other hand, when $\gamma \gg \delta\bar{\omega}$ we are in the so-called weak coupling regime, in which multiple valley flips occur before dephasing becomes significant, each random phase contribution is $\ll 1$, and the signs of these contributions are random, so that the total random phase evolves with τ as a random walk. We are then in the motional narrowing regime: the above expression for $W(\tau)$ is then approximated as

$$W_{weak}(\tau) \approx e^{-i(\bar{\omega}_0+\bar{\omega}_{av})\tau}e^{-\tau/T_W} \ , \tag{S44}$$

in which the weak-coupling timescale of exponential decay is

$$T_W = \frac{2\gamma}{\delta\bar{\omega}^2} \ . \tag{S45}$$

For fixed $\delta\bar{\omega}$ (i.e. fixed B field and maximum shuttling distance λ), the characteristic decay timescale T of qubit's phase is thus a non-monotonic function of $\gamma = Q_v v_S/\lambda$, with the minimum dephasing time given by

$$T_{min} \approx \frac{2}{\delta\bar{\omega}} \ , \tag{S46}$$

which is obtained when $\gamma \approx \delta\bar{\omega}$.

The measurement of T as function of B and v_S for shuttling channel at $y = 0$ are shown in Fig. 4 of the main text, and fitted T timescales of exponential decay fits are given in Table I there. The two visible trends are: (1) decrease of T with increasing B at $v_S = 5.6$ m/s, and (2) further decrease of T with increasing v_S at $B = 40$ mT. in Supplementary Fig. 17, we show a plot of $T(\gamma)$ (defined by $|W(T)| = 1/e$) for three values of magnetic field. The average g-factor intervalley difference used in the calculation is chosen to quantitatively reproduce the result (1) when $\gamma \approx 0.5$ μs^{-1}, corresponding to $Q_v \approx 0.015$, while allowing for further decrease of T when $\gamma = Q_v v_S/\lambda$ is increased. in Supplementary Fig. 18 we show the results for the dephasing factor $|W(\tau)|$ calculated with these parameters for $B = 10, 20, 40$ mT. Note that at higher values of B, when we enter the strong coupling regime, the envelope of the coherence signal exhibits plateaus at $\tau \approx k\pi/\delta\bar{\omega}$ (with integer k) characteristic for dephasing due to RTN.

The last experimental result to be be qualitativel reproduced is the decrease of T at $B = 40$ mT as v_S is increased from 5.6 to 22.4 m/s. In order to model this we need to know the shuttling velocity dependence of $Q_v(v_S)$, in order

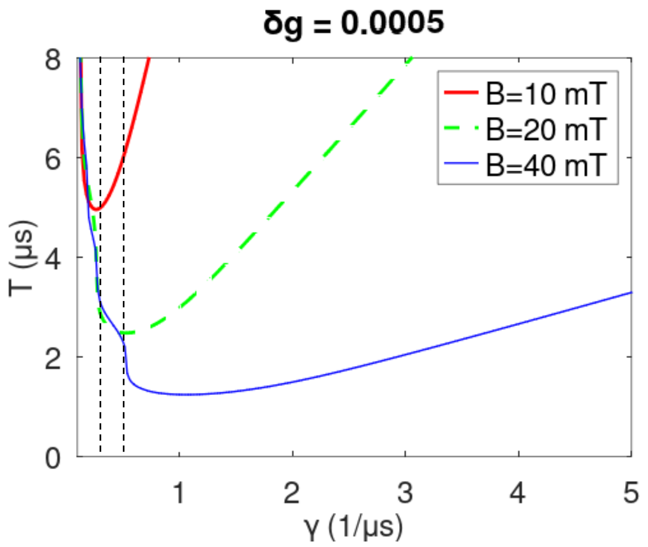

Supplementary Figure 17. A plot of approximate dephasing time $T(\gamma)$ due to multiple passages through $E_{\rm VS}$ minimum resulting in valley state changes occuring with probability Q_v per passage. δg is the difference of valley-dependent g-factors averaged over the shuttling length λ. The valley flip rate is $\gamma = Q_v v_S/\lambda$. For γ in the range between the two vertical dashed lines, increasing B from 10 to 20 mT leads to a suppression of T from > 4 μs to ≈ 2 μs, while the subsequent increase of B to 40 mT leads to a much smaller decrease of T. This reproduces the measured magnetic field dependence of T for $v_S = 5.6$ m/s, see Table I of the main text.

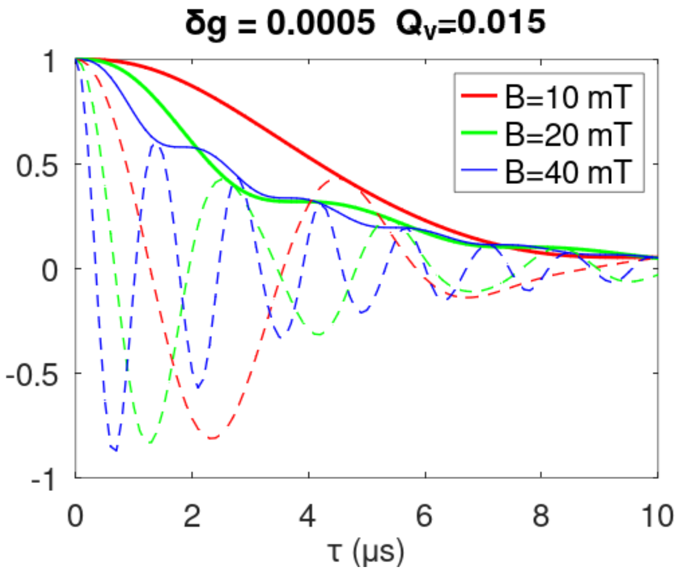

Supplementary Figure 18. Coherence decay from Eq. (S43) calculated for $\delta\bar{\omega}$ used in Supplementary Fig. 17, with $v_S = 5.6$ m/s and $Q_v = 0.015$. Solid lines: envelope of coherence function, $|W(\tau)|$; dashed lines are Re$W(\tau)$ with average g-factor determining the magnetic field dependence of $\bar{\omega}_0 + \bar{\omega}_{av}$ chosen to reproduce the measured oscillation frequency dependence on the magnetic field.

to relate γ to v_S. Clearly, if Q_v strongly increases with v_S, as in the Landau-Zener model in the low-velocity regime, in which $Q_v \ll 1$, even a two-fold increase of v_S will push $\gamma \propto v_S Q_v(v_S)$ to value deep in the weak coupling regime, resulting in **increase** of coherence time. On the other hand, at v_S high enough that the dynamics is approximately diabatic, we expect Q_v to approach a constant $Q_{v,diab}$. It is crucial to take now into account two things.

First, for trajectories of $\Delta(x)$ that correspond to local minima of $E_{\rm VS}(x)$ (see Section SUPPLEMENTARY NOTE VI), we **do not** expect $Q_{v,diab} = 1$, as it should be given by overlap squared of the initial lower (higher) energy valley eigenstatate and the final higher (lower) energy eigenstate. For $\Delta(x)$ trajectories shown in Supplementary Fig. 12

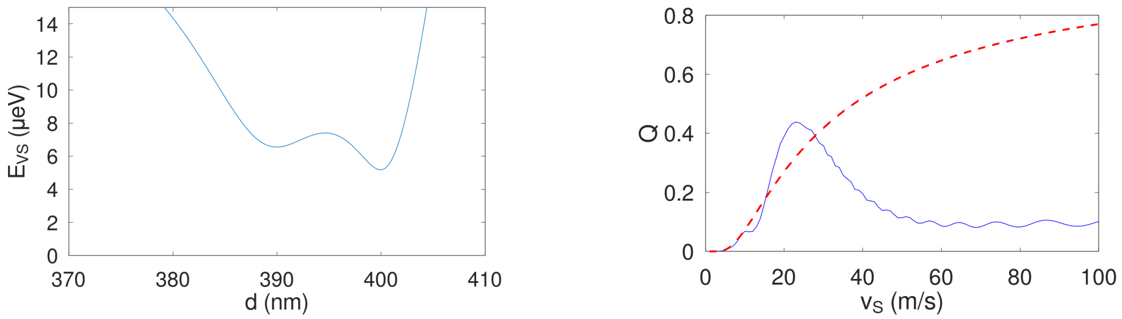

Supplementary Figure 19. Using $\Delta(x)$ from the fifth row of Fig. 12 downscaled by a factor of 2 we obtain the $E_{\rm VS}(d)$ dependence similar shown in the left panel, which has not only the qualitatively similar double-minmum shape as the one encountered in the experiment, but it also has the minimal $E_{\rm VS}$ of about 5 μeV. The valley flip probability Q_v for shuttling through such a double minimum of valley splitting is shown in the right panel, in which the solid blue line is the numerical calculation, and the red dashed line is the Landau-Zener approximation obtained in the way described in Sec. SUPPLEMENTARY NOTE VI.

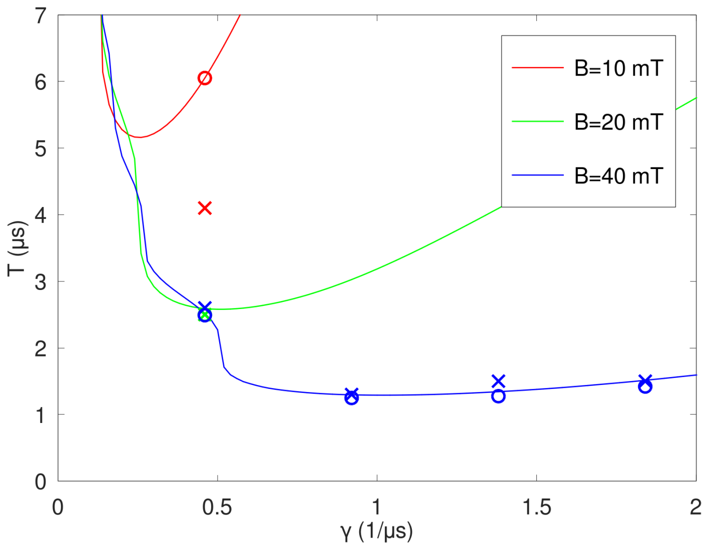

Supplementary Figure 20. Calculated $T(\gamma)$ for $B = 10$, 20, 40 mT with $Q_v = 0.023$ and $\delta\bar{\omega} = 4.8 \cdot 10^{-4} \mu_B B$. Measured values at corresponding v_S (determining $\gamma = Q_v v_S/\lambda$) are marked with crosses, with colors marking the B field. The circles are the theoretical values.

these overlaps, and thus the values of $Q_{v,diab}$ are not equal to 1; for example for the fifth trajectory shown there that corresponds to a double minimum of $E_{\rm VS}(x)$, Q_v saturates at ≈ 0.1 for $v_S > 100$ m/s. After dividing the value of valley coupling for the fifth trajectory of Δ, so that we obtain the minimum values of $E_{\rm VS}$ closer to the ones measured at $d_e = 230 - 240$ nm, we obtain the $Q_v(v_S)$ dependence shown in Supplementary Fig. 19

Second, the above discussed model of dephasing still applies, when $Q_{v,diab} \approx 1$. In this regime it is the lack of valley flip that is a rare event that stochastically perturbs the deterministic evolution, only now this evolution involves valley state change at every passage through d_e. After changing Q_v to $1 - Q_v$, and appropriately redefining $\delta\bar{\omega}$, all the above formulas and discussions apply to this case of almost-diabatic evolution.

These two observations, together with the previously made observation (see Sec. SUPPLEMENTARY NOTE VI) that $Q_v(v_S)$ can exhibit plateaus as a function of v_S when shuttling through a double minimum of $E_{\rm VS}(x)$, show that the assumption that Q_v is approximately velocity-independent is not unreasonable when dealing with shuttling through a valley splitting minimum with $E_{\rm VS}^{min} \leq 5$ μeV.

Coming back to the results shown in Supplementary Fig. 17: with Q_v that does not depend on v_S (at least in the considered range of velocities), or, even better, one that is decreasing with v_S in such a way that γ increases sub-linearly with v_S, we can obtain T decreasing down to the minimal value of ≈ 1 μs. The model in which we assume $\overline{\delta g} \approx 5 \cdot 10^{-4}$ and assume that $Q_v \approx 0.015 - 0.025$ is v_S-independent, is thus in semi-quantitative agreement

with the observations.

In order to quantify the degree of agreement more precisely, we have performed fits of the T times following from the above formulas for $W(\tau)$, using $\overline{\delta g}$ and Q_v as fitting parameters, and assuming that Q_v does not depend on the shuttling velocity. We have not used the measured T for $B = 10$ mT in the fitting, as we suspect that this value is in fact determined by the B- and v_S-independent dephasing of the stationary spin. The best fit was obtained for $Q_v = 0.023 \pm 0.003$ and $\overline{\delta g} = (4.8 \pm 0.3) \cdot 10^{-4}$, and the resulting calculated values of T (including the $B = 10$ mT point, for which the fitted value is larger than the measured one, consistently with our assumption that dephasing time of the shuttled dot at this field is > 4 μs) are shown in Supplementary Fig. 20.

[1] Cywinski, Ł. *et al.*. Singlet-triplet oscillations in multivalley Si quantum dots (2026). In preparation.
[2] Volmer, M. *et al.* Mapping of valley splitting by conveyor-mode spin-coherent electron shuttling. *npj Quantum Inf.* **10**, 61 (2024).
[3] Losert, M. P. *et al.* Strategies for enhancing spin-shuttling fidelities in Si/SiGe quantum wells with random-alloy disorder. *PRX Quantum* **5**, 040322 (2024).
[4] Lima, J. R. F. & Burkard, G. Partial landau-zener transitions and applications to qubit shuttling. *Phys. Rev. B* **111**, 235439 (2025).
[5] Ruskov, R., Veldhorst, M., Dzurak, A. S. & Tahan, C. Electron g-factor of valley states in realistic silicon quantum dots. *Phys. Rev. B* **98**, 245424 (2018).
[6] Woods, B. D., Losert, M. P., Joynt, R. & Friesen, M. g-factor theory of Si/SiGe quantum dots: spin-valley and giant renormalization effects. *arXiv:2412.19795* (2024). Preprint at https://arxiv.org/abs/2412.19795.
[7] Yang, C. H. *et al.* Spin-valley lifetimes in a silicon quantum dot with tunable valley splitting. *Nat. Commun.* **4**, 2069 (2013).
[8] Hwang, J. C. C. *et al.* Impact of g-factors and valleys on spin qubits in a silicon double quantum dot. *Phys. Rev. B* **96**, 045302 (2017).
[9] Paquelet Wuetz, B. *et al.* Atomic fluctuations lifting the energy degeneracy in Si/SiGe quantum dots. *Nat. Commun.* **13**, 7730 (2022).
[10] Losert, M. P. *et al.* Practical strategies for enhancing the valley splitting in Si/SiGe quantum wells. *Phys. Rev. B* **108**, 125405 (2023).
[11] Lima, J. R. F. & Burkard, G. Interface and electromagnetic effects in the valley splitting of Si quantum dots. *Mater. Quantum Technol.* **3**, 025004 (2023).
[12] Langrock, V. *et al.* Blueprint of a scalable spin qubit shuttle device for coherent mid-range qubit transfer in disordered Si/SiGe/SiO_2. *PRX Quantum* **4**, 020305 (2023).
[13] Struck, T. *et al.* Spin-EPR-pair separation by conveyor-mode single electron shuttling in Si/SiGe. *Nat. Commun.* **15**, 1325 (2024).
[14] Yoneda, J. *et al.* Noise-correlation spectrum for a pair of spin qubits in silicon. *Nat. Phys.* **19**, 1793 (2023).
[15] Cai, X., Connors, E. J., Edge, L. F. & Nichol, J. M. Coherent spin–valley oscillations in silicon. *Nat. Phys.* **19**, 386 (2023).
[16] Jock, R. M. *et al.* A silicon singlet–triplet qubit driven by spin-valley coupling. *Nat. Commun.* **13**, 641 (2022).
[17] Huang, P. & Hu, X. Spin relaxation in a si quantum dot due to spin-valley mixing. *Phys. Rev. B* **90**, 235315 (2014).
[18] Hollmann, A. *et al.* Large, tunable valley splitting and single-spin relaxation mechanisms in a Si/Si_xGe_{1-x} quantum dot. *Phys. Rev. Appl.* **13**, 034068 (2020).
[19] Friesen, M. & Coppersmith, S. N. Theory of valley-orbit coupling in a Si/SiGe quantum dot. *Phys. Rev. B* **81**, 115324 (2010).
[20] Paladino, E., Galperin, Y. M., Falci, G. & Altshuler, B. L. $1/f$ noise: Implications for solid-state quantum information. *Rev. Mod. Phys.* **86**, 361 (2014).
[21] Ramon, G. Dynamical decoupling of a singlet-triplet qubit afflicted by a charge fluctuator. *Phys. Rev. B* **86**, 125317 (2012).
[22] Szańkowski, P. Introduction to the theory of open quantum systems. *SciPost Phys. Lect. Notes* **68**, 1 (2023).
