# Supplementary material for: Impact of the local valley splitting on the coherence of conveyor-belt spin shuttling in 28Si/SiGe
Source: Nat Commun. 2026 Jun 19;17:5448. doi: 10.1038/s41467-026-74382-5 (PMC13282485; doi:10.1038/s41467-026-74382-5)
Supplement: Supplementary file 2 — Transparent Peer Review file [file 41467_2026_74382_MOESM2_ESM.pdf]

nature portfolio

Peer Review File

Impact of the local valley splitting on the coherence of conveyor-belt spin shuttling in $^{28}Si/SiGe$

Corresponding Author: Dr Lars Schreiber

This file contains all reviewer reports in order by version, followed by all author rebuttals in order by version.

Attachments originally included by the reviewers as part of their assessment can be found at the end of this file.

Version 0:

Reviewer comments:

Reviewer #1

(Remarks to the Author)
Referee report on "Reduction of the impact of the local valley splitting on the coherence of conveyor-belt spin shuttling in (28)Si/SiGe"

Key Results
The authors report on experimental mapping of the valley splitting landscape in a (28)Si/SiGe quantum well within in a spin-qubit shuttling device. The device is operated in conveyor-mode to probe the local valley splitting by a novel tomography approach that combines magnetic field tuning to track the resonance of the valley splitting and the Zeeman energy with electron shuttling and Pauli spin blockade readout. The authors report on significant local variations of the valley splitting (1.5 µeV to 200 µeV) induced by random alloy disorder in a 40nm x 400nm sample and find their results in line with the theoretically predicted Rice distribution. The long-term goal is a modular architecture of a quantum processor, where shuttling-devices play a key role for qubit transfer and manipulation. To this end, adaptation of the shuttling path to the disordered valley splitting landscape can help to mitigate spin-dephasing and decoherence by circumventing critical points in the channel (spin-valley hotspots, regions of low valley splitting).

The results are interesting as the disordered, locally varying valley splitting is considered as a major bottleneck for scalable spin-qubits in silicon, in particular for shuttling-based architectures. Demonstration of a practical mitigation strategy could meaningfully advance the viability of this technology platform towards practical quantum computing applications.

Validity
While the results presented in the manuscript are promising, I have some concerns about the strength of evidence and the robustness of the conclusions.

(1) Spatial sampling of only small domain
The valley-splitting map covers only a small domain (40 nm × 400 nm) compared to the quantum dot size (radius a_dot = 17.3 nm). Using the dot size as correlation length, I find that the probed domain corresponds to merely about 17 independent data points (ratio of areas). Therefore it is unclear to what extend the mapped sample is truly representative for typical device conditions and if the results can be extrapolated to larger distances etc.
In addition, I wonder if the present tomography method can be supplemented by an error analysis that provides a quantification of the uncertainty (-> error bars, confidence intervals etc.).
Moreover, it is not clear to me to what extend the tomography method still provides reliable information on much longer distances several µm deep in the channel. I expect that the uncertainty will increase for points far away from the SET, but I am missing a discussion of this in the manuscript.

(2) Device-to-device variability
The experiments were performed on a single device (or at least only one is reported). Given that the origin of valley splitting disorder, i.e., interface steps and alloy fluctuations, can vary strongly between wafers, devices, and fabrication runs, the reported findings may be a not representative. How likely is it that a different device will show similar valley landscapes, or

that the same device but different cooldown / wafer batch behaves similarly?

(3) No information on valley phase
The interpretation of experimental findings given by the authors in the main text mostly follows the logic of Landau-Zener physics, where (non-adiabatic) transitions are likely to occur at low energy gaps. The analysis of the valley dynamics in the supplementary note III, however, already shows that this is in fact not entirely true for the present system. Instead, the dynamics is dominated by the rate of change of the valley phase. While small valley splittings are naturally positively correlated with randomly occurring rapid changes of the valley phase, this proxy can also fail and lead to unfavorable shuttling paths.

(4) Scalability
The control strategy relies on trajectory selection to avoid critical spots in the landscape. In a realistic multi-qubit architecture, however, routing flexibility will be constrained by control wiring, positioning of readout zones, cross-talk etc. Moreover, I believe that tracking of the resonance line in the tomography raw data so far was done manually ("by eye"), which is impractical for larger data sets or even networks of devices (not to mention the measurement time required to map out the landscapes). The paper does not convincingly argue that trajectory planning remains feasible at scale. The authors made a proof-of-principle demonstration for a short channel, but scalability still appears vague to me.

Significance
If the mapping of valley splitting landscapes can be reliably scaled up to full shuttling lanes over several µm distances, and if trajectory-selection to circumvent critical spots remains viable under the constraints of a full device, then this work could significantly change how valley disorder is handled in silicon spin-qubit architectures. Rather than focusing on materials/fabrication improvements to enhance the valley splitting, the effort could be shifted towards control optimization to circumvent critical spots. This would potentially speed up development cycles and lead to a lowering of material constraints. Thus, I think the significance is high, but only if the approach is shown to be robust, reproducible, and scalable.

Data and methodology
The authors describe their experimental methodology and data post-processing routines in great detail. The tomography method is reasonably justified and was already described in more detail in a previous paper. Regarding the validity and statistical significance of results, I refer to my concerns raised above.

Suggested Improvements
(1) Larger-area mapping
The tomography approach could be applied to a larger area in order to test the statistical representativeness of the examined sample.

(2) Statistical analysis
Provide error bars, confidence intervals, and repeated-measurement statistics for valley splitting and coherence data. Robustness of results could be shown over multiple runs or cool-down cycles.

(3) Theoretical background in main text
I would recommend transferring some of the theoretical background given in the supplementary notes to the main text. In particular, I think that some of the mathematical notes given there on the shuttling model and spin-valley coupling are helpful to understand the measurement approach and therefore deserve to be moved to the main text.

(4) Multi-qubit shuttling
I assume that in large-scale quantum processors simultaneous shuttling of multiple electrons along the same channel might be necessary. I wonder if this is compatible with the proposed control strategy and would like to see a comment on this.

Clarity and context
The text is well-written and provides detailed information about the methodology. The introduction gives a solid background on why valley splitting disorder is a problem for silicon spin qubits. However, I think it is clearly aimed at a specialist audience, and I am not sure how accessible the text is to non-experts.

References
The manuscript includes a comprehensive literature review, citing relevant theoretical and experimental works on valley disorder and spin shuttling. To my understanding, this is fully adequate.

Recommendation
In summary, the present paper addresses an important and timely challenge in the field of silicon-based spin qubits. The experimental mapping of local valley splitting, correlated with spin-coherence in conveyor-mode shuttling, represents a significant progress. The proof-of-principle demonstration of coherence preservation over tens of microns (given careful trajectory selection) is a milestone contribution that is suitable for publication in "Nature communications". However, I

recommend some revisions addressing my concerns and questions raised above.

Reviewer #2

(Remarks to the Author)
See uploaded PDF.

Reviewer #3

(Remarks to the Author)
I co-reviewed this manuscript with one of the reviewers who provided the listed reports. This is part of the Nature Communications initiative to facilitate training in peer review and to provide appropriate recognition for Early Career Researchers who co-review manuscripts.

Reviewer #4

(Remarks to the Author)
In this article, by Volmer et al, the authors investigate the role of valley states and spin-valley coupling on the ability to shuttle electron spin qubits in Si/SiGe quantum dot devices, specifically when utilizing the conveyor-belt mode of spin transport. The ability to achieve mobile spin qubits, through coherent spin shutting, is a promising approach to create long distance entanglement as well as alleviate wire fan-out issues in densely packed spin qubit platforms.

This report carries on with the techniques and approaches shown in a previous Volmer et al. paper [npj Quantum Inf. 10, 61 (2024)] and advances that work by investigating and furthering the understanding of the specific error mechanisms associated with spin dephasing when shutting through low lying valley regions along a conveyor-mode transport path. Specifically, the demonstration of populating the excited valley state when moving through a very low valley state, even when de-tuned from the spin-valley hot-spot, is an important and notable observation. Additionally, the Supplementary Information (as well as main text) provides important analyses and methodologies which would provide a valuable resource to other interested in utilizing these approaches. I recommend the paper for publication, though I have listed several comments and questions below, which I feel should be addressed:

(1) I have a couple suggestions/comments for figure 1. If there is room, pictorially showing where the shuttling (QuBus) device gates (S1,S2,S3,S4) are along d. My thought here would be to place carton gates, high-lighting the gate widths and small gapes from inter-gate dielectrics, above 1(d). It would be good to see if any of the peaks/troughs of the valley splitting coincide with locations where the QD is residing under, or shared between, two S gates. Would the authors expect this to be the case? Also, is the (slight) horizontal feature at ~ 0.95T in 1(d) the valley hot-spot associated with the static QD? It would be worth identifying//commenting on this as one would expect a valley feature from that interaction as well.

(2) Is it possible to generate a plot of $\Delta g(d,y)$,similar to 2(d)? Does this information fall out of the plots like 1(d) (with some math to deconvolve the integrated vs local g-factor difference)? This would be of great interest to the community, even if only in the supplementary information. Along these lines, are there any correlations between Evs and Δg (or in the locations of rapid change in either value)?

(3) I feel there should be some comment on or comparison to the expected (theory) or observed (experimental) correlation lengths from literature. How does this compare to measured or theorized alloy disorder correlation lengths.

(4) I do understand the assignment of the rising background in figure 4b to a slow singlet triplet oscillation. This oscillation would be limited by the same T2* dynamics as the faster oscillation and would decay on a similar 2us timescale. Is there another mechanism that may be plausible? Is this a measurement artifact?

(5) Over how much time were the dephasing measurements averaged (T2* and T in Table I and in the discussion of Figure 4)? i.e. do they represent ergodic numbers. It would be good to explicitly state this somewhere.

(6) How does the 92% shuttle fidelity compare to results in the literature? There have been a lot of exciting results recently.

(7) In the discussion of figures 3(b,c,d), there is a comment in the bottom left paragraph of page 5 that higher velocity shuttling better preserved coherence (since the oscillation amplitude is unchanged). This is not clear to me from the data, how is this quantified?

(8) A reference (or references) for the realistic spin-valley-coupling of near 300neV would be helpful.

(9) Spin-valley coupling has been observed to dephase S/T qubits when operated near a valley hot-spot. How does this effect compare to that?

(10) The article indicates the dot location is found through methods used in Ref43; triangulation and Electrostatic simulation of full device in COMSOL. How does the dot size calculated from the auto correlation function compare to these simulations? A naïve picture might have the QD move like an inch-worm along the path expanding when the QD is residing under, or shared between, two S gates (since there is a dielectric gap between the gates) and contracting when it is fully located under a single QD. Does modeling show a smooth movement of the QD wavefunction?

Version 1:

Reviewer comments:

Reviewer #1

(Remarks to the Author)
The authors have answered my questions satisfactorily. I am happy with the revision and recommend the manuscript for publication.

Reviewer #2

(Remarks to the Author)
I thank the authors for their rebuttal and revised manuscript. I appreciate the authors detailed responses to the concerns raised in my original review, in particular the clarification of the state initialization, the improved explanation of the valley-splitting extraction procedure, the change of title, and the correction of the spatial-anisotropy issue in Fig. 2. These changes have significantly improved the clarity and accuracy of the manuscript. The additional supplementary material, clarifications in the main text, and improvements to figures and terms all contribute to a more complete and clear presentation of the impressive work within this manuscript. I thank the authors for sharing the pre-print manuscript for my reference, and look forward to seeing it on the arXiv shortly.

In my view, the revised manuscript now meets the standards for publication in Nature Communications. I therefore recommend publication in its current form.

Reviewer #3

(Remarks to the Author)
I co-reviewed this manuscript with one of the reviewers who provided the listed reports. This is part of the Nature Communications initiative to facilitate training in peer review and to provide appropriate recognition for Early Career Researchers who co-review manuscripts.

Reviewer #4

(Remarks to the Author)
Dear Nature Communication Editors,

Upon reading the point-by-point response letter and the revised manuscript by Volmer et al, I feel both my comments and points raised, and that of the other reviewers, were adequately addressed. The added details and discussions throughout the manuscript, and supplement, have improved the manuscript. Additionally, I'd like to thank the authors for the thorough responses and additional details which has clarified the questions I raised. I recommend this work for publication in Nature Communications.

Thank you,
Ryan Jock

Open Access This Peer Review File is licensed under a Creative Commons Attribution 4.0 International License, which permits use, sharing, adaptation, distribution and reproduction in any medium or format, as long as you give appropriate credit to the original author(s) and the source, provide a link to the Creative Commons license, and indicate if changes were made.
In cases where reviewers are anonymous, credit should be given to 'Anonymous Referee' and the source.
The images or other third party material in this Peer Review File are included in the article's Creative Commons license, unless indicated otherwise in a credit line to the material. If material is not included in the article's Creative Commons license and your intended use is not permitted by statutory regulation or exceeds the permitted use, you will need to obtain permission directly from the copyright holder.
To view a copy of this license, visit https://creativecommons.org/licenses/by/4.0/

We would like to thank the Reviewers for careful reading of the manuscript and giving constructive feedback that allowed us to improve the manuscript significantly. The comments of all the Reviewers show deep understanding of the work, as they had pointed us towards opportunity of clarification and improvement of the manuscript as well as the presentation, which we have acted on majorly. We truly believe that this has significantly improved the manuscript, and thank the Reviewers for their time and meticulousness. Below is a point-by-point discussion of all points raised by the Reviewers (black text) with our response (blue text) and changes made to the manuscript (red text). We have also have marked the changes to the manuscript in the attached markup version of the article and the supplementary information. Each change is referenced by a quote in the answer, and includes a referral line pointing towards the change.

First, let us summarize the major changes following suggestions of the Reviewers:

1. **Change of Title:** The title of the manuscript was changed from "Reduction of the impact of the local valley splitting on the coherence of conveyor-belt spin shuttling in ^{28}Si/SiGe" to "Impact of the local valley splitting on the coherence of conveyor-belt spin shuttling in ^{28}Si/SiGe"

2. **Change of Figure 1:** For better clarity, Figure 1 was changed to a two column spanning figure to improve the readability of the pulse scheme

3. **Change of Figure 2:** The authors noticed a mismatch between the raw data in Fig. 2a and the composed map in Fig. 2d originating from non-regularity of the underlying spline. This was discovered after a comment from Reviewer 2 regarding the seeming anisotropy of the map. We have corrected the 3D map.

4. **We have added supplemental material:**

   - Addition of Supplementary Note I: "Valley splitting vs. initialized valley state". By simulating the full 16×16 Hamiltonian of the system as a function of magnetic field, we demonstrate why the positions of the anticrossings is independent of the initialized valley state. Hence, we can detect the valley splitting, even if the initialized valley state is uncontrolled.

   - Addition of Supplementary Note V: "Unedited version of Figure 3". A version of Figure 3 without lines and arrows to show all data.

In addition, we have attached to this rebuttal letter the draft of Ref. 55 titled "Singlet-triplet oscillations in multivalley Si double quantum dots" requested by reviewer 2, which we are going to upload on a preprint server soon. These major changes are accompanied by other improvements triggered by comments of the Reviewers (see below).

I. REVIEWER 1

Recommendation: In summary, the present paper addresses an important and timely challenge in the field of silicon-based spin qubits. The experimental mapping of local valley splitting, correlated with spin-coherence in conveyor-mode shuttling, represents a significant progress. The proof-of-principle demonstration of coherence preservation over tens of microns (given careful trajectory selection) is a milestone contribution that is suitable for publication in "Nature communications". However, I recommend some revisions addressing my concerns and questions raised above.

We are grateful that the Reviewer acknowledges our demonstration as a milestone contribution and we thank the Reviewer for their positive recommendation to publish our article in Nature Communications. The comments of the Reviewer are very valuable and addressed below. We have made major modification to the manuscript based on the excellent comments of all four Reviewers. We hope that the Reviewer agrees that our manuscript has become substantially improved.

Key Results: The authors report on experimental mapping of the valley splitting landscape in a (28)Si/SiGe quantum well within in a spin-qubit shuttling device. The device is operated in conveyor-mode to probe the local valley splitting by a novel tomography approach that combines magnetic field tuning to track the resonance of the valley splitting and the Zeeman energy with electron shuttling and Pauli spin blockade readout. The authors report on significant local variations of the valley splitting (1.5 μeV to 200 μeV) induced by random alloy disorder in a 40 nm $\times$ 400 nm sample and find their results in line with the theoretically predicted Rice distribution. The long-term goal is a modular architecture of a quantum processor, where shuttling-devices play a key role for qubit transfer and manipulation. To this end, adaptation of the shuttling path to the disordered valley splitting landscape can help to mitigate spin-dephasing and decoherence by circumventing critical points in the channel (spin-valley hotspots, regions of low valley splitting).

We thank the Reviewer for the summary of the findings. We would like to point out that the applied method for mapping the valley splitting is already published in Ref. 43. There we applied it on a non-isotopically purified Si/SiGe shuttle device and successfully benchmarked it against the established method of magneto-spectroscopy. Indeed, we report that we can reproduce the method of mapping the valley splitting here. For the reproduction we use an isotopically purified Si/SiGe heterostructure and could increase considerably the size of the mapped area. In the second half of the manuscript, we show that the change in visibility of the S-T_0 oscillations indicating spin decoherence is directly related to local valley splitting features: (i) spin-valley hot-spots and (ii) valley splitting minima. Though theoretically predicted, this is the first experimental evidence of the spin decoherence mechanisms. Using the knowledge mapped valley splitting we can pick a shuttle path and enhance the spin shuttle fidelity. This is in line with our long-term vision mentioned by the reviews and detailed in Nature Communications (Ref. 28).

The results are interesting as the disordered, locally varying valley splitting is considered as a major bottleneck for scalable spin-qubits in silicon, in particular for shuttling-based architectures. Demonstration of a practical mitigation strategy could meaningfully advance the viability of this technology platform towards practical quantum computing applications.

We thank the Reviewer for their assessment that our results are interesting and paving the way towards practical quantum computing with shuttling-based architectures. The points of the Reviewer are very well structured and reveal the deep consideration of the Reviewer concerning scalable architectures.

Validity:

While the results presented in the manuscript are promising, I have some concerns about the strength of evidence and the robustness of the conclusions.

(1) Spatial sampling of only small domain The valley-splitting map covers only a small domain (40 nm × 400 nm) compared to the quantum dot size (radius a_{dot} = 17.3 nm). Using the dot size as correlation length, I find that the probed domain corresponds to merely about 17 independent data points (ratio of areas). Therefore it is unclear to what extend the mapped sample is truly representative for typical device conditions and if the results can be extrapolated to larger distances etc. In addition, I wonder if the present tomography method can be supplemented by an error analysis that provides a quantification of the uncertainty (→ error bars, confidence intervals etc.). Moreover, it is not clear to me to what extend the

tomography method still provides reliable information on much longer distances several µm deep in the channel. I expect that the uncertainty will increase for points far away from the SET, but I am missing a discussion of this in the manuscript.

The Reviewer addresses a couple of important points here. Indeed, we can measure valley splitting at thousands of locations in the shuttled area and fit the Rice distribution for an unprecedented large number of valley splitting samples. We also calculate the correlation length of the valley splitting map to be 18.1 nm (matching the QD radius; corrected value with 17.3 nm being the old value). The area of the map is 36 nm by 400 nm, if we refer to the center of the QD. For an area calculation - as done by the Reviewer – the QD radius has to be added to all the area circumference: 72 nm by 436 nm. Divided by area of a QD equal to 130 nm^2 (as apparently done by the referee), we arrive at 30.5 independent samples, not 17. This assumes a distance of two times the correlation length (2*18.1 nm) between samples. Commonly, samples at a distance of *one* correlation length is considered as approximately independent. Therefore, we have 122 independent samples, but even 30 is already a large sample size compared to other studies.

We added to the main text (lines 227-229):

> [...] valley splitting values. This implies 122 samples of measured E_{VS} spaced by a distance of a QD radius being the correlation length. As the mapping method might [...]

Concerning the error analysis of $E_{\mathrm{VS}}(d)$: In supplementary Figs. 3-9, we marked along d a B-field range by two black ines. Within this area the spin-valley resonance is visible, thus is represents a very conservative estimate of the confidence interval. Since we mark the spin-valley resonance by eye, it is hard to give a precise error analysis. In the supplements of Ref. 43, we provide a more detailed discussion of the leateral with of this resonance peak.

The Reviewer is right that there are limitations to be considered if one wants to further increase the area of the map. We observe a slight drop in visibility of the signature of valley resonance, if the electron passes several spin-valley hotspots along its trajectory. This can be mitigated by shuttling faster, and ultimately mitigated by optimizing the shuttle trajectory. We suggest the following algorithm to enlarge the area: First, an area of 40 nm by 500 nm is mapped and the best shuttle trajectory selected. Then the next and deeper into the shuttle reaching 40 nm by 500 nm area is mapped. To reach the second map the first is crossed by

an optimized trajectory. Then a third area is added and the previous two are crossed by the optimal trajectory and so on. In Ref. 37, the authors extrapolated their shuttle fidelity to a coherent shuttle distance of a millimeter in similar Si/SiGe heterostructures, although such a distance is far beyond what is required for quantum computing architecture. Therefore, there Is no fundamental limit to our method. In our study, the investigated area was limited by the gates of the shuttle device.

We added a notion to the method section (lines 701-713):

> [...] we omit it here.
>
> We observe a slight drop in visibility of the signature of valley resonance, if the electron passes several spin-valley hotspots along its trajectory. Passing serveral spin-valley hotspots can be mitigated by shuttling faster and ultimately mitigated by optimizing the shuttle trajectory. We suggest the following algorithm to enlarge the area: First, an area of 40 nm by 500 nm is mapped and the best shuttle trajectory selected. This trajectory is used to reach the next area (of the same size as the first one) in order to perform mapping there. Then a third area is added and the previous two are crossed by the optimal trajectory, and so on.
>
> In summary, a valley splitting [...]

(2) Device-to-device variability The experiments were performed on a single device (or at least only one is reported). Given that the origin of valley splitting disorder, i.e., interface steps and alloy fluctuations, can vary strongly between wafers, devices, and fabrication runs, the reported findings may be a not representative. How likely is it that a different device will show similar valley landscapes, or that the same device but different cooldown / wafer batch behaves similarly?

We agree with the Reviewer that it is important to check whether the found valley splitting distributions are representative. We confirm that the valley splitting depends on the Si/SiGe heterostructure and wafers. In Ref. 43, we used the same method to map a commercial Si/SiGe heterostructure with a thicker QW. The observed valley splitting distribution is narrower and offset from zero compared to the one of the study here. To check the validity of the mapping methodology employing qubit shuttling ,in Ref. 43 we compared the statistics of valley splittings obtained from mapping results to another shuttle

device fabricated on the same heterostructure in which valley splittings at multiple dot positions were measured by magnetospectroscopy. The results matched within statistical uncertainty.

We expect that changes in the fabrication of a device that alter charged defect density at the semiconductor/oxide interface, electric fields in growth direction in the quantum well, heat impact and Ge diffusion in the SiGe heterostructure have an impact on the valley splitting distribution. With our mapping method these differences in fabrication can be mapped and progress in material benchmarked.

In this manuscript, we do not focus on the methodology of valley splitting mapping. We focus on the relation of the valley splitting map to decoherence during shuttling. Here the measurement of the map and the measurement of the shuttle decoherence are two consecutive experiments conducted with a time gap of some weeks. Importantly, we observe that the decoherence during shuttling depends clearly on shuttle distance and shuttle trajectory. We observe that the shuttling decoherence is correlated with the valley splitting map (spin-valley hot-spots, low valley splitting regions). In this sense our valley spitting map is representative for the shuttle device and can be used to optimize decoherence by selecting the trajectory. We believe that the excellent reproducibility of a valley splitting map is based on the fact that the map is related to the position of the Ge atoms. Therefore, we do not see any drifts of the valley splitting after months. Of course, for another shuttle device the valley splitting landscape has to be mapped first and only then the trajectory can be optimized. For this new landscape the described phenomena at low valley splitting and spin-valley hotspots will occur according to the theory which we support in our experiment.

We added (lines 576-578):

> [...] these strategies and we exploit that the E_{VS} is highly reproducible for a device within a thermal cycle. We observe [...]

(3) No information on valley phase The interpretation of experimental findings given by the authors in the main text mostly follows the logic of Landau-Zener physics, where (non-adiabatic) transitions are likely to occur at low energy gaps. The analysis of the valley dynamics in the supplementary note III, however, already shows that this is in fact not entirely true for the present system. Instead, the dynamics is dominated by the rate of change of the valley phase. While small valley splittings are naturally positively correlated

with randomly occurring rapid changes of the valley phase, this proxy can also fail and lead to unfavorable shuttling paths.

The Reviewer has an important point here. Theory predicts that valley excitations are governed by phenomena not always described by Landau Zener physics. Actually, valley excitations depend on the ratio of the valley phase gradient and the valley splitting energy. We explain this in our supplementary information. Hence, in principle a valley excitation could be observed at a rather high valley splitting with the phase has an extremely sharp gradient according to theory. We have not observed this phenomenon here. The reason might be simply that such valley excitations is by far less probable to occur. We are currently working on a new mapping methodology for valley-splitting and for the *valley phase* such that occurrences of these rare events can be covered in the future. A preprint of this work can be found at https://arxiv.org/abs/2603.01844. We have added this to the discussion (lines 566-568):

> [...] spintronics [58]. Additionally mapping the g-factor, we can evaluate the complex intervalley coupling, offering a complete analysis of the valley physics [59]. Our work suggests [...]

(4) Scalability The control strategy relies on trajectory selection to avoid critical spots in the landscape. In a realistic multi-qubit architecture, however, routing flexibility will be constrained by control wiring, positioning of readout zones, cross-talk etc. Moreover, I believe that tracking of the resonance line in the tomography raw data so far was done manually ("by eye"), which is impractical for larger data sets or even networks of devices (not to mention the measurement time required to map out the landscapes). The paper does not convincingly argue that trajectory planning remains feasible at scale. The authors made a proof-of-principle demonstration for a short channel, but scalability still appears vague to me.

The Reviewer argues exactly in our spirit (see also Ref. 28). We focus very much on the simplification of control wiring and therefore we are using shuttling in conveyor-belt mode with only four signals required because most gates are electrically connected. For selecting a shuttle trajectory, the voltage on the screening pulse has to be pulsed. This requires an additional wire and the pulse needs to be calibrated. In comparison to Ref. 28, we need to add 4 lines (for 4 shuttle sections) per unit cell. The overhead is manageable.

The Reviewer is right that we track the valley splitting curve “by eye”. We expect that advanced fitting or deep neural network can track the resonances at least as good. It is important that the mapping of the valley splitting is not time-critical, since it does not require recalibrations once the ´quantum chip is cold. In comparison, many other chip parameters e.g. SET operation point or qubit manipulations zones (in dense QD arrays) need frequent recalibrations. In a large device with shuttle networks, mapping methods can be applied in parallel. The required time for mapping is estimated in method section "Efficiency of valley splitting mapping"

We add a notion about “by eye” method to the caption of Fig. 1 in line F1.10

Significance: If the mapping of valley splitting landscapes can be reliably scaled up to full shuttling lanes over several µm distances, and if trajectory-selection to circumvent critical spots remains viable under the constraints of a full device, then this work could significantly change how valley disorder is handled in silicon spin-qubit architectures. Rather than focusing on materials/fabrication improvements to enhance the valley splitting, the effort could be shifted towards control optimization to circumvent critical spots. This would potentially speed up development cycles and lead to a lowering of material constraints. Thus, I think the significance is high, but only if the approach is shown to be robust, reproducible, and scalable.

We thank the Reviewer for pointint out the high significance of our work. Indeed faster progress is possible with our method. For large chips with millions of qubits an optimized material with globally larger valley splitting is very welcome. We believe that our introduced methodology to map the valley splitting helps to benchmark the material progress.

Data and methodology: The authors describe their experimental methodology and data post-processing routines in great detail. The tomography method is reasonably justified and was already described in more detail in a previous paper. Regarding the validity and statistical significance of results, I refer to my concerns raised above.

We hope to convincingly address all the concerns on validity and statistical significance above. We hope that the Reviewer acknowledges that we relate the valley splitting map to decoherence phenomena during shuttling.

Suggested Improvements: (1) Larger-area mapping The tomography approach could be applied to a larger area in order to test the statistical representativeness of the examined sample.

Without doubt, we plan to gradually increase the size of the mapped area. Compared to Ref. 43, we could already make some considerable progress. There is no larger valley splitting map reported in the literature than what we show here.

Suggested Improvements: (2) Statistical analysis Provide error bars, confidence intervals, and repeated-measurement statistics for valley splitting and coherence data. Robustness of results could be shown over multiple runs or cool-down cycles.

It is a very valid point of the Reviewer to check, whether the valley splitting map and coherence data is reproducible. In fact, we recorded the valley splitting map in several overlapping patches, some with higher resolution to track the valley splitting within smaller confidence interval (see supplements). The match among patches is perfect, confirming the high reproducibility. We also repeated the shuttle experiments a couple of times and found the results very reproducible. We provide confidence interval for the valley splitting (see above). We see no necessity for measuring the device for multiple cool-down cycles as quantum chips usually stays cold. In the future, we may study whether valley splitting maps are robust against thermal cycles, but this is beyond the scope of this study where we compare a valley splitting map with decoherence during shuttling.

Suggested Improvements: (3) Theoretical background in main text I would recommend transferring some of the theoretical background given in the supplementary notes to the main text. In particular, I think that some of the mathematical notes given there on the shuttling model and spin-valley coupling are helpful to understand the measurement approach and therefore deserve to be moved to the main text.

The Reviewer is right that our extended theory in the supplement is very helpful to understand the measurement. The theory is an excellent summary of previous publication and extends to the observed decoherence phenomena. We decided to focus on the measurements and provide the connection to theory by giving a limited theory background in the main text. In the main text, we give an overview of the key theory sections in the last paragraph of the section "Spin decoherence during shuttling".

Suggested Improvements: (4) Multi-qubit shuttling I assume that in large-scale quantum processors simultaneous shuttling of multiple electrons along the same channel might be necessary. I wonder if this is compatible with the proposed control strategy and would like to see a comment on this.

The Reviewer makes an excellent point here. Indeed, the pulsing of a long screening gate

modifies the position of all QDs of a shuttle and therefore the trajectory of e.g. two qubits in one shuttle cannot be independently controlled. There are two solutions to this problem: (i) Only one qubit per shuttle. this is the approach of the spin-bus architecture Ref. 28. (ii) Split in the screening gate sections e.g. each 10 shuttle unit cells. Then qubits can follow at a distance of 3 µm. Then every shuttle section requires individual calibration of the trajectory with an individualized voltage pulse. In the end, it is a balance between qubit density vs signal wiring density.

Clarity and context: The text is well-written and provides detailed information about the methodology. The introduction gives a solid background on why valley splitting disorder is a problem for silicon spin qubits. However, I think it is clearly aimed at a specialist audience, and I am not sure how accessible the text is to non-experts.

The Reviewer is right that we do aim at a specialist audience with our introduction. We believe that is the right approach for an article in Nature Communications, as this journal addresses a specialized audience according to its scope. An article for the Nature journal would have a broader introduction.

References:

The manuscript includes a comprehensive literature review, citing relevant theoretical and experimental works on valley disorder and spin shuttling. To my understanding, this is fully adequate.

We agree with the opinion of the Reviewer. We updated some references that appeared on preprint servers or in peer-reviewed journals: [5],[32],[44],[46],[53],[61],[62].

II. REVIEWER 2

The article presents a detailed experimental study of the interplay between shuttling parameters (rate, distance, path, time) and the fidelity of a spin state over the shuttling operation. The work draws on the experimental method previously developed by this team (Ref 43) to corelate features in the shuttling fidelity directly with the local structure of the valley splitting and Zeeman energies. Shuttling protocols which preserve spin coherence are of significant importance in the semiconductor spin-qubit community. Further, this work presents a novel experimental characterization of a key material parameter, namely valley splitting, which will be of interest to the wider silicon quantum technology community. In

particular, the precise knowledge of valley hot-spots can be useful in order to avoid them to preserve coherence or as a go-to operation point where fast relaxation rates are desired for initialisation into a spin ground state. As such, I support publication of this manuscript in Nature Communications provided the authors are able to satisfactorily address the following concerns. Below are several major comments which the authors must address before I can support publication, and some minor comments to improve readability.

We thank the Reviewer for their positive assessment of our work and for supporting its publication in Nature Communications. We are particularly grateful for the Reviewer's recognition of the study's relevance to both the semiconductor spin-qubit community and the wider silicon quantum technology field. We also appreciate the insightful comments regarding the dual utility of mapping valley hot-spots—both for preserving coherence and for enabling fast relaxation during initialization. We have addressed the major and minor concerns in detail below and believe these revisions have significantly strengthened the manuscript.

1. The authors should provide a clearer explanation of initialization of the (3,1) state from the (4,0) configuration. At present the authors claim that after initializing (3,1) the "system can be described by a spin-singlet (S) state with potentially mixed valley-state, Si, where i is an element of $\{|+-\rangle, |+-\rangle, |++\rangle, |-,+\rangle\}$". The authors support this statement with two references, Refs 55 and 43. The initialization of the (3,1) spin-orbit-valley state is fundamental to the subsequent analysis, which involves interpreting resonances of the initialized state with the polarized triplets (based on ref 43). If I cannot comprehend what (3,1) state is initialized it is difficult to comprehend the meaning of the subsequent analysis.

We thank the Reviewer for pointing our attention to this blank space in our argumentation. The explanation for the state initialization is a key ingredient to understanding the physics of singlet-triplet oscillations in the vicinity of the hotspot. For one specific valley initialization, this is explained in Ref. 43. Moreover the Reviewer addresses the possibility of a valley mixture being initialized, which is also mentioned in the main text and has indications in the data. Indeed, here we need to address the full (two-electron) four-dimensional valley space as well as the full four-dimensional spin space. Hence, the state space expands to a total of 16 dimensions. This added complexity requires careful analysis of every possible hotspot (eight in total), which is covered in Ref. 55. We attach a draft of this paper to this letter, and we will upload it to a preprint server soon. In addition, we have added a simula-

tion of the eigenenergies of the full 16 dimensional state space in the updated supplementary information (see the new Supplementary Note I). Indeed, the analysis yields two anticrossings for each valley state that are positioned right at the valley splitting equivalent Zeeman energies independent of the valley initialization. The method of $E_{VS}(d)$ mapping is thus insensitive to the valley occupation pattern of the initialized $(3, 1)$ singlet. This crucial fact is now mentioned in the main text of the paper (see below), while the added Supplementary section makes the paper self-contained. Many more details on the initialization of all valley occupations of $(3, 1)$ singlets, and details of dynamics that depend on these occupations are contained in Ref. 55.

Firstly, the inclusion of the word "potentially" is vague. Are the authors implying they are creating a equal mix of the 8 singlet-triplet-valley states, or simply that this could happen, or do they expect only one valley-state (ie $|+-\rangle$) is initialized but they don't know which one. Secondly, the details of the initialization are primarily supported by Ref 55, which is identified as 'In preparation' in the reference list. This means I have no way to independently verify the scientific content of statement (perhaps the authors should wait until Ref 55 is peer reviewed, or at the very least on a preprint server). Finally, the authors also cite ref 43 for their state initialization. However, this work claims that one specific valley-orbit state is initialized, rather than a potential mix of all 4. I would appreciate a plot of the eigenenergy's of the relevant energies to support the understanding of the experiment.

The Reviewer addresses the lack of availability of ref. 55, a problem that already has been raised by the prior point. The current version of the preprint is appended. We apologize for this inconvenience. Moreover, we added a careful simulation of the eigenenergies to the supplementary material of this work (new Supplementary Note I). We agree that this visual representation of the physics aids the understanding greatly. From this, one can see that independent of the initialized valley configuration the Zeeman energy of the anticrossing relates to the valley splitting energy with permille uncertainty. Furthermore, the Reviewer addresses that the text is unclear about the valley initialization. In the new version of the manuscript we have removed the word "potentially", and we simply state that one of S_i singlets is initialized. The manuscript reads now (lines 93-95):

> Now, the system can be described by a spin-singlet (S) state ~~with a potentially mixed valley-state~~, S_i, where $i \in \{|+-\rangle, |--\rangle, |++\rangle, |-+\rangle\}$ [55] ~~represents the~~

~~valley state of~~ labels the singly-occupied valley states in the left and the right QD.

The independence of "mapping by finding spin-valley resonances" procedure on the i index is now explicitly mentioned in the text (lines 134-135):

> [...] of a QD. The matching is independent of the initialized valley state (see Supplementary Note I) and we do not [...]

Later, when signatures of initialization of statistical mixture of two S_i states are mentioned in the text, they are always accompanied by citation of Ref. 55, in which this issue is discussed at length.

Fundamentally I trust that the method the authors use to identify valley splitting is independent of the valley-orbit state that is initialized, as I naively expect that there is a symmetry in the behavior of the polarized triplets with respect to the 4 valley-orbit configurations. However, I would expect the authors of the paper to supply necessary figures/equations/assumptions to support this within this manuscript or supplement.

The Reviewer proves an immediate understanding of the matter at hand by suggesting symmetry between the valley states and the interaction of their unpolarized states with the polarized triplets of the other valley states. For a full understanding, one needs to do the involved analysis case-by-case to find that the intuitively suggested symmetry holds. This is demonstrated in the added Supplementary Note I. Moreover, the appended manuscript in preparation elaborates on all these issues, giving detailed support to the statements that the identification of valley splittings in both dots does require the knowledge of which of $(3, 1)$ singlet states is initialized.

2. Please provide an explicit statement of the fitting method used to quantify the valley-splitting from figure 1d in the main text. I found it confusing to determine exactly how the valley-splitting is quantified using just this manuscript alone. The authors should include a clear direct statement of how the dashed line in figure 1e) is determined in the caption or main text. Reading the supplement of this manuscript I was left more confused regarding the dashed line in figure 1e. In some sections of the supplement (e.g. Supp. Note II) the authors refer to "manual guides to the eye for the found valley splitting curve", however there is no explanation on how the curve is fitted to the experimental data. Then in the

caption they are referred to as "offset copies of spline fits" (such as in supplement figures 2-8). To understand how the dashed line in Figure 1e was assigned I had to look in the supplementary material of Ref 43, where I now understand the dashed line is determined by eye/hand based on discontinuities in the normalized singlet return probability. I have no issue with by eye/hand assignment of the valley-splitting, however it should be clearly stated in the caption.

The Reviewer requests an explicit statement on the readout method. We are happy to clarify this by adding

> (e) Same as d but $E_{\mathrm{VS}}(d)$ is highlighted by the black dashed spline that was carefully put by hand.

to the caption of Fig. 1 (line F. 1.10).

3. In figure 2d the features in the valley profile appear much sharper along the y-axis than along the x-axis. In particular, in the region of x from 100 nm to 220 nm there are several undulations in valley splitting with a spatial width of about 50 nm in x direction. However, the same undulations rise and fall over less than 10 nm in the y direction. Can the authors comment on this feature? Is this perhaps an underestimation of the conversion of voltage to y-displacement, are there physical reasons why the x and y valley profile would be different (crystallographic?), or is this just the case for this particular region?

We want to emphasize that this comment pointed us towards analyzing the data again. We found that from Fig. 2a to 2d, there was a translation mistake due to the irregular nature of splines. We immediately fixed this issue that propagates to Fig. 2b and 2c, resulting in minor changes in results presented there: a change in distribution parameters, as well as the correlation length. The relevant part of the updated text is as follows (lines 197-199)

> [....] that has a small deterministic component ~~$\gamma = 0.0(6)$ μeV and a large spread $\sigma = 61.5(8)$ μeV~~ $\gamma = 0.1(7)\,\mu\mathrm{eV}$ and a large spread $\sigma = 64.3(8)\,\mu\mathrm{eV}$ suggesting larger impact of alloy disorder [...]

We thank the Reviewer for this suggestion that improved the analysis significantly. Now the variations should be more convincingly isotropic.

The discussion of the features in figures 3b-d) seems quite qualitative and the language of 'spin coherence' leaves some open questions. In Figure 3b-d) the authors show plots

of singlet return probability as a function of shuttle distance and total shuttle time. The understanding is that for a fixed distance 'd' a vertical line cut (varying ts) represents shuttling to the same location at different speeds. To me the 'coherence' would represent the time decay of the state as a function of time. It is not clear if the authors are considering this vertical line cut (ts) to represent coherence time (ie the Ps decay in the oscillations with respect to the total shuttling time). My understanding is this is not necessarily what the authors are directly interested in, instead they are talking about the maximum probability of returning the initial state, which to me would be more like the 'shuttling fidelity'. Within this analysis the authors identify distances that produce a "sudden reduction in the visibility of the oscillations." At this point the language of 'coherence' becomes confusing as a reduction in coherence would suggest a quicker decay envelope. Instead, I think the authors are talking about the overall suppression in the amplitude of the oscillations which occurs. The total overall suppression of the oscillations beyond d=220nm, however this may not necessarily be a shortening of the coherence envelope. Instead, an overall suppression in oscillation amplitude could represent spreading the initial state across more possible quantum states, resulting in more complex dynamics (which does seem to happen based on the FFT's in Fig 3e-g). This comment could be addressed by more clearly defining the terms.

The Reviewer addresses issues with terminology regarding shuttling coherence as well as shuttling fidelity regarding Fig. 3. Indeed, the main result from Fig. 3c,d,f and g is the observation of an influence of the passage through spin-valley hotspot on the measured singlet return probability signal. The key result here is that we observe an increased reduction of singlet-triplet oscillation amplitude with slower shuttle velocity (indicated by the arrows), once we shuttle past the hotspot. This is due to the fact that the slower we shuttle to a certain position, the more time is spent near the hotspot, and the spin-valley dynamics during this passage is more adiabatic. To us, using the terminology of 'shuttle fidelity' implies that we benchmarked it in more detail, hence running into the risk of overpromising on our results. However, we agree that a clarification of terminology here improves the clarity of the manuscript. Therefore, we implemented the following changes (lines 241-280):

> We start with $B = 1.7\,\mathrm{T}$ (Fig. 3b), since the Zeeman energy of the moving QD satisfies $E_\mathrm{Z} > \max(E_\mathrm{VS}(d))$ (yellow line in Fig. 3a) and we expect no ~~spin decoherence~~ drop in ST-oscillation amplitude at spin-valley resonances defined

by $E_Z = E_{VS}$ [34]. For $P_S(d, \tau_S)$, we use various shuttle velocities, and some $P_S(d, \tau_S)$ are inaccessible (white areas) due to the maximum shuttle velocity of $5.6\,\mathrm{m\,s^{-1}}$. Similar to Ref. [36], we observe variations in the singlet-triplet oscillation frequency stemming from $\Delta g(d)$. More importantly, the amplitude of the singlet-triplet oscillations ~~and thus the spin coherence~~ is suddenly reduced at $d \approx 240\,\mathrm{nm}$ (dotted vertical lines in Fig. 3a,b). This is remarkable and not unexpected, as this sudden loss of ~~spin coherence~~ ST-oscillation amplitude coincides with the first spot at which $E_{VS} < 5\,\mu\mathrm{eV}$.

The $P_S(d, \tau_S)$ measured at $B = 0.3\,\mathrm{T}$ (Fig. 3c with maximal velocity $2.8\,\mathrm{m\,s^{-1}}$) exhibits a smaller frequency of ST-oscillations, as expected, but also reveals very different ~~spin-decoherence~~ signal-decay characteristics. Here, we observe a sudden reduction of the visibility of the oscillations and thus increase of ~~spin-decoherence~~ signal decay after passage beyond $d = 50\,\mathrm{nm}$ (dashed line in Fig. 3c). Remarkably, this coincides with the first spin-valley resonance passed by shuttling (dashed vertical red lines in Fig. 3a). Repeating the measurement with a reduced $B = 0.1\,\mathrm{T}$ (Fig. ??d), the sudden loss of ~~spin coherence~~ signal appears at larger $d = 240\,\mathrm{nm}$ (black solid line), which coincides with the first spin-valley resonance at the corresponding reduced E_Z (solid violet line in Fig. 3d). Furthermore, when following $P_S(d, \tau_S)$ oscillations for constant shuttle velocities (see arrows in Fig. 3c as a guide), we observe that shuttling across spin-valley resonances with higher velocity tends to preserve the ~~spin coherence~~ ST-oscillations compared to lower shuttle velocities for both magnetic fields. Specifically, at the largest $v_S = 2.8\,\mathrm{m/s}$, the oscillation amplitude is unchanged and the ~~spin coherence~~ signal strength is not affected by the resonance. The opposite trend is observed if the onset of ~~spin decoherence~~ signal decay coincides with the crossing of valley splitting minimum in Fig. 3b.

We thank the Reviewer for bringing this opportunity for clarification to our attention.

Minor comment 1: The title of the manuscript does not accurately reflect the nature of the work. As the authors state in their introduction, they “explore the interplay of spin coherence and conveyor-mode shuttling” in the context of regions of low spin-valley splitting and resonances between valley splitting and Zeeman energy. I agree that the

insights provided by this manuscript could in the future be used to further reduce the impact of valley splitting on shuttling operations. Additionally, I agree that the authors show that some parameters do show improved/reduced shuttling coherence. However, the current title implies they have demonstrated a systematic reduction in the impact of valley-splitting, which I don't find accurate. I would suggest a more appropriate title to be along the lines of 'A study of the interplay. . . ' etc.

We agree with the Reviewers comment and appreciate pointing us towards the necessity of the title being more accurate to the research conducted in the paper. Therefore the title was changed in the resubmitted manuscript:

> ~~Reduction of the impact~~ Impact of the local valley splitting on the coherence of conveyor-belt spin shuttling in ^{28}Si/SiGe

Minor comment 2: Figure 1, (particularly section c,d,e) are difficult to read and follow in the present form. Section c was confusing as it doesn't follow right-to-left and only some transition arrows are included. I would recommend figure 1 be full page wide.

We agree with this comment and refer to the resubmitted version for the new Fig. 1 that should be more clear now. A detailed list of edits done to clarify the presentation particularly regarding sections c,d,e follows here:

1. Unfolding the pulse segments such that one experiment run can be discribed in one line going from left to right (new panel c)
2. Clarifying symbolic representation below Roll-in and Roll-out
3. Different positioning of panels d,e in the top right of this figure. Due to panel c being an anchor point this is the only option
4. Deletion of the gate structures in panel c step 3 in order to give more space to panels d,e
5. Addition of an arrow indicating the experiments chronology
6. Enlargement of panel b as more white space became available

Minor comment 3: In Figure 3c) the arrow labelled 2.8m/s interrupts the visibility of the oscillations. This should be resolved as the text specifically compares the oscillation

amplitudes around this region. It is also difficult to judge how much the oscillations are varying by as the additional lines also interrupt the figure. It may be helpful to include an un-edited version of figures 3 b-d in the supplement.

We gladly provide an un-edited version of Fig. 3 b-d in the supplement under a newly introduced Supplementary Note V. Moreover, we added this sentence to the caption of Fig. 3 to raise awareness for this (Line F 3.7)

> A version of panels (b-g) can be found in Supplementary Note V.

The addition of a new Supplementary Note results in changed numbering of references Supplementary notes within the manuscript.

Minor comment 4: The section "Spin decoherence during shuttling" is missing an article. "We start with [...], since the Zeeman energy of the moving QD [...]".

We thank the Reviewer for spotting this mistake. We corrected it as suggested (line 241):

> We start with [...], since the Zeeman energy of the moving QD [...]

Minor comment 5: Could the authors use line number for journal proofs to allow more accurate addressing of sections. This will also help the Reviewers follow any changes you may add.

The markup version of the resubmission contains line numbers as inquired. Every change is indicated with a starting line. Moreover, to improve clarity of our edits, we added them colored in red. We hope to improve legibility and thank the Reviewer for helping us making the review process more pleasant for everybody.

Minor comment 6: Relate to major comment 1, the authors refer to 'valley-orbit' states. Are you able to comment on the relative energies of the orbital spacing, in order to justify 'valley-orbit' states rather than just 'valley' states.

The Reviewer raises questions about using the wording 'valley-orbit' state. Indeed, within the sentence (line 88)

> First, we (re)load four electrons into the leftmost quantum dot (QD) and wait for 100 µs until the loaded spin mixture has relaxed into two spin-singlet states in the two lowest valley-orbit states available.

we refer to the two valley states in the lowest orbital, with the first excited state being the second orbital lower valley. Here, we want to stress that we relax from a (necessarily) excited orbital in the lowest orbital, therefore the use of the term 'valley-orbit'. To make it more clear what we refer to, we changed this formulation to (line 88)

> relaxed into two spin-singlet states in the two lowest ~~valley-orbit~~ orbital and valley states available

We thank the Reviewer for making us aware of this opportunity for clarification.

Minor comment 7: The acronym 1DEC is defined only in the caption of Fig 1 and the methods section. It would be helpful to define it in the main text.

We thank the Reviewer for pointing us to this opportunity for clarification and have put the following adjustment in place (line 171)

> In order to extend to a two-dimensional map $E_{\mathrm{VS}}(d, y)$, we repeat the measurement protocol above and record seven traces that are offset by 6 nm perpendicular to the one-dimensional electron channel (1DEC) (see Fig. 1a).

III. REVIEWER 4

This report carries on with the techniques and approaches shown in a previous Volmer et al. paper [npj Quantum Inf. 10, 61 (2024)] and advances that work by investigating and furthering the understanding of the specific error mechanisms associated with spin dephasing when shutting through low lying valley regions along a conveyor-mode transport path. Specifically, the demonstration of populating the excited valley state when moving through a very low valley state, even when de-tuned from the spin-valley hot-spot, is an important and notable observation. Additionally, the Supplementary Information (as well as main text) provides important analyses and methodologies which would provide a valuable resource to other interested in utilizing these approaches. I recommend the paper for publication, though I have listed several comments and questions below, which I feel should be addressed:

We thank the Reviewer for their positive assessment of our work and for recommending it for publication. We are particularly grateful that the Reviewer recognizes the importance

of our observations regarding the population of excited valley states during passage through low valley splitting regions. We are also pleased to hear that the Reviewer finds the analyses and methodologies in the Supplementary Information to be a valuable resource for the community. We have addressed the specific comments and questions below.

(1) I have a couple suggestions/comments for figure 1. If there is room, pictorially showing where the shuttling (QuBus) device gates (S1,S2,S3,S4) are along d. My thought here would be to place cartoon gates, high-lighting the gate widths and small gapes from inter-gate dielectrics, above 1(d). It would be good to see if any of the peaks/troughs of the valley splitting coincide with locations where the QD is residing under, or shared between, two S gates. Would the authors expect this to be the case? Also, is the (slight) horizontal feature at 0.95T in 1(d) the valley hot-spot associated with the static QD? It would be worth identifying//commenting on this as one would expect a valley feature from that interaction as well.

We gladly add the mentioned modification true to scaling in distance d to the top of panel d. Moreover, we added the following sentence to the caption (Line F.1.9):

> Gate positions and widths true to d are indicated by symbols on top.

The Reviewer moreover inquires about the expected behavior of the valley splitting in the area between two gates. Here, two physical effects can be expected. Firstly, the shuttle pocket is subject to breathing changing the orbital splitting and thus the alloy area covered by the wavefunction. Secondly, thermal compression can add local strain to the alloy in the vicinity of the gate edges boosting the valley splitting.

Regarding the correlation of gate boundaries with valley splitting peaks/troughs, we analyzed the correlation of all valley splitting traces by plotting each datapoint as a function of the distance to its closest gate (see Fig. 1 semitransparent points) and compute their average (bold line). We do not see any systematic correlation in this analysis.

Regarding the mentioned horizontal feature at 0.95 T, the Reviewer astutely suggests that it might relate to the valley hot-spot of the static QD, similar to Ref. 43. We believe that this is probable and therefore we add to the text (line 154):

> Indeed, we track $E_{\mathrm{VS}}(d)$ despite some P_{S} background due to the static QD (at $B = 0.95\,\mathrm{T}$) and some vertical features [...].

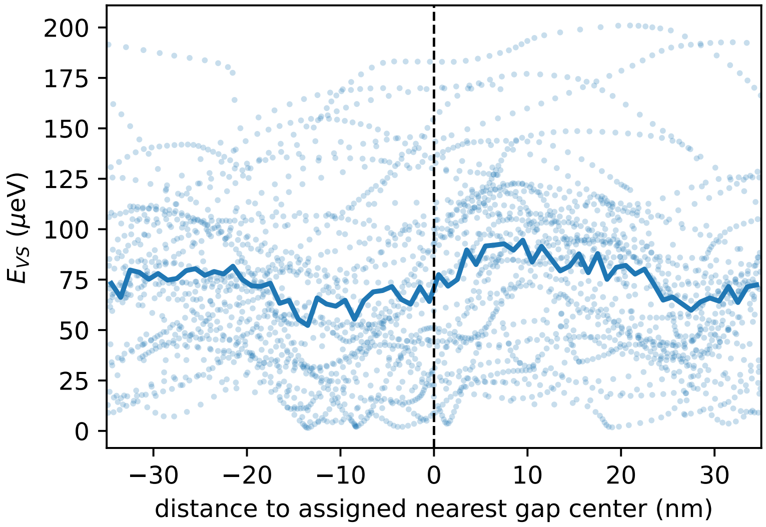

FIG. 1. Correlation of the Valley splitting to the distance to the closest gap center.

(2) Is it possible to generate a plot of $\Delta g(d, y)$,similar to 2(d)? Does this information fall out of the plots like 1(d) (with some math to deconvolve the integrated vs local g-factor difference)? This would be of great interest to the community, even if only in the supplementary information. Along these lines, are there any correlations between E_{VS} and Δg (or in the locations of rapid change in either value)?

In line with our train of thought, the Reviewer requests a plot of $\Delta g(d, y)$. This is something we plan on measuring, however it is beyond the scope of this work. We have just published a preprint on this measurement available at https://arxiv.org/abs/2603.01844

(3) I feel there should be some comment on or comparison to the expected (theory) or observed (experimental) correlation lengths from literature. How does this compare to measured or theorized alloy disorder correlation lengths.

The valley splitting correlation length inferred from the experiments is very close to the expected QD size. This is consistent with expectations following from theoretical models of spatial dependence of valley splitting due to alloy disorder at the interface (Paquelet Wuetz et al, Nat. Comm. (2022), Losert et al, PRB (2023), both cited in the paper), justifying our use of formula (2) that follows from these models. We have clarified this by changing the text near Eq. (2) in the following way:

Fitting the acf and thus the size of the shuttled QD by the formula that applies

when spatial randomness of E_{VS} is due to alloy disorder at the interface [43, 48]

$$\mathrm{acf}(d) = \exp\left(-\frac{1}{4-\pi}\frac{d^2}{a_{\mathrm{dot}}^2}\right), \tag{1}$$

we find a QD ~~size~~ radious of ~~$a_{\mathrm{dot}} = 17.3(1)\,\mathrm{nm}$~~$a_{\mathrm{dot}} = 18.2(2)\,\mathrm{nm}$, similar to Ref. [48] (16 nm) and Ref. [44] (19.2 nm).

(4) I do understand the assignment of the rising background in figure 4b to a slow singlet triplet oscillation. This oscillation would be limited by the same T_2^* dynamics as the faster oscillation and would decay on a similar 2 μs timescale. Is there another mechanism that may be plausible? Is this a measurement artifact?

We would like to thank for this insightful observation regarding the rising background in Figure 4b. The Reviewer is entirely correct that assigning this feature to a slow singlet-triplet oscillation presents a contradiction; such an oscillation would indeed be subject to the same T_2^* dephasing dynamics as the faster components and should therefore decay on a comparable 2 μs timescale, rather than persisting as observed. We must acknowledge that this rising background remains the one feature of our dataset that we cannot currently explain with certainty. While we have ruled out simple decoherence mechanisms, plausible hypotheses include the beating of multiple components or the unaccounted-for relaxation of an unintended spin state initialized during the pulse sequence. This will be a point of interest in future experiments.

(5) Over how much time were the dephasing measurements averaged (T2* and T in Table I and in the discussion of Figure 4)? i.e. do they represent ergodic numbers. It would be good to explicitly state this somewhere.

The measurements were taken over time spans of about 10 minutes each. We appreciate this comment and add the following sentence to clarify this in the main text (lines 389-391).

> [...] is fitted. Each measurement was taken over the span of approximately ten minutes.
>
> First, we select [...]

(6) How does the 92% shuttle fidelity compare to results in the literature? There have been a lot of exciting results recently.

We thank the Reviewer for this comment which likely refers to the most recent results from de Smet et al. in Nature Nanotechnology. While we did cite these results prominently

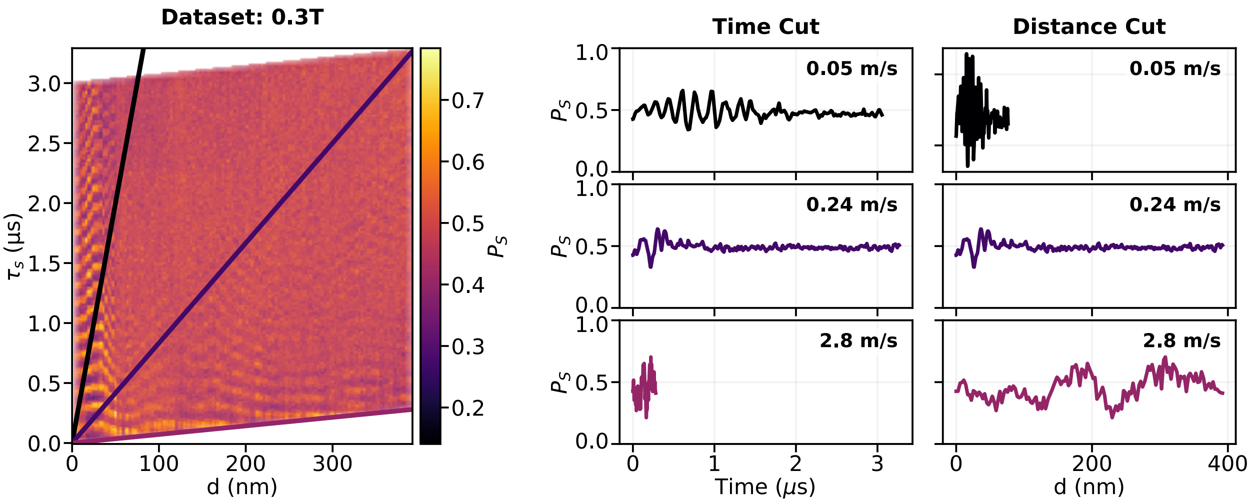

FIG. 2. Linecuts through Fig. 3c from the main text along different velocity axes. The linecuts are plotted both as a function of shuttle time as well as shuttle distance.

in the introduction of the work, we agree that they deserve a spotlight when addressing our shuttling fidelities as well as reasons for why they achieve larger fidelities. For this we added the following sentence in lines 533-537.

> [...] for the spin singlet. Comparable literature focused on high-fidelity spin shuttling [37], surpasses this. As we did not optimize the experiment for fast shuttle speeds and have additional decay due to the static electron, this is expected.

(7) In the discussion of figures 3(b,c,d), there is a comment in the bottom left paragraph of page 5 that higher velocity shuttling better preserved coherence (since the oscillation amplitude is unchanged). This is not clear to me from the data, how is this quantified?

In Fig. 2 we present a selection of linecuts at different velocities plotted as a function of both shuttle time and distance. One can observe that the slow oscillation in the bottom right panel (taken at $v_S = 2.8\,\mathrm{m\,s^{-1}}$) is still visible way beyond the 50 nm mark, whereas for slower velocities (where the spin resides within the hotspot for a longer time), the oscillation strength drops almost immediately. One caveat with these linecuts is that the slow variation of visibility (slow axis is the distance axis) introduces added variations which is why we excluded this analysis from the main and supplementary material.

(8) A reference (or references) for the realistic spin-valley-coupling of near 300neV would be helpful.

We thank the Reviewer for this request. While values approaching 300 neV are on the higher end of the spectrum, spin-valley coupling magnitudes in the range of 100 neV to 200 neV have been consistently reported in SiMOS devices, for example in Hao *et al.*, Nat. Comm. **5**, 3860 (2014), Hwang *et al.*, Phys. Rev. B **96**, 045302 (2017), and Jock *et al.*, Nat. Comm. **13**, 641 (2022).

In Si/SiGe, reported values have typically been lower. However, in our concurrent work on the same heterostructure (Ref. 55 in the manuscript, appended here as a preliminary version), we experimentally extract a spin-valley coupling of approximately 150 neV. This suggests that in the regime of low valley splitting (where mixing is strong), couplings exceeding 100 neV are realistic for Si/SiGe as well.

(9) Spin-valley coupling has been observed to dephase S/T qubits when operated near a valley hot-spot. How does this effect compare to that?

This is basically the same effect. In experiments on singlet-triplet qubits one can use the presence of spin-valley resonances to initialize (by appropriate time-dependence of detuning sweep) superpositions of valley states that are then subjected to dephasing due to valley splitting fluctuations. Examples of papers containing such experiments are Refs. 14 and 15 cited in the Supplementary Information of our paper. In Fig. 3 we see effects of qubit motion through a spin-valley resonance that results at low velocities in adiabatic transformation of a spin superposition into a valley state superposition, as explained in more detail in Supplementary Note VII. The suppresion of S-T oscillations when such adiabatic passages occur is, in our interpretation, due to dephasing of this valley state superposition. The adiabatic-to-diabatic change of character of dynamics at the spin-valley resonance as function of shuttling velocity allows us to identify this mechanism, and use the measurements shown in Figure 3 to characterize the encountered spin-valley hotspots.

(10) The article indicates the dot location is found through methods used in Ref 43; triangulation and Electrostatic simulation of full device in COMSOL. How does the dot size calculated from the auto correlation function compare to these simulations? A naïve picture might have the QD move like an inch-worm along the path expanding when the QD is residing under, or shared between, two S gates (since there is a dielectric gap between the gates) and contracting when it is fully located under a single QD. Does modeling show a smooth movement of the QD wavefunction?

In the supplementary information of Ref 43 (supplementary Figure 7), we used triangula-

tion by capacitive coupling of surrounding gates to a quantum dot formed in the channel to calibrate the displacement along the y-coordinate by the voltages applied to the screening gates. The spread of experimentally found y-displacement was in line with electrostatic simulations including electrostatic disorder. The Reviewer's picture of an inch-worm refers to what we call breathing and wobbling of the quantum dot when shuttled along the channel. The electrostatic simulation of this effect, including disorder from charged defects at the oxide interface, was the topic of section "III. OPTIMIZATION OF GATE DESIGN FOR CONVEYOR-BELT MODE SHUTTLING" of Ref. 34. The orbital splitting was plotted in Fig. 7d in that paper. We optimized the pitch of the gate structure and depth of the quantum well such that the "deterministic" variation of the orbital splitting is about the same as the "random" variation of orbital splitting due to the electrostatic disorder. The orbital splitting there is between 1 to 1.6 meV. In our device we use a thicker oxide and a higher driving voltage. The quantum dot radius of 18 nm is in line with the range of orbital splittings to be expected.

Singlet-triplet oscillations in multivalley Si double quantum dots

Łukasz Cywiński*
Institute of Physics, Polish Academy of Sciences, al. Lotników 32/46, PL 02-668 Warsaw, Poland

Mats Volmer, Tom Struck, and Lars R. Schreiber†
JARA-FIT Institute for Quantum Information, Forschungszentrum Jülich GmbH and RWTH Aachen University, Aachen, Germany

Charge separation from $(4,0)$ to $(3,1)$ state in a Si/SiGe double quantum dot was used in recent experiments involving creation of $(3,1)$ singlet, and subsequent shuttling of one of the electrons. We present a theoretical description of the process of charge separation and singlet-triplet mixing, arriving at expressions for singlet return probability that take into account experimentally observed finite probability of creation of singlets with various patterns of valley occupations. In our analysis we focus on magnetic fields for which electron spin Zeeman splitting is close to the valley splitting in one of the dots, when the spin-valley coupling causes strong renormalization of frequency of oscillations of singlet return probability. The latter effect has been recently used to perform valley splitting mapping by shuttling of one quantum dot to various locations with respect to the other. We give a detailed description of singlet-triplet dynamics near these spin-valley resonances and compare the results of calculations with measurements on double quantum dots in two distinct Si/SiGe heterostructures. Comparison of theory with experiments in which presence of a few valley occupation patterns is visible, gives insight into the valley dependence of g-factors in these structures, providing support for recently proposed theoretical model of this dependence. We also discuss how dephasing of singlet return probability oscillations near the spin-valley resonances is affected by valley splitting fluctuations caused by electric field noise.

I. INTRODUCTION

Electron spin qubits in silicon-based quantum dots - either Si/SiGe or SiMOS heterostructures - have achieved the level of gate errors and initialization/readout fidelities [1–8] compatible with scalability of quantum computer based on them. Electrons in silicon have weak spin-orbit coupling, making their spins less sensitive to charge noise and their g-factors less random than those of hole spins in silicon or germanium-based quantum dots [9]. Weak spin-photon coupling following from small spin-orbit interaction strength makes long distance coherent coupling between electron spin qubits challenging [10, 11]. Architectures based on long-distance coherent shuttling of electrons in silicon were proposed [12–15] to obviate the need for fine-tuning of spin-photon coupling, and rapid progress in long-distance shuttling of electrons [16–18] and coherent shuttling of electron spin qubits [19–23] has been made recently. However, using of electrons in Si as spin qubits comes with another drawback: the presence of two low-energy valley states [24–27] having energy splitting that is typically < 100 μeV in Si/SiGe structures. The mere presence of two valley states (not to mention the possibility of electron in a quantum dot occupying both of them with finite probability) poses challenges for qubit intialization, control, and readout [7, 28], motivating intense research aimed at understanding the physics controlling the magnitude of valley splitting [29–34]. Of particular significance for this Paper is the existence of spin-valley coupling enabled by interplay of spin-orbit coupling and valley-orbit mixing by interface roughness [35–38]. This coupling strongly mixes spin and valley degrees of freedom whenever the Zeeman splitting for an electron is a given quantum dot, E_Z, is close to the valley splitting, E_{V}, in this dot, i.e. when the qubit is close to the spin-valley resonance.

Possibility of exciting a silicon spin qubit to a higher-energy valley state during electron motion has to be carefully optimized against when shuttling qubits [12, 39–41]. On the other hand, shuttling of spin superposition states has recently been shown to be a useful tool for characterization of spatially random valley splitting over large areas of Si/SiGe quantum well [42, 43]. In the experiments [21, 42] a spin singlet is separated spatially in a double quantum dot (DQD), and then one of the dots is moved to a desired location, at distance d from its initial position, using conveyor-mode charge shuttling [12, 21]. Dynamics in the singlet (S) and unpolarized triplet (T_0) subspace is sensitive to difference of spin splittings in the two dots, given by $\Delta g \mu_B B$, where $\Delta g \lesssim 10^{-3}$ is the typical magnitude of g-factor difference for two Si/SiGe quantum dots [28, 42, 44]. When magnetic field is such that the spin qubit in the shuttled dot is close to the spin-valley resonance, spin-valley coupling leads to renormalization of the Zeeman splitting of the qubit [21, 45]. While such a renormalization amounts to a small ($< 10^{-3}$) fraction of E_Z of a single spin, it leads to a $\sim 100\%$ renormalization of singlet-unpolarized triplet splitting given by $\Delta g \mu_B B$. Subsequent shuttling of one of the dots back to its original position, followed by performing the Pauli spin block-

* lcyw@ifpan.edu.pl
† lars.schreiber@physik.rwth-aachen.de

ade (PSB) readout of singlet/triplet state of two spins in the DQD, allows for measurement of singlet-triplet precession that is very sensitive to the value of $|E_{\rm V} - E_Z|$ at the position d, at which most of the spin dynamics took place. This sensitivity leads to presence of characteristic discontinuities in (E_Z, d) map of the singlet return probability, and thus to reconstruction of $E_{\rm V}(d)$ dependence [42, 43].

In this Paper we give a detailed theoretical analysis of the singlet-return-probability signal in a setup used in [21, 42, 46], in which a charge separation from $(4,0)$ to $(3,1)$ state is used to initialize a spatially delocalized spin singlet. Our main focus is on ranges of magnetic fields for which the spin splitting in one of the dots is close to the valley splitting, so the spin-valley couplings in both dots are the crucial interactions in our DQD model [45, 47–50]. We give particular attention to the possibility of creation of finite occupation of more than one valley occupation pattern for $(3,1)$ singlet that leads to the presence of additional frequencies in the measured $P_S(t)$ signal. The presence of such additional frequencies was noted in the first experiments [21], but subsequent analysis of spin-valley resonances in [42] was done for one chosen frequency branch, while the results for $E_{\rm V}(d)$ mapping were independent on the choice of the branch, as we explain in detailed manner in this Paper. Comparison of theoretical predictions with detailed measurements of B dependence of $P_S(t)$ signals (actually, the frequency dependence of Fourier-transformed singlet return probability signals) near the spin-valley resonances in devices from two distinct Si/SiGe heterostructures, used in [42] and [43], respectively, gives support to the recently proposed model of valley-dependence of g-factors in Si/SiGe quantum dots [51, 52]. Furthermore, we discuss the dephasing of the $P_S(t)$ signal near the spin-valley resonances caused by fluctuations in both E_Z and $E_{\rm V}$, and uncover that in the vicinity of the spin-valley resonance it is the valley splitting noise that can dominate the decay of the signal, especially in isotopically purified samples.

The paper is structured in the following way. In Section II we describe the model for the DQD under consideration, focusing in Sec. II A on single-electron Hamiltonian in a silicon DQD, and proceeding in Sec. II B to description of the model describing the physics of 4 electons in $(4,0)-(3,1)$ charge occupations of the DQD. We provide a detailed description of the spin-valley mixing for all the possible valley occupations of $(3,1)$ singlets that can be created by detuning sweep leading to transfer of one electron from the left dot to the right one. In Sec. III we focus on the possibility of charge separation leading to initilization of mixture of two or more $(3,1)$ singlets with distinct valley occupation patterns. We first briefly discuss III A in the possible reasons for nonperfectly adiabatic character of charge separation during detuning sweep from $(4,0)$ to $(3,1)$, and then discuss in Sec. III B the impact of such non-adiabaticity on the singlet return probability signal observed in the PSB measurement. Since the final result of this Section is that the signal is expected to be a sum of signals corresponding to the singlets that are created with nonzero amplitude, in Section IV we analyze the magnetic field dependence of the signal for a given singlet, paying particular attention to behavior near one of spin-valley resonances. In Section V we include in these calculations the effects of fluctuations of spin and valley splittings in the two dots. With theoretical model of singlet return probability in presence of occupation of multiple valley states and dephasing, we proceed in Section VI to analysis of experimental results obtained in DQDs in two distinct Si/SiGe structures used in recent experiments on long-distance shuttling of one of the dots [42, 46]. In Sec. VI A we describe the experimetnal results and the parameters obtained from fitting the theoretical model to them, and in Sections VI B and VI C we discuss, respectively, the implications of these observations for magnitude of valley splitting fluctuations, and valley-dependence of g factors in Si/SiGe quantum dots.

II. THE MODEL

A. Single electron in a double quantum dot

We describe a single electron in a silicon DQD taking into account the lowest energy orbital states in left (L) and right (R) dot, the two lowest energy valley eigenstates per dot, labeled by ν_D with $\nu = g$ (e) for the ground (excited) valley state in dot $D = L/R$, and the electron's spin degree of freedom $\sigma = \uparrow, \downarrow$. The single-electron basis is thus spanned by $|\nu_D \sigma\rangle$ states. The valley eigenstates in given dot, $|\nu_D\rangle$, are eigenstates of the valley coupling Hamiltonian [24–27, 53–55]:

$$\hat{H}_v = \begin{pmatrix} 0 & \Delta_D \\ \Delta_D^* & 0 \end{pmatrix} , \tag{1}$$

written in the basis of $\{|+z\rangle, |-z\rangle\}$ states (Bloch waves at minima of conduction band along the [001] crystal direction located at $\pm k_z \approx 0.852\pi/a_0$ where a_0 is the lattice constant of Si), in which $\Delta_D \equiv |\Delta_D| e^{-i\phi_D}$ is the valley coupling, the values of which is determined by the structure of the interface (Si-SiGe interface in case of Si/SiGe structures) on the atomic lengthscale [24–27, 53–55]. The splitting between the valley eigenstates in dot D is thus given by $E_V^D = 2|\Delta_D|$. According to the current theoretical [29–31, 55] understanding that is supported by the newest experiments on statistic of valleys splitting [42, 43, 56], the value of Δ_D in Si/SiGe quantum dots is determined by alloy disorder. An important result of this model is the observation that for two dots (labeled L and R) for which the ground state envelope functions have little overlap, real an imaginary parts of Δ_L and Δ_R are Gaussian-distributed and approximately uncorrelated random variables [31].

The Hamiltonian can be written as a sum of spin-diagonal terms $|H\rangle_0$, Zeeman splitting term $\hat{H}_Z$, and the

spin-valley coupling $\hat{H}_{sv}$:

$$\hat{H}_{\rm se} = \hat{H}_0 + \hat{H}_Z + \hat{H}_{sv} \ . \quad (2)$$

The spin-diagonal term is given by

$$\hat{H}_0 = \epsilon_L \sum_\nu |\nu_L\rangle\langle\nu_L| + \epsilon_R \sum_\nu |\nu_R\rangle\langle\nu_R| + E_V^L |e_L\rangle\langle e_L| + E_V^R |e_R\rangle\langle e_R| + \sum_{\nu_R,\mu_L} \left(t_{\mu_L\nu_R} |\mu_L\rangle\langle\nu_R| + \text{h.c.}\right) \ , \quad (3)$$

where ϵ_D are the ground state orbital energies in each QD, E_V^D are valley splitting energies in dot D, and $t_{\mu_L\nu_R}$ are tunnel couplings between all the valley eigenstates in the two dots. For less cluttered notation we will omit from now on the L and R subscripts, and use notation $t_{\mu\nu}$ for tunnel couplings, with the first valley subscript referring to the L dot, and the second referring to the R dot. Since the "bare" tunneling t_0 is diagonal in the $|\pm z\rangle$ valley states [54], the tunnel couplings between valley eigenstates in the two dots are determined by the overlaps of the respective eigenstates in the two dots [57, 58]:

$$t_{ee} = t_{gg} = \frac{1}{2} t_0 (1 + e^{-i\Delta\phi}) \ , \qquad t_{eg} = t_{ge} = \frac{1}{2} t_0 (1 - e^{-i\Delta\phi}) \ , \quad (4)$$

where $\Delta\phi = \phi_L - \phi_R$ is the valley phase difference between the two dots.

The valley- and dot-dependent spin splittings are described by

$$\hat{H}_Z = \frac{1}{2} \sum_{D,\nu} E_Z^{\nu_D} |\nu_D\rangle\langle\nu_D| \hat{\sigma}_z^D \ , \quad (5)$$

in which $E_Z^{\nu_D} = g_{\nu_D}\mu_B B_z^D$ are dot- and valley-specific magnetic Zeeman splittings, with B_z^D accounting for possible presence of magnetic field gradient making the B fields at position of each dot distinct, and g_{ν_D} being the dot- and valley-dependent g-factor [51, 52, 59, 60]. When influence of hyperfine coupling with the nuclei of ^{29}Si (or nuclei of ^{73}Ge with which the electron wavefunction has finite overlap [2, 61]) needs to be considered, dot-specific Overhauser fields, h_z^D, should be added to spin splittings $E_Z^{\nu_D}$.

Finally, the spin-valley coupling is described by

$$\hat{H}_{sv} = \sum_{D=L,R} \left(v_D |g_D\uparrow\rangle\langle e_D\downarrow| + v'_D |g_D\downarrow\rangle\langle e_D\uparrow| + \text{h.c.}\right) \ , \quad (6)$$

in which v_i and v'_i are the spin-valley couplings [35, 62]. Their previously reported values range from 20 neV [35] to $100-200$ neV [45, 47, 48] in SiMOS devices, and between $\approx 20-30$ neV [50] and $60-80$ neV [42] in Si/SiGe. In this Paper we will not only revisit the measurement from [42], but also report v in $100-200$ neV range measured in a Si/SiGe heterostructure distinct from the one investigated in that reference. Note that since E_Z and $E_{\rm V}$ are defined to be positive quantities here, the spin-valley term v' couples the states that are never close in energy, so that the presence of this term can be disregarded.

B. Hamiltonian for a system initialized by charge separation starting from $(4,0)$ charge configuration

We consider now a DQD initialized in the ground state with 4 electrons loaded into the L dot, i.e. in the $(4,0)$ charge configuration that is stable for detuning $\epsilon \equiv \epsilon_L - \epsilon_R \ll -|t_0|$. In this state, both valleys are occupied by two electrons, and we can think of each valley as being occupied by a spin singlet [50], and thus we label this state as $|S(4,0)\rangle$, or $|S_0\rangle$ for shorter notation.

This state is tunnel-coupled via $t_{\mu\nu}$ to four states in $(3,1)$ charge configuration. For each of the these states, one of the valleys in the left (L) dot, μ is singly occupied, and the joint state of the electron in this valley and the electron in ν_R valley in the right (R) dot is a singlet. We will thus label these states as $|S_{\mu\nu}\rangle$, specifying the singly-occupied valley states in each of the dots, see Fig. 1a for illustration. All the singlets can also be labelled with $k = 0\ldots4$ index, with $k = 1,2,3,4$ corresponding to $\mu\nu = eg, ee, gg, ge$, respectively. We thus have the following Hamiltonian in the basis of $\{|S_0\rangle, |S_{eg}\rangle, |S_{ee}\rangle, |S_{gg}\rangle, |S_{ge}\rangle\} \equiv \{|S_0\rangle, |S_1\rangle, \ldots, |S_4\rangle\}$ given by

$$\hat{H} = \begin{pmatrix} \epsilon & \sqrt{2}t_{eg} & \sqrt{2}t_{ee} & \sqrt{2}t_{gg} & \sqrt{2}t_{ge} \\ \sqrt{2}t^*_{eg} & 0 & 0 & 0 & 0 \\ \sqrt{2}t^*_{ee} & 0 & E_V^R & 0 & 0 \\ \sqrt{2}t^*_{gg} & 0 & 0 & E_V^L & 0 \\ \sqrt{2}t^*_{ge} & 0 & 0 & 0 & E_V^L + E_V^R \end{pmatrix} , \quad (7)$$

where $\epsilon = \epsilon_L - \epsilon_R + E_V^L + U_L$ (with U_L being the Coulomb energy difference between $(4,0)$ and $(3,1)$ charge configurations), and energy shift of $3\epsilon_L + \epsilon_R + E_V^L$ was applied in order to make the anticrossing of $|S_0\rangle$ and $|S_{eg}\rangle$ states occur at $\epsilon = 0$. The eigenenergies of the above Hamiltonian as functions of ϵ are plotted as solid lines in Fig. 2 for $E_V^R = 53.5$ μeV and $E_V^L = 66.6$ μeV, measured for one of DQDs investigated in [42], with all the tunnel couplings $t_{\mu\nu}$ set to a common value of $t_c = 10$ μeV typical for Si/SiGe DQDs [50].

The Hilbert space of states with $(3,1)$ charge configuration built out of single-electron states used in Hamiltonian (2) is 16-dimensional. Apart from the four $|S_{\mu\nu}\rangle$ states appearing in the above Hamiltonian, it contains 4 groups of $|T^p_{\mu\nu}\rangle$ triplets with $p = 0, \pm1$ denoting the spin projection on the z axis, having energies $E(T^p_{\mu\nu}) = E(S_{\mu\nu}) + p\bar{E}_Z^{\mu\nu}$, where

$$\bar{E}_Z^{\mu\nu} = \frac{1}{2}(E_Z^{\mu_L} + E_Z^{\nu_R}) \ . \quad (8)$$

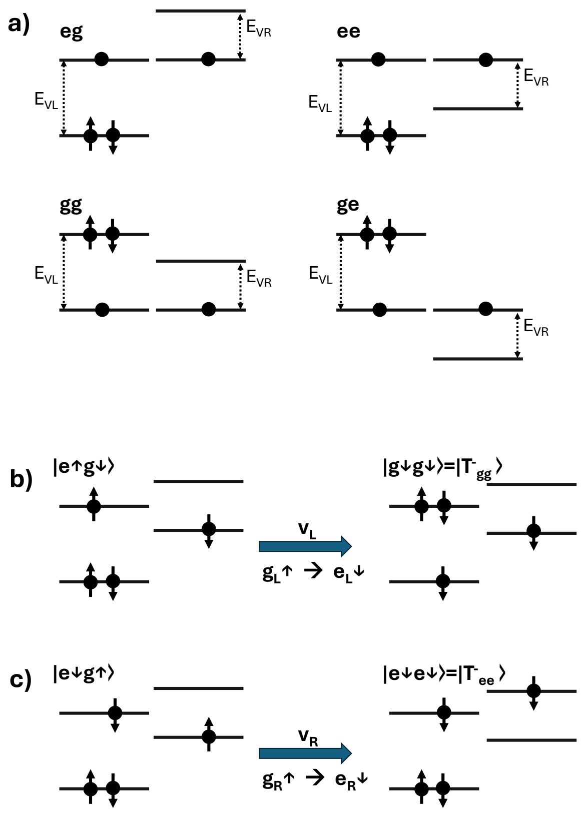

FIG. 1. (a) Occupations of valley states in L and R dots in $(3,1)$ charge configuration. The states in which the μ valley state in dot L and ν valley state in dot R are occupied by single electron are labeled $\mu\nu$. For $E_V^L > E_V^R$, when going clockwise from the eg state we encounter the states that can be created by interdot tunneling as the detuning is increased. For detuning sweep that is adiabatic with respect to t_{eg} tunnel coupling only the S_{eg} state is created; if the sweep is not adiabatic, the subsequent state, S_{ee}, can be created due to t_{ee} tunnel coupling, etc. (b,c) Illustration of states with "eg" valley occupation that are spin-valley coupled. The "eg" singlet with $(3,1)$ charge occupation is written as $|S_{eg}\rangle = (|e\uparrow g\downarrow\rangle - |e\downarrow g\uparrow\rangle)/\sqrt{2}$, and in (b) we see that spin-valley coupling in the left dot, v_L, mixes $|e\uparrow g\downarrow\rangle$ with the $|T^-_{gg}\rangle$ polarized triplet, while in (c) we see how the spin-valley coupling in the right dot, v_R, mixes $|e\downarrow g\uparrow\rangle$ with the $|T^-_{ee}\rangle$ polarized triplet.

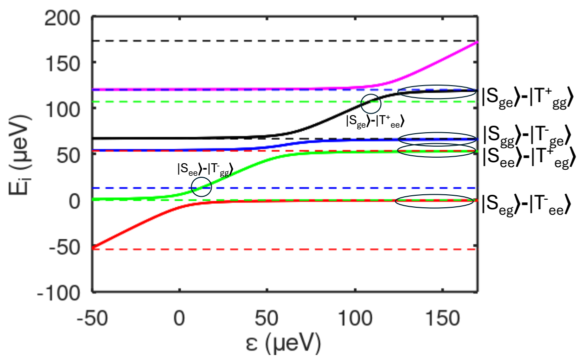

FIG. 2. Energies of singlet states (solid lines) and energies of spin-polarized $(3,1)$ triplet states (dashed lines) that can be mixed by spin-valley coupling with the singlets, calculated for $2\mu_B B = E_V^R$. Some of the spin-valley anticrosssings (funnels) that occur at detunings at which the singlet is a superposition of $(4,0)$ and $(3,1)$ charge states are marked with circles. The 4 regions in which a $(3,1)$ singlet is mixed with a polarized triplet at large detuning are marked by ellipses. These are the spin-valley resonances at which the spin-valley coupling in the right dot affects the singlet dynamics in the far detuned regime, see the lines corresponding to odd r in Table I. Another set of 4 such regions, corresponding to even r in this Table, appears when $2\mu_B B = E_V^L$.

The interdot differences in spin splittings, defined as

$$\Delta E_Z^{\mu\nu} = E_Z^{\mu} - E_Z^{\nu} \ , \tag{9}$$

couple $|S_{\mu\nu}\rangle$ states to $|T^0_{\mu\nu}\rangle$ states [63], while the spin-valley terms v_D, v'_D are coupling $|S_{\mu\nu}\rangle$ and $|T^0_{\mu\nu}\rangle$ states with $|T^{\pm}_{\mu\bar{\nu}}\rangle$ and $|T^{\pm}_{\bar{\mu}\nu}\rangle$ states with $p=\pm 1$ (see Fig. 1(b,c) for illustration in the case of the $|S_{eg}\rangle$ state), where we have introduced the notation in which $\bar{\mu}$ denotes $e(g)$ if $\mu = g(e)$.

These spin-valley interactions mixing $(3,1)$ singlets with the *polarized* triplets are relevant only at values of ϵ at which the unperturbed energies of the states differ by a value that is $\lesssim v_D$. These are the spin funnels analyzed in [49, 50] in the regime of sizable singlet-triplet exchange splitting, $J_{\mu\nu}$, i.e. for detuning with respect to a given anticrossing being of the order of the respective tunnel coupling. An example of such funnels at $|S_{ee}\rangle - |T^-_{gg}\rangle$ and $|S_{ge}\rangle - |T^+_{ee}\rangle$ anticrossings are marked by circles in Fig. 2. On the other hand, mixing of $|S_{\mu\nu}\rangle$ with $|T^0_{\mu\nu}\rangle$ via $\Delta E_Z^{\mu\nu}$ becomes relevant when ϵ is so large that we are in $J_{\mu\nu} \ll \Delta E_Z^{\nu\mu}$ regime in which $S - T_0$ precession due to Zeeman splitting difference can occur. If the Zeeman splitting of the polarized triplets with respect to the S/T_0 states is close to valley spllitting in any of the dots, at large ϵ each $|S_{\nu\mu}\rangle$ state is mixed with both the $|T^0_{\nu\mu}\rangle$ state, and the respective polarized tripled corresponding to a distinct valley occupation pattern. In Fig. 2 we schematically mark such regions by ellipses. Our focus here is on the dynamics of states coupled by Zeeman splitting difference ΔE_Z, *and also the spin-valley coupling* v_D in this far-detuned regime.

In Table I we list the polarized triplets that are appreciably mixed with respective singlets at magnetic fields for which the Zeeman energy $\bar{E}_Z^{\nu\mu} \approx 2\mu_B B$ is close to either E_V^L or E_V^R valley splitting. Let us give now the Hamiltonians governing the dynamics in subspaces containing each of the $|S_{\nu\mu}\rangle$ singlets. For further discussion it is convenient to use the basis of states $|\nu\uparrow\mu\downarrow\rangle \equiv (|T^0_{\nu\mu}\rangle + |S_{\nu\mu}\rangle)/\sqrt{2}$ and $|\nu\downarrow\mu\uparrow\rangle \equiv (|T^0_{\nu\mu}\rangle - |S_{\nu\mu}\rangle)/\sqrt{2}$. Using such a $\{|e\uparrow g\downarrow\rangle, |e\downarrow g\uparrow\rangle, |T^-_{ee}\rangle, |T^-_{gg}\rangle$ basis we have the Hamiltonian describing the coupling of $|S_{eg}\rangle$

singlet to the respective triplets given by

$$\hat{H}_{eg} = \begin{pmatrix} \frac{\Delta E_Z^{eg}}{2} & 0 & 0 & v_L \\ 0 & -\frac{\Delta E_Z^{eg}}{2} & v_R & 0 \\ 0 & v_R^* & E_V^R - \bar{E}_Z^{ee} & 0 \\ v_L^* & 0 & 0 & E_V^L - \bar{E}_Z^{gg} \end{pmatrix}. \tag{10}$$

Analogously, for $|S_{ge}\rangle$ we use the basis of $\{|g\uparrow e\downarrow\rangle, |g\downarrow e\uparrow\rangle, |T_{gg}^+\rangle, |T_{ee}^+\rangle\}$ to obtain

$$\hat{H}_{ge} = \epsilon_{ge}\mathbb{1} + \begin{pmatrix} \frac{\Delta E_Z^{ge}}{2} & 0 & v_R & 0 \\ 0 & -\frac{\Delta E_Z^{ge}}{2} & 0 & v_L \\ v_R^* & 0 & \bar{E}_Z^{gg} - E_V^R & 0 \\ 0 & v_L^* & 0 & \bar{E}_Z^{ee} - E_V^L \end{pmatrix}. \tag{11}$$

in which $\epsilon_{ge} = E_V^L + E_V^R$.

Note that for the system initialized in $|S_{eg}\rangle$ or $|S_{ge}\rangle$, the dynamics due to the above Hamiltonians occur in disjoint four-dimensional subspaces. On the other hand, for the system initialized in $|S_{ee}\rangle$ or $|S_{gg}\rangle$, the dynamics occurs in the same six-dimensional subspace spanned by $\{|e\uparrow e\downarrow\rangle, |e\downarrow e\uparrow\rangle, |g\uparrow g\downarrow\rangle, |g\downarrow g\uparrow\rangle, |T_{eg}^+\rangle, |T_{ge}^-\rangle\}$:

$$\hat{H}_{ee/gg} = \begin{pmatrix} E_V^R + \frac{\Delta E_Z^{ee}}{2} & 0 & 0 & 0 & v_R & v_L \\ 0 & E_V^R - \frac{\Delta E_Z^{ee}}{2} & 0 & 0 & 0 & 0 \\ 0 & 0 & E_V^L + \frac{\Delta E_Z^{gg}}{2} & 0 & 0 & 0 \\ 0 & 0 & 0 & E_V^L - \frac{\Delta E_Z^{gg}}{2} & v_L & v_R \\ v_R^* & 0 & 0 & v_L^* & \bar{E}_Z^{eg} & 0 \\ v_L^* & 0 & 0 & v_R^* & 0 & E_V^L + E_V^R - \bar{E}_Z^{ge} \end{pmatrix}. \tag{12}$$

However, unless the valley splittings in the two QDs are very close to each other, with $|E_V^L - E_V^R| \lesssim |v_D| \lesssim 0.1$ μeV, for any given value of magnetic field the spin-valley interaction in only one of the dots leads to significant mixing of states. Specifically, the $|e\uparrow e\downarrow\rangle$ state mixes with $|T_{eg}^+\rangle$ ($|T_{ge}^-\rangle$) when $E_Z^{eg} \approx E_V^R$ ($E_Z^{ge} \approx E_V^L$), while the $|g\downarrow g\uparrow\rangle$ state mixes with $|T_{ge}^-\rangle$ ($|T_{eg}^+\rangle$) when $E_Z^{ge} \approx E_V^R$ ($E_Z^{eg} \approx E_V^L$). When one of these states is significantly mixed with the respective triplet, the energy of the other is only weakly renormalized by spin-valley coupling with a triplet that is separated by an energy $\gg v_D$. The same holds for $\hat{H}_{eg}$ and $\hat{H}_{ge}$ Hamiltonians from Eqs. (10) and (11): if $|E_V^L - E_V^R| \gg |v_D|$, which is a generic situation for a DQD in Si/SiGe structure, only one of $|\nu\uparrow\mu\downarrow\rangle$ and $|\nu\downarrow\mu\uparrow\rangle$ states is appreciably mixed with the respective polarized triplet, while the shift of the energy of the other one can be treated in second order perturbation theory.

Consequently, in order to understand the dynamics of the system initialized in any of the $|S_{\mu\nu}\rangle$ states in the far-detuned regime, in the generic situation in which $|E_V^L - E_V^R| \gg |v_D|$ we only need to consider dynamics in one of four disjoint three-dimensional subspaces. Depending on the singlet of interest $|S_{\nu\mu}\rangle$, and whether we are interested in magnetic field range in which $2\mu_B B \approx E_V^R$ of E_V^R, we choose one of the 8 case listed in Table I, and in the basis of $\{|\mu\uparrow\nu\downarrow\rangle, |\mu\downarrow\nu\uparrow\rangle, |T_{\alpha\beta}^p\rangle\}$ we have

$$\hat{H}_{\mu\nu,\alpha\beta} = \begin{pmatrix} \Delta E_Z^{\nu\nu}/2 & 0 & v_D(1+\xi)/2 \\ 0 & -\Delta E_Z^{\mu\nu}/2 & v_D(1-\xi)/2 \\ v_D^*(1+\xi)/2 & v_D^*(1-\xi)/2 & p(\bar{E}_Z^{\alpha\beta} - E_V^D) \end{pmatrix}. \tag{13}$$

where $\xi = \pm 1$, $p = \pm 1$, and $D = L$ or R are taken from the row of Table I corresponding to the respective set of $\mu\nu$ and $\alpha\beta$ valley indices (equivalently, respective index $r = 1 \ldots 8$). Note that $\xi = 1$ (-1) means that it is the $|\mu\uparrow\nu\downarrow\rangle$ ($|\mu\downarrow\nu\uparrow\rangle$) that is coupled by v_D to the $|T_{\alpha\beta}^p\rangle$ triplet, while $p = 1$ (-1) means that as B field is increased, thus increasing $\bar{E}_Z^{\alpha\beta}$, the energy of the polarized triplet is approaching the energy of the state in $|S_{\mu\nu}\rangle - |T_{\mu\nu}^0\rangle$ manifold coupled with the triplet from below (above).

Depending on the parameters of the system, in order to precisely capture the frequency of singlet-triplet oscillations, sometimes we have to include the perturbative correction ($\sim |v_D|^2/(E_V^L - E_V^R)$, where $D' = L(R)$ when $D = R(L)$) to the unperturbed energy of the state that is not mixed with its corresponding triplet in this Hamiltonian. In the following, we will show results of numerical calculations performed using appropriate 4-dimensional Hamiltonians (note that the nontrivial part of the Hamiltonian (12) acts in a 4-dimensional subspace), but these results will be discussed with the help of generally very accurate analytical formulas obtained using the Hamiltonians from Eq. (13).

r	$S_{\mu\nu}$	Resonance	$T^p_{\alpha\beta}$	D	ξ	p
1	S_{eg}	$\bar{E}_Z^{ee} \approx E_V^R$	T^-_{ee}	R	-1	-1
2	S_{eg}	$\bar{E}_Z^{gg} \approx E_V^L$	T^-_{gg}	L	$+1$	-1
3	S_{ee}	$\bar{E}_Z^{ge} \approx E_V^R$	T^+_{eg}	R	$+1$	$+1$
4	S_{ee}	$\bar{E}_Z^{ge} \approx E_V^L$	T^-_{ge}	L	$+1$	-1
5	S_{gg}	$\bar{E}_Z^{ge} \approx E_V^R$	T^-_{ge}	R	-1	-1
6	S_{gg}	$\bar{E}_Z^{eg} \approx E_V^L$	T^+_{eg}	L	-1	$+1$
7	S_{ge}	$\bar{E}_Z^{gg} \approx E_V^R$	T^+_{gg}	R	$+1$	$+1$
8	S_{ge}	$\bar{E}_Z^{ee} \approx E_V^L$	T^+_{ee}	L	-1	$+1$

TABLE I. Parameters of the singlet-triplet mixing Hamiltonian from Eq. (13) for all combinations of $|S^{\nu\mu}\rangle$ singlets and polarized $\left|T^p_{\alpha\beta}\right\rangle$ states for which spin-valley mixing resonance occurs at values of Zeeman splitting given in the third column.

III. EFFECTS OF NONADIABATIC CHARGE DYNAMICS ON SINGLET RETURN PROBABILITY

During a perfectly adiabatic sweep of detuning from $(4,0)$ to $(3,1)$ charge regimes the $|S_{eg}\rangle$ state should be created if t_{eg} is finite - and if we have $t_{eg}=0$, then t_{ee} and t_{gg} have to be finite, and either $|S_{gg}\rangle$ or $|S_{ee}\rangle$ will be created, depending in which of the dots the valley splitting is larger. However, experiments on charge separation and subsequent shuttling of R dot in Si/SiGe structures in [21] showed signatures of initialization of more than one $S(3,1)$ state, as the singlet return probability signals for R dot shuttled for distance d and back contained clear signatures of presence of two frequencies, corresponding to two distinct spatial dependencies of g-factors of the shuttled qubit. In this Section we focus on the possible reasons for initialization of mixture of two (or more) singlets, and its consequences for the singlet return probability signal.

A. Mechanisms leading to nonadiabaticity during charge separation

We assume that the system is initialized in the $S(4,0)$ state at negative detuning. When ϵ is then changed very slowly to a positive value $\epsilon \gg t_{eg}$, so that the dynamics is truly adiabatic, the state reached in the $(3,1)$ regime is the $|S_{eg}\rangle$ singlet. More precisely, the modulus squared of overlap of the final state with $|S_{eg}\rangle$ is given by $P_{eg} \equiv 1 - Q_{eg} \approx 1$, where the small probability electron not being transferred to the R dot at the first anticrossing in Figs. 2 and 3 is given by the Landau-Zener formula [64],

$$Q_{eg} = \exp\left(-4\pi|t_{eg}|^2/\hbar v_\epsilon\right) \tag{14}$$

where v_ϵ is the detuning sweep rate, i.e. $\epsilon(t) \propto v_\epsilon t$ near the anticrossing. Now it is clear that for the sweep to be "slow enough" to adiabatically create the $|S_{eg}\rangle$ state we need $v_\epsilon \ll 4\pi|t_{eg}|^2/\hbar$. For $|t_{eg}|=10$ μeV this means $v_\epsilon \ll$ 2000 μeV/ns, but for $|t_{eg}|=1$ μeV this means $v_\epsilon \ll 20$ μeV/ns. In [42] the estimated detuning sweep rate was in the $v_\epsilon \in [500, 2000]$ μeV/ns range, so an assumption of fully adiabatic charge separation might not be justified: if $v_\epsilon = 2000$ μeV then $Q_{eg} < 0.1$ only when $|t_{eg}| > 15$ μeV.

Another possible cause of failure of adiabatic charge separation, which in fact becomes more relevant as the detuning sweep rate is decreased, is high-frequency charge noise in detuning [65, 66]. When $|t_{\mu\nu}| \lesssim 10$ μeV, fluctuations in detuning that have appreciable spectral density at frequencies in GHz range can lead to excitation of the system out of its instantaneous ground state to a higher energy state when the two are mixed by tunnel coupling near the tunnel-induced anticrossing. Let us denote the corresponding excitation rate at the anticrossing of $|S_0\rangle$ with $|S_{\nu\mu}\rangle$ by $\Gamma_+^{\nu\mu}$. As discussed in [66], the resulting probability of "incoherent" (i.e. detuning noise-induced) failure of charge separation at given anticrossing, scales as $\Gamma_+^{\nu\mu}/v_\epsilon$, as slower sweep leads to longer time spent at the ancticrossing. When the tunneling induced gap $2t_{\nu\mu}$ is larger than $k_B T$, the excitations are exponentially suppressed, $\Gamma_+^{\nu\mu} \propto \exp(-2|t_{\mu\nu}|/k_B T)$. However, for typical temperature $T=100$ mK and $|t_{\mu\nu}| \leq 10$ μeV we have $2|t_{\mu\nu}|/k_B T \leq 2$, and such a suppression is rather weak. For such low tunnel couplings high-frequency charge noise can thus become quite efficient at introducing charge separation errors during the detuning sweep.

After a failure of adiabatic charge separation at the first enountered antocrossing, the $(4,0)$ state can become converted into $(3,1)$ state at the subsequently encountered tunnel-induced anticrossing of singlets, see Fig. 3 for an illustration. With ϵ varying linearly with time, the Hamiltonian from Eq. (7) corresponds to a 5-level generaliation of the Landau-Zener problem, with energy of $|S_0\rangle$ state crossing through energies of four $(3,1)$ singlet states. Fortunately, in the relevant here case of a single level crossing multiple levels, the unperturbed energies of which are parallel lines as functions of ϵ, there exists an exact solution for the occupations of all levels after the end of the deuning sweep [67]. Labeling here the $(3,1)$ singlets with $k=1\dots4$ index in the order in which the anticrossings with $|S_0\rangle$ state occur, the probability of creation of k-th charge separated singlet, p_k^∞ by sweep of detuning to large positive values is given by

$$p_k^\infty = (1-Q_k)\prod_{n=1}^{k-1} Q_n \ , \tag{15}$$

where Q_k is given by Eq. (14) with an appropriate t_k, and the probability of failure of charge separation, i.e. the occupation of the $(4,0)$ state (the $|S_0\rangle$ singlet), is given by

$$p_0^\infty = \prod_{n=1}^{4} Q_n \ . \tag{16}$$

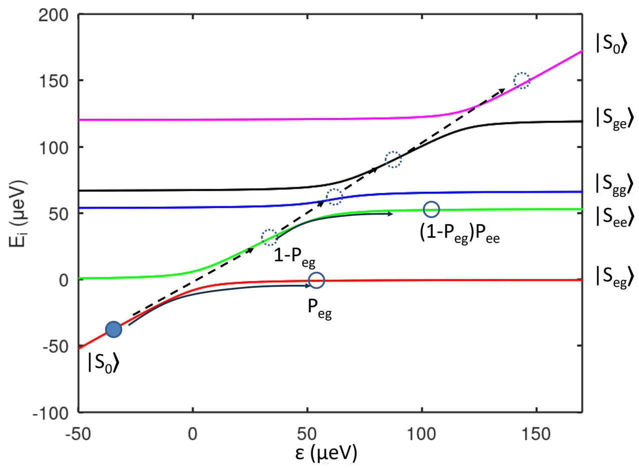

FIG. 3. Solid lines: spectrum of Hamiltonian (7) as a function of detuning ϵ for $E_V^R = 53.5$ μeV, $E_V^L = 66.6$ μeV, and all tunnel couplings equal to $t_c = 10$ μeV. $|S_0\rangle$ is the singlet ground state of the $(4,0)$ charge configuration, while $|S_{\nu\mu}\rangle$ are the 4 singlets with $(3,1)$ charge configuration, with valley occupations depicted in Fig. 1. Solid (dashed) lines with arrows show adiabatic (diabatic) evolution pathways: system initialized in $|S_0\rangle$ at large negative detuning upon detuning sweep evolves to $|S_{eg}\rangle$ state with probability P_{eg}, while it stays in $|S_0\rangle$ with probability $Q_{eg} \equiv 1 - P_{eg}$, and subsequently it can adiabatically evolve into $|S_{ee}\rangle$, with the probability of populating that state being $(1 - P_{eg})P_{ee}$, etc. Pathways of subsequent evolution, with exception of the "fully diabatic" one for which the system stays in $|S_0\rangle$ state at the end of detuning sweep, and probabilitites of reaching other states, are not shown for clarity of the Figure.

B. Contributions to the singlet return probability signal

If we do not assume that the detuning sweep is adiabatic, we have to contend with a multi-level Landau-Zener problem defined by Hamiltonian (7) with $\epsilon(t) \propto v_\epsilon t$. At the end of the sweep from negative to positive ϵ, the density matrix of the system is given by

$$\hat{\rho}(0) = \sum_{k=0}^{4} p_k^\infty |S_k\rangle \langle S_k| + \sum_{k\neq k'} c_{k,k'} |S_k\rangle \langle S_{k'}| \ , \quad (17)$$

where $k = 0, \ldots, 4$ labels all the singlets, see Eq. (7), p_k^∞ are the final occupations of all the singlet states (with p_0^∞ being the probability of *failure* of charge separation), and $c_{k,k'}$ are the coherences between the singlets. Subsequent evolution due to the total Hamiltonian (which is a direct sum of $\hat{H}_{\mu\nu}$ Hamiltonians from Eqs. (10), (11), and (12)), leaves p_k^∞ unchanged, while the coherences become time-dependent.

We now assume that these $c_{k,k'}(t)$ dephase to zero on the timescale of nontrivial dynamics and decay of $P_S(t)$ signal. This is a very natural assumption for $c_{0,k} = c_{k,0}^*$ coherences between states with distinct charge distribution, as these decay due to charge-noise induced fluctuations of energy levels in the two quantum dots (for survey of reported magnitudes on charge noise in Si/SiGe QDs see [68]), and the characteristic timescale of decay of these coherences is $\lesssim 1$ ns. As for $c_{k,k'}$ coherence with $k, k' \geq 1$, they will dephase due to fluctuations of valley splittings, but also due to the following mechanism specific to the way in which they are created. In experiments involving charge separation of spin singlet ans subsequent PSB measurement, the detuning is swept from $\epsilon_i \ll -|t|$ to $\epsilon_f \gg |t|$, the systems is kept at this ϵ_f for waiting time t_w, and then the detuning is swept back to ϵ_i. When two $S(3,1)$ states that differ in energy of E_V (equal to valley splitting in one of the dots, or the sum of these two valley splittings) are populated due to nonadiabatic character of dynamics, the total time spent in the superposition of these states is $t_w + 2(\epsilon_f - E_V)/v_\epsilon$. In a realistically noisy system, the rms of quasistatic shift of detuning due to charge noise is $\sigma_\epsilon \sim 5$ μeV (corresponding to coherence time of DQD charge qubit of $T_{2,c}^* = \hbar\sqrt{2}/\sigma_\epsilon \sim 0.2$ ns). When both ϵ_0 and ϵ_f are shifted by $\delta\epsilon \sim \sigma_\epsilon$, the time spent in the superposition of the two singlets changes by $\delta t \sim 2\sigma_\epsilon/v_\epsilon$. The resulting rms of phase of the relevant $c_{k,k'}$ is $\sigma_\phi \approx E_V \delta t/\hbar$. For $E_V \approx 50$ μeV, $\sigma_\epsilon = 5$ μeV, and the range of $v_\epsilon = 500 - 2000$ μeV/ns used in [21, 42], we have $\delta t \sim 10$ ps that gives $\sigma_\phi \sim 1$. The dephasing due to this mechanism is thus significant, justifying our neglect of coherences $c_{k,k'}$ even at shortest values of t_w considered in experiments. Consequently, we assume that at time t at which the backward sweep of detuning to the PSB regime is initiated the state of the system is given by

$$\hat{\rho}(t) = p_0^\infty |S_0\rangle \langle S_0| + \sum_{\mu,\nu=e,g} p_{\mu\nu}^\infty \overline{|\Psi_{\mu\nu}(t)\rangle \langle \Psi_{\mu\nu}(t)|} \ , \quad (18)$$

in which we have switched back to labeling the $(3,1)$ singlets with $\mu\nu$ indices describing the valley occupation pattern. In this expression the overbar denotes averaging over distributions of all the quasi-statically fluctuating parameters in the Hamiltonian that governs the evolution from the initial $|S_{\mu\nu}\rangle$ states to the final $|\Psi_{\mu\nu}(t)\rangle$ states. The latter can be written as

$$\begin{aligned} |\Psi_{\mu\nu}(t)\rangle &= \exp\Big(-i\hat{H}t/\hbar\Big) |S_{\mu\nu}\rangle \\ &= a_{\mu\nu}(t) |S_{\mu\nu}\rangle + \sum_{m=-1,0,1} b_{\mu\nu}^m(t) \left|T_{\alpha\beta}^m\right\rangle \ , \end{aligned} \quad (19)$$

with $T_{\alpha\beta}^m$ being the appropriate triplet states coupled to $|S_{\mu\nu}\rangle$ by $\hat{H}$, see Table I. As discussed in Sec. II B, instead of using the Hamiltonian acting in 17-dimensional space spanned by $|S_0\rangle$ and 16 states from $(3,1)$ charge configuration, one can use Hamiltonians from Eqs. (10), (11), and (12), or even a Hamiltonian $\hat{H}_{\mu\nu,\alpha\beta}$ from Eq. (13) with appropriately chosen values of subscript indices, in order to model the time dependence of state evolving from a given $|S_k\rangle$ state.

If the sweep of detuning back to ϵ_i, at which the PSB readout will take place, is done adiabatically with respect

to tunneling, i.e. with $v_\epsilon \ll 4\pi|t_{\mu\nu}|^2/\hbar$ for every $t_{\mu\nu}$, each $|\Psi_{\mu\nu}\rangle$ gets converted to $\left|\Psi^a_{\mu\nu}\right\rangle$ in which $|S_{\mu\nu}\rangle$ is replaced by $|S_0\rangle$, so that the final state $\hat{\rho}^a(t)$ is given by Eq. (18) in which each $|\Psi_{\mu\nu}\rangle$ is replaced by such $\left|\Psi^a_{\mu\nu}\right\rangle$ The singlet return probability is then given by

$$\begin{aligned} P_S^{(a)}(t) &\equiv \mathrm{Tr}\Big(|S_0\rangle \langle S_0| \, \hat{\rho}^a(t)\Big) \ , \\ &= p_0^\infty + \sum_{\mu,\nu} p_{\mu\nu}^\infty \overline{|a_{\mu\nu}(t)|^2} \\ &\equiv p_0^\infty + \sum_{\mu,\nu} p_{\mu\nu}^\infty P_{S,\mu\nu}(t), \end{aligned} \tag{20}$$

in which we have identified $\overline{|a_{\mu\nu}(t)|^2}$ with singlet return probability signal, $P_{S,\mu\nu}(t)$, that would be observed when $|S_k\rangle$ state is prepared by charge separation, and then PSB measurement is done after a slow detuning sweep that adiabatically converts $|S_k\rangle$ state into the $|S_0\rangle$ state. The calculation of $P_{S,k}(t)$ when fluctations of parameters in the Hamiltonian is disregarded is detailed in Sec. IV, while in Sec. V the noise-averaged quantity $\overline{|a_{\mu\nu}(t)|^2}$ (i.e. the $P_{S,\mu\nu}(t)$ signal including the effects of dephasing due to quasi-static fluctuations in spin and valley splitting) is calculated.

However, it is more natural to assume that the sweep of detuning back to ϵ_i is a time-reversal of the sweep that resulted in the imperfect charge separation that resulted in state from Eq. (18). In such a case, in each $|\Psi_{\mu\nu}\rangle \langle\Psi_{\mu\nu}|$ density operator in the $\hat{\rho}^{na}(t)$ state obtained by non-adiabatic (na) sweep, the occupation of $|S_0\rangle$ state is given by the occupation of $|S_{\mu\nu}\rangle$ state before the sweep (i.e. $|a_{\mu\nu}(t)|^2$)), multiplied by the probability of converting this state into $|S_0\rangle$. For the time-reversed sweep, this probability is equal to the probability of obtaining $|S_{\mu\nu}\rangle$ state from $|S_0\rangle$ state by the charge-separating sweep, i.e. it is given by $p^\infty_{\mu\nu}$. If the dynamics of diagonal elements of $\hat{\rho}^{(na)}$ is then frozen during the PSB readout, the measured singlet return probability is

$$P_S^{(na)}(t) = (p_0^\infty)^2 + \sum_{\mu,\nu} \left(p_{\mu\nu}^\infty\right)^2 \ P_{S,\mu\nu}(t). \tag{21}$$

It should be stressed that it is by no means obvious that the diagonal elements of $\hat{\rho}^{(na)}$ are indeed frozen during the readout. In fact, in [21, 42] after return sweep of detuning to the PSB range of values, the finite interdot tunnel coupling was maintained for 500 ns before the charge state was frozen in by reducing the tunnel coupling, and the charge state readout but the nearby SET is begun. During this 500 ns long stage, inelastic tunneling from $S(3,1)$ states to the S_0 state is possible. In the simplest scenario in which these inelastic tunneling processes occur after sweeping the detuning to the PSB regime so quickly that any dynamics of $\hat{\rho}^{na}$ except for $S(3,1) \rightarrow S(4,0)$ conversion can be neglected. The resulting singlet return probability signal is then given by Eq. (20) - all the nonadiabatic effects that occured during the sweep of detuning back to ϵ_i are now simply “healed” by inelastic tunneling.

Let us illustrate the above with an example involving only the first two $(3,1)$ singlets — S_{eg} and S_{ee}, labeled here S_1 and S_2 — encountered during the nonadiabatic detuning sweep for ϵ_i to ϵ_f and then back. During the forward detuning sweep, the first of them, S_{eg}, is initialized with probability $p_1 = 1 - Q_1$, where Q_1 is given by Eq. (14). The probability of diabatic passage through the first anticrossing is Q_1, and since the probabilities of subsequent adiabatic (diabatic) passage through the second anticrossing are p_2 ($Q_2 = 1 - p_2$), the occupation of S_2 state at detuning ϵ_f is given by $p_2(1 - p_1)$, in agreement with Eq. (15), while the occupation of S_0 state (the probability of failure of charge separation) is $Q_1Q_2 = (1 - p_1)(1 - p_2) = p^\infty_0$, in agreement with Eq. (16). After evolution for time t, the amplitudes of having $S_{1(2)}$ states are $a_{1(2)}(t)$, see Eq. (19). Next, during the return sweep of detuning, the S_1 state is adiabatically converted into S_0 with probability p_1, leading to $(p_1)^2\overline{|a_1(t)|^2}$ contribution to the $P_S(t)$ signal, while with probability $p_1(1 - p_1)$ the system is “stuck” in S_1. Similarly, for S_2 to be converted in S_0, the state needs to follow a time-reversed path of adiabatic passage through the second anticrossing and a *diabatic* passage through the first one, so that the resulting contribution to $P_S(t)$ is $[p_2(1 - p_1)]^2\overline{|a_1(t)|^2} = (p^\infty_2)^2\overline{|a_1(t)|^2}$. The “wrong” pathways of evolution lead to final occupation S_2 of $(1 - p_2)p^\infty_2$, and to an additional contribution of occupation of “stuck” S_1 state given by $p_2p_1p^\infty_2$. Finally, the S_0 remains occupied after the backwards sweep with probability $(p^\infty_0)^2$, while its evolution during this sweep contributes $p_2p^\infty_0$ to occupation of S_2 and $(1-p_2)p_1p^\infty_0$ to occupation of S_1. If we now assume that the occupations of S_1 and S_2 states are rapidly converted by inelastic tunneling into occupations of S_0, the terms in Eq. (21) are modified in the following way:

- The contribution to $P_S(t)$ from not leaving the S_0 state during the whole dynamics changes from $(p^\infty_0)^2$ to $(p^\infty_0)^2 + p_2p^\infty_0 + (1 - p_2)p_1p^\infty_0 = p^\infty_0$.
- The contribution from S_2 state that evolved for time t at $\epsilon = \epsilon_f$ changes from $(p^\infty_2)^2\overline{|a_2(t)|^2}$ to $[(p^\infty_2)^2 + (1 - p_2)p^\infty_2 + p_2p_1p^\infty_2]\overline{|a_1(t)|^2} = p^\infty_2\overline{|a_2(t)|^2}$.
- The contribution from S_1 state changes from $p^\infty_1\overline{|a_1(t)|^2}$ to $[p^\infty_1 + (1 - p_1)]\overline{|a_1(t)|^2} = p^\infty_1\overline{|a_1(t)|^2}$.

In this way $P_S(t)$ given by Eq. (21) is changed to the one given by Eq. (20) due to fast inelastic tunneling of the $S(3,1)$ state into the $S(4,0)$ state at the beginning of the PSB readout stage.

IV. OSCILLATIONS OF THE SINGLET RETURN PROBABILITY SIGNAL NEAR THE SPIN-VALLEY HOTSPOT

In absence of spin-valley mixing, the time-dependence of $P_{S,\mu\nu}(t)$ defined in Eq. (20) is only due to S-T_0 mixing

by the Zeeman splitting difference $\Delta E_Z^{\mu\nu}$. After averaging the result over a quasi-static distribution of spin splittings we obtain the well-known result [63],

$$P_{S,\mu\nu} = \frac{1}{2} + \frac{1}{2}\cos\left(\Delta E_Z^{\mu\nu} t\right)\exp\left(-\frac{1}{2}\sigma_{\mu\nu}^2 t^2\right) \ , \tag{22}$$

in which $\sigma_{\mu\nu}$ is the rms of distribution of $\Delta E_Z^{\mu\nu}$. We will now include the effects of spin-valley mixing on the $P_{S,\mu\nu}(t)$ signal when the Zeeman splitting in close to the valley splitting in one of the dots.

Depending on the kind of singlet $|S_{\mu\nu}\rangle$ and $\bar{E}_Z \approx E_V^L$ or E_V^R, we are thus dealing with one of 8 resonances from Table I. We will use the number of resonance $r = 1\ldots 8$ from this Table as an index identifying the relevant parameter, i.e. S_r will stand for respective $S_{\mu\nu}$, T_r will stand for the respective $T_{\alpha\beta}^p$, $\hat{H}_r$ will stand for the respective $\hat{H}_{\mu\nu,\alpha\beta}$ from Eq. (13), etc. The probability amplitude of the system initialized in $|S_r\rangle$ state to be in this state at time t is $a_r(t) = \langle S_r| e^{-i\hat{H}_r t} |S_r\rangle$, and the corresponding singlet return probability is $P_{S,r}(t) \equiv |a_r(t)|^2$.

Let us introduce the detuning from the relevant spin-valley anticrossing,

$$\delta_r = \frac{1}{2}\xi_r \Delta E_Z^r + p_r(E_V^r - \bar{E}_Z^r) \ , \tag{23}$$

and the parameter γ_r defined as

$$\gamma_r = \frac{1}{2}\xi_r \Delta E_Z^r - p_r(E_V^r - \bar{E}_Z^r) \ , \tag{24}$$

so that the part of Hamiltonian from Eq. (13) that is acting nontrivially in the relevant two-dimensional subspace is given by $\frac{1}{2}\mathbb{1}\gamma_r + v\hat{\sigma}_x + \frac{1}{2}\delta_r\hat{\sigma}_z$. We also introduce the mixing angle θ_r given by

$$\cos\theta_r \equiv \frac{\delta_r}{\sqrt{\delta_k^2 + 4|v_k|^2}} \ , \tag{25}$$

so that for $\delta_r/|v_r| \to \infty$ we have $\theta_r \to 0$, while on the other side of the anticrossing, for $\delta_r/|v_r| \to -\infty$ we have $\theta_r \to \pi$. .

Using the above we can derive

$$P_{S,r}(t) = C_r + \sum_{b=0,\pm} A_r^b \cos\omega_r^b \ . \tag{26}$$

where the constant part of the signal is C_r, and the A_r^b the amplitudes of the oscillating parts are

$$\begin{aligned} C_r &= \frac{1}{2} - \frac{1}{8}\sin^2\theta_r \ , \\ A_r^0 &= \frac{1}{8}\sin^2\theta_r \ , \\ A_r^\pm &= \frac{1}{4}(1 \pm \cos\theta_r) \ , \end{aligned} \tag{27}$$

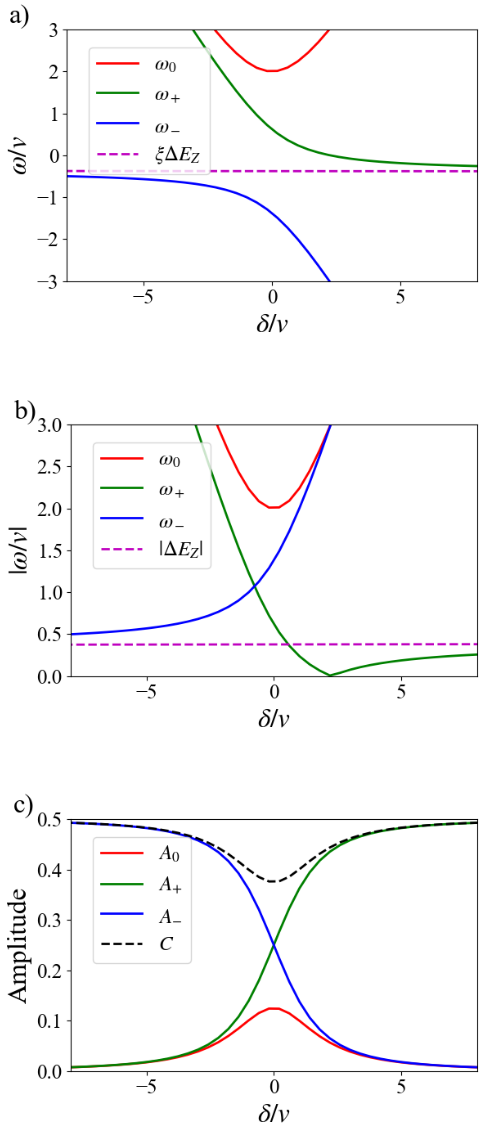

FIG. 4. a) Frequencies present in $P_S(t)$ signal (thick solid lines) in the vicinity of spin-valley resonance in one of the dots, given by Eqs. (28) and (29), and $\xi_r\Delta E_Z^r$ frequency (dashed line) that would be observed in absence of spin-valley coupling. The calculation is done for $\xi = 1$, $p = -1$, and $\Delta E_Z < 0$. The frequencies and detuning δ are expressed in units of v, the spin-valley coupling at a relevant resonance, making the features of the plots other than the position of the dashed no-spin-valley-coupling line universal. The used value of $|\Delta E_Z|$ at resonance corresponds to $-0.38v$. This is realized, for example, for resonance involving $|S_{eg}\rangle$ and electron in the left dot, when $E_V^L = 66.6$ μeV and $v = v_L = 58$ neV, which are the values measured for a DQD in [42]. For these values, the span of δ/v shown in the Figure corresponds to a span of 8 mT around an anticrossing at ≈ 570 mT. b) The same, but with absolute value of the frequencies plotted. c) The corresponding amplitudes from Eq. (27).

and the frequencies are

$$\omega_r^0 = \sqrt{\delta_r^2 + 4|v_r|^2}\ , \tag{28}$$

$$\omega_r^\pm = \frac{1}{2}(\xi_r \Delta E_Z^r + \gamma_r \pm \sqrt{\delta_r^2 + 4|v_r|^2}) = \frac{1}{2}(2\gamma_r + \delta_r \pm \sqrt{\delta_r^2 + |v_r|^2})\ . \tag{29}$$

Let us define $s \equiv \mathrm{sgn}(\delta_r)$. Far away from the hotspot, i.e. for $|\delta_r| \gg 2|v_r|$, after using the fact that $\gamma_r + \delta_r = \xi_r \Delta E_Z^r$, we see that only the oscillation with frequency $\omega_r^s \approx \xi_r \Delta E_Z^r$ has appreciable amplitude given by $A_r^s \approx 1/2$. To be precise, on the low (high) magnetic field side of the resonance we have $s = p_r$ ($s = -p_r$) and

$$\omega_r^s \approx \xi_r \Delta E_Z^r + s|v_r|^2/|\delta_r|\ , \tag{30}$$

while $A_r^s \approx \frac{1}{2} - \frac{1}{2}|v_r/\delta_r|^2$ and the other two oscillations have amplitudes are $\propto \frac{1}{2}|v_r/\delta_r|^2 \ll 1$. The result for no spin-valley mixing from Eq. (22) is thus recovered at sufficiently large detuning from the resonance.

The behavior of the "dominant" frequency as the relevant resonance is approached by changing the B field can be understood in the following way. For given r, depending on the value ξ_r, only one of the $|\uparrow\downarrow\rangle$ or $|\downarrow\uparrow\rangle$ levels is coupled to $|T_r\rangle$, see the discussion below Eq. (13). As the magnetic field is varied, the energy of the $|T_r\rangle$ state is approaching the range of energies of $|\uparrow\downarrow\rangle$ and $|\downarrow\uparrow\rangle$ states either from above, or below, depending on the sign of p_r. Consequently, the sign of perturbative renormalization of splitting between $|\uparrow\downarrow\rangle$ or $|\downarrow\uparrow\rangle$ levels has opposite signs on the low- and high-field sides of the resonance.

On the other hand, close to the resonance, when $|\delta_r| \ll 2|v_r|$, oscillations with three distinct frequencies are present in $P_{S,r}(t)$: we have then $A_r^0 \approx 1/8$ and $A_r^\pm \approx \frac{1}{4} \pm \delta_r/2|v_r|$ (and note that $C_a \approx 3/8$). The frequency of the oscillation that appears only close to resonance,

$$\omega_r^0 \approx 2|v_r| + \frac{\delta_r^2}{4|v_r|}\ , \tag{31}$$

is to first order independent of δ_r. On the other hand, the two "dominant" oscillations have $\omega_\pm$ given by

$$\omega_r^\pm \approx \frac{1}{2}(\xi_r \Delta E_Z^r + \gamma_r \pm 2|v_r| \pm \frac{\delta_r^2}{4|v_r|}) = \frac{3}{4}\xi_a \Delta E_Z^a + \frac{p_r}{2}(\bar{E}_Z^r - E_V^r) \pm |v_r| \pm \frac{\delta_r^2}{8|v|}\ , \tag{32}$$

Three things should be noted. The first is that $\omega_r^\pm$ vary rapidly with changing of the magnetic field: $|\partial\omega_r^\pm/\partial B| = \frac{1}{2}|\partial \bar{E}_Z^r/\partial B| \approx \mu_B$. This is half of the value of B derivative of the polarized triplet energy. (Note that these frequencies become fully "polarized triplet-like", i.e. $|\partial\omega_r^\pm/\partial B| \approx 2\mu_B$, only away from the resonance, when the corresponding amplitudes are very small.)

The second observation is the appearance of the term linearly proportional to the respective valley splitting, E_V^r, in expression (32). This means that while far away from the resonance the dephasing of the signal is due to fluctuations of the "bare" ΔE_Z^r caused by interaction with nuclei, or possibly electric-noise induced fluctuations of g-factors, close to the resonance the valley splitting fluctuations also contribute to the dephasing of the singlet return probability signal [45].

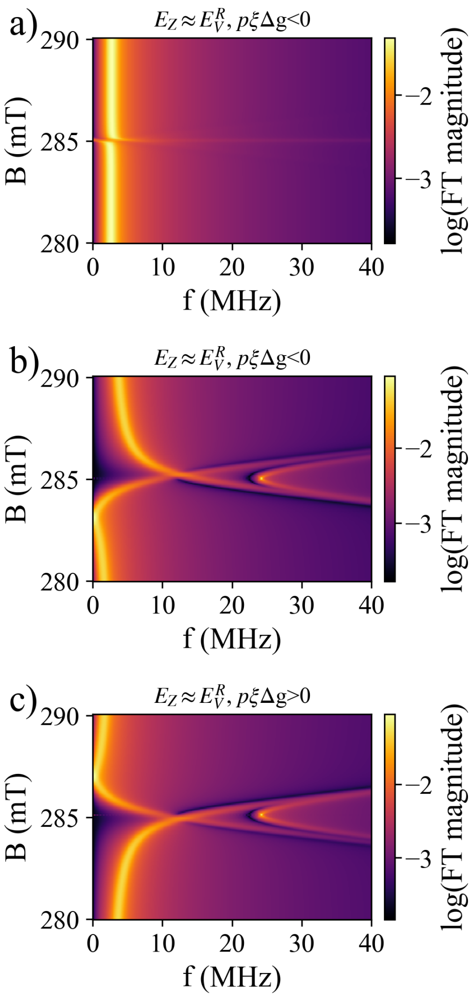

FIG. 5. Fourier transform of the singlet return probability near a spin-valley anticrossing. The calculation is done for B field in the vicinity of spin-valley hotspot in the R dot (resonances with odd r in Table I) for $E_V^R = 33$ μeV, $|\Delta g| = 6.58 \cdot 10^{-4}$, and dephasing parameters $\sigma_{ZR} = 1$ neV, $\sigma_{VR} = 10$ neV. Zero frequency components have been removed by subtracting the long-time value of $P_S(t)$ from the signals before Fourier-transforming them. (a) Weak coupling results for $v_D = 5$ neV, for which $|v_D| \ll \Delta E_Z$. (b,c) Two possible patterns of frequency and magnetic field dependence of Fourier transform of singlet return probability near a spin-valley anticrossing when $v_D = 50$ neV, so that $|v_D| \gg \Delta E_Z$. The result depends on the sign of $p_r \xi_r \Delta E_Z^r$ quantity. When calculations are done in the vicinity of spin-valley hotspot in the L dot, the two patterns are exchanged, i.e. the pattern from (c) appears when $p_r \xi_r \Delta E_Z^r < 0$ for even r.

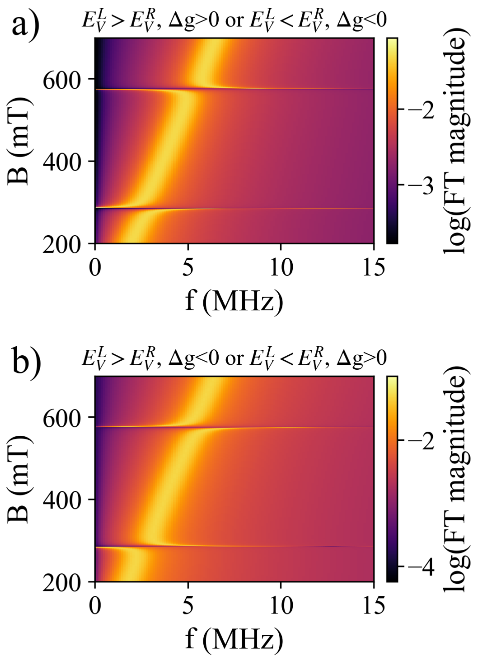

FIG. 6. Two possible patterns of frequency and magnetic field dependence of mu Fourier transform of singlet return probability that are realized *for any* of the $S_{\mu\nu}$ singlets from Fig. 1a depending on the signs of $\Delta g_{\mu\nu} = g_{\mu_L} - g_{\nu_R}$ and $E_V^L - E_V^R$.

The third thing to note is that exactly at resonance we have $\omega_r^\pm = \xi_r \Delta E_Z^r \pm |v_r|$. When $|v_r| \ll |\Delta E_Z^r|$, the renormalization of $\omega_r^\pm$ away from $\xi_r \Delta E_Z^r$ value is relatively small. On the other hand, when $|v_r| \gg |\Delta E_Z^r|$, the renormalization of oscillations having easily observable amplitudes is large, with the largest-amplitude oscillation having its frequency renormalized to zero on one side of the resonance, see Fig. 4(b). This regime of large relative renormalization of singlet-triplet precession frequency near a spin-valley resonance will be our main focus, as the recent measurement [42] of v_D and ΔE_Z at magnetic fields at which $\bar{E}_Z \approx E_V^D$ in Si/SiGe structures put us exactly in this regime.

Looking at a wide range of magnetic fields, the $\omega^\pm$ frequencies vary by orders of magnitude, see Fig. 4. However, when $|\omega_r^\pm| \gg \Delta E_Z^r$, the corresponding amplitude $A_r^\pm$ is very low, and such a high-frequency contribution to the total $P_S(t)$ signal is unobservable. The B field dependence of frequencies ω_r^b and amplitudes A_r^b is most transparently illustrated by plotting the Fourier tansform of Eq. (26) as a function of B field and frequency f. In Fig. 5a we show such f and B dependence of Fourier transform of $P_{S,r}(t)$ when the precession frequencies are weakly renormalized, $|v_r| \ll |\Delta E_Z^r|$. The dominant feature of the singlet return probability signal is a slight upward or downward (depending on being on the low/high B field side resonance) renormalization of the dominant frequency. On the other hand, when $|v_r| \gg |\Delta E_Z^r|$, we obtain more interesting patterns shown in panels (b) and (c): for $v_D \approx 50$ neV the dominant frequency is now qualitatively renormalized in a range of $\lesssim 10$ mT width around the resonance, and an oscillation with frequency $\omega^0 \approx 2|v_r|$ appears clearly in the signal for $|\delta| < 2|v_r|$. The frequency of high-amplitude component of $P_S(t)$ is renormalized to zero on low (high) B field side of the resonance for $\xi p \Delta g < 0$ (> 0), when $|\delta_r| \approx |v_r|^2/|\Delta E_Z^r|$, see Fig. 5b (5c).

Careful inspection of parameters of all the resonances given in Table I shows that the type of pattern is controlled by the sign of $p_r \xi_r \Delta E_Z^r$ product. Furthermore, when we look at the range of magnetic fields covering both the spin-valley resonances, the "global" pattern for any of the four (3,1) singlets looks like one of the two possibilities shown in Fig. 6, depending on the sign of ΔE_Z for the considered singlet and on whether we have $E_V^L > E_V^R$ or $E_V^L < E_V^R$.

V. DEPHASING OF THE SINGLET RETURN PROBABILITY OSCILLATIONS

For a fully realistic description of the singlet return probability signal we need to include the presence of quasi-static fluctuations of spin splittings in the two dots, as they are the dominant cause of dephasing of spin qubits when no effort, such a using dynamical decoupling, is made to remove the influence of low-frequency noise on them, see Eq. (22) for $P_S(t)$ away from anticrossings, or for negligible spin-valley coupling. We label the rms of Gaussian distribution of spin splitting in dot D as σ_{ZD}. The key physical source of these fluctuations in natural Si structures is hyperfine interaction with nuclei of ^{29}Si. In structures using isotopically enriched Si and without magnetic field gradients (in presence of which fluctuations of dot position due to electric field noise typically dominate the dephasing [1, 2]), hyperfine interaction with ^{73}Ge nuclei in the barrier material can be relevant [61], and QD motion due to electric field noise in presence of spatial dependence of electron g-factor (see [42] and [43] for examples of measured spatial dependence of g-factor for a shuttled Si/SiGe QD) could also contribute to σ_{ZD} at large enough magnetic fields.

As discussed in Sec. IV, near the spin-valley resonance r the frequencies present in the $P_{S,r}(t)$ signal depend also on the valley splitting E_V^D in quantum dot D in which the resonance occurs. We thus introduce in the model finite rms of Gaussian distribution of valley splitting in dot D, σ_{VD}. It is known that E_V depends on electric fields determining the overlap of the electron wavefunction with Si/SiGe interface and also on in-plane position of the dot [69] (for more discussion see Sec. VI B). Consequently, an always–present $1/f$ charge noise leads to quasi-static fluctuations of E_V^D.

The singlet return probability in each individual measurement is then calculated for Zeeman and valley splittings given by $E_Z^D + \delta E_Z^D$ and $E_V^D + \delta E_V^D$, and then an average over Gaussian distributions of all these parame-

ters is performed in order to obtain the $P_S(t)$ signal. We assume now that the fluctuations in all the parameters are independent. This should be a very good approximations when E_Z fluctuations are due to hyperfine interaction with the nuclear spins of ^{29}Si and ^{73}Ge, while E_V^D fluctuations are due to charge noise. We expand ω_r^b ($b=0,\pm$) frequencies from Eqs. (28) and (29) that are relevant near resonance r to linear order in the the fluctuating parameters, and after perfoming the Gaussian averages we obtain

$$P_{S,r}(t) = C_r + \sum_{b=0,\pm} A_r^b \cos\omega_r^b \times e^{-(t/T^*_{2,r,b})^2} , \quad (33)$$

which is the same as Eq. (26), only with Gaussian decay envelopes multiplying the respective oscillating factors. The dephasing times are given by $T^*_{2,r,b} \equiv \sqrt{2}/\sigma_{r,b}$, and the variance of fluctuations of ω_r^b frequencies are given by

$$\sigma^2_{r,0} = \frac{1}{4}\cos^2\theta_r \left[(\xi_r - p_r)^2\sigma^2_{ZL} + (\xi_r + p_r)^2\sigma^2_{ZR} + 4\sigma^2_{V,r}\right] , \quad (34)$$

$$\begin{aligned}\sigma^2_{r,\pm} = & \frac{1}{16}\left[\xi_r(3\pm\cos\theta_r) + p_r(1\mp\cos\theta_r)\right]^2\sigma^2_{ZL} + \\ & \frac{1}{16}\left[\xi_r(3\pm\cos\theta_r) - p_r(1\mp\cos\theta_r)\right]^2\sigma^2_{ZR} + \\ & \frac{1}{4}(1\mp\cos\theta_r)^2\sigma^2_{V,r} , \end{aligned} \quad (35)$$

where $\sigma^2_{V,r}$ is the variance of valley splitting in dot D, where D is taken from the rth row of Table I.

We can see that far away from the resonance, when $\cos\theta_r \approx \pm 1$, the dominant oscillation has frequency ΔE_Z^r and variance given by $\sigma_Z^2 \equiv \sigma^2_{ZL} + \sigma^2_{ZR}$, so it decays on timescale of $T_2^* = \sqrt{2}/\sigma_Z$, which is the well-known result for dephasing of singlet-triplet oscillation when spin-valley coupling is irrelevant [63], see Eq. (22). On the other hand, close to a resonance we have

$$\begin{aligned}\sigma^2_{r,\pm} \approx & \frac{10}{16}(\sigma^2_{ZL}+\sigma^2_{ZR}) + \frac{6}{16}\xi_r p_r(\sigma^2_{ZL}-\sigma^2_{ZR}) + \frac{\sigma^2_{V,r}}{4} + \\ & \pm\frac{\delta_r}{16|v_r|}(\xi_r - p_r)\sigma^2_{ZL} \pm \frac{\delta_r}{16|v_r|}(\xi_r + p_r)\sigma^2_{ZR} \pm \frac{\delta_r}{4|v_r|}\sigma^2_{V,r} ,\end{aligned} \quad (36)$$

where we have kept terms of first order in $\delta_r/|v_r|$. Inspection of Table I shows that for resonances involving spin-valley hotspot in L (R) dot, we have $\xi_r - p_r = 2\xi_r$ ($\xi_r - p_r = 0$), $\xi_r + p_r = 0$ ($\xi_r + p_r = 2\xi_r$), and $\xi_r p_r = -1$ ($\xi_r p_r = 1$), so for L dot resonances, i.e. even r, we have

$$\sigma^2_{\text{even } r,\pm} \approx \frac{1}{4}\sigma^2_{ZL} + \sigma^2_{ZR} + \frac{1}{4}\sigma^2_{VL} \pm \frac{\delta_r}{8|v_L|}(\xi_r\sigma^2_{ZL} + 2\sigma^2_{VL}) , \quad (37)$$

while for R dot resonances, i.e. odd r, we have

$$\sigma^2_{\text{odd } r,\pm} \approx \sigma^2_{ZL} + \frac{1}{4}\sigma^2_{ZR} + \frac{1}{4}\sigma^2_{VR} \pm \frac{\delta_r}{8|v_R|}(\xi_r\sigma^2_{ZR} + 2\sigma^2_{VR}) . \quad (38)$$

If $\sigma_{VD} \ll \sigma_{ZD}$, measurement of dephasing times of the signals oscillating with $\omega^\pm$ frequencies at the two resonances allows for fitting of both σ_{ZL} and σ_{ZR}. On the other hand, when $\sigma_{VD} \gg \sigma_{ZD}$, such measurement allow for fitting of values of both σ_{VL} and σ_{VR}.

Away from resonance, for $|\delta_r| \gg 2|v_r|$, starting from Eq. (35) we obtain that variance of the "dominant" frequency ω_r^s (where $s = \text{sgn}(\delta)$), which has amplitude $A_r^s \approx 1/2$, is given by

$$\sigma^2_{\text{even } r} \approx \sigma^2_{ZL} + \sigma^2_{ZR} - \frac{2|v_L|^2}{\delta_r^2}\sigma^2_{ZL} + \frac{|v_L|^4}{\delta_r^4}\sigma^2_{VL} , \quad (39)$$

$$\sigma^2_{\text{odd } r} \approx \sigma^2_{ZL} + \sigma^2_{ZR} - \frac{2|v_R|^2}{\delta_r^2}\sigma^2_{ZR} + \frac{|v_R|^4}{\delta_r^4}\sigma^2_{VR} . \quad (40)$$

We see that when $\sigma_{VD} \ll \sigma_{ZD}$, the dephasing time of the singlet return probability signal approaches the value unperturbed by spin-valley coupling very rapidly once detuning becomes larger than $2|v_r|$, i.e. once the mixing with the polarized triple is suppressed. From Eqs. (37)-(40) we see then that as long as σ_{ZL} and σ_{ZR} are of the same order of magnitude, the T_2^* of of the highest-amplitude component of the signal varies only slightly in a narrow range of B fields (of ≈ 2 mT width for $|v_r| \approx 50$ neV) around the resonance.

On the other hand, if $\sigma_{VD} \gg \sigma_{ZD}$, at resonance involving dot D we have $\sigma^2_{r,\pm} \approx \frac{1}{4}\sigma^2_{VD}$, It is important to note that far from the resonance, for $|\delta/2v| \ll 1$, from the above Equations we see that the dephasing is visibly affected by valley splitting fluctuations as long as $|\delta_r/v_D| < \sqrt{\sigma_{VD}/\sigma_Z}$. This means that for large enough σ_{VD} we can see strong effects of valley splitting fluctuations on dephasing time of $P_S(t)$ away from the near-resonance range of B fields, in which the signal consists of three oscillations with appreciable amplitude.

Finally, let us note that $\sigma^2_{r,0}$ goes to zero at resonance, as $\cos^2\theta_a \approx |\delta_r/2v_r|^2$ for $|\delta_r| \ll |v_r|$. Dephasing of this oscillation at resonance is then caused by second order terms in expansion of ω_0 with respect to fluctuations. In order to simplify the discussion of this slow dephasing at such a "sweet spot" with respect to noise, let us assume that valley splitting fluctuations are much larger than eeman splitting fluctuations splitting fluctuations, so that close to resonance we have $\omega_r^0 \approx 2|v_r| + \delta E^2_{V,r}/4|v_r|$. Instead of Gaussian decay, we obtain then an asymptotic power-law decay well known from calculations of dephasing due to quadratic influence of quasistatic noise [70–73]:

$$\overline{\cos\omega_r^0 t} = \frac{\cos\left(2|v_r|t + \frac{1}{2}\arctan t/\tau\right)}{(1+t^2/\tau^2)^{1/4}} , \quad (41)$$

from which we see that the half-decay time of envelope of oscillation is approximately $4\tau = 8|v_r|/\sigma^2_{V,r}$, and asymptotic decay is $\propto 1/\sqrt{t}$. As $8|v_r|/\sigma_{V,r}$ is typically large (see Section VI B for discussion involving realistic parameters of Si/SiGe quantum dots), this half-decay time is much larger than $T^*_{2,r,\pm} \approx 2\sqrt{2}/\sigma_{V,r}$ of the remaining oscillations close to the resonance. We can thus approximately

treat the oscillation with $\omega_r^0 \approx 2|v_r|$ frequency as nondecaying compared to the other two oscillations present in the signal close to the resonance.

An example dependence of the $P_S(t)$ on magnetic field in vicinity of a spin-valley resonance is shown in Fig. 7, where results of calculations using the above formulas for seven values of B field in 10 mT wide range of fields around the resonance at $B_r \approx 285$ mT, corresponding to $E_{VR} = 33$ μeV. The calculations are performed with the parameters used previously in Fig. 5c for resonance in the R dot, with $v_R = 50$ neV and $\sigma_{VR} = 10$ neV, while $\sigma_{ZL} = \sigma_{ZR} = 1$ neV. For $|B - B_r| \approx 5$ mT we see oscillations decaying with $T_2^* \approx \hbar/\sigma_{ZD}$, but with visibly distinct frequencies due to spin-valley mixing still having a non-negligible influence. In Fig. 7a, at $B = 287.11$ mT we have a result (green line) when the dominant frequency is approximately zero due to strong spin-valley induced renormalization. The quickly decaying low-amplitude component of this signal is due to a subdominant contribution that dephases on $\approx \hbar/\sigma_{VR}$ timescale. At $B = B_r$ (dashed blue line) we see fast dephasing of two dominant frequencies, with the sum of the two contributions to the signal exhibiting a beating pattern due to distinct values of these $\omega_r^\pm$, while for $t > 200$ ns we see only the oscillation with $\omega_r^0 = 2|v_R|$ frequency that does not exhibit any decay on $\sim$ microsecond timescale. In Fig. 7b we see that after lowering the field by a fraction of a mT from B_r (the red line), the oscillation with $\omega_r^0 = 2|v_R|$ frequency practically disappears due to reactivation of dephasing away from the "sweet spot", while the oscillations with $\omega_r^\pm$ are still undergoing dephasing in about 200 ns. As B field is moved further away from the resonance (green and blue lines), the decay time of these oscillations grows towards the far-off resonance value of $T_2^* = 658$ ns.

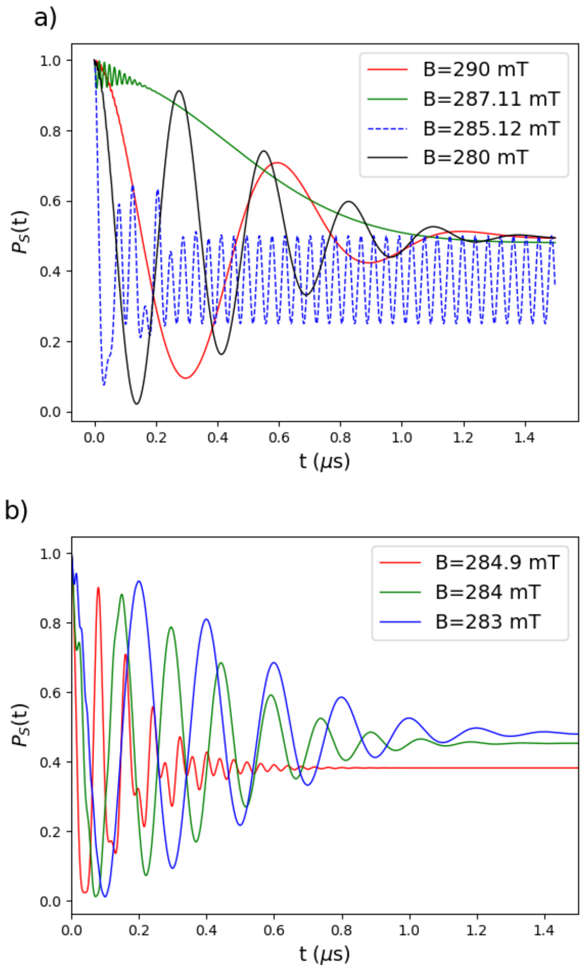

FIG. 7. $P_S(t)$ signals calculated for resonance $r = 1$ from Table I calculated for parameters used in Fig. 5c: $E_V^R = 33$ μeV, $v_R = 50$ neV, $\Delta g = 6.58 \cdot 10^{-4}$, $\sigma_{ZL} = \sigma_{ZR} = 1$ neV (corresponding to $T_2^* = 658$ for B fields far from resonance), and $\sigma_{VR} = 10$ neV. In a) we show the results illustrating mostly the B-dependence of observed frequencies of oscillations: for $B = 290$ mT and 280 mT we see single oscillation (with frequency close to the unperturbed one given by $\Delta g \mu_B B/h \approx 2.6$ MHz in $B = 280$ mT case), which decay due to fluctuations of spin splitting. At $B = 287.11$ mT the frequency of the oscillation with the largest amplitude is very close to zero, while at $B = 285.12$ mT we are exactly at the resonance: the two oscillations with larger amplitude exhibit beating and decay in < 300 ns, and at longer times we see the non-decaying oscillation with frequency given by $2v_R/h$. In b) we show the evolution of the signal as the B field is decreased from the resonance value: the red line, for B field that differs from the resonance value by ≈ 0.2 mT should be compared with the blue dashed line in (a).

VI. COMPARISON WITH EXPERIMENTS IN SI/SIGE DOUBLE QUANTUM DOTS

A. Experiments on double quantum dots in two distinct Si/SiGe structures

Charge separation from $(4,0)$ state resulting in initialization of $(3,1)$ spin singlet in a DQD, subsequent shuttling of the R dot to location at distance d from the initial one, followed by shuttling back to the tunnel-coupled DQD arrangement and Pauli spin blockade readout of singlet occupation, was studied in recent qubit shuttling experiments undoped Si/SiGe structures. In [21, 42] a structure having natural abundance of ^{29}Si isotope was investigated, while in [43, 46] a structure containing isotopically enriched silicon with < 800 ppm of ^{29}Si was studied. The structures came from completely distinct growth protocols, for details see [21] and [46], respectively.

We focus here on the experimental protocol in which the evolution of charge-separated singlet occurs predominantly in the static two-dot configuration, with dot L in its original place, and dot R kept at distance d from its original position for time t. This includes the case of $d = 0$, i.e. of simply waiting in $(3,1)$ charge configuration for time t and performing the PSB readout of singlet occupation. The experimental data presented in this Section corresponds to this case of no back-and forth

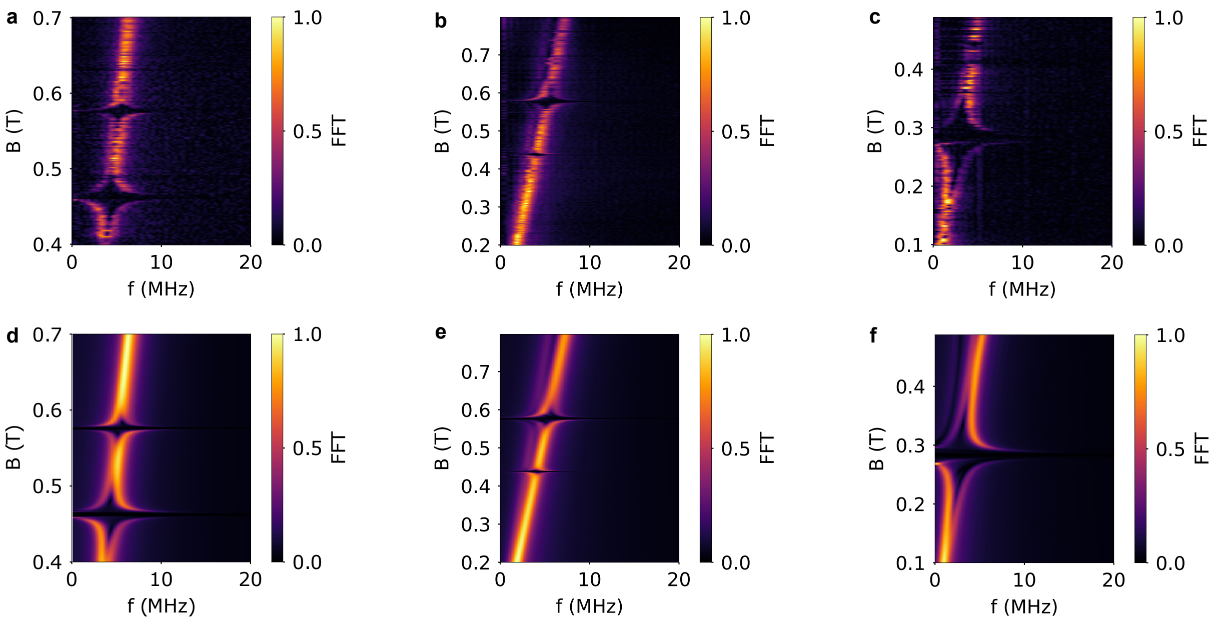

FIG. 8. Upper row: Fourier transform of singlet-return probability measured in a range of magnetic fields in structure from [21, 42] for three positions of the DQD along the y axis. Lower row: theoretical fit to the data using parameters from Table II.

shuttling of the R dot. However, the previously described theory for singlet return probability signal applies also to the experiments with back-and-forth shuttling, provided that (1) shuttling to distance d and back does not perturb the spin and valley state of the shuttled electron, and (2) the evolution in the relevant singlet-triplet subspaces during the motion of the R dot is either negligible, or it has been accounted for by correcting the signal for the singlet-triplet oscillation that occurred during the motion of the R dot. Let us however note that making sure that assumption (1) holds requires caution. Simply shuttling as fast as possible with high-frequency oscillation of voltages on clavier gates [12] in a conveyor belt setup can lead to changes of the valley state of the shuttled electron when conditions for adiabaticity of dynamics are broken at local minima of valley splitting [12, 46].

First, let us discuss three sets of data for DQD with static R dot acquired during the experiments described in [42]. These sets were obtained for distinct values of screening gate voltages that resulted in shifting of the DQD position in the y direction (with the line connecting L and R dot efining the x axis). With the dataset 1 (D1) corresponding to a DQD at $y = 0$, the datasets 2 and 3 correspond to the DQDs at $y = -6$ and 6 nm, respectively. The Fourier transform of the singlet return probability as function of frequency $f = \omega/2\pi$ and B field is plotted for the three settings of screening voltages in Fig. 8(a,b,c). The frequency in $\tilde{P_S}(f, B)$ signal that is dominant in the whole range of B fields was identified in [42] for datasets shown in ig. 8(a,b). For both, the B dependence of this frequency was following the pattern from Fig. 6(b). Evolution of the observed pattern after shuttling of electron in R dot allowed for identification of the resonance associated with the static dot, and consequently establishing that $E_V^L > E_V^R$ in the initial DQD. Using the definitions of ΔE_Z and Δg used here, from the qualitative shape of the B dependence of the dominant frequency in the signal we arrive at the conclusion that $\Delta g < 0$ for the singlet contributing the largest-amplitude part of the signal (note that Δg was defined in [42] with an opposite sign, and positive value of Δg given there is consistent with the discussion here). The identification of the sign of ΔE_Z was made in [42] assuming that $|S_{eg}\rangle$ singlet was initialized. However, the analysis presented in Section IV shows that this identification remains correct even if any other singlet was initialized.

For data shown in Fig. 8c only one resonance is visible. Based on evolution of the pattern upon shuttling of R dot we assign this resonance to this dot, and conclude that E_V^L is either so large that the L dot resonance is not visible in the measured range of fields (which was larger that the one in Fig. 8c, where we present a zoom on the single resonance), or v_L was so small that the resonance could not be discerned. If $E_V^L > E_V^R$, we also have $\Delta g < 0$ also in this dataset.

Let us focus now on the features of the data that were not given attention in [42]. A more careful look at the data in Fig. 8(a,b,c) shows that the observed patterns are in fact *sums of the two patterns from Fig. 6*, although the pattern from Fig. 6a has an amplitude smaller than the pattern from Fig. 6b by a factor of about 2. This is an unambiguous signature of the fact that *at least two* $|S_{\mu\nu}\rangle$ *singlets are initialized* by sweeping the detuning from $(4, 0)$ to $(3, 1)$ charge regime. As noted previously in Sec. III, the presence of two components in the signal is not surprising, as it had been seen in a previous exper-

	D1 ($y=0$)	D2 ($y=-6$ nm)	D3 ($y=6$ nm)	D4 (enriched Si sample)
E_V^L (μeV)	66.7 ± 0.07	66.7 ± 0.05	–	112.1 ± 0.007
E_V^R (μeV)	53.6 ± 0.07	50.7 ± 0.04	32.7 ± 0.06	95.53 ± 0.006
v_L (neV)	57.2 ± 0.2	68.5 ± 0.05	–	154.1 ± 0.05
v_R (neV)	90.5 ± 0.2	33.5 ± 0.06	136 ± 0.3	43.96 ± 0.07
Δg_1 (10^{-4})	$\mathbf{-6.40 \pm 0.04}$	$\mathbf{-6.41 \pm 0.02}$	$\mathbf{-7.15 \pm 0.1}$	$\mathbf{15.88 \pm 0.02}$
Δg_2 (10^{-4})	$\mathbf{6.76 \pm 0.08}$	$\mathbf{6.68 \pm 0.03}$	$\mathbf{6.9 \pm 0.2}$	$\mathbf{-15.6 \pm 0.1}$
Δg_3 (10^{-4})	-7.03 ± 0.07	5.22 ± 0.03	4.4 ± 1	-33.93 ± 0.08
Δg_4 (10^{-4})	7.39 ± 0.07	-4.95 ± 0.03	-9.6 ± 0.9	34.21 ± 0.09
p_1 (10^{-2})	$\mathbf{14.1 \pm 0.3}$	$\mathbf{14.92 \pm 0.03}$	$\mathbf{12.2 \pm 0.1}$	$\mathbf{7.83 \pm 0.04}$
p_2 (10^{-2})	$\mathbf{8.0 \pm 0.3}$	$\mathbf{7.27 \pm 0.03}$	$\mathbf{4.6 \pm 0.1}$	$\mathbf{0.88 \pm 0.02}$
p_3 (10^{-2})	4.04 ± 0.3	3.11 ± 0.03	1.5 ± 0.1	$(-4.58 \pm 2) \cdot 10^{-2}$
p_4 (10^{-2})	2.4 ± 0.3	2.38 ± 0.03	-0.1 ± 0.9	$(-4.47 \pm 2) \cdot 10^{-2}$
σ_Z (neV)	1.26 ± 0.20	1.395 ± 0.055	1.17 ± 0.19	0.48 ± 0.72
σ_{VL} (neV)	5.72 ± 0.37	19.16 ± 0.13	–	30.72 ± 0.27
σ_{VR} (neV)	15.50 ± 0.43	3.402 ± 0.075	9.15 ± 0.38	3.281 ± 0.065

TABLE II. Parameters obtained by fitting the $P_S(t)$ signals the Fourier transforms of which are shown in Figs. 8(a,b,c) (respectively D1,D2,D3) and Fig. 9(a) (D4). E_V^D are valley splittings in dot $D = L(R)$, v_D are the spin-valley couplings in dot D, Δg_k are the values of g-factor difference for up to four $(3,1)$ singlets that contribute to the signal, and p_k are the amplitudes of the signals corresponding to these singlets. Note that negative p_k means that the oscillation is phase-shifted by $\sim \pi$ with respect to the dominant part of the signal. σ_Z is the fitted rms of spin splitting difference, and σ_{VD} is the rms of valley splitting in dot D. For each dataset we have marked in bold the amplitudes of the two largest components of the signal, and the corresponding Δg.

iment on the same sample [21] involving shuttling the R dot for distance d and immediately back. However, it is striking that the two strongest components in the measured signals correspond to very similar values of $|\Delta g|$: the two dominant parts of the signal that are clearly visible at resonances merge into a single line away from the resonances, showing that the values of $|\Delta E_Z|$ for the two (or more) singlets are approximately equal to each other, with accuracy given by the typical linewidth of in Fig. 8, corresponding to $T_2^* \sim 1$ μs away from the resonances. Only in the dataset shown in Fig. 8(b) we can see a third component of the signal(further weaker than the second one), which corresponds to $|\Delta g|$ about 25% smaller than the value for the dominant components.

In order to extract quantitative information from all the data we have performed global fits to the *time-domain* results for singlet-return probability, allowing for finite occupations p_k of four singlets, $(k = 1 \dots 4)$ with corresponding values of g-factor difference Δg_k. For each dataset, we use the the theoretical model described above that contains 15 parameters: E_V^D, v_D, p_k, Δg_k, σ_Z, and σ_{VD}. In the first round of fitting, we have concentrated on obtaining the values of parameters other than σ_{ZD} and σ_{VD}, by using a simplified phenomenological model in which T_2^* decay of all the contributions to $P_S(t)$ is allowed to vary in the vicinity of the resonances. Values of valley splitting and spin-valley couplings obtained from this fit are then used (as fixed) in the second round of fitting, in which we fit again p_k, Δ_k, and also σ_Z and σ_{VD}, while excluding the data very near the resonances (precisely for detuning $|\delta| \leq |v_D|$). The reason for this is twofold. Firstly, the presence of two oscillations with amplitudes of the same order of magnitude, but distinct frequencies, near the anticrossings due to opposite signs of respective Δg, leads to destructive interference effects in $P_S(t)$ signal that are hard to distinguish from effect of suppression of T_2^* due to influence of valley splitting fluctuations. Secondly, a bit away from resonance, for $|\delta > |v_D|$, we can use Eq. (39) or (40), respectively, depending on the dot in which the closest resonance occurs, to obtain a simple analytical model for B field dependence of decay time of all the components of the signal. The results of the whole procedure are given in Table II. Fourier transforms of $P_S(B;t)$ data calculated using these parameters are shown in the lower row of Fig. 8.

The following features of the fitted sets of parameters are worth noting. For the three datasets, the ratios of maximal p_1 to the next largest p_2 are given by 1.75, 2, and 2.6, respectively, while the relative differences of fitted $|\Delta g_1|$ and $|\Delta g_2|$ for the two components are between 3% and 6%. As one can see in the Figure, only in the second dataset there is a reliable signature of the third component (in fact a pair components with $p_3 \approx p_4$ equal to about 0.3 of p_2) with $|\Delta g_3| \approx |\Delta g_4|$ being about 25% smaller than $|\Delta g_{1(2)}|$. Note that components with finite p_3 and p_4 in dataset 1 are arguably spurious, and they simply contribute to additional line broadening of the signals associated with p_1 and p_2 amplitudes. Interestingly, the obtained magnitudes valley splitting fluctuations are larger by about an order of magnitude than the Zeeman splitting fluctuations, and they exhibit visible dot-dependence.

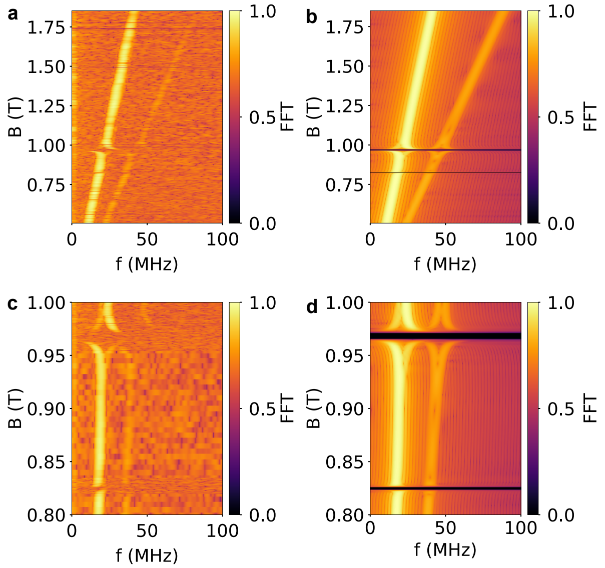

FIG. 9. Fourier transform of singlet return probability as function of frequency f and magnetic field B: (a) experiment in isotopically enriched Si/SiGe double quantum dot and (b) theoretical fit with parameters given in the last column of Table II. (c) and (d) are respective zoomed plots focused on the two visible resonances. Note that the color scale scale in this Figure is logarithmic.

Let us stress that if the values of dot (D) and valley (ν) dependent g-factors, g_{ν_D}, were independent random variables, a generic pattern arising from presence of two singlets should exhibit two discernible frequencies across the investigated range of magnetic fields, corresponding to distinct values of $|\Delta g_{\mu\nu}|$ and $|\Delta g_{\mu'\nu'}|$ for the $|S_{\mu\nu}\rangle$ and $|S_{\mu'\nu'}\rangle$ singlets present in the initialized mixed state. The results from Fig. 8(a,b,c) suggest a presence of nontrivial symmetry in the distribution of g-factors in the measured structure: the values of Δg_1 and Δg_2 extracted for the two highest-amplitude components of the signal have opposite signs, but nearly equal absolute values.

This hypothesis that such a symmetry is a general feature of Si/SiGe quantum dots is strengthened by an observation of analogous pattern of frequencies in DQD measurement in a distinct structure involving isotopically enriched Si used in shuttling experiments reported in [46], see Fig. 9. Note that in this Figure the color scale is logarithmic, allowing one to see the presence of two distinct frequencies away from resonances, with one of the components having its amplitude larger by ~ 100 than the other. At the first sight one could conclude that this is precisely the above-mentioned "generic" pattern corresponding to a mixture of two initialized singlets, each having a random value of $\Delta g_{\mu\nu}$. However, a closer inspection of the results zoomed on the resonances strongly suggests the presence of *two pairs of singlets, with opposite signs but equal moduli of* Δg *within pair.* The signal there can be indeed fitted by a sum of four contributions, a dominant one corresponding to $\Delta g \approx 15.9$, the subdominant one (with amplitude smaller by a factor of ~ 10) with $\Delta g \approx -15.6$, and two much weaker components (with amplitudes smaller by one more order of magnitude) with $\Delta g \approx \pm 34$, see Table II. Note that the values of Δg in this sample are larger by a factor of at least 2 that in the previously discussed one (this is confirmed by comparison of measurements of g-factor maps spanning an area much larger than the sizes of the quantum dots in this sample [43] and in the one studied in [42]). The ratio $p_1/p_2 \approx 10$ is much larger that in the other structure, and as noted before $p_1/p_{3(4)} \approx 100$. This shows that the effects of non-adiabaticity during charge separation are weaker in this structure, suggesting larger values of $t_{\mu\nu}$ - although the presence of 4 components in the fit to the signal shows that all the valley occupations of $(3,1)$ singlets are still initialized with finite probability.

B. Dephasing seen in experiments

Let us discuss now the values of standard deviations of fluctuations of spin and valley splitting. The value of σ_Z in this isotopically enriched sample is smaller by at least a factor of 2 than in the sample with natural silicon. Qualitatively this is expected. Quantitative discussion of the obtained values is left for future research.

Valley splitting fluctuations are caused by electric field noise: fluctuations in z component of electric field modify the overlap of the electron wavefunction with Si/SiGe interface, and fluctuations of the in-plane components of the field shift the position of the dot. Both effects should lead to changes of valley splitting for a given quantum dot. We focus here on the latter effect, as the results from Table I, and the valley splitting mappings results from [42] provide us with estimates of derivatives of E_V with respect to in-plane coordinates. Traces of $E_V^R(x)$ from [42] imply $|\mathrm{d}E_V^R/\mathrm{d}x| \approx 0.5$ μeV/nm for DQDs from Fig. 8. On the other hand, from values given in Table II, recalling that DQDs in D2 and D3 are shifted by about ± 6 nm along y axis with respect to the DQD corresponding to D1 set of results, we get two values of $|\mathrm{d}E_V^R/\mathrm{d}x| \approx 0.5$ μeV/nm and ≈ 3 μeV/nm. We can then estimate the order-of-magnitude of in-plane fluctuations of QD position from experiments on spin decoherence caused by electric field noise in presence of magnetic field gradient. From [2] one can extract the standard deviation of QD position due to $1/f$ charge noise (with data acquisition time of about 10 minutes, which is similar to the time used for each trace in the discussed experiments) of $\sigma_x \approx 5 \cdot 10^{-3}$ nm. A similar value can be inferred from levels of in-plane electric field noise measured in [74]. Using this value of σ_x, we arrive at $\sigma_V \approx 5$ neV for $|\mathrm{d}E_V^D/\mathrm{d}x| = 1$ μeV/nm. Taking into account that we have used the estimates of σ_x from other experiments on distinct Si/SiGe structures, so that it should be treated as an order-of-magnitude estimate, we can thus state that values of σ_V of a few neV are expected, and $\sigma_V \sim 10$ neV is consistent with mechanism of charge noise induced in-

plane motion in presence of measured spatial dependence of valley splitting.

C. Implications for valley-dependence of g-factors

These observations concerning the pattern of g-factor differences in the measured DQDs are in fact naturally explainable by the recently developed theoretical model of valley dependence of g-factors in Si/SiGe quantum dots [51, 52], which predicts

$$g_{\nu_D} \approx g_0 \pm \delta g_0 \cos\phi_D \equiv g_0 \pm \delta g_D \ , \tag{42}$$

in which $+$ ($-$) sign corresponds to $\nu = e$ (g) valley state, g_0 and δg_0 are weakly dependent on dot position and shape (assuming that the dot shape does not change drastically when it is moved from one place to another in a given heterostructure), and ϕ_D is the phase of the valley coupling matrix element for electron in dot D, $\Delta_D \equiv |\Delta_D| e^{i\phi_D}$ [31]. According to recent theoretical works [29–31, 55] discussing the influence of alloy disorder on the value of $\Delta(\mathbf{r})$ for quantum dot centered at position $\mathbf{r}$, real and imaginary components of $\mathbf{r}$ are Gaussian random fields with correlation length of the order of quantum dot size.

The opposite signs of valley-dependent correction to g_0 for the two valleys immediately give us that for singlets created by intra-valley (i.e. valley eigenstate conserving) tunneling, $|S_{gg}\rangle$ and $|S_{ee}\rangle$, we have

$$\Delta E_Z^{ee} = \delta g_L - \delta g_R = -\Delta E_Z^{gg} \ , \tag{43}$$

and for the singlets created by inter-valley tunneling we obtain an analogous relationship between the g-factor differences

$$\Delta E_Z^{eg} = \delta g_L + \delta g_R = -\Delta E_Z^{ge} \ . \tag{44}$$

If the inter-dot tunneling is predominantly of the valley-conserving character, i.e. $|t_{ee}| = |t_{gg}| \gg |t_{eg}| = |t_{ge}|$, see Eq. (4), so that S_{ee} and S_{gg} singlets have a chance to be created by adiabatic passage, with both p_{ee} and p_{gg} both non-negligible, while the tunnel anticrossings involving S_{eg} and S_{ge} singlets are passed almost diabatically due to smallness of the valley-flip tunneling, we expect the $P_S(t)$ signal to contain two components corresponding to very similar values of Δg, but opposite signs - just as in the results shown in Figs. 8 and 9. The same is expected to occur when the valley-flip tunneling is much stronger than the valley-conserving tunneling.

All the experimental results discussed above can be explained in a natural way using this model. The results for DQDs with non-shuttled R dot in structure from [42] correspond to either $|\delta g_L - \delta g_R|$ or $|\delta g_L + \delta g_R|$ being equal to approximately $7 \cdot 10^{-4}$, with little variation in Δg exhibited when the DQD is moved in the y direction by a distance of 6 nm. The appearance of component with distinct $|\Delta g| \approx 5 \cdot 10^{-4}$ in the second dataset can be then explained by assuming that the value of $|\delta g|$ in one dot is much larger than in the other: precisely, assumption of $|\delta g| \approx 5.75 \cdot 10^{-4}$ in one dot, and $|\delta g| \approx 0.75 \cdot 10^{-4}$ in the other, reproduces the observed set of Δg values. On the other hand, if we approximate the fit to the fourth dataset by $|\Delta g_{1(2)}| \approx 16$ and $|\Delta g_{1(2)}| \approx 34$, we arrive at $|\delta g| \approx 24$ in one dot, and $|\delta g| \approx 9$ in the other. Note that depending on assignment of ee/gg and eg/ge valley character to each pair of singlets, and on the choice of assignment of signs of Δg within each pair, any of 16 possible assigments of $(\pm 9, \pm 25)$ to $(\delta g_L, \delta g_R)$ can be realized.

VII. SUMMARY

We have presented a theoretical model of initialization, dynamics, and readout of $(3,1)$ singlets in silicon double quantum dots in which spin-valley coupling is non-negligible. One purpose of this paper is to give a detailed account of the influence of spin-valley coupling on dynamics of these singlets in order to give a full picture of the effect of large spin-valley coupling induced renormalization of frequency of singlet return probability signal. The presence of this renormalization was used in [42], and more recently in [43], to perform the mapping of valley splitting by shuttling one of the dots from the standard DQD setup (the two dots ≤ 100 nm away, with tunnel coupling between them controlled by the barrier gate) by up to a few hundreds of nanometers from its initial position. Comparison of the predictions of the model with the experiments conducted on two distinct Si/SiGe structures has also shown the following. (1) charge separation from $(4,0)$ to $(3,1)$ cannot be assumed to be perfectly adiabatic, and initialization of statistical mixture of $(3,1)$ singlets with distinct valley occupation pattern occured in both experiments. (2) While creation of mixture of valley occupations was not intended, careful analysis of the singlet return probability signals near the spin-valley resonances has uncovered an interesting symmetry of the g-factors in the studied dots. These results give support to the recently proposed theory of valley-dependence of g-factors in Si/SiGe quantum dots [51, 52] (note that the same symmetry was predicted for SiMOS dots with large valley splittings in [60]). (3) Valley splitting fluctuations were predicted to contribute to dephasing of singlet-triplet oscillations in the vicinity of the spin-valley resonances, and to dominate this dephasing if these fluctuations are larger than the spin splitting fluctuations (due to nuclear noise of g-factor variations caused by electric field noise). Fits of the model to the data from two distinct structures have given strong hints that the dephasing time indeed decreases by at least an order of magnitude in the vicinity of the resonances.

ACKNOWLEDGMENTS

This project was supported by .... and by the European Union's Horizon 2020 research and innovation programme under grant agreement 101174557 (project QLSI^2).

---

[1] J. Yoneda, K. Takeda, T. Otsuka, T. Nakajima, M. R. Delbecq, G. Allison, T. Honda, T. Kodera, S. Oda, Y. Hoshi, N. Usami, K. M. Itoh, and S. Tarucha, A quantum-dot spin qubit with coherence limited by charge noise and fidelity higher than 99.9%, Nature Nanotechnology **13**, 102 (2018).

[2] T. Struck, A. Hollmann, F. Schauer, O. Fedorets, A. Schmidbauer, K. Sawano, H. Riemann, N. V. Abrosimov, Ł. Cywiński, D. Bougeard, and L. R. Schreiber, Low-frequency spin qubit detuning noise in highly purified ^{28}si/sige, npj Quantum Inf. **6**, 40 (2020).

[3] T. Struck, J. Lindner, A. Hollmann, F. Schauer, A. Schmidbauer, D. Bougeard, and L. R. Schreiber, Robust and fast post-processing of single-shot spin qubit detection events with a neural network, Sci. Rep. **11**, 16203 (2021).

[4] A. Noiri, K. Takeda, T. Nakajima, T. Kobayashi, A. Sammak, G. Scappucci, and S. Tarucha, Fast universal quantum gate above the fault-tolerance threshold in silicon, Nature **601**, 338 (2022).

[5] A. R. Mills, C. R. Guinn, M. J. Gullans, A. J. Sigillito, M. M. Feldman, E. Nielsen, and J. R. Petta, Two-qubit silicon quantum processor with operation fidelity exceeding 99%, Sci. Adv. **8**, eabn5130 (2022).

[6] X. Xue, M. Russ, N. Samkharadze, B. Undseth, A. Sammak, G. Scappucci, and L. M. K. Vandersypen, Quantum logic with spin qubits crossing the surface code threshold, Nature **601**, 343 (2022).

[7] S. G. J. Philips, M. T. Madzik, S. V. Amitonov, S. L. de Snoo, M. Russ, N. Kalhor, C. Volk, W. I. L. Lawrie, D. Brousse, L. Tryputen, B. P. Wuetz, A. Sammak, M. Veldhorst, G. Scappucci, and L. M. K. Vandersypen, Universal control of a six-qubit quantum processor in silicon, Nature **609**, 919 (2022).

[8] I. F. de Fuentes, E. Raymenants, B. Undseth, O. Pietx-Casas, S. Philips, M. Madzik, S. L. de Snoo, S. V. Amitonov, L. Tryputen, A. T. Schmitz, A. Y. Matsuura, G. Scappucci, and L. M. K. Vandersypen, Running a six-qubit quantum circuit on a silicon spin qubit array, arXiv:2505.19200 10.48550/arXiv.2505.19200 (2024).

[9] Y. Fang, P. Philippopoulos, D. Culcer, W. A. Coish, and S. Chesi, Recent advances in hole-spin qubits, Mat. Quant. Tech **3**, 012003 (2023).

[10] F. Borjans, X. G. Croot, X. Mi, M. J. Gullans, and J. R. Petta, Strong Spin-Photon Coupling in Silicon, Nature **577**, 195 (2020).

[11] N. Samkharadze, G. Zheng, N. Kalhor, D. Brousse, A. Sammak, U. C. Mendes, A. Blais, G. Scappucci, and L. M. K. Vandersypen, Resonant microwave-mediated interactions between distant electron spins, Science **359**, 1123 (2018).

[12] V. Langrock, J. A. Krzywda, N. Focke, I. Seidler, L. R. Schreiber, and Ł. Cywiński, Blueprint of a scalable spin qubit shuttle device for coherent mid-range qubit transfer in disordered si/sige/sio$_2$, PRX Quantum **4**, 020305 (2023).

[13] J. M. Boter, J. P. Dehollain, J. P. van Dijk, Y. Xu, T. Hensgens, R. Versluis, H. W. Naus, J. S. Clarke, M. Veldhorst, F. Sebastiano, and L. M. K. Vandersypen, Spiderweb array: A sparse spin-qubit array, Phys. Rev. Appl. **18**, 024053 (2022).

[14] F. Ginzel, M. Fellner, C. Ertler, L. R. Schreiber, H. Bluhm, and W. Lechner, Scalable parity architecture with a shuttling-based spin qubit processor, Phys. Rev. B **110**, 075302 (2024).

[15] M. Künne, A. Willmes, M. Oberländer, C. Gorjaew, J. D. Teske, H. Bhardwaj, M. Beer, E. Kammerloher, R. Otten, I. Seidler, R. Xue, L. R. Schreiber, and H. Bluhm, The spinbus architecture for scaling spin qubits with electron shuttling, Nat. Commun. **102**, 195418 (2023).

[16] I. Seidler, T. Struck, R. Xue, N. Focke, S. Trellenkamp, H. Bluhm, and L. R. Schreiber, Conveyor-mode single-electron shuttling in Si/SiGe for a scalable quantum computing architecture, npj Quantum Information **8**, 100 (2022).

[17] A. Zwerver, S. Amitonov, S. de Snoo, M. Madzik, M. Rimbach-Russ, A. Sammak, G. Scappucci, and L. Vandersypen, Shuttling an electron spin through a silicon quantum dot array, PRX Quantum **4**, 030303 (2023).

[18] R. Xue, M. Beer, I. Seidler, S. Humpohl, J.-S. Tu, S. Trellenkamp, T. Struck, H. Bluhm, and L. R. Schreiber, Si/sige qubus for single electron information-processing devices with memory and micron-scale connectivity function, Nat. Comm. **15**, 2296 (2024).

[19] J. Yoneda, W. Huang, M. Feng, C. H. Yang, K. W. Chan, T. Tanttu, W. Gilbert, R. C. C. Leon, F. E. Hudson, K. M. Itoh, A. Morello, S. D. Bartlett, A. Laucht, A. Saraiva, and A. S. Dzurak, Coherent spin qubit transport in silicon, Nat. Commun. **12**, 4114 (2021).

[20] M. D. Smet, Y. Matsumoto, A.-M. J. Zwerver, L. Tryputen, S. L. de Snoo, S. V. Amitonov, S. R. Katiraee-Far, A. Sammak, N. Samkharadze, Ö. Gül, R. N. M. Wasserman, E. Greplova, M. Rimbach-Russ, G. Scappucci, and L. M. Vandersypen, High-fidelity single-spin shuttling in silicon, Nat. Nanotech. **20**, 866 (2025).

[21] T. Struck, M. Volmer, L. Visser, T. Offermann, R. Xue, J.-S. Tu, S. Trellenkamp, Ł. Cywiński, H. Bluhm, and L. R. Schreiber, Spin-epr-pair separation by conveyor-mode single electron shuttling in si/sige, Nat. Comm. **15**, 1325 (2024).

[22] N. D. Foster, J. D. Henshaw, M. Rudolph, D. R. Luhman, and R. M. Jock, Dephasing and error dynamics affecting a singlet-triplet qubit during coherent spin shuttling, npj Quantum Inf. **11**, 63 (2025).

[23] Y. Matsumoto1, M. De Smet1, L. Tryputen, S. L. de Snoo, S. V. Amitonov, A. Sammak, M. Rimbach-Russ, G. Scappucci, and L. M. K. Vandersypen, Two-qubit logic and teleportation with mobile spin qubits in silicon, arXiv:2503.15434 (2025).

[24] M. Friesen, S. Chutia, C. Tahan, and S. N. Coppersmith, Valley splitting theory of sige/si/sige quantum wells, Phys. Rev. B **75**, 115318 (2007).

[25] A. Saraiva, M. Calderon, X. Hu, S. Das Sarma, and B. Koiller, Physical mechanisms of interface-mediated intervalley coupling in si, Phys. Rev. B **80**, 081305 (2009).

[26] M. Friesen and S. N. Coppersmith, Theory of valley-orbit coupling in a si/sige quantum dot, Phys. Rev. B **81**, 115324 (2010).

[27] F. A. Zwanenburg, A. S. Dzurak, A. Morello, M. Y. Simmons, L. C. L. Hollenberg, G. Klimeck, S. Rogge, S. N. Coppersmith, and M. A. Eriksson, Silicon quantum electronics, Rev. Mod. Phys. **85**, 961 (2013).

[28] E. Kawakami, P. Scarlino, D. R. Ward, F. R. Braakman, D. E. Savage, M. G. Lagally, M. Friesen, S. N. Coppersmith, M. A. Eriksson, and L. M. K. Vandersypen, Electrical control of a long-lived spin qubit in a si/sige quantum dot, Nature Nanotechnology **9**, 666 (2014).

[29] B. Paquelet Wuetz, M. P. Losert, S. Koelling, L. E. A. Stehouwer, A.-M. J. Zwerver, S. G. J. Philips, M. T. Madzik, X. Xue, G. Zheng, M. Lodari, S. V. Amitonov, N. Samkharadze, A. Sammak, L. M. K. Vandersypen, R. Rahman, S. N. Coppersmith, O. Moutanabbir, M. Friesen, and G. Scappucci, Atomic fluctuations lifting the energy degeneracy in Si/SiGe quantum dots, Nat. Commun. **13**, 7730 (2022).

[30] J. R. F. Lima and G. Burkard, Interface and electromagnetic effects in the valley splitting of si quantum dots, Mater. Quantum. Technol. **3**, 025004 (2023).

[31] M. P. Losert, M. A. Eriksson, R. Joynt, R. Rahman, G. Scappucci, S. N. Coppersmith, and M. Friesen, Practical strategies for enhancing the valley splitting in si/sige quantum wells, Phys. Rev. B **108**, 125405 (2023).

[32] J. Klos, J. Tröger, J. Keutgen, M. P. Losert, N. V. Abrosimov, J. Knoch, H. Bracht, S. N. Coppersmith, M. Friesen, O. Cojocaru-Mirédin, L. R. Schreiber, , and D. Bougeard, Atomistic compositional details and their importance for spin qubits in isotope-purified silicon quantum wells, Adv. Sci. **11**, 2407442 (2024).

[33] A. Thayil, L. Ermoneit, and M. Kantner, Theory of valley splitting in si/sige spin-qubits: Interplay of strain, resonances and random alloy disorder, arXiv:2412.20618 10.48550/arXiv.2412.20618 (2024).

[34] B. D. Woods, H. Soomro, E. S. Joseph, C. C. Frink, R. Joynt, M. A. Eriksson, and M. Friesen, Spin-orbit coupling and operation of multivalley spin qubits, npj Quant. Info. **10**, 54 (2024).

[35] C. Yang, A. Rossi, R. Ruskov, N. Lai, F. Mohiyaddin, S. Lee, C. Tahan, G. Klimeck, A. Morello, and A. S. Dzurak, Spin-valley lifetimes in a silicon quantum dot with tunable valley splitting, Nat. Comm. **481**, 344 (2012).

[36] A. Corna, L. Bourdet, R. Maurand, A. Crippa, D. Kotekar-Patil, H. Bohuslavskyi, R. Laviéville, L. Hutin, S. Barraud, X. Jehl, M. Vinet, S. De Franceschi, Y.-M. Niquet, and M. Sanquer, Electrically driven electron spin resonance mediated by spin–valley–orbit coupling in a silicon quantum dot, npj Quant. Info. **4**, 6 (2018).

[37] X. Zhang, R.-Z. Hu, H.-O. Li, F.-M. Jing, Y. Zhou, R.-L. Ma, M. Ni, G. Luo, G. Cao, G.-L. Wang, X. Hu, H.-W. Jiang, G.-C. Guo, and G.-P. Guo, Giant anisotropy of spin relaxation and spins-valley mixing in a silicon quantum dot, Phys. Rev. Lett. **124**, 257701 (2020).

[38] P. Huang and X. Hu, Fast spin-valley-based quantum gates in si with micromagnets, npj Quant. Info. **7**, 162 (2022).

[39] M. P. Losert, M. Oberländer, J. D. Teske, M. Volmer, L. R. Schreiber, H. Bluhm, S. Coppersmith, and M. Friesen, Strategies for enhancing spin-shuttling fidelities in Si/SiGe quantum wells with random-alloy disorder, PRX Quantum **5**, 040322 (2024).

[40] A. David, A. M. Pazhedath, L. R. Schreiber, T. Calarco, H. Bluhm, and F. Motzoi, Long distance spin shuttling enabled by few-parameter velocity optimization, arXiv:2409.07600 10.48550/arXiv.2409.07600 (2024).

[41] R. Németh, V. K. Bandaru, P. Alves, M. P. Losert, E. Brann, O. M. Eskandari, H. Soomro, A. Vivrekar, M. A. Eriksson, and M. Friesen, Omnidirectional shuttling to avoid valley excitations in Si/SiGe quantum wells, arXiv:2412.09574 10.48550/arXiv.2412.09574 (2024).

[42] M. Volmer, T. Struck, A. Sala, B. Chen, M. Oberländer, T. Offermann, R. Xue, L. Visser, J.-S. Tu, S. Trellenkamp, Ł. Cywiński, H. Bluhm, and L. R. Schreiber, Mapping of valley-splitting by conveyor-mode spin-coherent electron shuttling, npj Quantum Information **10**, 61 (2024).

[43] M. Volmer, T. Struck, A. Sala, J.-S. Tu, S. Trellenkamp, D. Degli Esposti, G. Scappucci, Ł. Cywiński, H. Bluhm, and L. R. Schreiber, Extracting intervalley coupling from symmetric g-factors via two-dimensional shuttle mapping in si/sige, arXiv:26??.????? (2026).

[44] R. Ferdous, E. Kawakami, P. Scarlino, M. P. Nowak, D. R. Ward, D. E. Savage, M. G. Lagally, S. N. Coppersmith, M. Friesen, M. A. Eriksson, L. M. K. Vandersypen, and R. Rahman, Valley dependent anisotropic spin splitting in silicon quantum dots, npj Quant. Info. **4**, 26 (2018).

[45] R. M. Jock, N. T. Jacobson, M. Rudolph, D. R. Ward, M. S. Carroll, and D. R. Luhman, A silicon singlet–triplet qubit driven by spin-valley coupling, Nature Commun. **13**, 641 (2022).

[46] M. Volmer, T. Struck, J.-S. Tu, S. Trellenkamp, D. Degli Esposti, G. Scappucci, Ł. Cywiński, H. Bluhm, and L. R. Schreiber, Reduction of the impact of the local valley splitting on the coherence of conveyor-belt spin shuttling in ^{28}si/sige, arXiv:2510.03773 (2025).

[47] X. Hao, R. Ruskov, M. Xiao, C. Tahan, and H. Jiang, Electron spin resonance and spin–valley physics in a silicon double quantum dot, Nat. Commun. **5**, 3860 (2014).

[48] J. C. C. Hwang, C. H. Yang, M. Veldhorst, N. Hendrickx, M. A. Fogarty, W. Huang, F. E. Hudson, A. Morello, and A. S. Dzurak, Impact of g-factors and valleys on spin qubits in a silicon double quantum dot, Phys. Rev. B **96**, 045302 (2017).

[49] Y.-Y. Liu, L. Orona, S. F. Neyens, E. MacQuarrie, M. Eriksson, and A. Yacoby, Magnetic-gradient-free two-axis control of a valley spin qubit in $\mathrm{si}_x\mathrm{ge}_{1-x}$, Phys. Rev. Appl. **16**, 024029 (2021).

[50] X. Cai, E. J. Connors, L. F. Edge, and J. M. Nichol, Coherent spin–valley oscillations in silicon, Nat. Phys. **19**, 386 (2023).

[51] B. D. Woods, M. P. Losert, R. Joynt, and M. Friesen, g-factor theory of si/sige quantum dots: spin-valley and giant renormalization effects, arXiv:2412.19795 10.48550/arXiv.2412.19795 (2024).

[52] B. D. Woods, M. P. Losert, N. R. Elston, M. A. Eriksson, S. N. Coppersmith, R. Joynt, and M. Friesen, g-factor theory of si/sige quantum dots: spin-valley and giant renormalization effects, arXiv:2507.05160 10.48550/arXiv.2507.05160 (2025).

[53] A. L. Saraiva, M. J. Calderón, R. B. Capaz, X. Hu, S. Das Sarma, and B. Koiller, Intervalley coupling for interface-bound electrons in silicon: An effective mass study, Phys. Rev. B **84**, 155320 (2011).

[54] D. Culcer, X. Hu, and S. Das Sarma, Interface roughness, valley-orbit coupling, and valley manipulation in quantum dots, Phys. Rev. B **82**, 205315 (2010).

[55] A. Thayil, L. Ermoneit, and M. Kantner, Theory of valley splitting in si/sige spin qubits: Interplay of strain, resonances, and random alloy disorder, Phys. Rev. B **112**, 115303 (2025).

[56] J. C. Marcks, E. C. B. Emily Eagen 4, M. P. Losert, T. Oh, J. Reily, C. S. Wang, DanielKeith, F. A. Mohiyaddin, F. Luthi, M. J. Curry, J. Zhang, F. J. Heremans, M. Friesen, and M. A. Eriksson, Valley splitting correlations across a silicon quantum well containing germanium, Nat. NComm. **16**, 11381 (2025).

[57] B. Tariq and X. Hu, Impact of the valley orbit coupling on exchange gate for spin qubits in silicon, npj Quant. Info. **8**, 53 (2011).

[58] X. Zhao and X. Hu, Measurement of tunnel coupling in a si double quantum dot based on charge sensing, Phys. Rev. Appl. **17**, 064043 (2022).

[59] M. Veldhorst, R. Ruskov, C. H. Yang, J. C. C. Hwang, F. E. Hudson, M. E. Flatté, C. Tahan, K. M. Itoh, A. Morello, and A. S. Dzurak, Spin-orbit coupling and operation of multivalley spin qubits, Phys. Rev. B **92**, 201401 (2015).

[60] R. Ruskov, M. Veldhorst, A. S. Dzurak, and C. Tahan, Electron g-factor of valley states in realistic silicon quantum dots, Phys. Rev. B **98**, 245424 (2018).

[61] L. Cvitkovich, P. Stano, C. Wilhelmer, D. Waldhör, D. Loss, Y.-M. Niquet, and T. Grasser, Coherence limit due to hyperfine interaction with nuclei in the barrier material of Si spin qubits, Phys. Rev. Appl. **22**, 064089 (2024).

[62] P. Huang and X. Hu, Spin relaxation in a si quantum dot due to spin-valley mixing, Phys. Rev. B **90**, 235315 (2014).

[63] J. M. Taylor, J. R. Petta, A. C. Johnson, A. Yacoby, C. M. Marcus, and M. D. Lukin, Relaxation, dephasing, and quantum control of electron spins in double quantum dots, Phys. Rev. B **76**, 035315 (2007).

[64] S. N. Shevchenko, S. Ashhab, and F. Nori, Landau-Zener-Stückelberg Interferometry, Phys. Rep. **492**, 1 (2010).

[65] J. A. Krzywda and L. Cywiński, Adiabatic electron charge transfer between two quantum dots in presence of $1/f$ noise, Phys. Rev. B **101**, 035303 (2020).

[66] J. A. Krzywda and L. Cywiński, Interplay of charge noise and coupling to phonons in adiabatic electron transfer between quantum dots, Phys. Rev. B **104**, 075439 (2021).

[67] S. Blundobler and V. Elser, S-matrix for generalized landau-zener problem, J. Phys. A: Math. Gen. **26**, 1211 (1993).

[68] L. Kranz, S. K. Gorman, B. Thorgrimsson, Y. He, D. Keith, J. G. Keizer, and M. Y. Simmons, Exploiting a single-crystal environment to minimize the charge noise on qubits in silicon, Adv. Mat. **32**, 2003361 (2020).

[69] A. Hollmann, T. Struck, V. Langrock, A. Schmidbauer, F. Schauer, T. Leonhardt, K. Sawano, H. Riemann, N. V. Abrosimov, D. Bougeard, and L. R. Schreiber, Large, tunable valley splitting and single-spin relaxation mechanisms in a Si/si$_x$ge$_{1-x}$ quantum dot, Phys. Rev. Applied **13**, 034068 (2020).

[70] G. Falci, A. D'Arrigo, A. Mastellone, and E. Paladino, Initial decoherence in solid state qubits, Phys. Rev. Lett. **94**, 167002 (2005).

[71] G. Ithier, E. Collin, P. Joyez, P. J. Meeson, D. Vion, D. Esteve, F. Chiarello, A. Shnirman, Y. Makhlin, J. Schriefl, and G. Schön, Decoherence in a superconducting quantum bit circuit, Phys. Rev. B **72**, 134519 (2005).

[72] F. H. L. Koppens, D. Klauser, W. A. Coish, K. C. Nowack, L. P. Kouwenhoven, D. Loss, and L. M. K. Vandersypen, Universal phase shift and nonexponential decay of driven single-spin oscillations, Phys. Rev. Lett. **99**, 106803 (2007).

[73] G. Ramon and L. Cywiński, Qubit decoherence under two-axis coupling to low-frequency noises, Phys. Rev. B **105**, L041303 (2022).

[74] J. Yoneda, J. S. Rojas-Arias, P. Stano, K. Takeda, A. Noiri, T. Nakajima, D. Loss, and S. Tarucha, Noise-correlation spectrum for a pair of spin qubits in silicon, Nat. Phys. **19**, 1793 (2023).

~~Reduction of the impact~~ Impact of the local valley splitting on the coherence of conveyor-belt spin shuttling in ^{28}Si/SiGe

Mats Volmer,[1] Tom Struck,[1] Arnau Sala,[1] Jhih-Sian Tu,[2] Stefan Trellenkamp,[2] Davide Degli Esposti,[3] Giordano Scappucci,[3] Łukasz Cywiński,[4] Hendrik Bluhm,[1] and Lars R. Schreiber[1,*]

[1] *JARA-FIT Institute for Quantum Information, Forschungszentrum Jülich GmbH and RWTH Aachen University, Aachen, Germany*
[2] *Helmholtz Nano Facility (HNF), Forschungszentrum Jülich, Jülich, Germany*
[3] *QuTech and Kavli Institute of Nanoscience, Delft University of Technology, Lorentzweg 1, 2628 CJ Delft, The Netherlands*
[4] *Institute of Physics, Polish Academy of Sciences, Warsaw, Poland*

Electron spins in silicon offer a promising path toward scalable, fault-tolerant quantum computing, with the potential to host millions of qubits. However, scaling up dense quantum-dot arrays and enabling qubit interconnections through shuttling are hindered by uncontrolled lateral variations of the valley splitting energy E_{VS}. We map E_{VS} across a 40 nm × 400 nm region of a ^{28}Si/Si$_{0.7}$Ge$_{0.3}$ shuttle device and analyze the spin coherence of a single electron spin transported by conveyor-belt shuttling. We observe that the E_{VS} varies over a wide range from 1.5 µeV to 200 µeV and is dominated by SiGe alloy disorder. In regions of low E_{VS} and at spin-valley resonances, spin coherence is reduced and its dependence on shuttle velocity matches predictions. Rapid and frequent traversal of low-E_{VS} regions induces a regime of enhanced spin coherence explained by motional narrowing. By selecting shuttle trajectories that avoid problematic areas on the E_{VS} map, we achieve transport over tens of microns with coherence limited by the coupling to a static electron spin entangled with the mobile qubit. Our results provide experimental confirmation of the theory of spin-decoherence of mobile electron spin-qubits and present practical strategies to integrate conveyor-mode qubit shuttling into silicon quantum chips.

INTRODUCTION

Single electron spins confined to gate-defined static quantum dots (QDs) in highly isotopically purified planar ^{28}Si/SiGe quantum wells [1] represent excellent spin qubits [2–5]. Their manipulation, initialization, and detection fidelities [6–11] have reached the quantum error correction threshold and are competing with other qubit platforms [12–14]. Spin qubits in silicon excel due to their tiny footprint and compatibility with silicon foundry fabrication [15–18]. Proposals for scalable architectures address cross-talk and wiring fanout bottlenecks [19] by employing mobile spin qubits [20–25] and are applicable to quantum error correction protocols [26–30].

In particular, the transport of electrons across distances of tens of microns, confined to one of 34 (linear) and 51 (T-junction) moving Si/SiGe quantum dots, has been demonstrated [31, 32]. Such a conveyor-mode shuttler, also called QuBus, requires only four control signals, independent of its length [33, 34], which is highly advantageous for co-integration with classical control tiles [19, 35]. High-fidelity spin qubit transport, two-qubit gates, and entanglement distribution have also been demonstrated using conveyor-mode shuttling [36–38]. However, the shuttle lanes in such devices have so far been shorter than 0.5 µm. According to theory, spin coherence during conveyor-mode shuttling is limited by valley excitations, which depend on shuttle velocity, magnetic field, and valley coupling [34, 39, 40]. The latter is determined by the local arrangement of Ge atoms at the alloy-disordered Si/SiGe interface at the position of the quantum dot [41–46]. In particular, uncontrolled excitations might occur at spots with low valley splitting energy E_{VS}, which is the energy difference between the valley ground state and the first excited valley state [47]. Enhancing valley splitting has therefore become crucial for the Si/SiGe platform [46, 48, 49], since regions of low E_{VS} degrade the performance of both static and mobile qubits and pose a substantial challenge for scalability [3, 34, 50]. Schemes for optimal control of the spin-qubit shuttling process, balancing fidelity and signal complexity, have been proposed [51–53], but experimental validation is still lacking.

Here, we explore this interplay of spin coherence and conveyor-mode shuttling for a known lateral map of E_{VS} in an isotopically purified ^{28}Si/SiGe QuBus. First, we employ our shuttle-based method to measure a detailed two-dimensional E_{VS} map of our device [43]. Second, we investigate the spin coherence of the shuttle process across a specific path through this E_{VS} landscape for various magnetic fields, highlighting the impact of spin-valley resonances and regions of low E_{VS}. Finally, we shuttle along optimal and non-optimal trajectories at various velocities and B-fields, repeatedly reaching accumulated shuttle distances of several tens of microns with an infidelity of less than 8% for a shuttle distance of 10 µm.

* lars.schreiber@physik.rwth-aachen.de

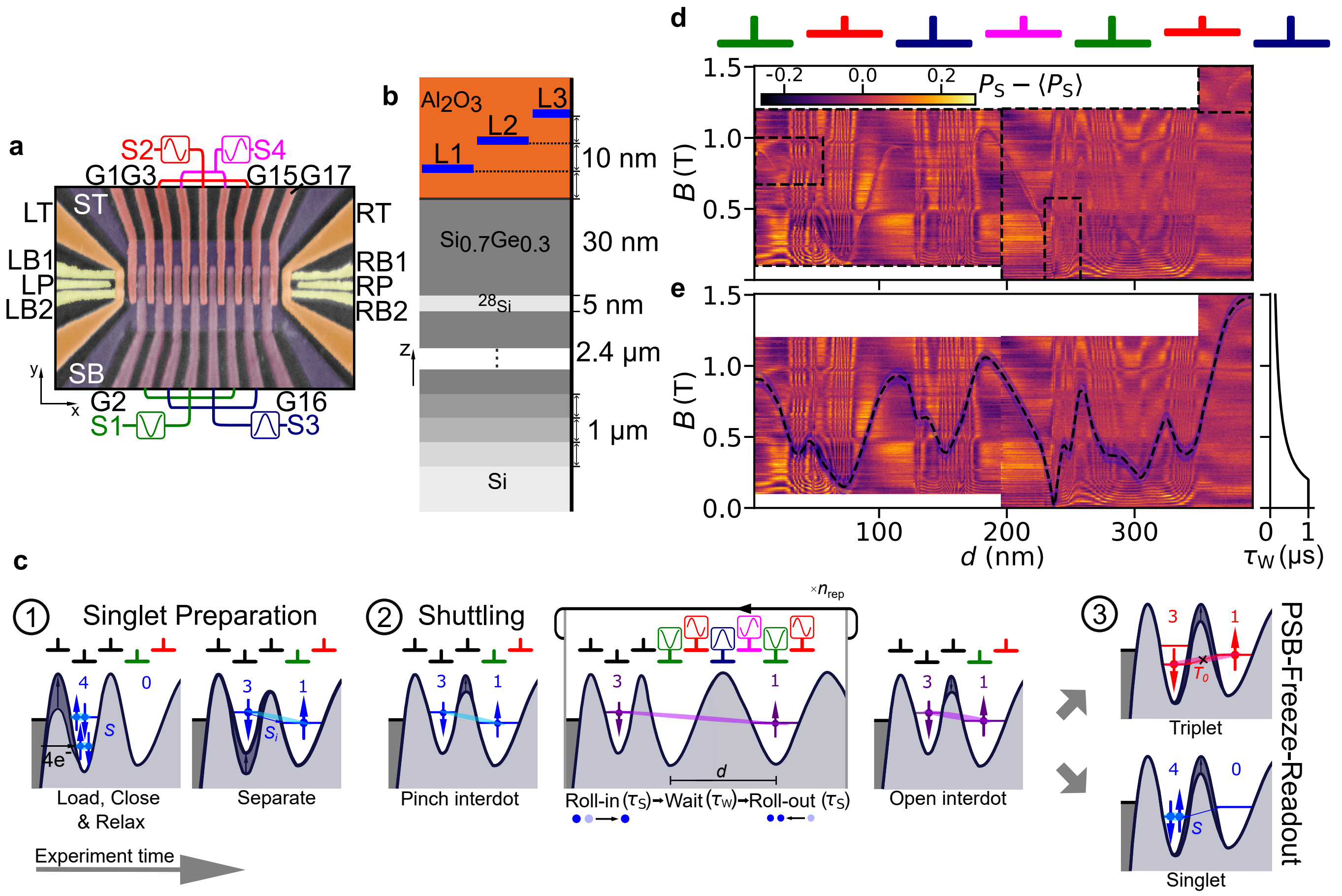

FIG. 1. Device and method. (a) False-colored scanning electron micrograph of the three labeled metal layers of the device identical to the one used in this experiment. Electrical connection of S-gates according to solid lines. Scale bar is 500 nm. (b) Schematic cross-section of the device including Si-buffer, relaxed SiGe (dark gray), Si (white), metallic gates (blue) isolated by Al_2O_3 (orange). (c) Pulse sequence of the experiment explained by the schematic electrostatic potential at the left side of the one dimensional electron channel (1DEC). Three stages – singlet preparation, shuttling and Pauli Spin Blockade (PSB) readout are explained in the text. Horizontal lines represent gates and numbers are QD occupation. All pulse sequences are parametrized by $\{d, \tau_W, \tau_S, n_{rep}, B\}$. (d) Singlet return probability P_S as a function of magnetic field B and shuttle distance d for $y = 6\,\mathrm{nm}$ set by constant voltages 0.2 V and 0.1 V applied to gates ST and SB, respectively. $P_S(d, B)$ is composed of five measurements (each enclosed by dashed lines). Positions of clavier gates on L2 and L3 with respect to d are indicated by symbols on top. (e) Same as d but $E_{VS}(d)$ is highlighted by the black dashed spline which is carefully positioned by eye. Right: Wait time $\tau_W(B)$.

Device and methods

We use the left side of a $^{28}Si/Si_{0.7}Ge_{0.3}$ linear QuBus shuttle device with two single electron transistors (SETs) at the end of a 1.2 μm long shuttle section defined by three patterned metal layers (Fig. 1a). Shuttle (S)-gates used for the conveyor-mode shuttling in Fig. 1a are electrically connected outside the chip similar to Refs. [31, 33, 36, 43]. Thus, the shuttle gates for the four gate sets S1-S4 are controlled by only four voltages. This eases the tuning of the voltages and integration of future cryo-electronics. The SETs are used as proximal charge sensors and electron reservoirs for the linear shuttle section. The device consists of a semiconductor heterostructure as described in Ref. [54] and three electrically isolated metal layers (cross-section in Fig. 1b). Details of the device design and fabrication can be found in the methods section.

The device is operated at 60 mK within a global in-plane magnetic field B and controlled by voltage pulses applied to the six gates G2, G3, and S1–S4 (sketched in Fig. 1c). First, the device is tuned electrostatically into the operational regime, at which one SET is formed by gates LB1, LP and LB2, and a double quantum dot (DQD) is formed by gates G1–G3 and the leftmost gates of S1 and S2 (right plunger and right barrier, respectively). Voltages applied to gates S1 through S4 form the QuBus shuttle, which consists of a series of not tunnel-coupled QDs. Each pulse sequence starts with the singlet preparation (1st step in Fig. 1c). First, we (re)load four electrons into the leftmost quantum dot

(QD) and wait for 100 µs until the loaded spin mixture has relaxed into two spin-singlet states in the two lowest ~~valley-orbit~~ orbital and valley states available. Then, we tunnel one of the spin-entangled electrons into the right QD of the DQD and raise the interdot barrier by a negative voltage on gate G3 (2nd step in Fig. 1c). Now, the system can be described by a spin-singlet (S) state ~~with a potentially mixed valley-state~~, S_i, where $i \in \{|+-\rangle, |--\rangle, |++\rangle, |-+\rangle\}$ [55] ~~represents the valley state of~~ labels the singly-occupied valley states in the left and the right QD. Two electrons in the left QD remain as an additional, but inert spin-singlet [43] and can be neglected for further state description. At the third stage (Fig. 1c), the conveyor-mode shuttling is activated by applying sinusoidal voltage pulses $V_{S,i}$ on the four gates S_i

$$V_{S,i} = A_i \cdot \cos(2\pi f t - (i-1)\cdot \pi/2) + B_i. \qquad (1)$$

where f is the drive frequency, A_i is the constant drive amplitude (150 mV for S1 and S3, 180 mV for S2 and S4) and B_i is the constant voltage offset (0.7 V for S1 and S3, 0.84 V for S2 and S4, similar to [43]). This pulse separates the right electron spin in a moving QD from the other three electrons, which remain in the static leftmost QD formed underneath the gate G2. The corresponding one-way shuttle time $\tau_S/2$ and distance d are given by $d = \lambda \cdot f \cdot \tau_S/2$, where $\lambda = 280$ nm is the period of the shuttle signal (equal to four times the gate pitch), while the shuttling velocity is given by $v_S \equiv 2d/\tau_S$. After shuttling, we optionally wait for a time τ_W before shuttling back by the time-reversed shuttle pulse, thus a shuttle cycle takes $\tau_S + \tau_W$. Optionally, the shuttle cycle is repeated n_{rep} times, resulting in a total time of $n_{rep}(\tau_S + \tau_W)$. After shuttling, we lower the interdot barrier and detune the DQD into Pauli Spin Blockade (PSB) for 500 ns to trigger the spin-selective charge transition. Finally, the charge state is frozen by raising the interdot barrier (3rd step in Fig. 1c) and the spin-singlet (S) is discriminated from the spin-triplet (T) by the SET current in a single shot. The singlet return probability P_S is then calculated from the statistics of at least 800 pulse sequence repetitions.

RESULTS

Valley splitting map

In order to explore the impact of E_{VS} on spin-coherent conveyor-mode shuttling, we map the local valley splitting utilizing the strong renormalization of the shuttled electron g-factor $g_{R\nu}$ (ν represents the valley state) when the local valley splitting matches the Zeeman splitting [43]. Notably, we directly measure the valley splitting of a QD. The matching is independent of the initialized valley state (see Supplementary Note I) and we do not approximate the valley splitting by measuring the singlet-triplet splitting within one QD as it is the case for other methods. For one trace $E_{VS}(d)$, we record P_S for various B (stepped by 5 mT) following the pulse sequence using $n_{rep} = 1$, a constant frequency of $f = 20$ MHz for all $d \in [0\,\text{nm}, 392\,\text{nm}]$ (Fig. 1d). In contrast to Ref. [43], we observe larger variations of E_{VS} and need to expand the scanned B range in order to identify the spin-valley anticrossings for all d (black dashed spline line in Fig. 1e), where E_{VS} equals the local Zeeman energy of the shuttled QD positioned at distance d for a fixed time $\tau_W(B)$ as plotted in the right panel of Fig. 1e. The chosen dependence of τ_W on B ensures that the acquired Larmor-phase $\varphi_W \propto B \cdot \tau_W$ at d is balanced with B for optimal visibility of the spin-valley anticrossings (see Supplementary Note II for details).

Indeed, we track $E_{VS}(d)$ despite some P_S background due to the static QD (at $B = 0.95$ T) and some vertical features due to a large change of electron g-factor difference $\Delta g_{\mu\nu}(d) = g_{L\mu} - g_{R\nu}(d)$ where L is the static QD and R the mobile QD, while $\mu, \nu \in \{-, +\}$ are the valley indices, see Ref. [43]. Additionally, to compensate for slow variations in detection contrast, we align the data by subtracting the linewise mean $\langle P_S \rangle$. Note that the spin-valley resonances and thus $E_{VS}(d)$ seamlessly match at the borders of the $P_S(B, d)$ patches (dashed lines in Fig. 1d) measured on different days. This underlines that the $E_{VS}(d)$ is a robust and static property of the shuttle device. Sometimes remeasured patches with higher resolution were required if the $E_{VS}(d)$ trace could not be identified unambiguously in the first place.

In order to extend to a two-dimensional map $E_{VS}(d, y)$, we repeat the measurement protocol above and record seven traces that are offset by 6 nm perpendicular to the one-dimensional electron channel (1DEC) (see Fig. 2a). Each trace contains 400 measurement points along the 392 nm shuttle distance (see Supplementary Note III for raw data of every trace). The long split gate (gates SB and ST) is set to 150 mV at $y = 0$ nm. The perpendicular offset is set by applying a symmetric voltage bias of 100 mV per 6 nm on gates ST and SB. We calibrated this offset by triangulation of the QDs y-position in a similar device [43].

We observe several spots at which E_{VS} is close to zero with a global minimum of $E_{VS} = 1.5 \pm 1.2$ µeV at $(d, y) = (231\,\text{nm}, 12\,\text{nm})$. According to our literature research, this is the smallest E_{VS} value directly measured and published so far. For example, we find two close local minima with $E_{VS} \leq 5$ µeV at $(d, y) = (232\,\text{nm}, 0\,\text{nm})$ and at $(d, y) = (245\,\text{nm}, 0\,\text{nm})$, which merge into one local minimum towards $y = \pm 12$ nm. Such features were observed in a previous two-dimensional map of E_{VS} [43] and can be motivated by E_{VS} being proportional to the modulus of the complex ~~spin-valley coupling Δ_{sv}~~ intervalley coupling Δ (see [42] and Supplementary Note IV). Remarkably, the two traces for $y = \pm 18$ nm exhibit $E_{VS} > 20$ µeV spanning from $d = 0$ nm to $d = 280$ nm.

From the full two-dimensional E_{VS} map, we calculate a histogram of E_{VS} containing 2800 samples (Fig. 2b), as a total trace spanning 392 nm contains 400 E_{VS} points.

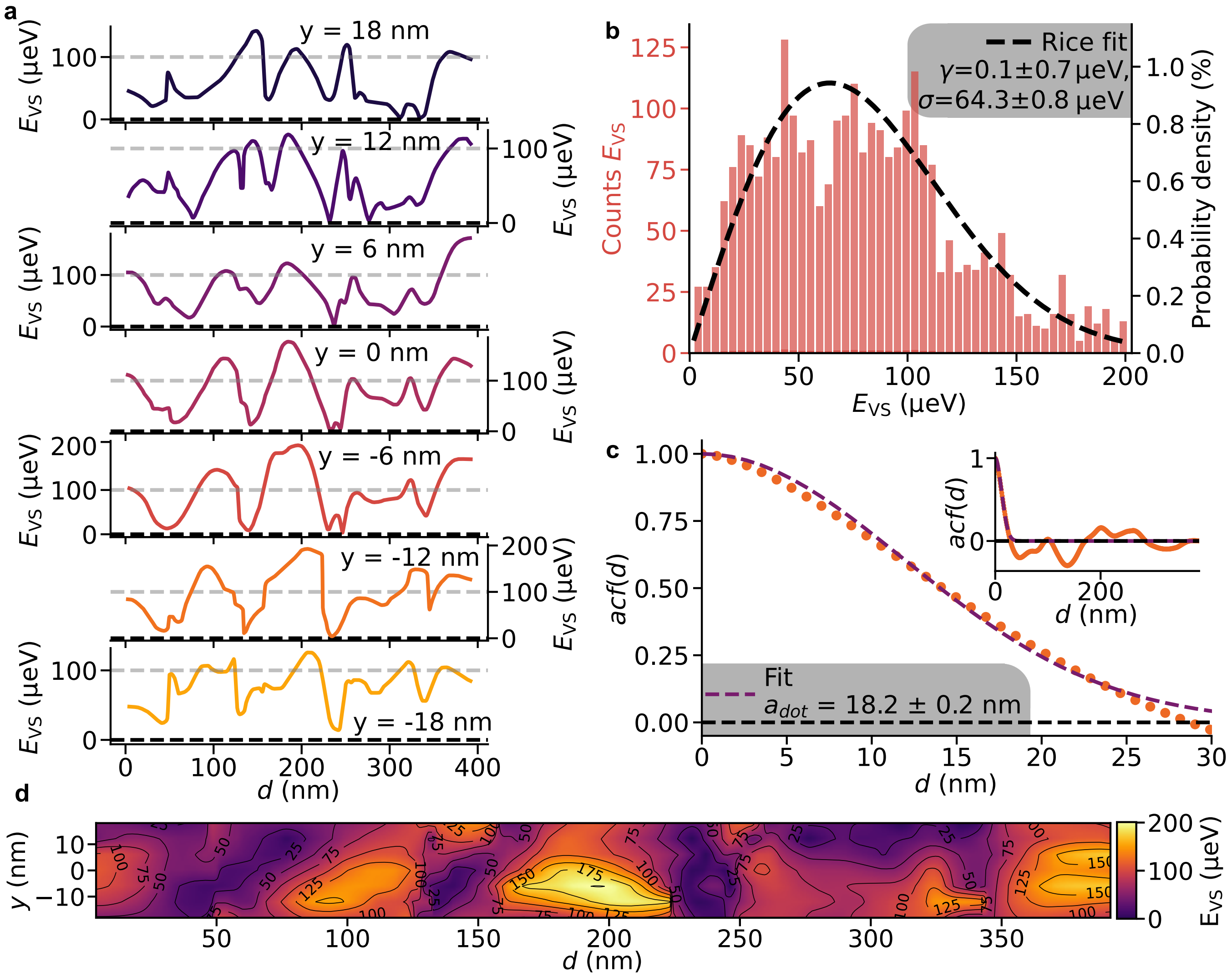

FIG. 2. Valley splitting map. (a) Valley splitting traces as a function of shuttle distance d for different 1DEC positions y. (b) Histogram of the valley splitting measurements. A fit of a Rice probability density (black dashed line) is parameterized by mean γ and width σ (cmp. Ref. [43]). (c) Autocorrelation function (acf) of the valley splitting as a function of shuttle distance d (averaged over all traces). A Gaussian fit (dashed, purple) is included. Inset: Zoom-out. (d) Two-dimensional valley splitting map $E_{\mathrm{VS}}(d, y)$ by linear interpolation of the traces in a.

The histogram follows a Rice distribution [43] that has a small deterministic component ~~$\gamma = 0.0(6)$ μeV and a large spread $\sigma = 61.5(8)$ μeV~~ $\gamma = 0.1(7)$ µeV and a large spread $\sigma = 64.3(8)$ µeV suggesting larger impact of alloy disorder than in another heterostructure measured before [43, 44, 56]. This can be explained by the thinner quantum well, which results in more wave function overlap with the SiGe barrier, leading to larger average E_{VS} than previously measured [43], but at the expense of larger variation of valley splitting.

The histogram of the E_{VS} map thus gives important insight into the origin of E_{VS} and can be employed as a benchmark for the quality of the semiconductor heterostructure. We also compute the autocorrelation function acf(d) for all seven traces and plot the average in Fig. 2c. Fitting the acf and thus the size of the shuttled QD by the formula that applies when spatial randomness of E_{VS} is due to alloy disorder at the interface [43, 48]

$$\mathrm{acf}(d) = \exp\left(-\frac{1}{4-\pi}\frac{d^2}{a_{\mathrm{dot}}^2}\right), \qquad (2)$$

we find a QD ~~size~~radius of ~~$a_{\mathrm{dot}} = 17.3(1)$ nm~~$a_{\mathrm{dot}} = 18.2(2)$ nm, similar to Ref. [43] (16 nm) and Ref. [44] (19.2 nm). The inset shows no correlation beyond 30 nm. This implies that samples in the histogram are correlated and deviations from the Rice distribution predicted by theory of SiGe alloy disorder are not significant. Finally, we combine all E_{VS} traces by linear interpolation to create one two-dimensional valley splitting map in Fig. 2d. This map extends to almost four times the size of a pre-

viously published map of a heterostructure with natural abundance of silicon isotopes [43] and has a three times larger span of valley splitting values. This implies 122 samples of measured E_{VS} spaced by a distance of a QD radius being the correlation length. As the mapping method might become a significant tool for tracking the material progress for Si/SiGe qubit chips, we added comments on the time efficiency of the method into the method section Efficiency of valley mapping.

Spin decoherence during shuttling

Next, we use the knowledge of the local valley splitting landscape to explore the impact of local E_{VS} on the spin coherence during conveyor-mode shuttling. We start with a shuttle trajectory set to $y = 0\,\mathrm{nm}$ with $E_{\mathrm{VS}}(d, y = 0)$ shown in Fig. 3a. We explore $P_{\mathrm{S}}(d, \tau_{\mathrm{S}})$ for a fixed magnetic field B and $n_{\mathrm{rep}} = 1, \tau_{\mathrm{W}} = 0$.

We start with $B = 1.7\,\mathrm{T}$ (Fig. 3b), since the Zeeman energy of the moving QD satisfies $E_{\mathrm{Z}} > \max(E_{\mathrm{VS}}(d))$ (yellow line in Fig. 3a) and we expect no ~~spin decoherence~~ drop in ST-oscillation amplitude at spin-valley resonances defined by $E_{\mathrm{Z}} = E_{\mathrm{VS}}$ [34]. For $P_{\mathrm{S}}(d, \tau_{\mathrm{S}})$, we use various shuttle velocities, and some $P_{\mathrm{S}}(d, \tau_{\mathrm{S}})$ are inaccessible (white areas) due to the maximum shuttle velocity of 5.6 m/s. Similar to Ref. [36], we observe variations in the singlet-triplet oscillation frequency stemming from $\Delta g(d)$. More importantly, the amplitude of the singlet-triplet oscillations ~~and thus the spin coherence~~ is suddenly reduced at $d \approx 240\,\mathrm{nm}$ (dotted vertical lines in Fig. 3a,b). This is remarkable and not unexpected, as this sudden loss of ~~spin coherence~~ ST-oscillation amplitude coincides with the first spot at which $E_{\mathrm{VS}} < 5\,\mu\mathrm{eV}$.

The $P_{\mathrm{S}}(d, \tau_{\mathrm{S}})$ measured at $B = 0.3\,\mathrm{T}$ (Fig. 3c with maximal velocity 2.8 m/s) exhibits a smaller frequency of ST-oscillations, as expected, but also reveals very different ~~spin-decoherence~~ signal-decay characteristics. Here, we observe a sudden reduction of the visibility of the oscillations and thus increase of ~~spin-decoherence~~ signal decay after passage beyond $d = 50\,\mathrm{nm}$ (dashed line in Fig. 3c). Remarkably, this coincides with the first spin-valley resonance passed by shuttling (dashed vertical red lines in Fig. 3a). Repeating the measurement with a reduced $B = 0.1\,\mathrm{T}$ (Fig. 3d), the sudden loss of ~~spin coherence~~ signal appears at larger $d = 240\,\mathrm{nm}$ (black solid line), which coincides with the first spin-valley resonance at the corresponding reduced E_{Z} (solid violet line in Fig. 3d). Furthermore, when following $P_{\mathrm{S}}(d, \tau_{\mathrm{S}})$ oscillations for constant shuttle velocities (see arrows in Fig. 3c as a guide), we observe that shuttling across spin-valley resonances with higher velocity tends to preserve the ~~spin coherence~~ ST-oscillations compared to lower shuttle velocities for both magnetic fields. Specifically, at the largest $v_{\mathrm{S}} = 2.8\,\mathrm{m/s}$, the oscillation amplitude is unchanged and the ~~spin coherence~~ signal strength is not affected by the resonance. The opposite trend is observed if the onset of ~~spin decoherence~~ signal decay coincides with the crossing of valley splitting minimum in Fig. 3b.

We support our observations by the Fourier transforms of $P_{\mathrm{S}}(d, \tau_{\mathrm{S}})$, plotted as functions of frequency f divided by $2\mu_B B/h$ (Fig. 3e-g) underneath the corresponding panels Fig. 3b,c,d. This extracts the integrated difference Δg of the electron g-factors for various shuttle distances d, because the acquired phase during shuttling is the integral over the Larmor phases ϕ the electron spin acquires along its shuttle trajectory [36]

$$\phi_{\mu\nu} = 2 \cdot \int_0^{\tau_{\mathrm{S}}/2} \frac{\mu_B B}{\hbar} \Delta g_{\mu\nu}(x(t)) dt \approx \frac{\mu_B B}{\hbar} \overline{\Delta g}_{\mu\nu}(d) \cdot \tau_{\mathrm{S}}, \tag{3}$$

where $\overline{\Delta g}_{\mu\nu}(d) = \frac{1}{d} \int_0^d \Delta g_{\mu\nu}(x) \mathrm{d}x$, and we have assumed that v_{S} is approximately constant despite the presence of electrostatic disorder.

In Fig. 3e, we observe one dominant $\overline{\Delta g}_{\mu\nu}(d)$ and one faint component amplified by the log-color-scale. They correspond to two distinct singlet-triplet frequencies. Importantly, the amplitude of both drastically change at approximately 240 nm, matching the minimum of E_{VS}, at which a sudden reduction of the spin coherence is observed. Strikingly, the two frequency components split into four. We interpret these frequencies in terms of mixtures of valley states of the initialized spin-singlet: Before shuttling the singlet is in a dominant valley state and a small admixture of a different valley state in the static QD [55]. This remains unchanged during the shuttling through regions of large E_{VS}. After passing the E_{VS} minimum, however, both valley states of the shuttled QD become occupied as well (see Supplementary Notes IV and VI), resulting in a total of four singlet-triplet frequencies corresponding to four $\mu\nu \in \{++, --, +-, -+\}$ combinations. Hence, the passage of the E_{VS} minimum causes a partial loss of spin coherence and partial valley excitation, and therefore the shuttling is not fully adiabatic [34]. The magnitude of the $\overline{\Delta g}_{\mu\nu}(d)$ components and their symmetry will be explained in more detail elsewhere, but it is important to keep in mind that $\overline{\Delta g}_{\mu\nu}(d)$ depends on all $\Delta g_{\mu\nu}(x)$ on the shuttle trajectory according to Eq. 3.

The Fourier transform of the singlet-triplet data recorded at $B = 0.3\,\mathrm{T}$ (Fig. 3f) again reveals the initialization of a mixture of valley occupations, with one component dominant over the other. After the passage of the first spin-valley resonance, both components nearly vanish. Interestingly, at larger d the visibility of one component tends to recover and vanish in the vicinity of the passage through the next resonance marked by another vertical dashed line (Fig. 3f, $d = 150\,\mathrm{nm} - 220\,\mathrm{nm}$). The reason for the apparent recovery of the oscillations is a peculiarity of the $P_{\mathrm{S}}(d, \tau_{\mathrm{S}})$ measurement scheme. Larger values of d are dominantly recorded at larger shuttle velocities, and consequently shuttle sequences with small velocities do not contribute to the Fourier transform at larger d. Since the passage of spin-valley resonances with larger velocities tends to preserve the spin coherence, as we noted above, the coherence at larger d tends to re-

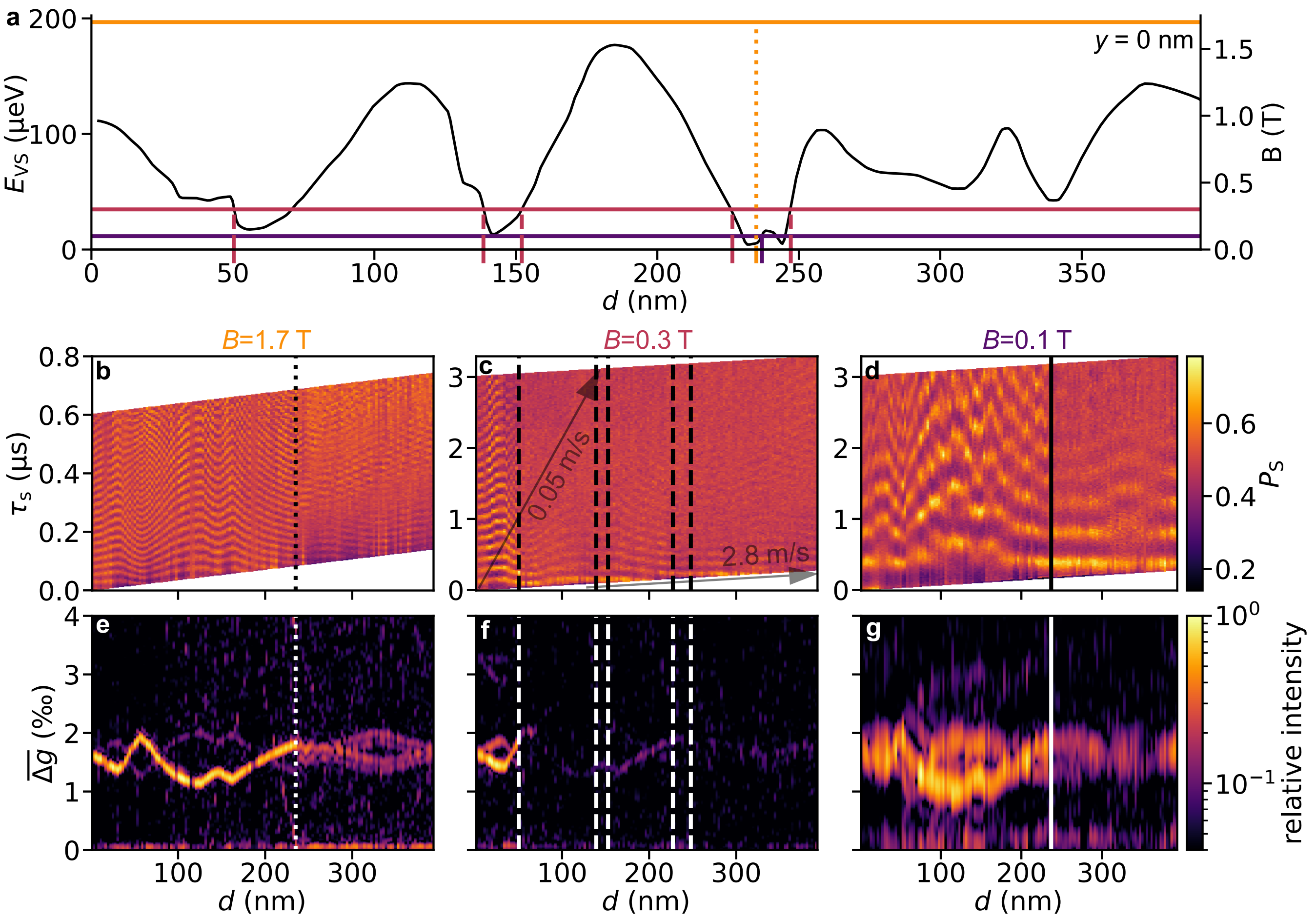

FIG. 3. Impact of E_{VS} on coherent spin shuttling. (a) Valley splitting trace at $y = 0$ nm from Fig. 2a with colored horizontal lines indicating the Zeeman energy for $B \in \{1.7, 0.3, 0.1\}$ T. Spin-valley resonances are marked by vertical dashed lines and a region of low E_{VS} by a dotted line. (b,c,d) Singlet-return probability P_S as a function of shuttle distance d and shuttle time τ_S (for one direction) measured with the fixed three magnetic fields $B \in \{1.7, 0.3, 0.1\}$ T are shown in b,c and d, respectively. Vertical lines indicate spin-valley resonances and region of low E_{VS} from a. Traces for fixed shuttle velocities (e,f,g) are indicated by the arrows in c. (e,f,g) FFT along τ_S of the data shown above as a function of integrated g-factor difference $\overline{\Delta g}$ for magnetic fields $B \in \{1.7, 0.3, 0.1\}$ T. A version of panels (b-g) can be found in Supplementary Note V.

cover. A natural explanation of this behavior is the transition between adiabatic and diabatic passage through the spin-valley resonance [34]. The adiabatic passage at low v_S leads to spin-valley flip-flop that converts superposition of spin states into a superposition of valley states (see Supplementary Note VII). The latter is then rapidly dephased due to valley splitting fluctuations (see Supplementary Note VIIIB). We quantitatively confirm this effect by simulation of the data in Fig. 3f based on realistic assumption of the spin-valley-coupling $\Delta_{sv} \lesssim 300$ neV and the recorded E_{VS} map (see Supplementary Note IX), from which $dE_{VS}/dx \approx 3\,\mu$eV/nm is extracted at the resonance. The probability P_{svf} for a spin-valley flip-flop at the spin-valley resonance is:

$$P_{svf} = 1 - \exp\left(-2\pi\Delta_{sv}^2/\hbar v_\delta\right), \quad (4)$$

where $v_\delta \equiv |dE_{VS}/dx| \cdot v_S$. For $v_S > 2.8$ m/s, we find $P_{svf} \ll 1$; thus the passage is diabatic, and the spin-valley flip-flop is suppressed.

The Fourier transform of the singlet-triplet data recorded at $B = 0.1$ T is broadened as only a few singlet-triplet oscillations are recorded (Fig. 3g). Most importantly, the amplitudes of the components abruptly diminish at the passage of the first spin-valley resonance located at $d = 230$ nm according to the mapped E_{VS}. Hence, it confirms our notion of the impact of the spin-valley resonance for the spin coherence of the shuttle process.

In summary, we observe two spin dephasing channels directly related to the E_{VS} map. First, passing a region of small E_{VS}, the spin coherence is partly lost and both valley states in the mobile QD become occupied. Second, passing a spin-valley resonance, defined by $E_Z = E_{VS}$, with low v_S results in conversion of spin qubit to a valley qubit that suffers stronger dephasing caused by E_{VS} fluctuations. At higher velocities (here $v_S > 2.8$ m/s),

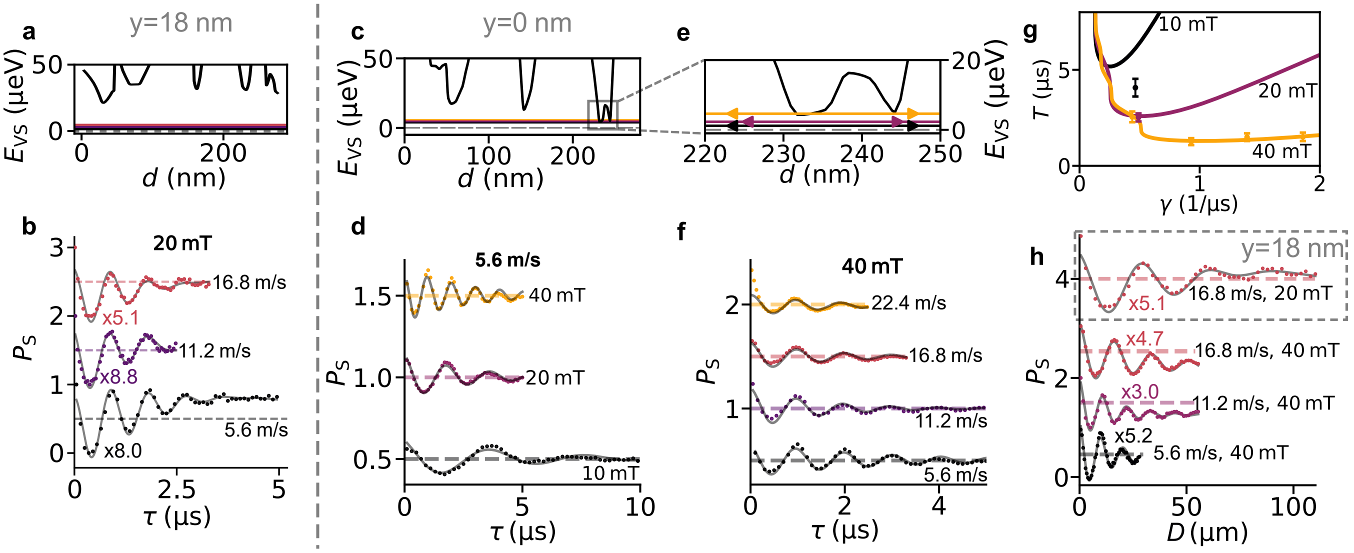

FIG. 4. Repetitive impact of shuttling through an $E_{\mathrm{VS}}(x)$ landscape on coherent spin shuttling. (a) Zoom-in of E_{VS} trace at $y = 18\,\mathrm{nm}$ from Fig. 2a with colored horizontal lines indicating the Zeeman energy for $B = 20\,\mathrm{mT}$. (b) P_{S} as a function of total shuttle time $\tau = 2n_{\mathrm{rep}}\tau_{\mathrm{S}}$ recorded at $B = 20\,\mathrm{mT}$ and at labeled shuttle velocity v_{S}. Every data point represents n_{rep} shuttling of distance 280 nm and back at $y = 18\,\mathrm{nm}$. Solid gray lines are least-squares fits to the data. Dashed horizontal lines are the mean of all data points in the scan. Data points are scaled by given factor to normalized visibility to 1. P_{S} is offset vertically by 1 for clarity. (c) Zoom-in of valley splitting trace at $y = 0\,\mathrm{nm}$ from Fig. 2a with colored horizontal lines indicating the Zeeman energy for $B \in \{10, 20, 40\}\,\mathrm{mT}$. (d) Same as in b, but $y = 0\,\mathrm{nm}$ and fixed shuttle velocity $v_{\mathrm{S}} = 5.6\,\mathrm{m/s}$ and $B \in \{10, 20, 40\}\,\mathrm{mT}$. P_{S} is offset vertically by 0.5 for clarity. (e) Same as in c, but larger zoom. (f) Same as in d, but for fixed magnetic field $B = 40\,\mathrm{mT}$ and shuttle velocities $v_{\mathrm{S}} \in \{5.6, 11.2, 16.8, 22.4\}\mathrm{m/s}$. For the fit of the 22.4 m/s dataset, we exclude the first three points for fitting. (g) Modeled exponential decay timescale T for repetitive impact of E_{VS} minimum as a function of valley excitation rate γ and B (solid lines) with the six fitted T values (see also Tab. I) from panels d and f marked as solid circles. (h) P_{S} as a function of total shuttle distance $D = 2n_{\mathrm{rep}}\lambda$ for various (v_{S}, B, y). Data and fits are taken from panels b,d,f and rescaled according to label with P_{S} offset vertically for clarity.

the passage can be fully diabatic and no spin-valley-flip occurs. In Supplementary Note VIII, all mechanisms of decoherence during the shuttling such as hyperfine noise (VIIIA), valley splitting fluctuations activated by spin-valley flip-flop (VIIIB), spin relaxation near a spin-valley resonance (VIIIC) and valley excitation (VIIID) are discussed in more details. Calculation reproducing the features seen in Fig. 3f is described in Supplementary Note IX.

Shuttling across larger distances

Finally, we quantitatively investigate the spin coherence of the shuttle process in an extended parameter space. In order to amplify the impact of spots of interest on the E_{VS} map, we repeat the shuttle process forward and backward n_{rep} times at a fixed shuttle trajectory of distance $\lambda = 280\,\mathrm{nm}$ (one period), a fixed shuttle velocity $v_{\mathrm{S}} = \lambda f$, a fixed magnetic field B and $\tau_{\mathrm{W}} = 0$ (Fig. 1c). Then the decay of P_{S} (the loss of coherence of the spin singlet) as a function of the accumulated shuttle time τ at a total distance $D = 2n_{\mathrm{rep}} \cdot 280\,\mathrm{nm}$ is fitted. Each measurement was taken over the span of approximately ten minutes.

First, we select a shuttle trajectory without near-zero E_{VS} values ($y = 18\,\mathrm{nm}$, $x = 0 - 280\,\mathrm{nm}$ in Fig 2a) and $B = 20\,\mathrm{mT}$ as a reference. Any spin-valley resonances are avoided, since $E_{\mathrm{Z}} \ll E_{\mathrm{VS}}(x)$ (Fig. 4a). $P_{\mathrm{S}}(\tau)$ is measured for three shuttle velocities (Fig. 4b). We observe decaying spin-singlet oscillations on top of a rising background. Most importantly, the curves and more specifically the decay is independent of the shuttle velocity v_{S}. Hence, the total shuttle time τ, and not the accumulated shuttled distance D seem to influence the spin decoherence during the shuttle process. Note that in Ref. [34], we observed rising $T_2^*(d)$ as a function of d due to motional narrowing. Here, motional narrowing might enhance the $T_2^*(D)$ as well, but equally for all $D = 2n_{\mathrm{rep}}\lambda$, since quasistatic noise is averaged across the distance of λ only, but for all n_{rep}, thus for all data points recorded, see Supplementary Note VIIIA. The rising background might be due to a very slow singlet-triplet oscillation, stemming from a different valley-combination of the occupied singlet state. We capture it by a second frequency component:

$$P_{\mathrm{S}}(\tau, T_2^*) = A_1 \cos(\omega_1 \tau + \phi_1)\, e^{-\tau^2/T_2^{*2}} + A_2 \cos(\omega_2 \tau + \phi_2) + \tfrac{1}{2} + \epsilon, \qquad (5)$$

where T_2^* is the decay of the entangled singlet state and ϵ, A_i, ϕ_i are constants capturing SPAM errors and relative valley occupation and oscillation phase. We find a good fit with a Gaussian decay, but the first P_S data point of all three traces is systematically too large for unknown reason. We find $T_2^* \approx 1.7\,\mu s$ (Tab. I), which is a reasonable value for a spin singlet decay in an isotopically purified ^{28}Si/SiGe, since the value is expected to be by a factor of $\sqrt{2}$ smaller than the single spin ensemble dephasing time [11]. Remarkably, the spin-singlet decay in a DQD with raised barrier (shuttle distance $d = 0\,$nm) is found to be 1.4 µs, which is longer than for the natural Si/SiGe ($\approx 0.6\,\mu s$ in Ref. [36]), but shorter than the decay including the shuttle process extended by motional narrowing and presumably limited by the spin dephasing in the static (left) QD. Hence, the shuttle process tends to not add to the spin-decoherence of the spin singlet, if the impact of E_{VS} minima and spin-valley resonances can be avoided. The shuttle distance can be increased by larger shuttle velocity as only the shuttling time compared to the T_2^* corrected by motional narrowing is governing the singlet decay.

Next, we alter the shuttle trajectory to $y = 0\,$nm, $x = 0 - 280\,$nm and thus cross a region of near-zero E_{VS} (Fig. 4c), at which we expect an impact on the spin coherence. We measure $P_S(\tau)$ for $B \in [10, 20, 40]\,$mT at a fixed $v_S = 5.6\,$m/s (Fig. 4d). The trajectory does not cross a spin-valley resonance at $B = 10\,$mT, while there are several such passages at $B = 40\,$mT (Fig. 4e). We observe that the decay of $P_S(\tau)$ is significantly longer for $B = 10\,$mT and about equal at $B = 20\,$mT and $B = 40\,$mT. Hence, we can shuttle the spin the longest distance D at $B = 10\,$mT. At first sight, it is surprising that the decay at $B = 20\,$mT and $B = 40\,$mT is about equal, as there is no passage of spin-valley resonance at $B = 20\,$mT. Furthermore, we observe that the decay of $P_S(\tau)$ and thus the spin coherence recorded at $B = 40\,$mT tends to decrease with increasing shuttle velocity (Fig. 4f), although $v_S > 2.8\,$m/s, and therefore passage across the spin-valley resonances should be almost diabatic (no spin-valley-flip occurs), as we noted above, and the timescale of decay of coherence due to spin-valley flip-flop should be velocity-independent, see Supplementary Note XA.

Therefore, a detailed analysis of the B and v_S dependence of the passage of the region with near-zero E_{VS} is required. We show that the phenomena in Fig. 4d,f can be explained by the passage of the region of small E_{VS} alone. First, we note that the form of P_S decay is better approximated by an exponential than by the Gaussian used in Eq. (5) if the decoherence is caused by accumulation of errors occurring over time τ

$$P_S(\tau) = A\cos(\omega\tau + \phi)e^{-\tau/T} + \tfrac{1}{2} + \epsilon, \qquad (6)$$

where T is the fitted decay time (Tab. I) and A, ϵ are constants capturing SPAM errors. Indeed, for all parameters B and v_S, except for the lowest values ($B = 10\,$mT, $v_S = 5.6\,$m/s), the exponential decay fitted the measured data in Fig. 4d,f significantly better than a Gaussian decay. The first three data points for Fig. 4f (22.4 m/s) are excluded from the fit, as they show an additional exponential background. This suggests that for the $y = 0$ shuttling trajectory the decoherence for $B > 10\,$mT is dominated by dynamic processes affecting the shuttled spin instead of just inhomogeneous spin dephasing T_2^*.

y (nm)	v (m/s)	B (mT)	T_2^* (µs)	T (µs)
18	16.8	20	1.5 ± 0.1	
18	11.2	20	1.5 ± 0.1	
18	5.6	20	2.0 ± 0.1	
0	22.4	40		1.5 ± 0.2
0	16.8	40		1.5 ± 0.2
0	11.2	40		1.3 ± 0.2
0	5.6	40		2.6 ± 0.1
0	5.6	20		2.5 ± 0.1
0	5.6	10		4.1 ± 0.5

TABLE I. Fit parameters for the cumulative shuttling measurements. For $y = 18\,$nm, we fit the data using Eq. 5. The T_2^* corresponds to the dephasing parameter in the fit equation. For $y = 0\,$nm, we fit the data using Eq. 6 and extract the decay time T. Note that we measure the dephasing of a separated entangled spin state, and therefore T_2^* and T include dephasing of the spins in the static and the mobile QDs.

Spin dephasing due to multiple passages through a valley splitting minimum is caused by valley-flip events that lead to accumulation of stochastic phase contributions due to valley-dependent g-factor, $g_{R\nu}(d)$ of the shuttled spin, and random times spent in each valley state during the total shuttling time $\tau = n_{\mathrm{rep}}\tau_S$. As valley relaxation can be neglected [34], this dephasing is controlled by two parameters: $\delta\bar{\omega} \equiv \bar{\omega}_+ - \bar{\omega}_-$, in which $\bar{\omega}_\nu = \mu_B B \overline{\Delta g_{\mu\nu}}(\lambda)/\hbar$, with $\overline{\Delta g_{\mu\nu}}$ from Eq. (3), is evaluated for the shuttled spin in valley $\nu = \pm$, and the effective valley flip rate $\gamma \equiv Q_v(v_S)v_S/\lambda$, in which $Q_v(v_S)$ is the probability of a valley-flip per passage. For $\gamma \gg \delta\bar{\omega}$ the net random phase accumulated due to multiple valley flips undergoes motional narrowing, the coherence decay is exponential, and its timescale is $T = 2\gamma/\delta\bar{\omega}^2$. On the other hand, for $\gamma \ll \delta\bar{\omega}$ the decay is approximately exponential, with timescale $T = 1/\gamma \propto 1/(Q_v v_S)$. Thus, the $T(\gamma)$ dependence is non-monotonic, with the minimum dephasing time $T_{\min} \approx 2/\delta\bar{\omega}$ obtained for $\gamma \approx \delta\bar{\omega}/\sqrt{2}$ (Fig. 4g). If we assume approximately velocity-independent Q_v, which holds for strongly nonadiabatic dynamics expected for shuttling in the relevant velocity range for $E_{VS}(d)$ from Fig. 4e, then $T(\gamma, B)$ depends on only the two QuBus-specific parameters $(Q_v, \delta\bar{\omega})$. Fitting $T(B, \gamma)$ from Tab. I to the theory, we find reasonable values $Q_v = 0.023 \pm 0.003$ and $\delta\bar{\omega}/\mu_B B \approx 4.8 \cdot 10^{-4} \pm 3 \cdot 10^{-5}$ yielding a good fit to our model (detailed discussion in Supplementary Note XB). The largest value of T from data recorded at the lowest B is most probably dominated by the dephasing in the static QD, and thus does

not reach the theoretical value for the mobile QD. Therefore, we excluded it from the fit of the model. Let us stress the key observation that the decoherence time T can be enhanced not only by lowering Q_v (by lowering v_S, since at low enough velocities Q_v certainly decreases with decreasing v_S) [34], but also by increasing v_S, and thus Q_v, in order to reach the motional narrowing regime, which is analogous to the magnetic-field-dependent Dyakonov-Perel spin-dephasing mechanisms for carrier-scattering of free spin ensembles [57].

Finally, we compare the decay of P_S in terms of total shuttle distance D for various shuttle trajectories and parameters v_S and B (Fig. 4h). Targeting maximum spin-coherent shuttle distance without valley excitations, the shuttle velocity should be high and the trajectory should avoid minima of E_VS. Picking such a trajectory, we find a Gaussian decay dominated by the inhomogenous spin-dephasing of the spin in the static QD and enhanced spin-dephasing of the mobile QD due to motional narrowing. For a pessimistic estimate, we assume both ensemble spin-dephasing times to be equal and arrive at a spin shuttle fidelity of $\mathcal{F} = 92\,\%$ across a total distance $D = 10\,\mu\mathrm{m}$ from

$$\mathcal{F}(D) = \exp\left(-\left(\frac{D}{v_\mathrm{S}\sqrt{2}T_2^*}\right)^2\right), \tag{7}$$

for a shuttling velocity of $v_\mathrm{S} = 16.8\,\mathrm{m/s}$ at $y = 18\,\mathrm{nm}$ with $T_2^* = 1.5\,\mu\mathrm{s}$ for the spin singlet. Comparable literature focused on high-fidelity spin shuttling [37], surpasses this. As we did not optimize the experiment for fast shuttle speeds, and have additional decay due to the static electron, this is expected.

DISCUSSION

We have explored the spin decoherence mechanisms during conveyor-mode shuttling of a single electron spin in an isotopically purified ^{28}Si/SiGe QuBus and its correlation to the measured two dimensional map of valley splitting energies. The values of E_VS in the map range between $1.5\,\mu\mathrm{eV}$ and $200\,\mu\mathrm{eV}$, and follow predictions of E_VS governed by SiGe alloy disorder [48]. Specifically, the trend towards a larger spread of E_VS with larger mean values matches the thinner quantum well mapped here compared to Ref. [43]. Controlling the shuttle trajectory, velocity, and magnetic field, we observe the local impact of E_VS during shuttling of a single spin. The observed impact on spin coherence of special points on the E_VS map can be categorized into two types: (I) Decoherence due to valley excitation in the mobile QD. This valley excitation occurs in regions of low E_VS. (II) Spin-valley flip-flop due to a passage through a spin-valley resonance that turns the spin superposition into a quickly dephasing valley superposition. Our observations confirm the relevance of these spin-dephasing mechanisms during the conveyor-belt spin shuttling [34], but also trigger the prediction of a new regime of enhanced spin coherence due to a large number of excitation events controlled by enhancing the shuttle velocity. This transition is an analogue of the cross-over of the Elliott-Yafet to the magnetic-field-dependent Dyakonov-Perel spin-dephasing mechanisms ~~found at~~ for carrier-scattering of free spin ensembles in spintronics [58]. Additionally mapping the g-factor, we can evaluate the complex intervalley coupling, offering a complete analysis of the valley physics [59]. Our work suggests and supports strategies for achieving high-fidelity spin-shuttling at large distances [53, 60–62]. First, local spots of valley excitations (predominantly found at low E_VS) and spin-valley resonances can be avoided by appropriate two-dimensional trajectories [51], and/or the overall E_VS has to be enhanced by advanced heterostructures [48, 63–65]. Our mapping method of E_VS is the key to exploit these strategies and we exploit that the E_VS is highly reproducible for a device within a thermal cycle. We observe that high shuttle velocities are preferable beyond running the quantum chip at a high clock rate: (I) Larger shuttle distances are achieved at a given static T_2^*, which is enhanced by motional narrowing of quasistatic spin splitting noise. Here, we find a maximal infidelity of $8\,\%$ for a shuttle distance of $10\,\mu\mathrm{m}$. (II) Spots of spin-valley resonance are diabatically passed. At very high shuttle velocities, low magnetic fields and large distances, the Dyakonov-Perel dephasing regime is entered, at which trajectories with many spots of valley excitation are preferred. In our experiment, we trigger this transition by repeatedly passing an E_VS region with two small minima. Notably, our measurements discriminate only spin states and probably shuttling in the Dyakonov-Perel regime results in a mixed occupancy of the involved two valley states in the mobile QD. This poses a problem for spin manipulation unless mitigated by valley relaxation.

The transition to mobile spin qubits and high-fidelity conveyor-mode spin shuttling is considered the key enabler for scalable architectures of semiconductor quantum computers. We present confirmation of predicted spin-dephasing mechanisms, predict an alternative regime of high-fidelity shuttling, and support current strategies for advancing the fidelity of spin-qubit conveyor-mode shuttling.

METHODS

Device fabrication and design

The device consists of a $\mathrm{Si_{0.7}Ge_{0.3}}/^{28}\mathrm{Si}/\mathrm{Si_{0.7}Ge_{0.3}}$ heterostructure grown on a silicon (001) wafer by CVD. It forms an electron quantum well in the 5 nm thick, isotopically purified ^{28}Si layer with <800 ppm ^{29}Si concentration (Fig. 1b). The upper $\mathrm{Si_{0.7}Ge_{0.3}}$ layer has a nominal thickness of 30 nm and is passivated by an amorphous Si-rich cap [54]. The lower SiGe barrier is $2.4\,\mu\mathrm{m}$ thick and grown on a relaxed, stepped SiGe buffer made of three equidistant $1\,\mu\mathrm{m}$ thick steps. The tensile strained ^{28}Si

layer is significantly thinner than the Matthews-Blakeslee critical thickness for strain relaxation. This minimizes the chance of having relaxation-induced crystal defects at the critical quantum well interfaces and has the potential to increase valley splitting energies, as the wave function overlap with the SiGe barriers is increased [42]. Ohmic contacts are fabricated by phosphorus ion implantation at Helmholtz-Center Dresden-Rossendorf, followed by thermal activation at 730 °C for 30 s. The three metal gate layers fabricated at the Helmholtz Nano Facility [66] (called L1, L2 and L3 from bottom to top; positions in Fig. 1b) are made of a Ti/Pt ebeam-evaporated stack [67] with thickness 5 nm and 15 nm/22 nm/29 nm, respectively. They are shaped by 100 keV electron-beam lithography and a metal lift-off process. They are electrically isolated from each other and the SiGe heterostructure by three 10 nm amorphous Al_2O_3 layers fabricated by atomic layer deposition.

The device design features two single electron transistors used as proximal charge sensors and electron reservoirs [68] positioned at the ends of a one dimensional electron channel (1DEC) (Fig. 1a). The 1DEC is formed by a 1.2 µm split gate in the (110) crystallographic direction on the first metal layer (L1: SB, ST; purple) with 200 nm spacing. It is covered by 17 narrow clavier gates distributed among metal layers L2 and L3 (L2: G2, S1, S3, G16; L3: G1, G3, S2, S4, G15, G17; dark magenta and bright red) with a combined gate pitch of 70 nm. Importantly, all S-gates labeled the same are electrically connected outside the chip as sketched in Fig. 1a, similar to Refs. [31, 33, 36, 43]. Thus, the clavier gates for the four gate sets S1-S4 are controlled by only four voltages. Each SET consists of three finger gates (L1: LP, RP; L2: LB1, LB2, RB1, RB2; yellow in Fig. 1a) and one top gate (L3: LT, RT; orange). The shuttle operation is limited due to the rightmost gate of S4 being unintentionally disconnected.

Experimental setup

The device is mounted on the mixing chamber stage of an Oxford Instruments dry dilution refrigerator at a temperature of ≈ 60 mK and a global in-plane magnetic field generated by a superconducting magnet. All gates are connected via 10 kHz low-pass filtered loom lines (DC lines). Additionally, signals on G2, G3 as well as S1-S4 inside the refrigerator are carried to the sample PCB via coaxial cables with a bandwidth of 20 GHz (RF lines) and are added to the DC lines with RC bias tees with a cutoff frequency of 5 Hz. At room temperature, DC voltages are generally applied using custom-made low-noise digital-to-analog converters. Pulsed voltages are applied by Zürich Instruments HDAWG arbitrary waveform generators on the RF lines. For each of the high frequency gates, the voltage pulses are also added to the DC lines in order to avoid transients from long measurements. The SET current is read-out by Basel Precision Instruments transimpedance amplifiers and AlazarTech digitizer cards.

Efficiency of valley splitting mapping

Mapping the local valley splitting is an efficient method for benchmarking heterostructures. Here, we estimate time requirements from state-of-the-art values for spin initialization and detection. The total measurement time T_{meas} for a two-dimensional map is a product of the following parameters

$$T_{\mathrm{meas}} = \tau_{ss} \cdot n_B \cdot n_{\mathrm{samples}} \cdot n_x \cdot n_y + \tau_{\mathrm{B}}, \quad (8)$$

where τ_{ss} is the single-shot measurement time of PSB, $n_x = l_x/\delta_x$ and $n_y = l_y/\delta_y$ are the number of samples in parallel and perpendicular direction to the 1DEC, each given by the ratio of total size l_i and resolution δ_i. n_B is the number of magnetic fields required to resolve the valley splittings clearly and n_{samples} the number of repetitions to calculate P_{S}. τ_B is the time it takes to change to a different magnetic field.

Parameter	Value
τ_{ss}	2 µs [4]
n_B	600
n_{samples}	100
τ_{B}	1 s · n_B
l_x	10 µm [31]
δ_x	5 nm
n_x	2000
l_y	40 nm
δ_y	5 nm
n_y	8
T_{meas}	2520 s

TABLE II. State-of-art values for our time estimate of a two-dimensional valley splitting map of size $l_x \times l_y$.

Our time estimate is based on values according to Tab. II. We suggest a lateral spacing of 5 nm as this is smaller than the quantum dot size. Hence, we arrive at 2000 sample points along the 1DEC and eight traces. The magnetic field needs to be swept at least in fields that cover the low valley splitting ranges below ≈ 180 µeV, so 1800 mT with 3 mT precision, thus $n_B = 600$. We include 1 s for changing the magnetic field to the next value. Sufficient precision of P_{S} is achieved by $n_{\mathrm{samples}} = 100$. Ideally, one would work with pulsed voltages on the long split gate defining the position of the 1DEC. This way, a retuning of the SET between displacements of the 1DEC (change of y) is not required. Since SET tuning is a matter of quality of automation, we omit it here.

We observe a slight drop in visibility of the signature of valley resonance, if the electron passes several spin-valley hotspots along its trajectory. Passing serveral spin-valley hotspots can be mitigated by shuttling faster and ultimately mitigated by optimizing the shuttle trajectory. We suggest the following algorithm to enlarge the area:

First, an area of 40 nm by 500 nm is mapped and the best shuttle trajectory selected. This trajectory is used to reach the next area (of the same size as the first one) in order to perform mapping there. Then a third area is added and the previous two are crossed by the optimal trajectory, and so on.

In summary, a valley splitting map of a 10 micron shuttle device can be obtained fast to either find the best shuttle trajectory or to just benchmark heterostructures. The former can be further accelerated by a smart optimization algorithm that does not require measurement of the full 2D map. As the valley splitting map is encoded by the position of the Ge atoms in the quantum well region of a shuttle device, frequent recalibration is not necessary.

DATA AVAILABILITY

The data that support the findings of this study are available in the Zenodo repository.

ACKNOWLEDGEMENTS

We acknowledge valuable discussions with Merritt P. Losert and Mark Friesen and the support of the Dresden High Magnetic Field Laboratory (HLD) at the Helmholtz-Zentrum Dresden - Rossendorf (HZDR), member of the European Magnetic Field Laboratory (EMFL). This work was funded by the German Research Foundation (DFG) within the project 289786932 (SCHR 1404/2-2) and under Germany's Excellence Strategy - Cluster of Excellence Matter and Light for Quantum Computing" (ML4Q) EXC 2004/2 - 390534769, and by the European Union's Horizon Research and Innovation Actions under Grant Agreement No. 101174557 (QLSI2). The device fabrication has been done at HNF - Helmholtz Nano Facility, Research Center Juelich GmbH [66]. This research was sponsored in part by The Netherlands Ministry of Defence under Awards No. QuBits R23/009. The views, conclusions, and recommendations contained in this document are those of the authors and are not necessarily endorsed nor should they be interpreted as representing the official policies, either expressed or implied, of The Netherlands Ministry of Defence. The Netherlands Ministry of Defence is authorized to reproduce and distribute reprints for Government purposes notwithstanding any copyright notation herein.

AUTHOR CONTRIBUTIONS

M.V. set up and conducted the experiments assisted by T.S. Authors M.V., T.S., Ł.C. and L.R.S. analyzed the data. The heterostructure was grown by D.D.E. and G.S. Device fabrication was done by J.T. and S.T and device pre-screening by A.S. Theory has been developed by Ł.C with help of L.R.S and M.V. The experiment was designed and supervised by L.R.S. L.R.S. and H.B. provided guidance to all authors. M.V., Ł.C. and L.R.S. wrote the manuscript which was commented on by all other authors.

COMPETING INTERESTS

H.B, L.R.S., T.S. and M.V. are co-inventors of patent applications that cover conveyor-mode shuttling and/or its applications. G.S. is founding advisor of Groove Quantum BV and declares equity interests. L.R.S. and H.B. are founders and shareholders of ARQUE Systems GmbH. The other authors declare no competing interest.

---

[1] Burkard, G., Ladd, T. D., Pan, A., Nichol, J. M. & Petta, J. R. Semiconductor spin qubits. *Rev. Mod. Phys.* **95**, 025003 (2023).

[2] Struck, T. *et al.* Low-frequency spin qubit energy splitting noise in highly purified ^{28}Si/SiGe. *npj Quantum Inf.* **6**, 2056 (2020).

[3] Philips, S. G. J. *et al.* Universal control of a six-qubit quantum processor in silicon. *Nature* **609**, 919–924 (2022).

[4] Stano, P. & Loss, D. Review of performance metrics of spin qubits in gated semiconducting nanostructures. *Nat. Rev. Phys.* **4**, 672 (2022).

[5] Fernández de Fuentes, I. *et al.* Running a six-qubit quantum circuit on a silicon spin-qubit array. *PRX Quantum* **7**, 010308 (2026).

[6] Yoneda, J. *et al.* A quantum-dot spin qubit with coherence limited by charge noise and fidelity higher than 99.9%. *Nat. Nanotechnol.* **13**, 102 (2018).

[7] Struck, T. *et al.* Robust and fast post-processing of single-shot spin qubit detection events with a neural network. *Sci. Rep.* **11**, 16203 (2021).

[8] Noiri, A. *et al.* Fast universal quantum gate above the fault-tolerance threshold in silicon. *Nature* **601**, 338 (2022).

[9] Mills, A. R. *et al.* Two-qubit silicon quantum processor with operation fidelity exceeding 99%. *Sci. Adv.* **8**, eabn5130 (2022).

[10] Xue, X. *et al.* Quantum logic with spin qubits crossing the surface code threshold. *Nature* **601**, 343 (2022).

[11] Wu, Y.-H. *et al.* Simultaneous high-fidelity single-qubit gates in a spin qubit array (2025). Arxiv preprint at https://arxiv.org/abs/2507.11918.

[12] Bluvstein, D. *et al.* A quantum processor based on coherent transport of entangled atom arrays. *Nature* **604**, 451–456 (2022).

[13] Arute, F. *et al.* Quantum supremacy using a programmable superconducting processor. *Nature* (2019).

[14] Monroe, C. *et al.* Programmable quantum simulations of spin systems with trapped ions. *Rev. Mod. Phys.* **93**, 025001 (2021).
[15] Zwerver, A. M. J. *et al.* Qubits made by advanced semiconductor manufacturing. *Nat. Electron.* **5**, 184–190 (2022).
[16] Neyens, S. *et al.* Probing single electrons across 300-mm spin qubit wafers. *Nature* **629**, 80–85 (2024).
[17] George, H. C. *et al.* 12-Spin-Qubit Arrays Fabricated on a 300 mm Semiconductor Manufacturing Line. *Nano Letters* **25**, 793–799 (2025).
[18] Huckemann, T. *et al.* Industrially fabricated single-electron quantum dots in Si/SiGe heterostructures. *IEEE Electron Device Lett.* **46**, 868–871 (2025).
[19] Vandersypen, L. M. K. *et al.* Interfacing spin qubits in quantum dots and donors—hot, dense, and coherent. *npj Quantum Inf.* **3**, 34 (2017).
[20] Fujita, T., Baart, T. A., Reichl, C., Wegscheider, W. & Vandersypen, L. M. K. Coherent shuttle of electron-spin states. *npj Quantum Inf.* **3**, 22 (2017).
[21] Mills, A. R. *et al.* Shuttling a single charge across a one-dimensional array of silicon quantum dots. *Nat. Commun.* **10**, 1063 (2019).
[22] Yoneda, J. *et al.* Coherent spin qubit transport in silicon. *Nat. Commun.* **12**, 4114 (2021).
[23] Noiri, A. *et al.* A shuttling-based two-qubit logic gate for linking distant silicon quantum processors. *Nat. Commun.* **13**, 5740 (2022).
[24] Zwerver, A. *et al.* Shuttling an electron spin through a silicon quantum dot array. *PRX Quantum* **4**, 030303 (2023).
[25] Foster, N. D., Henshaw, J. D., Rudolph, M., Luhman, D. R. & Jock, R. M. Dephasing and error dynamics affecting a singlet–triplet qubit during coherent spin shuttling. *npj Quantum Inf.* **11**, 63 (2025).
[26] Taylor, J. M. *et al.* Fault-tolerant architecture for quantum computation using electrically controlled semiconductor spins. *Nat. Phys.* **1**, 177–183 (2005).
[27] Boter, J. M. *et al.* Spiderweb array: A sparse spin-qubit array. *Phys. Rev. Appl.* **18**, 024053 (2022).
[28] Künne, M. *et al.* The SpinBus architecture for scaling spin qubits with electron shuttling. *Nat. Commun.* **15**, 4977 (2024).
[29] Ginzel, F. *et al.* Scalable parity architecture with a shuttling-based spin qubit processor. *Phys. Rev. B* **110**, 075302 (2024).
[30] Yenilen, B., Sala, A., Bluhm, H., Müller, M. & Rispler, M. Performance of the spin qubit shuttling architecture for a surface code implementation (2025). Preprint at https://arxiv.org/abs/2503.10601.
[31] Xue, R. *et al.* Si/SiGe QuBus for single electron information-processing devices with memory and micron-scale connectivity function. *Nat. Commun.* **15**, 2296 (2024).
[32] Beer, M. *et al.* Conveyor-mode electron shuttling through a T-junction in Si/SiGe (2026). 2601.03942.
[33] Seidler, I. *et al.* Conveyor-mode single-electron shuttling in Si/SiGe for a scalable quantum computing architecture. *npj Quantum Inf.* **8**, 100 (2022).
[34] Langrock, V. *et al.* Blueprint of a scalable spin qubit shuttle device for coherent mid-range qubit transfer in disordered Si/SiGe/SiO_2. *PRX Quantum* **4**, 020305 (2023).
[35] Zhao, Q.-T. *et al.* Ultra-low-power cryogenic complementary metal oxide semiconductor technology. *Nat. Rev. Electr. Eng.* **2**, 277–290 (2025).
[36] Struck, T. *et al.* Spin-EPR-pair separation by conveyor-mode single electron shuttling in Si/SiGe. *Nat. Commun.* **15**, 1325 (2024).
[37] De Smet, M. *et al.* High-fidelity single-spin shuttling in silicon. *Nat. Nanotechnol.* **20**, 866–872 (2025).
[38] Matsumoto, Y. *et al.* Two-qubit logic and teleportation with mobile spin qubits in silicon (2025). Preprint at https://arxiv.org/abs/2503.15434.
[39] Lima, J. R. F. & Burkard, G. Valley splitting depending on the size and location of a silicon quantum dot. *Phys. Rev. Mater.* **8**, 036202 (2024).
[40] Lima, J. R. F. & Burkard, G. Partial landau-zener transitions and applications to qubit shuttling. *Phys. Rev. B* **111**, 235439 (2025).
[41] Hollmann, A. *et al.* Large, tunable valley splitting and single-spin relaxation mechanisms in a Si/Si_xGe_{1-x} quantum dot. *Phys. Rev. Appl.* **13**, 034068 (2020).
[42] Paquelet Wuetz, B. *et al.* Atomic fluctuations lifting the energy degeneracy in Si/SiGe quantum dots. *Nat. Commun.* **13**, 7730 (2022).
[43] Volmer, M. *et al.* Mapping of valley splitting by conveyor-mode spin-coherent electron shuttling. *npj Quantum Inf.* **10**, 61 (2024).
[44] Marcks, J. C. *et al.* Valley splitting correlations across a silicon quantum well containing germanium. *Nat. Commun.* **16**, 11381 (2025).
[45] Klos, J. *et al.* Atomistic compositional details and their importance for spin qubits in isotope-purified silicon quantum wells. *Adv. Sci.* **11**, 2407442 (2024).
[46] Thayil, A., Ermoneit, L. & Kantner, M. Theory of valley splitting in Si/SiGe spin qubits: Interplay of strain, resonances, and random alloy disorder. *Phys. Rev. B* **112**, 115303 (2025).
[47] Friesen, M., Chutia, S., Tahan, C. & Coppersmith, S. N. Valley splitting theory of SiGe/Si/SiGe quantum wells. *Phys. Rev. B* **75**, 115318 (2007).
[48] Losert, M. P. *et al.* Practical strategies for enhancing the valley splitting in Si/SiGe quantum wells. *Phys. Rev. B* **108**, 125405 (2023).
[49] Woods, B. D. *et al.* Coupling conduction-band valleys in SiGe heterostructures via shear strain and Ge concentration oscillations. *npj Quantum Inf.* **10**, 54 (2024).
[50] Kawakami, E. *et al.* Electrical control of a long-lived spin qubit in a Si/SiGe quantum dot. *Nat. Nanotechnol.* **9**, 666 (2014).
[51] Losert, M. P. *et al.* Strategies for enhancing spin-shuttling fidelities in Si/SiGe quantum wells with random-alloy disorder. *PRX Quantum* **5**, 040322 (2024).
[52] David, A. *et al.* Long distance spin shuttling enabled by few-parameter velocity optimization (2024). Preprint at https://arxiv.org/abs/2409.07600.
[53] Németh, R. *et al.* Omnidirectional shuttling to avoid valley excitations in Si/SiGe quantum wells. *PRX Quantum* **7**, 010336 (2026).
[54] Paquelet Wuetz, B. *et al.* Reducing charge noise in quantum dots by using thin silicon quantum wells. *Nat. Commun.* **14**, 1385 (2023).
[55] Cywinski, Ł. *et al.* Singlet-triplet oscillations in multi-valley Si quantum dots (2026). In preparation.
[56] Chen, E. H. *et al.* Detuning axis pulsed spectroscopy of valley-orbital states in Si/Si-Ge quantum dots. *Phys.*

Rev. Appl. **15**, 044033 (2021).

[57] D'yakonov, M. I. & Perel', V. I. Spin relaxation of conduction electrons in noncentrosymmetric semiconductors. *Soviet Physics Solid State* **13**, 3023–3026 (1972). Translated from Fiz. Tverd. Tela 13, 3581–3585 (1971).

[58] Žutić, I., Fabian, J. & Das Sarma, S. Spintronics: Fundamentals and applications. *Rev. Mod. Phys.* **76**, 323–410 (2004).

[59] Volmer, M. *et al.* Mapping g-factors and complex intervalley coupling in Si/SiGe by conveyor-mode shuttling (2026). Preprint at https://arxiv.org/abs/2603.01844.

[60] Lima, J. R. F. & Burkard, G. Interface and electromagnetic effects in the valley splitting of Si quantum dots. *Mater. Quantum Technol.* **3**, 025004 (2023).

[61] Oda, Y., Losert, M. P. & Kestner, J. P. Suppressing si valley excitation and valley-induced spin dephasing for long-distance shuttling. *Phys. Rev. Lett.* **136**, 020802 (2026).

[62] Pazhedath, A. M. *et al.* Large spin-shuttling oscillations enabling high-fidelity single-qubit gates. *Phys. Rev. Appl.* **24**, 034029 (2025).

[63] McJunkin, T. *et al.* Valley splittings in Si/SiGe quantum dots with a germanium spike in the silicon well. *Phys. Rev. B* **104**, 085406 (2021).

[64] McJunkin, T. *et al.* SiGe quantum wells with oscillating Ge concentrations for quantum dot qubits. *Nat. Commun.* **13**, 7777 (2022).

[65] Gradwohl, K.-P. *et al.* Enhanced nanoscale Ge concentration oscillations in Si/SiGe quantum well through controlled segregation. *Nano Lett.* **25**, 4204–4210 (2025).

[66] Albrecht, W., Moers, J. & Hermanns, B. HNF - Helmholtz Nano Facility. *J. Large Scale Res. Facil. (JLSRF)* **3**, A112 (2017).

[67] Volmer, F. *et al.* How to solve problems in micro- and nanofabrication caused by the emission of electrons and charged metal atoms during e-beam evaporation. *Journal of Physics D: Applied Physics* **54**, 225304 (2021).

[68] Klos, J., Hassler, F., Cerfontaine, P., Bluhm, H. & Schreiber, L. R. Calculation of tunnel couplings in open gate-defined disordered quantum dot systems. *Phys. Rev. B* **98**, 155320 (2018).

Supplementary Material to Impact of the local valley splitting on the coherence of conveyor-belt spin shuttling in ^{28}Si/SiGe

Mats Volmer,[1] Tom Struck,[1] Arnau Sala,[1] Jhih-Sian Tu,[2] Stefan Trellenkamp,[2] Davide Degli Esposti,[3] Giordano Scappucci,[3] Łukasz Cywiński,[4] Hendrik Bluhm,[1] and Lars R. Schreiber[1,*]

[1] *JARA-FIT Institute for Quantum Information, Forschungszentrum Jülich GmbH and RWTH Aachen University, Aachen, Germany*
[2] *Helmholtz Nano Facility (HNF), Forschungszentrum Jülich, Jülich, Germany*
[3] *QuTech and Kavli Institute of Nanoscience, Delft University of Technology, Lorentzweg 1, 2628 CJ Delft, The Netherlands*
[4] *Institute of Physics, Polish Academy of Sciences, Warsaw, Poland*

CONTENTS

SUPPLEMENTARY NOTE I. Valley splitting detection vs. initialized valley state 2

SUPPLEMENTARY NOTE II. Optimal visibility of the spin-valley anticrossings 5

SUPPLEMENTARY NOTE III. Raw data for the valley splitting map 5

SUPPLEMENTARY NOTE IV. The time-dependent Hamiltonian for spin and valley degrees of freedom of the shuttled dot 5

SUPPLEMENTARY NOTE V. Unedited version of Figure 3 13

SUPPLEMENTARY NOTE VI. Nonadiabatic valley excitation 14

SUPPLEMENTARY NOTE VII. Dynamics due to shuttling through a spin-valley resonance: the spin-valley flip-flop 16

SUPPLEMENTARY NOTE VIII. Mechanisms of decoherence during the shuttling 18
A. Dephasing in absence of spin-valley resonances and valley excitations 18
B. Dephasing due to valley splitting fluctuations activated by the spin-valley flip-flop 19
C. Spin relaxation near the spin-valley relaxation resonance 21
D. Valley excitation after shuttling through the valley splitting minimum 21

SUPPLEMENTARY NOTE IX. Discussion of experimental results: effect of single there-and-back shuttling through the spin-valley resonance 22

SUPPLEMENTARY NOTE X. Discussion of experimental results: Multiple passages through valley splitting minimum and spin-valley resonances 23
A. Passages through a spin-valley resonance 24
B. Passages through a valley splitting minimum 25

References 29

* lars.schreiber@physik.rwth-aachen.de

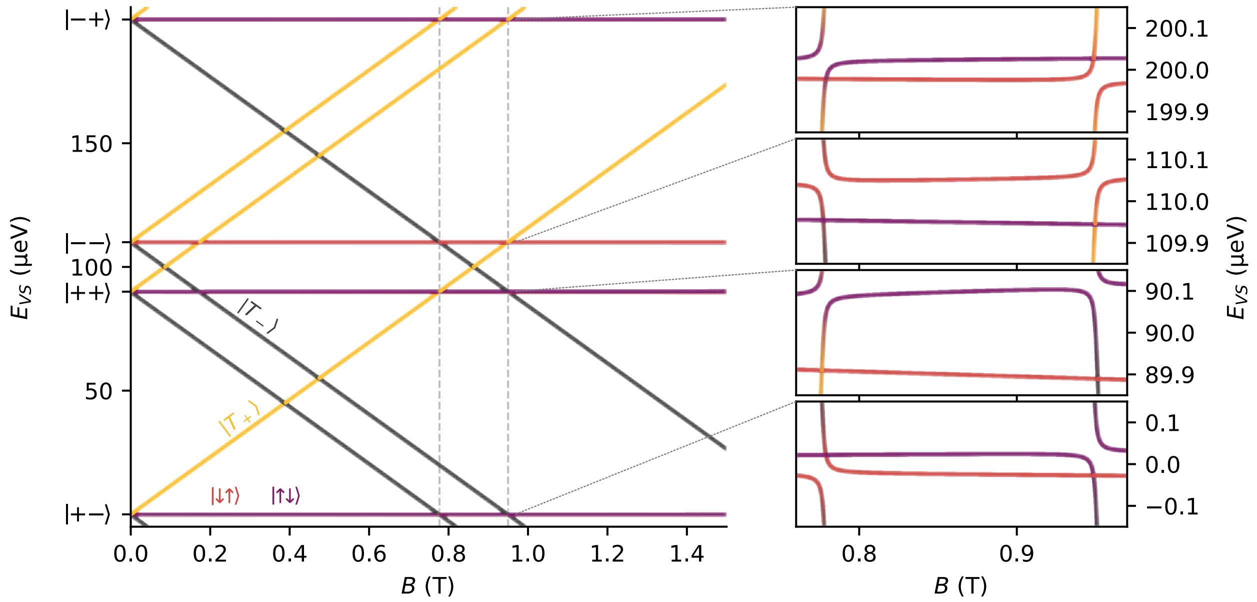

Supplementary Figure 1. Simulated eigenenergies of the full spin and valley degrees of freedom as a function of magnetic field. Insets are included in the vicinity of the anticrossings. Valley states are labelled at the y-axis and spin states are encoded in color.

SUPPLEMENTARY NOTE I: VALLEY SPLITTING DETECTION VS. INITIALIZED VALLEY STATE

In the main text we state that we cannot identify into which specific valley state we initialize the (3,1) charge state in the DQD. For slow enough sweep of interdot detuning, charge transfer from $(4,0)$ state (with both valleys in the L dot fully occupied) to $(3,1)$ state should lead to transfer of one of electrons from the excited valley state in L dot, $|+\rangle_L$, to the ground state in the R dot, $|-\rangle_R$, i.e. creation of S_{+-} singlet with $(3,1)$ charge occupation. This, however, requires the dynamics to be adiabatic with respect to inter-valley tunnel coupling t_{+-} between the respective valley states in the two dots. If this coupling is small, and the intra-valley tunnel couplings, t_{++} and t_{--}, are larger than the inter-valley one, for given detuning sweep rate the charge transfer between $|+\rangle_L$ and $|-\rangle_R$ states might fail (completely, or partially, i.e. the S_{+-} state will be created with proability < 1), while creation of either S_{++} or S_{--} state will occur with high probability due to adiabaticity of dynamics involving the intra-valley tunneling. Full discussion of implications of such nonadiabatic charge and valley dynamics during $(4,0) \rightarrow (3,1)$ transition is given in [1]. Here we focus on showing that the key physical effect underpinning the method of valley splitting mapping used in the main manuscript - a strong renormalizaiton of singlet-triple presession frequency near spin-valley resonances in both dots - occurs independently of the valley occupation pattern of the initialized singlet (or a statistical mixture of singlets).

We focus on $(3,1)$ states, $|v_L v_R \sigma_L \sigma_R\rangle$, labeling them with $v_L = \pm$ (single-occupied valley state in dot L), $v_R = \pm$ (valley state in dot R occupied by the signle electron in that dot), and $\sigma_D =\uparrow / \downarrow$ labeling the projections on the quantization axis of the spins of the electrons in the singly-occupied valley states. The Hamiltonian is defined on a 16-dimensional Hilbert space spanned by the basis $\mathcal{B}$, ordered energetically into four distinct valley manifolds:

$$\begin{aligned}\mathcal{B} &= \{|v_L v_R\rangle \otimes |s_L s_R\rangle\} \\ &= \{\underbrace{|+-\downarrow\downarrow\rangle, \dots,}_{\text{Ground }(H_{+-})} \underbrace{|++\downarrow\downarrow\rangle, \dots,}_{\text{R-Excited }(H_{++})} \underbrace{|--\downarrow\downarrow\rangle, \dots,}_{\text{L-Excited }(H_{--})} \underbrace{|-+\downarrow\downarrow\rangle, \dots}_{\text{Dual-Excited }(H_{-+})} \}^T\end{aligned} \tag{S1}$$

Within each valley block, the spin basis follows the order $\{\downarrow\downarrow, \downarrow\uparrow, \uparrow\downarrow, \uparrow\uparrow\}$. The full Hamiltonian H is represented in

block-matrix form as:

$$H = \begin{pmatrix} H_{+-} & T_R & T_L & 0 \\ T_R^\dagger & H_{++} & 0 & T'_L \\ T_L^\dagger & 0 & H_{--} & T'_R \\ 0 & {T'_L}^\dagger & {T'_R}^\dagger & H_{-+} \end{pmatrix} \tag{S2}$$

The diagonal blocks describe the magnetic field-dependent Zeeman energy and the static valley splitting offsets. For a specific block with valley configuration (v_L, v_R), the Hamiltonian is diagonal in the spin subspace:

$$H_{v_L v_R} = \mathrm{diag}\,(E_{\downarrow\downarrow}, E_{\downarrow\uparrow}, E_{\uparrow\downarrow}, E_{\uparrow\uparrow}) + E_{\text{offset}} \cdot \mathbb{1}_4 \tag{S3}$$

The spin energies are given by $E_{s_L s_R} = \frac{\mu_B B}{2}(g_L s_L + g_R s_R)$. The valley offsets are defined relative to the ground state: zero for H_{+-}, $E_{VS,r}$ for H_{++}, $E_{VS,l}$ for H_{--}, and $E_{VS,l} + E_{VS,r}$ for H_{-+}.

The off-diagonal matrices describe the spin-valley mixing only on the same electron. We exclude cases where a spin flip on the left electron coincides with a valley flip on the right electron. We formally distinguish between transitions originating from the ground valley state (T) and those originating from an already excited valley state (T').

The right-dot coupling T_R connects the ground state ($|+-\rangle$) to the right-excited state ($|++\rangle$). The matrix T'_R describes the same right-dot transition but starting from the left-excited configuration ($|--\rangle \leftrightarrow |-+\rangle$). In this model, we assume $T_R = T'_R$, with the matrix form:

$$T_R = \begin{pmatrix} 0 & 0 & 0 & 0 \\ \Delta_{sv,R} & 0 & 0 & 0 \\ 0 & 0 & 0 & 0 \\ 0 & 0 & \Delta_{sv,R} & 0 \end{pmatrix} \tag{S4}$$

This interaction strictly preserves the left spin state while flipping the right spin and valley indices.

Similarly, T_L and T'_L describe the left-dot valley transition. T_L couples the ground state to the left-excited state ($|+-\rangle \leftrightarrow |--\rangle$), while T'_L couples the right-excited state to the dual-excited state ($|++\rangle \leftrightarrow |-+\rangle$). Assuming $T_L = T'_L$, the coupling matrix is:

$$T_L = \begin{pmatrix} 0 & 0 & 0 & 0 \\ 0 & 0 & 0 & 0 \\ \Delta_{sv,L} & 0 & 0 & 0 \\ 0 & \Delta_{sv,L} & 0 & 0 \end{pmatrix} \tag{S5}$$

This interaction preserves the right spin state while coupling the left spin and valley indices. Using realistic parameters [2] in table I, we arrive at the eigenenergies plotted in Fig. 1. We find that independent of which valley state was initialized, the anticrossings with other valley states always appear at the same magnetic fields (see insets in Fig. 1) which correspond to the valley splitting energies. The positions are precise up to the relative variation of the g-factors, so the systematic uncertainty is in the permille order of magnitude.

TABLE I. Parameters utilized in the numerical diagonalization. The g-factors $g_{l,0}$ and $g_{r,0}$ correspond to the ground state valley configuration $(L+, R-)$.

Parameter	Symbol	Value	Unit
Valley Splitting			
Left QD	$E_{VS,l}$	110	μeV
Right QD	$E_{VS,r}$	90	μeV
g-factors			
Left (Ground / Excited)	$g_{l,0}$ / $g_{l,1}$	2.002 / 1.999	-
Right (Ground / Excited)	$g_{r,0}$ / $g_{r,1}$	2.001 / 1.998	-
Spin-Valley Coupling			
Spin-Valley Mixing (R)	$\Delta_{sv,R}$	100	neV
Spin-Valley Mixing (L)	$\Delta_{sv,L}$	100	neV

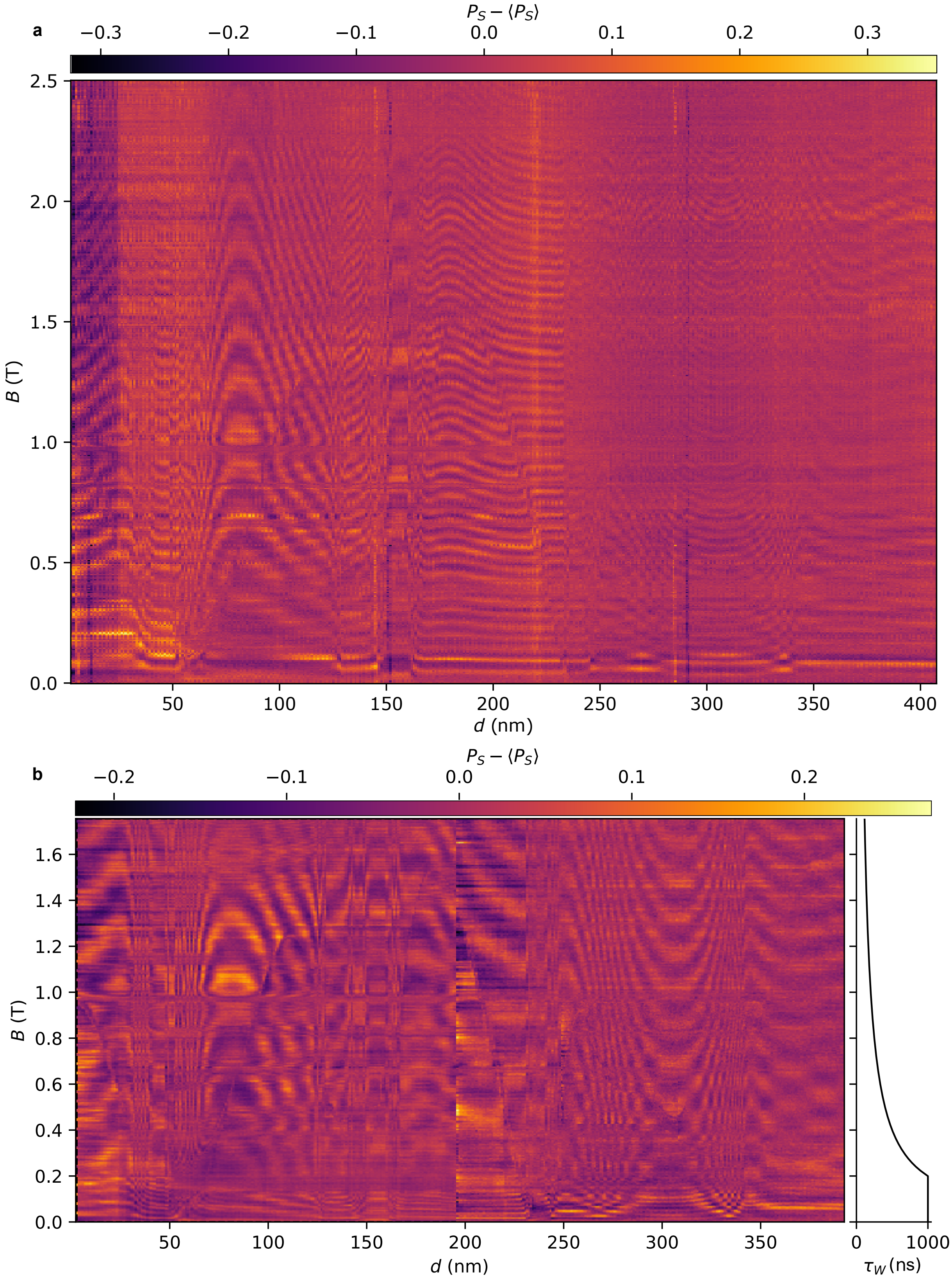

Supplementary Figure 2. Optimal visibility of the spin-valley anticrossings. (a) Valley Splitting map with a constant wait time of 300 ns within the shuttler. (b) Valley Splitting map with varying wait time as a function of magnetic field.

SUPPLEMENTARY NOTE II: OPTIMAL VISIBILITY OF THE SPIN-VALLEY ANTICROSSINGS

In the main text we state that the the wait time is adapted to the external magnetic field in order to identify the spin-valley resonance for valley-splitting mapping. Here, we support this statement by comparing the valley splitting map recorded with a constant wait time of 300 ns (Supplementary Fig. 2a) and one recorded with wait time partially being anti-proportional to the magnetic field (Supplementary Fig.2b). The phase accumulated during the wait time scales linearly with the magnetic field, hence we choose a wait time function that scales with the inverse magnetic field. A function that allowed sensible values across the full range of magnetic fields is

$$\tau_W(B) = \min(1000\,\mathrm{ns}, 200\,\mathrm{ns\,T}/B). \tag{S6}$$

In order to avoid the divergence at zero field, we limit the wait time to 1000 ns. Thus, the wait time below 200 mT is constant so that the background oscillations remain. Everywhere else, the oscillations are damped such that the valley splitting line is more clearly isolated. For some traces, this reduces the background so much that there is almost no oscillation left.

SUPPLEMENTARY NOTE III: RAW DATA FOR THE VALLEY SPLITTING MAP

In this section, we display the raw data for the valley splitting map. In more than thirty high resolution measurements, we captured this complete valley splitting map. Here, we display all of them with the following optimization of the visibility. We always subtract the linewise mean to align the data and subsequently cut the color scale from the 5% to the 95% quantile in order to eliminate outliers that distract the color scale. Further improvements of the visibility are due to the diverse background scenarios unattainable. All figures firstly display a full plot of the trace with dashed areas indicating the individual measurements. The subsequent two plots are the large left half and right half of the measurement. Subsequently, we provide the detailed plot in ascending order of d. All plots are equipped with manual guides to the eye for the found valley splitting curve. These plots are shown in Fig. 3 through Fig. 9

SUPPLEMENTARY NOTE IV: THE TIME-DEPENDENT HAMILTONIAN FOR SPIN AND VALLEY DEGREES OF FREEDOM OF THE SHUTTLED DOT

The Hamiltonian describing the coupling between the $|\pm k_z\rangle$ valleys for an electron strongly confined in the z-direction, the spatial wavefunction of which is time-dependent is given by

$$\hat{H}_v(t) = \begin{pmatrix} 0 & \Delta^*(t) \\ \Delta(t) & 0 \end{pmatrix} = |\Delta(t)| \left[\cos\phi(t)\hat{\tau}_x + \sin\phi(t)\hat{\tau}_y\right] \ , \tag{S7}$$

in which we have written the real and imaginary part of the valley coupling $\Delta(t)$ as $\Delta_R(t) = |\Delta(t)|\cos\phi(t)$ and $\Delta_I(t) = |\Delta(t)|\sin\phi(t)$. The time dependence of the valley coupling $\Delta(t)$ follows from the time dependence of the envelope wavefunction of the electron. Under an assumption that the shuttling of the electron leads to a shift of the mean position of the electron, $x(t)$, with possible deformations of the shape of the envelope wavefunction neglected, the time dependence of this coupling simply follows from spatial dependence of the coupling when a QD of fixed shape is positioned at $x(t)$, i.e. $\Delta(t) = \Delta[x(t)]$, and the wavefunction is overlapping with a particular realization of atomic disorder (positions of Si and Ge atoms) at the Si/SiGe interface. Examples of trajectories of $\Delta(x)$ can be found e.g. in [3, 4], and in Sec. SUPPLEMENTARY NOTE VI.

In order to see how time dependence of $\Delta[x(t)]$ activated by shuttling of the dot leads to valley excitations we use the basis of instantaneous valley eigenstates $|\pm(t)\rangle$ that fulfil $\hat{H}_v(t)\,|\pm(t)\rangle = \pm|\Delta(t)|\,|\pm(t)\rangle$. We assume that at the initial time t_0 the electron is in one of the valley eigenstates, $|\pm(t_0)\rangle$. After introducing the unitary transformation between the eigenstates at initial time t_0 and later time t defined by $|\pm(t)\rangle = \hat{U}(t,t_0)\,|\pm(t_0)\rangle$ we arrive at the effective Hamiltonian $\tilde{H}_v(t) = \hat{U}\hat{H}(t)\hat{U}^\dagger + i\hbar(\mathrm{d}\hat{U}/\mathrm{dt})\hat{U}^\dagger$ that in the basis of $\{|-(t)\rangle, |+(t)\rangle\}$ instantaneous valley eigenstates reads

$$\tilde{H}_v(t) = \begin{pmatrix} -|\Delta(t)| & -\frac{\hbar}{2}\dot{\phi}(t) & , \\ -\frac{\hbar}{2}\dot{\phi}(t) & |\Delta(t)| & \end{pmatrix} \tag{S8}$$

where $\dot{\phi}(t) = \mathrm{d}\phi(t)/\mathrm{dt}$. Transitions between the instantaneous eigenstates become significant when $\dot{\phi}$ becomes comparable to $|\Delta|$: if we assume a constant $\dot{\phi} \equiv \omega$, and a constant $|\Delta|$ (so that the trajectory of $\Delta(t)$ corresponds to a circle

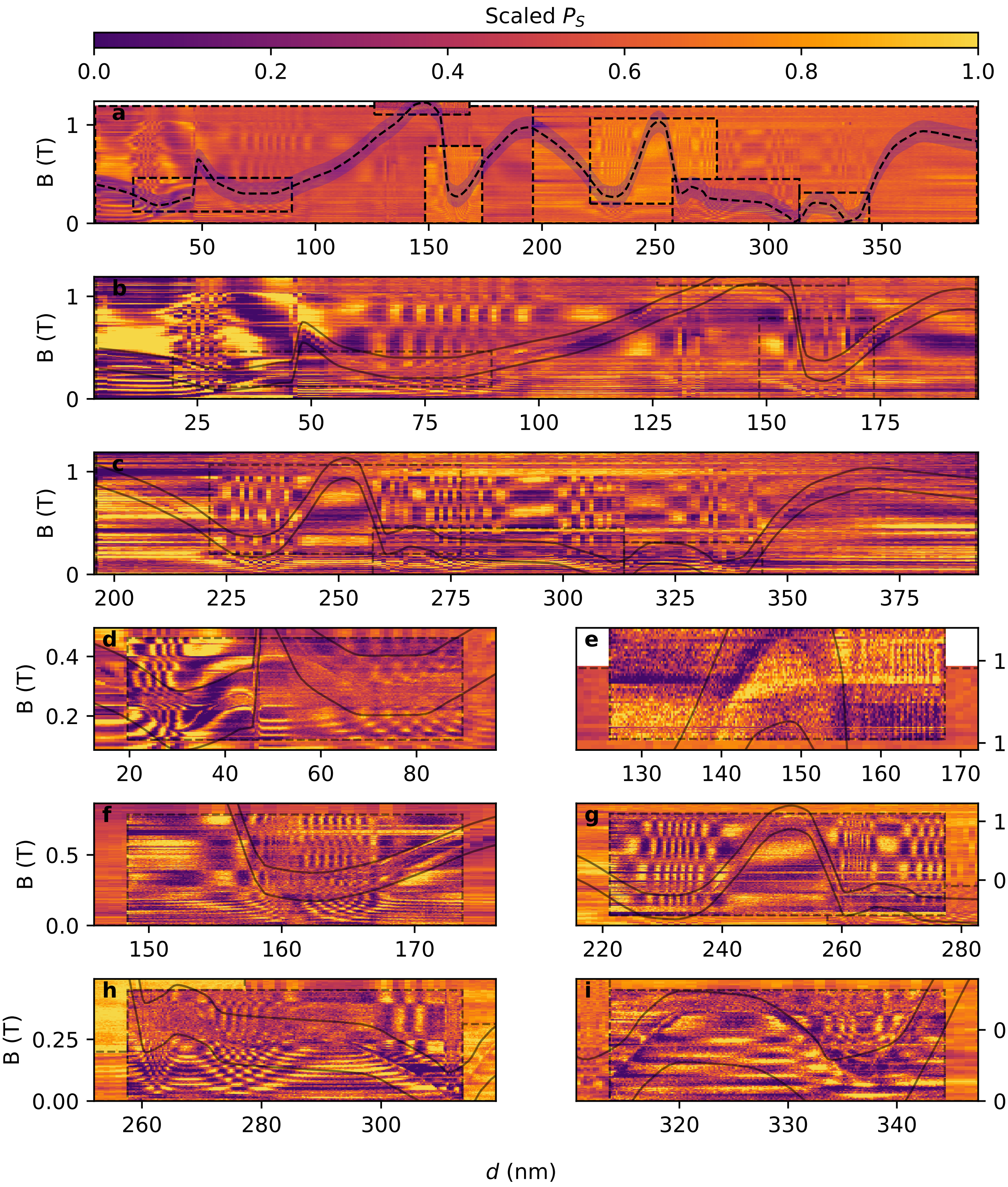

Supplementary Figure 3. Raw data for applying a voltage of $V_{\mathrm{ST}} = 0.3\,\mathrm{V}$ and $V_{\mathrm{SB}} = 0\,\mathrm{V}$ leading to a coordinate of $y = 18\,\mathrm{nm}$ with native contrast. Guides to the eye are added at $\pm 100\,\mathrm{mT}$ with respect to the spline position in panels b-i.(a) Complete Map composed of eight different measurements. (b) Broad measurement of the first 196 nm. (c) Broad measurement of the second 196 nm. (d-i) Fine measurements ordered from left to right. Two offset copies of the spline fit are added as a guide to the eye.

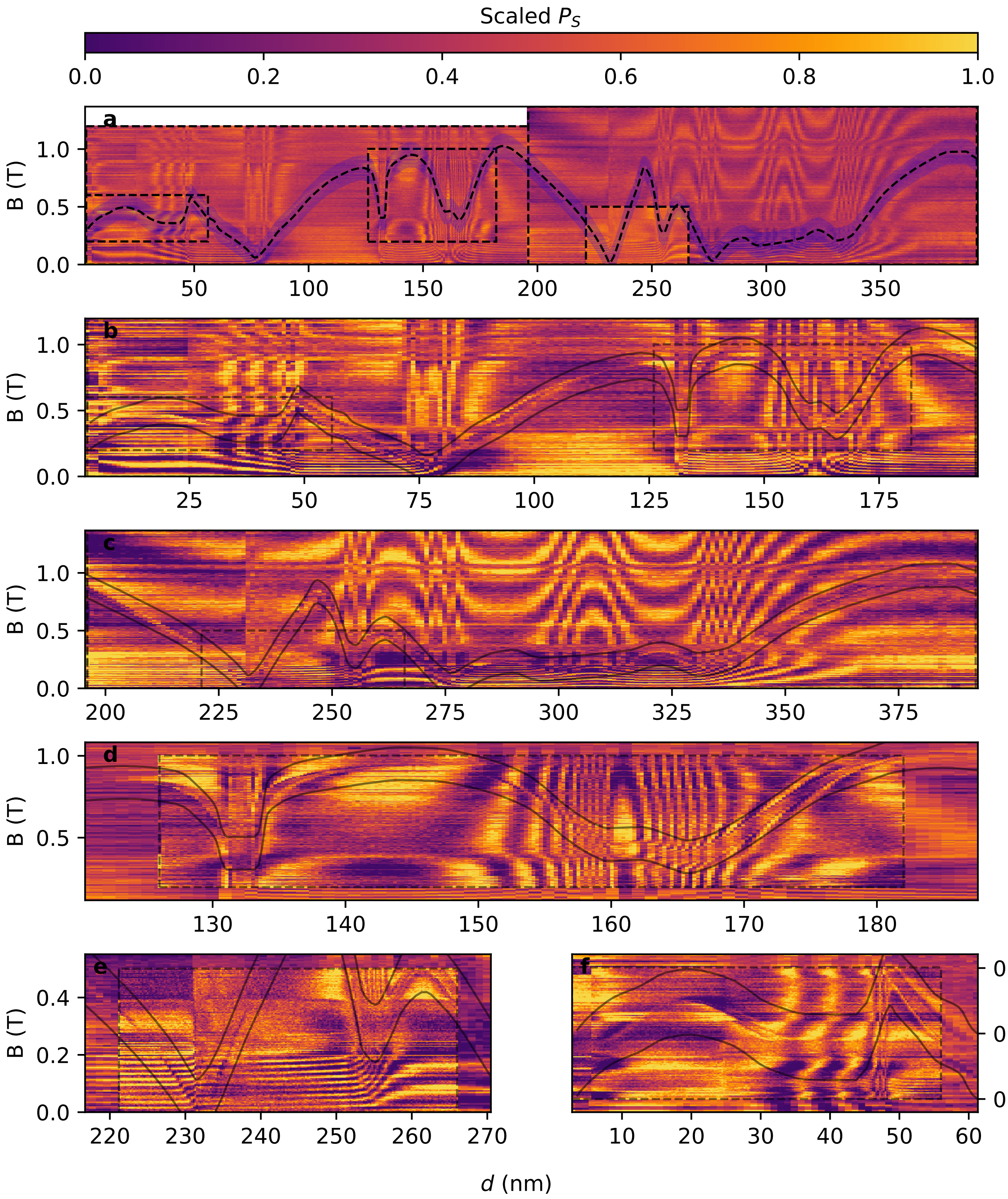

Supplementary Figure 4. Raw data for applying a voltage of $V_{ST} = 0.25\,V$ and $V_{SB} = 0.05\,V$ leading to a coordinate of $y = 12\,nm$ with native contrast. Guides to the eye are added at $\pm 100\,mT$ with respect to the spline position in panels b-f.(a) Complete Map composed of five different measurements. (b) Broad measurement of the first 196 nm. (c) Broad measurement of the second 196 nm. (d) Largest fine measurement at around 150 nm. (e-f) Fine measurements ordered from left to right. Two offset copies of the spline fit are added as a guide to the eye.

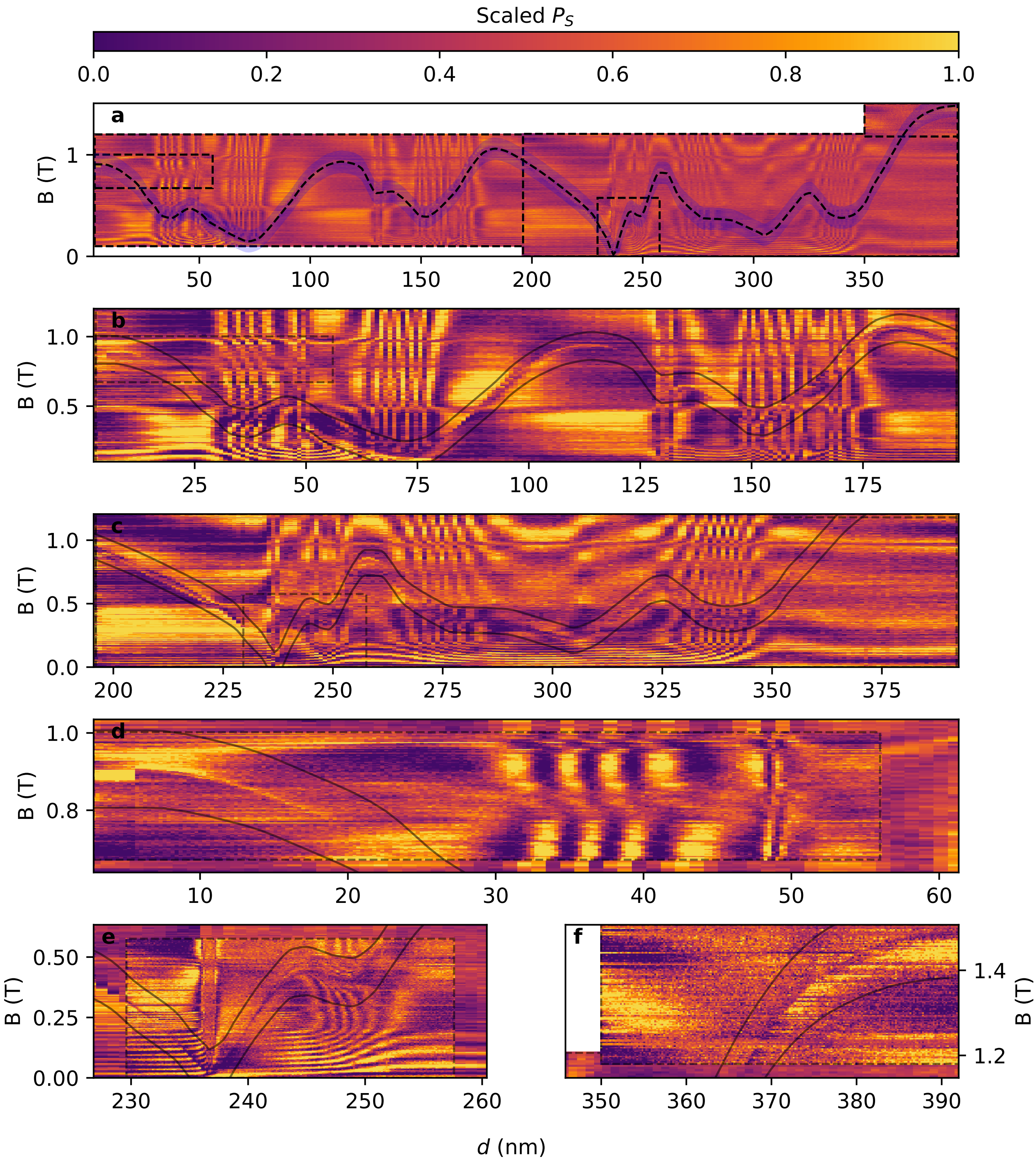

Supplementary Figure 5. Raw data for applying a voltage of $V_{\mathrm{ST}} = 0.2\,\mathrm{V}$ and $V_{\mathrm{SB}} = 0.1\,\mathrm{V}$ leading to a coordinate of $y = 6\,\mathrm{nm}$ with native contrast. Guides to the eye are added at $\pm 100\,\mathrm{mT}$ with respect to the spline position in panels b-f.(a) Complete Map composed of five different measurements. (b) Broad measurement of the first 196 nm. (c) Broad measurement of the second 196 nm. (d) Largest fine measurement at around 30 nm. (e-f) Fine measurements ordered from left to right. Two offset copies of the spline fit are added as a guide to the eye.

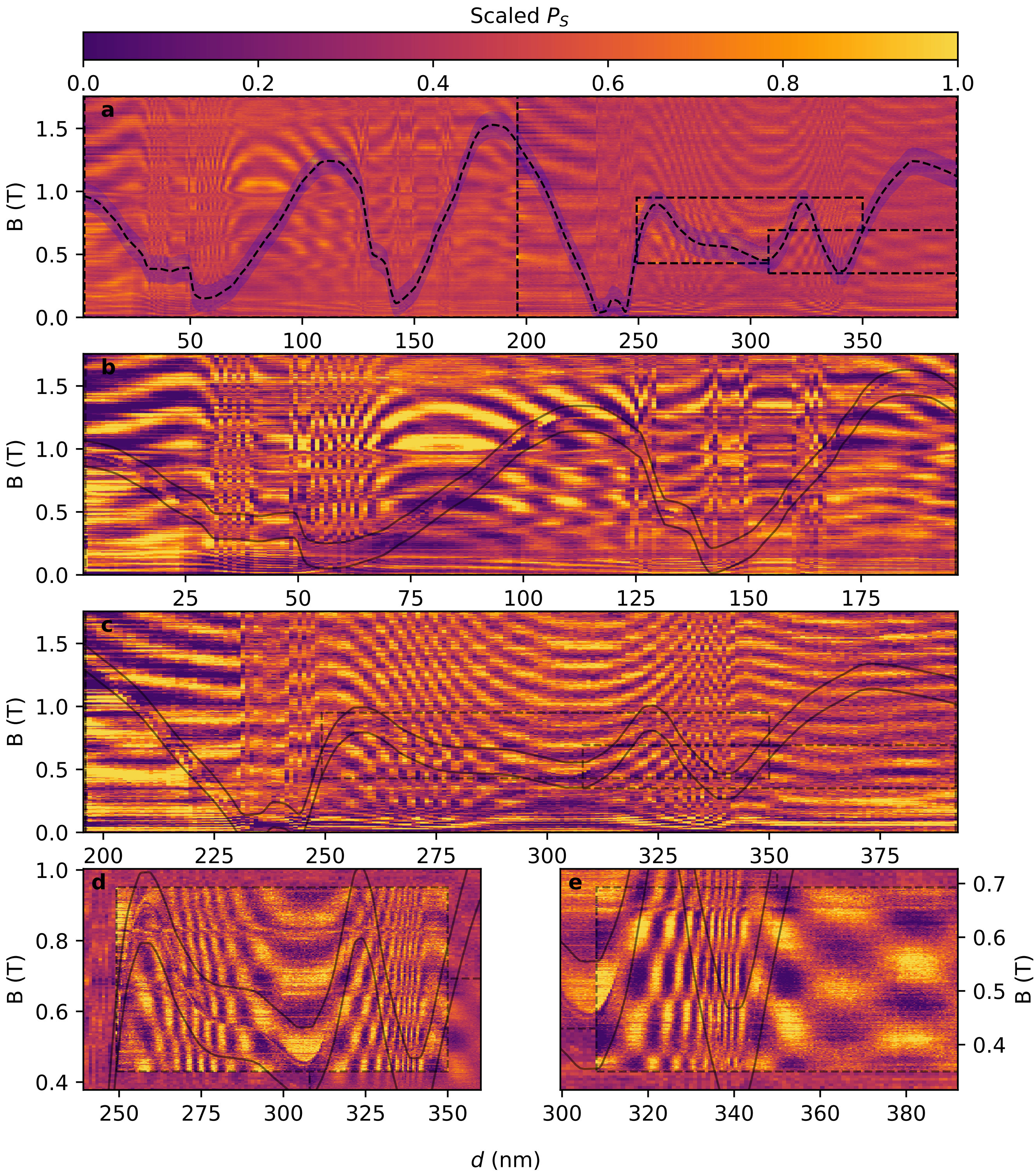

Supplementary Figure 6. Raw data for applying a voltage of $V_{\mathrm{ST}} = 0.15\,\mathrm{V}$ and $V_{\mathrm{SB}} = 0.15\,\mathrm{V}$ leading to a coordinate of $y = 0\,\mathrm{nm}$ with native contrast. Guides to the eye are added at $\pm 100\,\mathrm{mT}$ with respect to the spline position in panels b-e.(a) Complete Map composed of four different measurements. (b) Broad measurement of the first 196 nm. (c) Broad measurement of the second 196 nm. (d-e) Fine measurements ordered from left to right. Two offset copies of the spline fit are added as a guide to the eye.

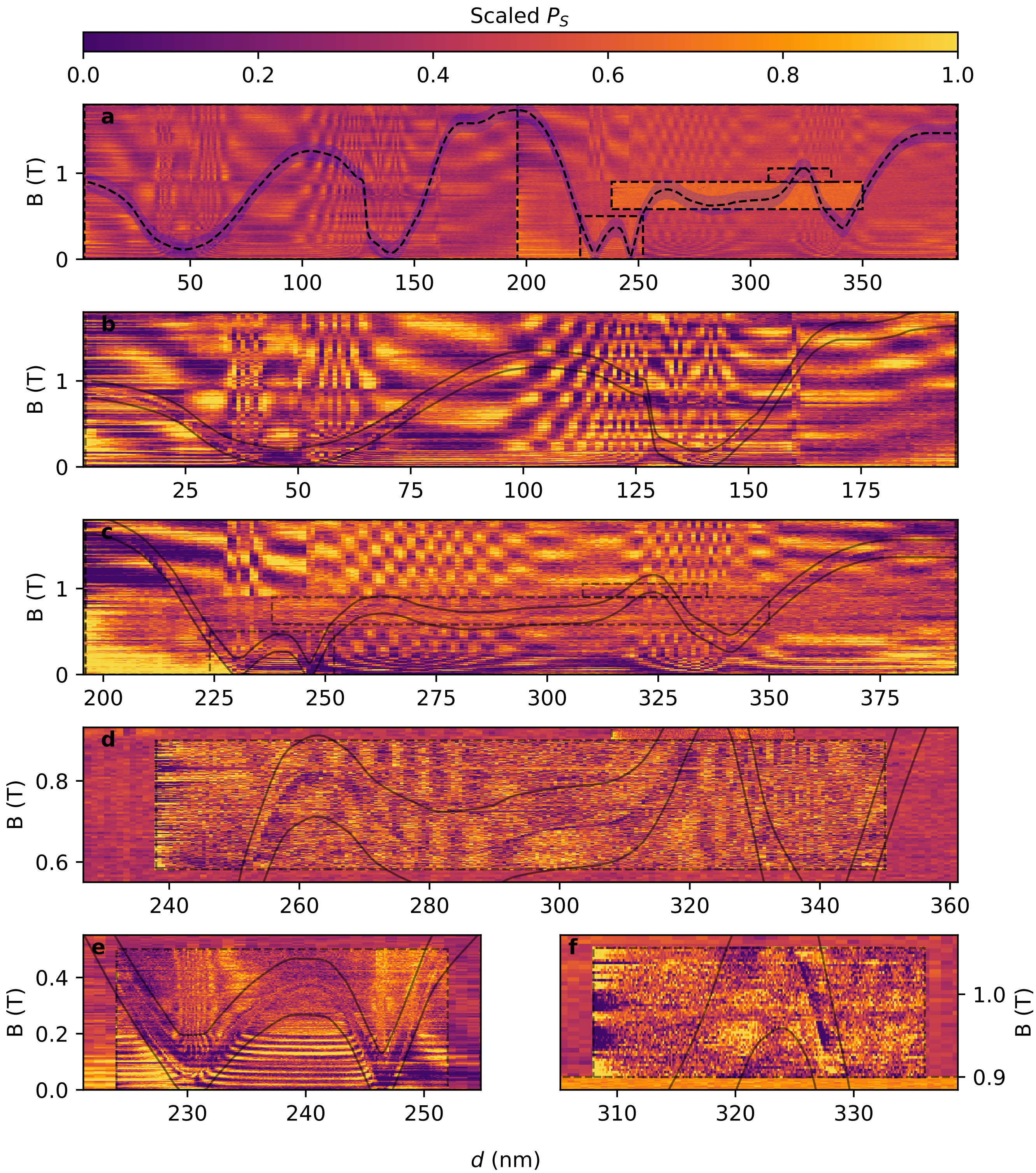

Supplementary Figure 7. Raw data for applying a voltage of $V_{ST} = 0.1\,V$ and $V_{SB} = 0.2\,V$ leading to a coordinate of $y = -6\,nm$ with native contrast. Guides to the eye are added at $\pm 100\,mT$ with respect to the spline position in panels b-f.(a) Complete Map composed of five different measurements. (b) Broad measurement of the first 196 nm. (c) Broad measurement of the second 196 nm. (d) Largest fine measurement at around 300 nm. (e-f) Fine measurements ordered from left to right. Two offset copies of the spline fit are added as a guide to the eye.

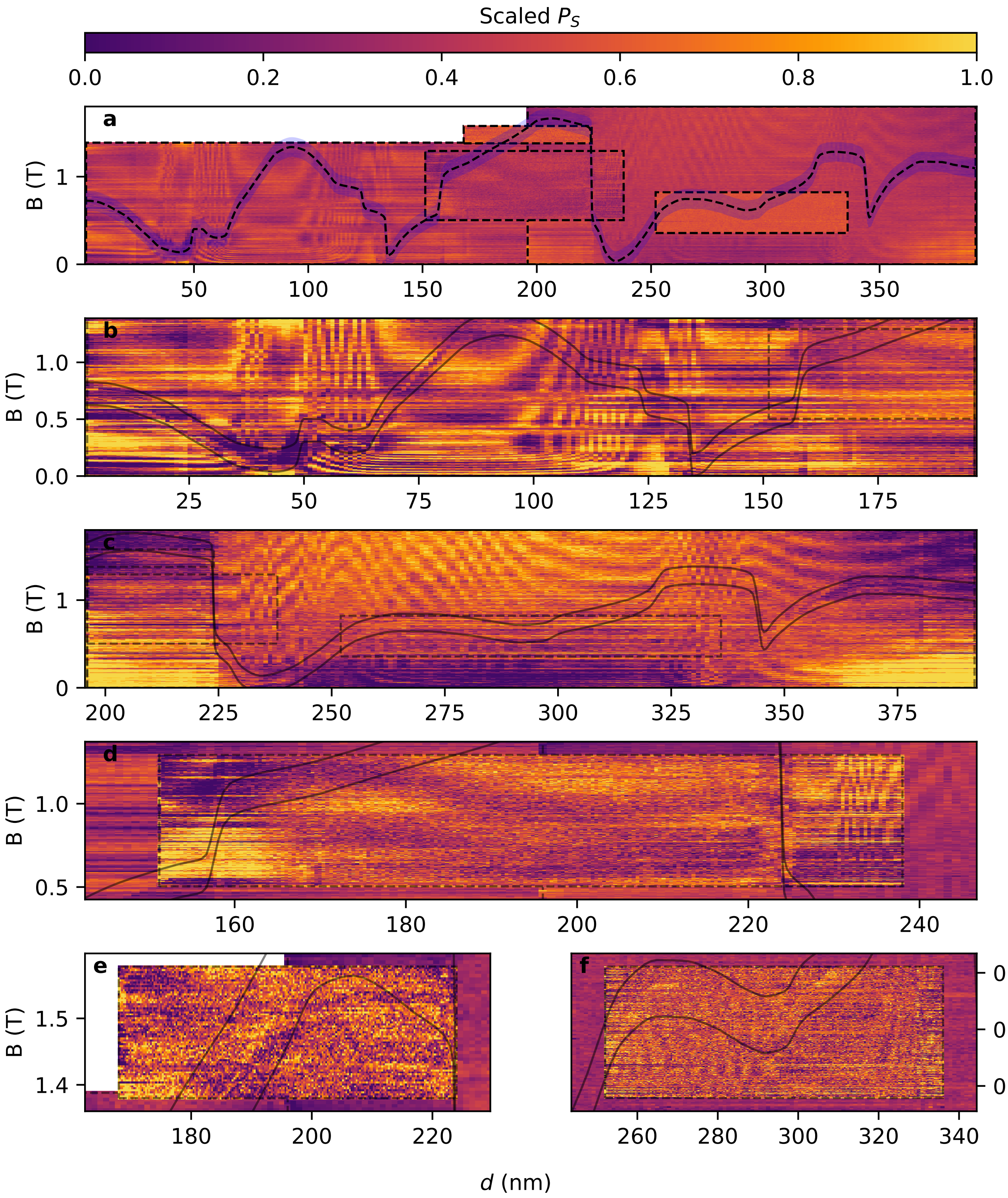

Supplementary Figure 8. Raw data for applying a voltage of $V_{\mathrm{ST}} = 0.05\,\mathrm{V}$ and $V_{\mathrm{SB}} = 0.25\,\mathrm{V}$ leading to a coordinate of $y = -12\,\mathrm{nm}$ with native contrast. Guides to the eye are added at $\pm 100\,\mathrm{mT}$ with respect to the spline position in panels b-f.(a) Complete Map composed of five different measurements. (b) Broad measurement of the first 196 nm. (c) Broad measurement of the second 196 nm. (d) Largest fine measurement at around 200 nm. (e-f) Fine measurements ordered from left to right. Two offset copies of the spline fit are added as a guide to the eye.

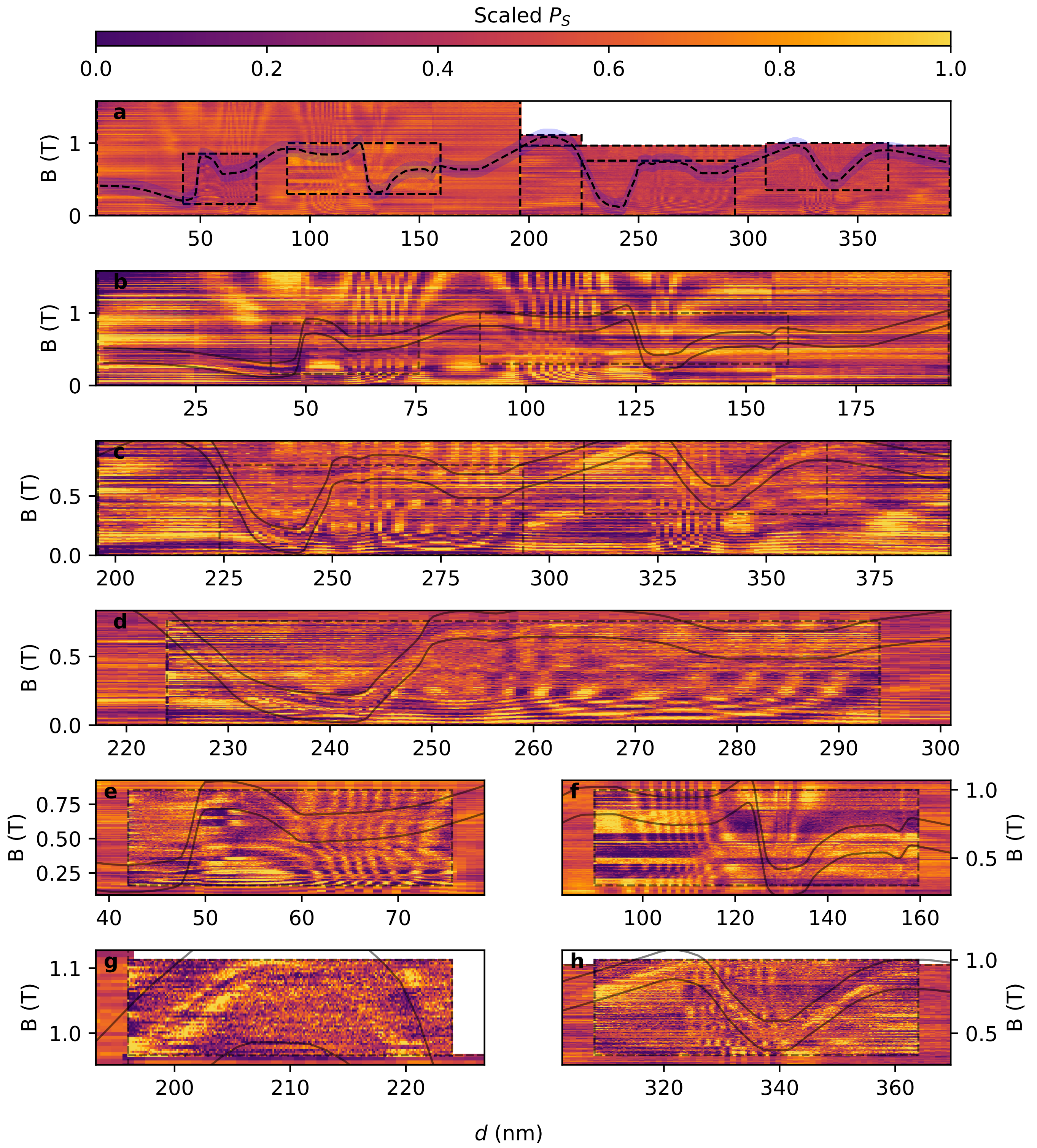

Supplementary Figure 9. Raw data for applying a voltage of $V_{\mathrm{ST}} = 0\,\mathrm{V}$ and $V_{\mathrm{SB}} = 0.3\,\mathrm{V}$ leading to a coordinate of $y = -18\,\mathrm{nm}$ with native contrast. Guides to the eye are added at $\pm 100\,\mathrm{mT}$ with respect to the spline position in panels b-h.(a) Complete Map composed of seven different measurements. (b) Broad measurement of the first 196 nm. (c) Broad measurement of the second 196 nm. (d) Largest fine measurement at around 260 nm. (e-h) Fine measurements ordered from left to right. Two offset copies of the spline fit are added as a guide to the eye.

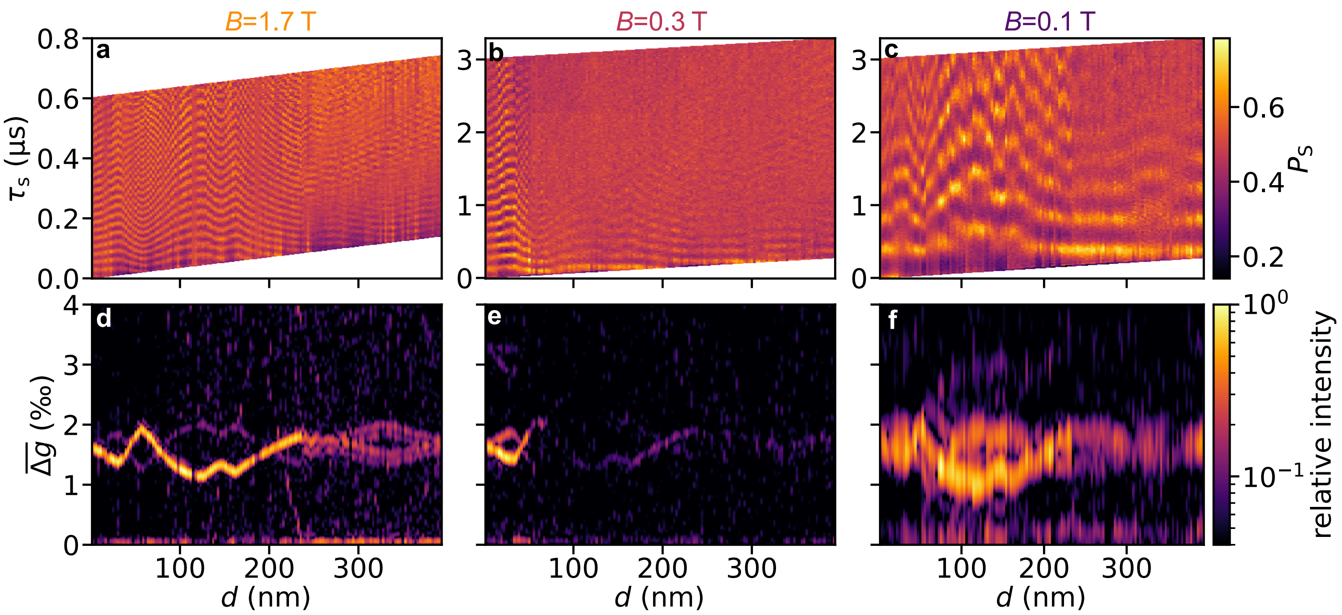

Supplementary Figure 10. Figure 3 from the main text without guides to the eye. Panels (a-f) relate to panels (b-g) of Figure 3 from the main text.

in the complex plane), the maximum occupation of the valley state that is not adiabatically connected to the initial one is

$$Q_{v,max} = \frac{(\hbar\omega)^2}{4|\Delta|^2 + (\hbar\omega)^2} \ , \tag{S9}$$

which is $\approx |\hbar\omega/2\Delta|^2$ when $|\Delta| \gg \hbar\omega$.

It is well established that interplay of interface roughness and spin-orbit interaction causes the g-factor of electron's spin to depend on the valley Hamiltonian eigenstate [5, 6], and it leads to an appearance of finite spin-valley coupling matrix elements connecting $|-\uparrow\rangle$ to $|+\downarrow\rangle$ and $|+\uparrow\rangle$ to $|-\downarrow\rangle$ states [7, 8]. After including these terms we arrive at the following time-dependent Hamiltonian acting in the four-dimensional subspace of lowest energy spin/valley states of the shuttled dot, $\{|-\uparrow(t)\rangle, |-\downarrow(t)\rangle, |+\uparrow(t)\rangle, |+\downarrow(t)\rangle\}$,

$$\tilde{H}(t) = \begin{pmatrix} \frac{1}{2}[E_Z^- - E_{\rm VS}] & 0 & -\frac{\hbar}{2}\dot{\phi} & \Delta_{sv} \\ 0 & \frac{1}{2}[-E_Z^- - E_{\rm VS}] & \Delta'_{sv} & -\frac{\hbar}{2}\dot{\phi} \\ -\frac{\hbar}{2}\dot{\phi} & \Delta'^*_{sv} & \frac{1}{2}[E_Z^+ + E_{\rm VS}] & 0 \\ \Delta^*_{sv} & -\frac{\hbar}{2}\dot{\phi} & 0 & \frac{1}{2}[-E_Z^+ + E_{\rm VS}] \end{pmatrix} . \tag{S10}$$

In the above Hamiltonian the Zeeman splitting in $\pm$ valley, $E_Z^{\pm} = g_{\pm}\mu_B B$, the valley splitting $E_{\rm VS} = 2|\Delta|$, spin-valley couplings Δ_{sv} and Δ'_{sv}, and valley phase ϕ are all implicitly time-dependent.

The shuttled electron could be initialized in either $|-\rangle$ or $|+\rangle$ valley state. For simplicity of presentation let us assume that it is initialized in the lower energy valley state, $|+\rangle$, as most of discussion below applies after changing the initial state to $|-\rangle$. Whenever the initial valley state of the electron matters for the dynamics, we will make an appropriate remark.

SUPPLEMENTARY NOTE V: UNEDITED VERSION OF FIGURE 3

In this section, we show a plot of an unedited version of Figure 3 from the main text without arrows and lines (Supplementary Figure 10)

SUPPLEMENTARY NOTE VI: NONADIABATIC VALLEY EXCITATION

When spin-valley coupling is negligible, or the Zeeman splitting and $E_{\rm VS}(x)$ profile along the shuttling path are such that no spin-valley resonances are encountered, or v_S is large enough to passage through these resonances is very close to being diabatic, the Hamiltonian (S10) can be treated as sum of two Hamiltonians, $\hat{H}_\uparrow(t) + \hat{H}_\downarrow(t)$, each acting in subspace of fixed spin:

$$\hat{H}_\uparrow(t) = \begin{pmatrix} \frac{1}{2}E_Z^-(t) & 0 \\ 0 & \frac{1}{2}E_Z^+(t) \end{pmatrix} + \tilde{H}_v(t) \, ,$$
$$\hat{H}_\downarrow(t) = \begin{pmatrix} -\frac{1}{2}E_Z^-(t) & 0 \\ 0 & -\frac{1}{2}E_Z^+(t) \end{pmatrix} + \tilde{H}_v(t) \, , \tag{S11}$$

in which $\hat{H}_v$ is given by Eq. (S8). Let us define the difference of Zeeman splittings for the shuttled electron in the two valley states:

$$\delta E_{Z,R} \equiv E_Z^+ - E_Z^- \, . \tag{S12}$$

When $E_{\rm VS} \gg \frac{1}{2}|\delta E_{Z,R}|$, we can neglect the difference of Zeeman splittings in two valleys when considering nontrivial (i.e. valley-changing) dynamics due to $\hat{H}_v(t)$. Note that at highest considered magnetic fields of about 1 Tesla, and for a large value of intervalley g-factor difference, $\delta g \equiv g_+ - g_- \approx 10^{-3}$, we have $|\delta E_{Z,R}| \approx 0.1$ μeV, which is an order of magnitude smaller than the lowest measured $E_{\rm VS}$. We can thus safely assume that even at points of low $E_{\rm VS}$, at which $\dot{\phi}$ could cause intervalley transitions, the dynamics caused by this term is to a very good approximation the same for both spin directions. We will also assume that the valley dynamics caused by $\tilde{H}_v(t)$ occurs in windows of time (narrow regions of x) that are narrow enough to allow for treating the dynamics as impulse-like from the point of view of spin evolution, i.e. the passage of the qubit through a region of small $E_{\rm VS}$ and large $\dot{\phi}$ amounts to the following state transformation:

$$\frac{1}{\sqrt{2}}(|-\uparrow\rangle + e^{i\alpha}|-\downarrow\rangle) \longrightarrow \frac{1}{\sqrt{2}}(a_-|-\rangle + a_+|+\rangle) \otimes (|\uparrow\rangle + e^{i\alpha}|\downarrow\rangle) \, , \tag{S13}$$

in which we have assumed, without any loss of generality, that an equal spin superposition is being shuttled, and α is the relative phase between two amplitudes of the spin qubit at the moment of entering the narrow region of valley excitation. The probability of valley excitation is then given by $Q_v \equiv |a_+|^2$.

Let us estimate when the valley excitations probability can become significant. According to the current understanding of influence of atomistic disorder at Si/SiGe interface on the valley coupling, the real and imaginary components of the coupling are independent Gaussian random fields [3, 9–11], each having autocorrelation length of the order of the spatial extent of the orbital ground state wavefunction, and statistical properties of values $E_{\rm VS}$ encountered over a large enough area are very well captured by the Rice probability distribution, $P(E_{\rm VS}) = \frac{E_{\rm VS}}{\sigma^2}\exp\left(-(E_{\rm VS}^2+\gamma^2)/2\sigma^2\right) I_0(E_{\rm VS}\gamma/\sigma^2)$, where $I_0(z)$ is the modified Bessel function of the first kind. As discussed in the main text, in the measured sample we have $\sigma \approx 61$ μeV and $\gamma < 1$ μeV, so we are in the "disordered" regime in which $\sigma \gg \gamma$. The typical change of magnitude of both real and imaginary component of Δ when the dot is shuttled by a distance larger than twice the in-plane confinement length (about 30 nm) is then $\approx \sqrt{\pi}\sigma$ [10]. Using the data from Fig. 2 in the main text we arrive at an estimate that a typical distance $|\Delta(x_1) - \Delta(x_2)|$ in the complex plane of valley coupling for $|x_2 - x_1| \approx 30$ nm is ≈ 100 μeV, and consequently an estimate for typical magnitudes of derivatives $v_\Delta \equiv |{\rm d}\Delta_{R/I}/{\rm d}x|$ is $v_\Delta \sim 3$ μeV/nm.

For given $|\Delta|$ the maximal value of $\dot{\phi}$ is obtained when the $\Delta(x)$ trajectory locally corresponds to motion in a direction that is tangent to a circle of constant $|\Delta|$. Using the v_Δ rate of change of Δ due to change of x introduced above, we see that $|\Delta||\dot{\phi}| \sim v_\Delta v_S$, where v_S is the shuttling velocity, and consequently $|\dot{\phi}| \sim v_\Delta v_S/|\Delta|$. Small values of $|\Delta|$ are thus necessary for sizable nonadiabaticity of evolution described by Hamiltonian (S8). For a given trajectory of $\Delta(t)$ in the complex plane, the valley excitations will occur most probably when the shuttled dot passes through local minima of $E_{\rm VS}(x) = 2|\Delta(x)|$. Using $v_\Delta \approx 3$ μeV/nm and $v_S \approx 1$ m/s, we can expect significant effect of valley excitation only when the minimal $|\Delta| < 10$ μeV.

In order to verify the accuracy of the above estimates, and to gain more insight into valley excitation probability caused by fast shuttling through $E_{\rm VS}$ minima, we have numerically generated landscape of $\Delta(x)$ using the statistical description for random fields ${\rm Re}\Delta(x)$ and ${\rm Im}\Delta(x)$ given in [3, 10]. In Figure 11 we show an example of trajectory of $\Delta(x)$ for $x \in [0, 700]$ nm, and the corresponding $E_{\rm VS}(x)$. Six minima of valley splitting with $E_{\rm VS} < 25$ μeV are marked with boxes. The $\Delta(x)$ trajectories near each minimum are shown in Fig. 12.

Before we discuss the calculation of probability of valley excitation during shuttling through these minima, let us comment on the relationship between the dynamics following from $\hat{H}_v(t)$, and the one described by the Landau-Zener

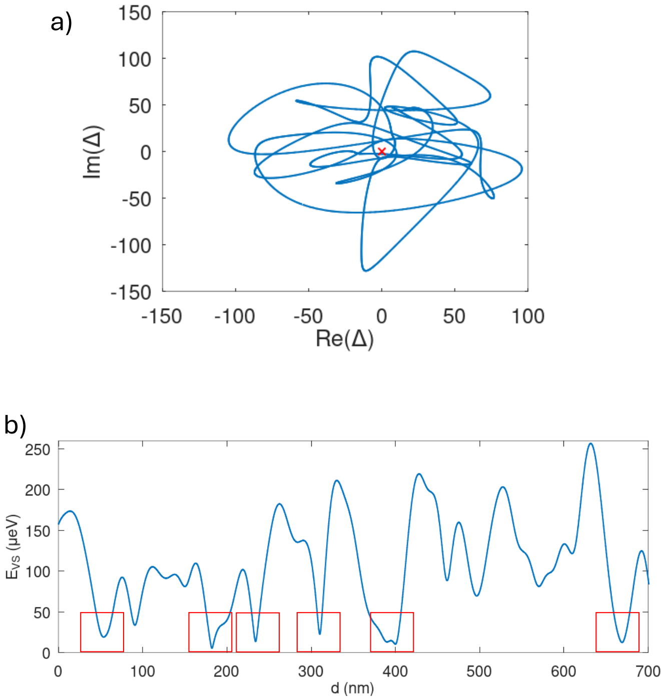

Supplementary Figure 11. a) Simulated trajectory of valley coupling parameter $\Delta(d)$ for $d \in [0, 700]$ nm. b) The corresponding d dependence of valley splitting $E_{\mathrm{VS}}(f) = 2|\Delta(d)|$. Six regions containing local minima with $E_{\mathrm{VS}} < 25$ μeV are marked with red squares. The values of E_{VS} at the consecutive minima are 19.1, 5.3, 13.6, 22.5, 10.4, and 12.5 μeV.

model. This model corresponds to $\Delta(x)$ trajectory being a straight line in the complex plane [4] that is traversed with constant velocity when $\mathrm{d}x/\mathrm{d}t \equiv v_S$ is constant. For any trajectory of $\Delta(x)$, the Landau-Zener approximation is obtained by finding the point on the trajectory of minimum value of $|\Delta|$, denoted as $|\Delta_0|$, and finding the tangent to the trajectory at this point and the value of $v_\Delta = \sqrt{(\mathrm{d}\Delta_R/\mathrm{d}x)^2 + (\mathrm{d}\Delta_I/\mathrm{d}x)^2}$, with derivatives evaluated at $x = x_0$ at which local minimum of valley splitting occurs, i.e. $E_{\mathrm{VS}}(x_0) = 2|\Delta_0|$. Valley dynamics due to shuttling along the tangent is described (after an appropriate choice of basis) by the Landau-Zener Hamiltonian

$$\hat{H}_{LZ} = \begin{pmatrix} v_\Delta v_S t & |\Delta_0| \\ |\Delta_0| & -v_\Delta v_S t \end{pmatrix} , \tag{S14}$$

which gives the valley excitation probability

$$Q_{LZ} = \exp\left(-\pi |\Delta_0|^2 / \hbar v_\Delta v_S\right) . \tag{S15}$$

Let us now see how well the dynamics near the E_{VS} minima can be approximated by the Landau-Zener model. In Fig. 12 we plot the $\Delta(x)$ trajectories near the 6 valley splitting minima marked in Fig. 11. For each of the trajectories, we calculate the probability of valley excitation assuming that the state at initial x is the lower-energy valley eigenstate, evolving it with time-dependent Hamiltonian $\hat{H}_v(t)$ (in which the time dependence comes from $\Delta(x)$ with $x = v_S t$), and calculating the modulus squared of the overlap of the final state with the higher-energy eigenstate of $\hat{H}_v$ in which $\Delta = \Delta_f$ is the valley coupling at the end of the given trajectory. The resulting final occupation of the excited valley state, Q_v, is plotted as a function of shuttling velocity v_S, and compared with the result of the Landau-Zener approximation calculated as discussed above.

The obtained results confirm the expectation that the Landau-Zener model does not necessarily accurately reproduce the exact Q_v, even if the knowledge of $\Delta(x)$ trajectory is used to choose the effective parameters for this model. This is most apparent for the 1st and 5th minimum. It should be noted that the discrepancy between the exact Q_v and Q_{LZ} in the latter case becomes more prominent at v_S larger than shown in the Figure. In both cases $\Delta(x)$ makes a partial or full loop around $\Delta = 0$. Let us note that for the 5th trajectory, this results in the presence of two closely spaced minima in $E_{\mathrm{VS}}(x)$.

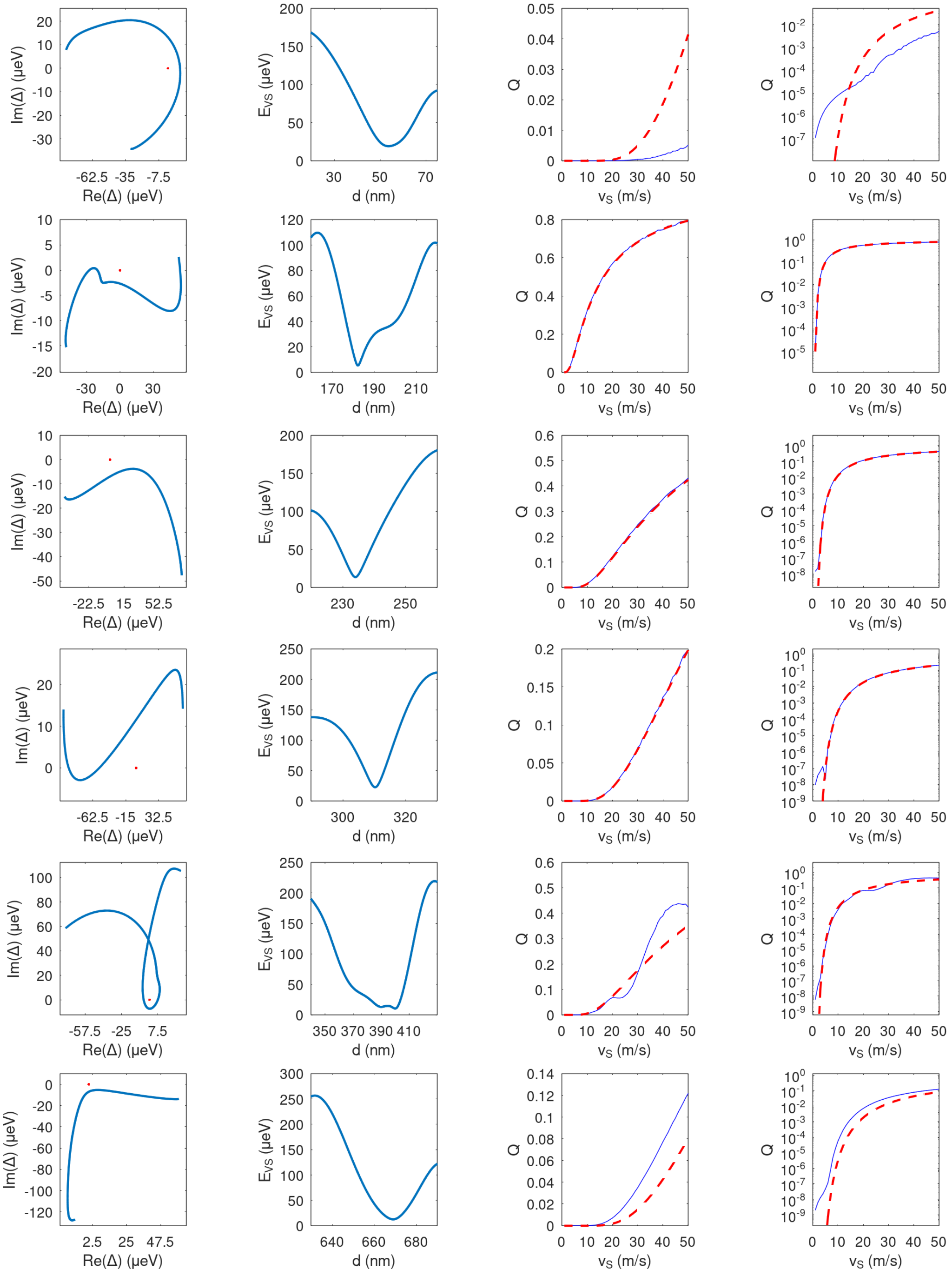

Supplementary Figure 12. Rows correspond to consecutive minima of E_{VS} marked in Fig. 11. 1st column: trajectory of $\Delta(x)$. 2nd column: the corresponding $E_{\mathrm{VS}}(x) = 2|\Delta(x)|$. 3rd column: Blue solid line is the probability of excitation out of initial lower energy valley state, Q_v, when shuttling over the distance corresponding to range of x for the plot from the 2nd column; red dashed line: Landau-Zener approximation Q_{LZ} from Eq. (S15); 4th column: the same in the logarithmic scale.

SUPPLEMENTARY NOTE VII: DYNAMICS DUE TO SHUTTLING THROUGH A SPIN-VALLEY RESONANCE: THE SPIN-VALLEY FLIP-FLOP

As we have by definition $E_{\mathrm{VS}} \geq 0$ and $E_Z^{\pm} > 0$, only $|-\uparrow\rangle$ and $|+\downarrow\rangle$ states can get close in energy, so only the Δ_{sv} spin-valley coupling can be relevant for dynamics, and it is in fact relevant only when $|\frac{1}{2}(E_Z^- + E_Z^+) - E_{\mathrm{VS}}| \lesssim 2|\Delta_{sv}|$. This is the region close to a spin-valley resonance defined by $E_z = E_{\mathrm{VS}}$ with $E_Z \approx E_Z^- \approx E_Z^+$. The measured values

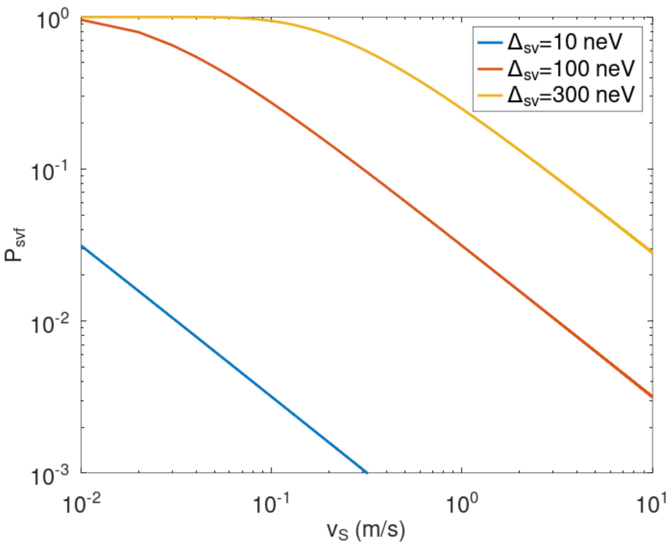

Supplementary Figure 13. Probability of adiabatic spin-valley flip-flop due to shuttling through the spin-valley resonance, Eq. (S17), plotted as a function of shuttling velocity v_S for three values of spin-valley coupling Δ_{sv}. We have used $|\mathrm{d}E_{\mathrm{VS}}/\mathrm{d}x| = 3$ μeV/nm, which is the average value from Fig. 3a in the main text.

of Δ_{sv} range from ≈ 20 neV [7], through ≈ 50 neV [2] to values in $100 - 150$ neV range measured here and in [8]. It is important to note that due to smallness of these couplings, they are relevant only in very narrow ranges of values of dot positions/shuttling times. Using the $E_{\mathrm{VS}}(d)$ map from Fig. 3a from the main text we obtain an average value of $|\mathrm{d}E_{\mathrm{VS}}/\mathrm{d}x| \approx 3$ μeV/nm, with maximal values measured ≈ 15 μeV/nm. The typical range of dot positions across which E_{VS} varies by ≈ 300 neV is thus $\Delta x \approx 0.1$ nm. We can safely neglect the spatial dependence of all the parameters in Hamiltonian from Eq. (S10) except for that of valley splitting, which can be very well approximated by $E_{\mathrm{VS}}(x) = \mathrm{const.} + v_\delta t$, where $v_\delta \equiv (\mathrm{d}E_{\mathrm{VS}}/\mathrm{d}x) \cdot v_S$. We arrive then at the spin-valley coupling Hamiltonian of the Landau-Zener form written in the $\{|-\uparrow\rangle, |+\downarrow\rangle\}$ basis:

$$\hat{H}_{sv} = \frac{1}{4}(E_Z^- - E_Z^+)\mathbb{1} + \begin{pmatrix} \frac{1}{2}v_\delta t & \Delta_{sv} \\ \Delta_{sv}^* & -\frac{1}{2}v_\delta t \end{pmatrix} . \tag{S16}$$

For evolution starting in $|-\uparrow(-t_i)\rangle$ eigenstate at time $-t_i$ fulfilling $|v_\delta t_i| \gg v$, and ending at t_f that also fulfills $|v_\delta t_f| \gg v$, the final occupation of the state with spin and valley indices changed, i.e. the state reached during an **adiabatic** evolution, follows from the Landau-Zener formula

$$P_{svf} = 1 - \exp\left(-2\pi\Delta_{sv}^2/\hbar v_\delta\right) , \tag{S17}$$

where the svf subscript stands for "spin-valley flipped". More precisely, the qubit traversing through a spin-valley resonance evolves as

$$\frac{1}{\sqrt{2}}(|-\uparrow\rangle + e^{i\alpha}|-\downarrow\rangle) \longrightarrow \frac{1}{\sqrt{2}}(a_{svf}|+\downarrow\rangle + b_{svf}|-\uparrow\rangle + e^{i\alpha}|-\downarrow\rangle) , \tag{S18}$$

where a_{svf} and b_{svf} follow from the solution of the Landau-Zener model, i.e $|a_{svf}|^2 = P_{svf}$ and $|b_{svf}|^2 = 1 - P_{svf}$. When $\hbar v_\delta \ll 2\pi\Delta_{sv}^2$ at a given resonance, the evolution is adiabatic, and the final state is given by

$$\frac{1}{\sqrt{2}}\left(|+\rangle + e^{i\alpha}|-\rangle\right) \otimes |\downarrow\rangle , \tag{S19}$$

showing that an adiabatic passage through a spin-valley anticrossing converts the spin superposition into the valley superposition, as noted in [12]. An analogous effect occurs if the incoming electron is in $|+\rangle$ valley state: then the $|+\uparrow\rangle + e^{i\alpha}|+\downarrow\rangle$ state is converted by adiabatic passage through the resonance to $(|+\rangle + e^{i\alpha}|-\rangle) \otimes |\uparrow\rangle$.

In Figure 13, we show how P_{svf} given by Eq. (S17) varies as a function of shuttling velocity for values of spin-valley coupling Δ_{sv} spanning the range of these couplings seen in experiments. The key notion is that for $\Delta_{sv} \gtrsim 100$ neV the transition between adiabatic and diabatic dynamics upon shuttling across a spin-valley resonance happens for $v_S \sim 1$ m/s. For significantly lower $|\Delta_{sv}|$ the dynamics is expected to be diabatic (i.e. with small probability of spin-valley flip-flop) for $v_S > 0.01$ m/s.

SUPPLEMENTARY NOTE VIII: MECHANISMS OF DECOHERENCE DURING THE SHUTTLING

A: Dephasing in absence of spin-valley resonances and valley excitations

Let us draw attention to an important feature of the dynamics of spin and valley degrees of freedom of the shuttled electron that follow from Hamiltonian (S10). As the shuttling velocity v_S is lowered, off-diagonal terms driving the spin-conserving inter-valley transitions, $\hbar\dot{\phi}$, tend to zero. On the other hand, the spin-valley coupling terms, Δ_{sv}, are independent of shuttling velocity. Consequently, when the shuttled electron encounters the spin-valley resonance, then at the lowest velocities at which the evolution is fully adiabatic, the electron undergoes a spin-valley flip at each resonance. Later we will discuss how such a spin-valley flip changes the nature of dephasing of the qubit, but first let us discuss the dephasing of the qubit that undergoes the evolution that is adiabatic with respect to the valley-changing terms, and no spin-valley resonances are present. Alternatively, if the resonances are present, we assume now that dynamics during passage through them is as close to diabatic as possible, so that the state of the electron is completely unaffected by passage through them.

The dynamics following from Hamiltonian (S10) is then rather trivial: the qubit remains in the instantaneous valley eigenstate, while the spin splitting $E^{-}_{Z,R}(t)$ varies due to electron motion and spatial dependence of g-factor of the qubit (given by $g_{-}(x(t)$ when the electron is in the lower-energy valley state). In this case of purely adiabatic valley dynamics and no influence of spin-valley resonances on the shuttling path, the spin qubit shuttled for time $\tau_S/2$ acquires the relative phase between its two states given by

$$\phi(\tau_S/2) = \int_0^{\tau_S/2} E^{-}_{Z,R}(x(t))\mathrm{d}t \tag{S20}$$

where $E^{-}_{Z,R}(x)$ is the Zeeman splitting for qubit is a dot localized at x. In the experiments in this paper a spin singlet state of the shuttled qubit and an electron left behind in half-filled valley in the stationary L dot is created, and the coherence measurement is done after shuttling the qubit back and performing the Pauli spin blockade readout. Consequently, the phase acquired by the singlet-triplet superposition during back-and forth shuttling that takes the total time τ_S is given by an analogous expression in which $E^{-}_{Z,R}$ is replaced by $\Delta E_Z \equiv E_{Z,L} - E^{-}_{Z,R}$, and shuttling in both directions contributes to the phase. Let us now assume that the electron motion can be well approximated as having constant velocity v_S, with the sign of v_S simply changed on the way back. This leads to an expression equivalent to Eq. (3) in the main text:

$$\phi(\tau_S) = 2\frac{1}{\hbar}\int_0^{\tau_S/2} \Delta E_Z(vt)\mathrm{d}t = \left[\frac{1}{d}\int_0^{d}\frac{1}{\hbar}\Delta E_Z(d)\mathrm{d}x\right]\tau_S \equiv \bar{\omega}(d)\tau_S \equiv 2\pi\bar{f}(d)\tau_S \tag{S21}$$

in which $d = v_S\tau_S/2$, and $\bar{f}(d)$ is the precession frequency averaged over the shuttling distance d.

Hyperfine coupling to very slowly fluctuating nuclei , and low-frequency charge noise leading to slow fluctuations of electron g-factors cause quasi-static (i.e. static at the few microsecond timescale of single shuttling, but changing over the whole time of data acquisition) shifts of Zeeman splittings in the two dots. For the shuttled R dot these shifts are of course dependent on the dot position. Averaging over a Gaussian distribution of these shifts for the static dot contributes a constant $\sigma^2_L/\hbar^2$ to variance of $\tilde{\omega}(d)$. On the other hand, the contribution from the moving dot depends on the shuttling distance d (for derivation see e.g. the supplementary online information of [13]). Assuming that the shift for the shuttled dot is a Gaussian random field with autocorrelation length l_c and Gaussian-shaped autocorrelation function (which applies to the case of $E_{Z,R}$ fluctuations due to nuclear Overhauser fields, see [13]), the resulting variance of the distance-averaged frequency is given by

$$\frac{1}{\hbar^2}\sigma^2(d) = \frac{1}{\hbar^2}\sigma^2_L + \frac{1}{\hbar^2}\sigma^2_S(d;\sigma_R, l_c)\ , \tag{S22}$$

with the variance contributed by the shuttled spin, $\sigma^2_S(d)$, given by

$$\sigma^2_S(d;\sigma_R,l_c) = \sigma^2_R\left[\frac{2l_c^2}{d^2}\left(e^{-d^2/2l_c^2}-1\right) + \frac{\sqrt{2\pi}l_c}{d}\mathrm{Erf}(d/\sqrt{2}l_c)\right]\ , \tag{S23}$$

where σ^2_R is the typical variance of the spin splitting for the dot positioned within the shuttling channel. Note that for $d \gg l_c$ we have $\sigma^2_S(d) \approx \sqrt{2\pi}\sigma^2_R l_c/d$. For nuclear Overhauser field noise and Gaussian approximation for the shape of electron's envelope wavefunction l_c is the confinement length of electrons' wavefunction along the shuttling direction, and $l_c \approx 20$ nm. For contribution due to g-factor fluctuations caused by low frequency charge noise we can suspect

that the relevant autocorrelation length will be the electric field noise autocorrelation length (estimated to be ~ 100 nm in [14]). The key feature of this result (which requires only that the noise is quasi-static, and is independent of assumptions on the shape of the spatial autocorrelation function of the noise) is however that the decay of the singlet return probability signal oscillation is in this case Gaussian:

$$P_S(\tau_S) = \frac{1}{2} + \frac{1}{2}\cos\left[(\bar{\omega}(d)\tau_S\right]\exp\left[-\left(\frac{\tau_S}{T^*_{2,L}}\right)^2\right]\exp\left[-\left(\frac{\tau_S}{T^*_2(d)}\right)^2\right] , \tag{S24}$$

where $T^*_2(d) \equiv \hbar\sqrt{2}/\sigma_S(d)$, and the static dot dephasing time is $T^*_{2,L} \equiv \hbar\sqrt{2}/\sigma_L$. Note that $T^*_2(d)$ depends **only** on the shuttling distance d - it has no dependence on the shuttling velocity v_S with which the path to d and back is traversed. It also does not depend on the number of repetitions of the shuttling, if the cycle of going to d and back is repeated n_{rep} times: in such a case we obtain the above formula with τ_S simply replaced by the total shuttling time given by $\tau \equiv n_{\text{rep}}\tau_S$. Results from shuttling experiments in [13] were well fit with the above formulas, and characteristic dependence $T^*_2(d) \propto \sqrt{d/l_c}$ due to motional narrowing of influence of quasi-static random fields was observed there.

When other processes (which we discuss below) additionally contribute to the decoherence of the shuttled spin, the decay term in Eq. (S24) describing the dephasing of the spin in the static L dot stays the same, while the second Gaussian decay term becomes modified (with changed $T^*_2(d)$) or replaced by a qualitatively distinct term, e.g. exponential decay $\propto \exp(-\tau/T)$.

B: Dephasing due to valley splitting fluctuations activated by the spin-valley flip-flop

Let us focus now on dephasing of the valley superposition state from Eq. (S19). For simplicity we assume that a fully adiabatic dynamics leads to a spin-valley flip-flop that occurs at a single resonance at $x_{sv} < d$, where d is the distance to which the qubit is shuttled, and then the qubit is shuttled back to its starting position while passing through the resonance again. Of course, during the backward shuttling the qubit's $|+\downarrow\rangle$ state get adiabatically mapped back onto $|-\uparrow\rangle$ state.

The relative phase of two states of the valley qubit that exists during shuttling from x_{sv} to d and then back to x_{sv} is sensitive to fluctuations of valley splitting. Furthermore, in the relevant here case of a system initialized in an entangled state involving an electron in valley ν on the L dot and the shuttled electron in the $-$ valley state in the moving R dot, the state existing at $x < x_{sv}$ is given by

$$|\Phi_{in}\rangle = \frac{1}{\sqrt{2}}(|\nu\uparrow,-\downarrow\rangle + e^{i\alpha}|\nu\downarrow,-\uparrow\rangle) \tag{S25}$$

and upon crossing through the spin-valley resonance it is converted to

$$|\Phi_{out}\rangle = \frac{1}{\sqrt{2}}(|\nu\uparrow,-\downarrow\rangle + e^{i\alpha'}|\nu\downarrow,+\downarrow\rangle) \tag{S26}$$

and the energy splitting between the two states is then given by

$$\Delta E = \frac{1}{2}(\nu E_{VL} + E_{Z,L} - E_{VR} - E_{ZR} - \nu E_{VL} + E_{ZL} - E_{VR} + E_{ZR}) = E_{ZL} - E_{VR} , \tag{S27}$$

so that the phase superposition state after spin-valley flip-flop of the moving dot is sensitive to fluctuations of the spin splitting in the L dot and of the valley splitting of the R dot.

The phase picked up by the superposition during the shuttling to d and back is then given by

$$\phi(\tau_S) = \frac{1}{\hbar}\left[E_{ZL} - \left(\frac{1}{x_{sv}}\int_0^{x_{sv}} E_{ZR}(x)\mathrm{d}x\right)\cdot\frac{x_{sv}}{d} + \left(\frac{1}{d-x_{sv}}\int_{x_{sv}}^{d} E_{VR}(x)\mathrm{d}x\right)\cdot\frac{d-x_{sv}}{d}\right]\tau_S \equiv \bar{\omega}(x_{sv},d)\tau_S \tag{S28}$$

The crucial thing to note here is that since $E_{VR} \in [1, 100]$ μeV, while $|E_{ZL} - E_{ZR}|$ is at most ~ 0.1 μeV at highest considered B fields, the contribution of the integral over E_{VR} to the frequency of singlet-triplet oscillation $\bar{\omega}(x_{sv}, d)$ strongly dominates over the contribution from integrals over Zeeman splitting as $d - x_{sv}$ distance is increased. When $(d - x_{sv})/d$ becomes larger than the ratio of $|E_{ZL} - E_{ZR}|/E_{VR}$ that is typically $\ll 0.1$, the frequency $\bar{\omega}(x_{sv}, d)$ increases by at at least an order of magnitude. In order to see such a large increase of oscillation frequency when d becomes larger than x_{sv} the $P_S(\tau_S)$ signal should be sampled with frequency orders of magnitude higher than the one used to

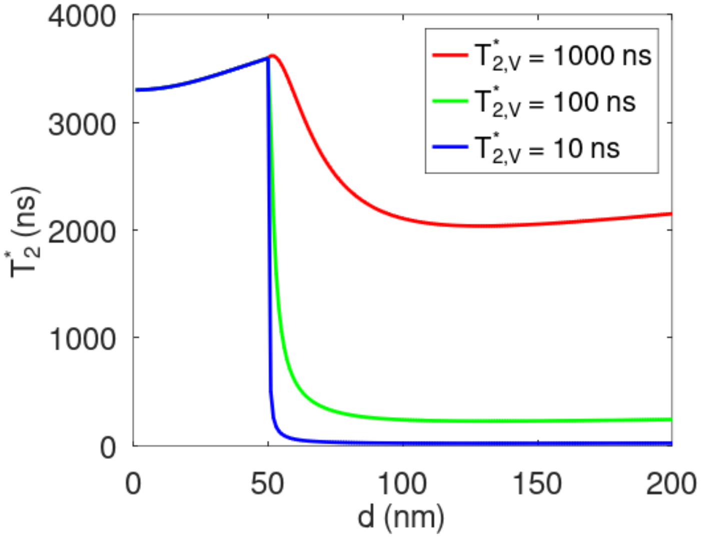

Supplementary Figure 14. Dephasing time T_2^* following from Eqs. (S30) and (S29) characterizing the decay of singlet return probability when the shuttled qubit passes *adiabatically* through a spin-valley resonance at $x_{sv} = 50$ nm when shuttled across distance d and back. The values of rms of spin splitting in both the stationary and the shuttled dot are $\sigma_L = \sigma_R = 0.2$ neV, resulting in $T_2^* \approx 3.3$ μs for a singlet initialized in a static double quantum dot. The superposition of spin states of the shuttled qubit, Eq. (S25), is adiabatically converted into the superposition of valley states, Eq. (S26), upon crossing the spin-valley resonance at x_{sv}, and it then undergoes dephasing due to valley splitting fluctuations until the qubit is shuttled again through the spin-valley resonance on its way back. Three values of rms of valley splitting, σ_V, are considered, corresponding to dephasing times of valley superposition in a static dot given by $T^*_{2,V} = \hbar\sqrt{2}/\sigma_V = 10$, 100, 1000 ns.

reconstruct much slower oscillations in the $10 - 100$ MHz range due to Zeeman splitting differences between L and R dots. A signal sampled with too large time delay between mesurement times will look as white noise.

Let us consider now the dephasing of the oscillations. In presence of noise in spin and valley splittings, $\delta E_{Z,L(R)}$ and δE_{VR}, respectively, the calculation of variance of this phase follows the derivation from [13]. We make a natural assumption that $\delta E_{VR}(x)$ is uncorrelated with $\delta E_{ZR}(x)$. Fluctuations of valley splitting in a stationary dot are expected to be caused by low-frequency electric field noise. Noise in out-of-plane electric field affects the overlap between the electron's wavefunction and the Si/SiGe interface (which obviously leads to fluctuations of $E_{\rm VS}$), while noisy in-plane electric field shifts the dot, leading to random change in valley splitting due to dot-position dependence of $E_{\rm VS}$. For the dot moving along the x axis the effect of electric fields in z and y direction on valley splitting will be the same, while fluctuating electric fields in x direction will lead to randomness in velocity fluctuations. It is thus reasonable to assume that the valley splitting fluctuations experienced by the moving dot are quite similar to fluctuations for stationary dots. Largest reported dephasing times of valley qubits in such stationary dots are of the order of at most a microsecond [15, 16], and much shorter valley dephasing times were extracted from measurements in Si/SiGe structures from [13] and those used here [1].

While the charge-noise induced rms of valey splitting for a dot at position x, $\sigma_V(x)$, is expected to depend on x, for simplicity we will calculate the valley-splitting-fluctuation induced dephasing by using a single number, σ_V, as a measure of typical magnitude of $\delta E_{\rm VS}$ fluctuations along the shuttling path. We will also assume that the spatial autocorrelation function of valley splitting fluctuations has a Gaussian form, $\langle \delta E_{\rm VS}(x)\delta E_{\rm VS}(x')\rangle \propto \exp\bigl(-(x-x')^2/2l_{c,v}^2\bigr)$, where $l_{c,v}$ is the autocorrelation length of valley splitting fluctuations. With these assumptions we can reuse the results of calculation leading to Eq. (S23) for the case of dephasing of valley superposition. We are now dealing with a qubit the phase of which is affected by Overhauser field fluctuations during shuttling from $x = 0$ to $x = x_{sv}$, then by E_V fluctuations during shuttling from $x = x_{sv}$ to $x = d$ and back to $x = x_{sv}$, and finally again by Overhauser field fluctuations during shuttling from $x = x_{sv}$ to $x = 0$. Calcualtion analogous to one leading to Eq. (S23) leads to the following expression for variance of $\hbar\bar{\omega}(x_{sv}, d)$:

$$\sigma^2(x_{sv}, d) = \sigma_L^2 + \left(\frac{x_{sv}}{d}\right)^2 \sigma_S^2(x_{sv}; \sigma_R, l_c) + \left(\frac{d - x_{sv}}{d}\right)^2 \sigma_S^2(d - x_{sv}; \sigma_V, l_{c,v}) \ , \tag{S29}$$

where $\sigma_S^2(x;\sigma_{R(V)}, l_c(l_{c,v}))$ is given by Eq. (S23). In Figure 14 we plot the resulting dephasing time

$$T_2^*(x_{sv}, d) = \frac{\hbar\sqrt{2}}{\sigma(x_{sv}, d)} \ , \tag{S30}$$

for $\sigma_L = \sigma_R = 0.2$ neV, which corresponds to $T_2^* \approx 3$ μs for a spin singlet in absence of shuttling, and for values of σ_V corresponding to valley superposition dephasing times $T_{2,V}^* \approx 10$ ns, 100 ns, and 1 μs. The value of $x_{cv} = 50$ nm and range of d are chosen to match the experiment at $B = 0.3$ T. For correlation lengths we use $l_c = 20$ nm (as expected based on simulated size of the shuttled dot [13]) and choose $l_{c,v} = l_c$ for simplicity (note that we do not expect these two correlation lengths to have distinct orders of magnitude). The key thing to note is that when $\sigma_V \gg \sigma_{L(R)}$, an adiabatic spin-valley flip-flop leads to very strong dephasing of the singlet return probability.

C: Spin relaxation near the spin-valley relaxation resonance

Spin-valley coupling Δ_{sv} leads to strong mixing of $|-\uparrow\rangle$ and $|+\downarrow\rangle$ states when $|E_{\mathrm{VS}} - E_Z| \lesssim 2|\Delta_{sv}|$. This mixing results in enhanced probability of spin relaxation from $\widetilde{|-\uparrow\rangle} = a|-\uparrow\rangle + b|+\downarrow\rangle$ (with $|a|^2 \approx |b|^2 \approx 1/2$ close to the resonance) eigenstate that is adiabatically connected to $|-\uparrow\rangle$ away from the spin-valley resonance [7, 17]. The spin relaxation rate at such a relaxation hotspot, Γ_s, becomes equal to half of valley relaxation rate, Γ_v, when $|E_{\mathrm{VS}} - E_Z| \ll |\Delta_{sv}$, while for $|E_{\mathrm{VS}} - E_Z| \gg |\Delta_{sv}|$ we have $\Gamma_s \propto \Gamma_v |\Delta_{sv}|^2/(E_{\mathrm{VS}} - E_Z)^2 \ll \Gamma_v$. Consequently, as the value of E_{VS} of the shuttled dot is swept through the spin-valley resonance with rate v_δ, see Eq. (S16), the total probability of spin relaxation at the resonance, p_{sr}, can be upper bounded by $\Gamma_v \Delta t$, where $\Delta t = 4|\Delta_{sv}|/v_\delta$ is the time spent in the vicinity of the relaxation hotspot. As $v_\delta \propto v_S$, the probability of spin relaxation increases with decreasing shuttling velocity v_S.

D: Valley excitation after shuttling through the valley splitting minimum

As discussed in Sec. SUPPLEMENTARY NOTE VI, shuttling through the E_{VS} minimum puts the shuttled electron in a superposition of valley eigenstates (see Eq. S13), while maintaining the superposition state of the spin degree of freedom. Let us note that rather fast (compared to timescales of shuttling) dephasing of valley superpositions, discussed in Sec. SUPPLEMENTARY NOTE VIII B, quickly turns the valley superposition into incoherent statistical mixture of qubit being in the initial valley state (before passage through the E_{VS} minimum) with probability of $1 - Q_v$, and in the other valley eigenstate with probability Q_v, see Sec. SUPPLEMENTARY NOTE VI for discussion of expected magnitude and velocity-dependence of Q_v. During subsequent evolution the spin states occupying the two valleys will undergo precession with distinct frequencies due to finite difference of g-factors for the shuttled electron in two valley eigenstantes resulting in finite difference of Zeeman splittings, $\delta E_{Z,R}(x)$. Shuttling across the valley-splitting minimum acts then as an incoherent beam splitter for the qubit, and the spin phases acquired during subsequent evolution through the two "paths" are distinct. As discussed in [12], if valley relaxation occurs with non-negligible probability during shuttling, it leads to randomization of qubit phase and results in the spin qubit arriving in a partially dephased state to its destination. The presence in the measured signals of multiple frequencies, associated with occupation of both valley states in the stationary and the shuttled dot, means that the valley relaxation is not very efficient on timescales of up to ~ 10 μs that are of relevance for the discussed experiments. This means that for the qubit shuttled across distance d and then subjected to measurement that is not valley selective (as the Pauli Spin Blockade measurement used in the experiment here clearly is), spin precession consisting of contributions with two frequencies would be obtained. While arrival of a qubit in each of two valley states with nonzero probability poses challenges for subsequent processing of quantum information encoded in the spin degree of freedom, this process does not really qualify as spin dephasing. However, when describing the experiments involving multiple there-and-back shuttle cycles before the measurement is done, we need to average over many paths in valley space (due to processes of valley state change occurring with finite probability at multiple times of passages through E_{VS} minima), and leads to averaging over many frequencies, resulting in true dephasing of the signal. This will be discussed in detail in Sec. SUPPLEMENTARY NOTE X B.

SUPPLEMENTARY NOTE IX: DISCUSSION OF EXPERIMENTAL RESULTS: EFFECT OF SINGLE THERE-AND-BACK SHUTTLING THROUGH THE SPIN-VALLEY RESONANCE

In Fig. 3c,f of the main text we see how passage through the spin-valley resonance present at $x_{sv} \approx 50$ nm when $B = 0.3$ T suppresses the amplitude of the singlet-triplet oscillations. Also, as one can most clearly see by focusing on the lower-left corner of Fig. 3c, this suppression of oscillations becomes stronger as the velocity with which the resonance is traversed decreases, with $v_S \sim 0.1$ m/s roughly delineating the boundary between retaining the same order of magnitude of visibility of oscillations and strong suppression of this visibility. Above we have discussed two mechanism that could explain this effect: (1) transition between diabatic and adiabatic passage through the spin-valley resonance as v_S is lowered, with adiabatic passage turning the spin qubit into a valley qubit and making it sensitive to valley splitting fluctuations, and (2) spin relaxation rate enhancement in the vicinity of the spin-valley resonance (the relaxation hotspot effect), leading to spin relaxation probability which scales with time spent in this vicinity, and thus with inverse of shuttling velocity v_S. Let us now discuss the expected significance of these two mechanisms while taking into account the parameters of the system inferred from experiments.

First we consider spin relaxation at the spin-valley resonance. As discussed above, the probability of relaxation is $p_{sr} < 4\Gamma_v|\Delta_{sv}|/v_\delta$, where $v_\delta = |\mathrm{d}E_V(x)/\mathrm{d}x| \cdot v_S$. Using the data on $E_V(x)$ from Fig. 3a of the main text we estimate $|\mathrm{d}E_V(c)/\mathrm{d}x| \approx 10$ μeV/nm at $x_{sv} = 50$ nm. Taking $\Delta_{sv} = 300$ neV as the estimate of maximal spin-valley coupling expected in Si/SiGe structures, we obtain that p_{sr} can become ~ 0.1 at $v_S \approx 0.1$ m/s if the valley relaxation rate is $\Gamma_v \geq \mathrm{ns}^{-1}$, i.e. the valley relaxation time is < 1 ns. This value is orders of magnitude shorter than the valley relaxation times (or spin-valley relaxation times near the hotspots) reported in literature [7, 17, 18]

Let us probe the issue of minimal expected valley relaxation time a bit deeper. The valley relaxation rate, Γ_v, strongly depends on E_V, as $\Gamma_v \propto E_V^7$ [12], and also on the interface roughness at a given dot position, as this roughness activates the valley-orbit coupling [19] that is necessary for valley relaxation. It is thus impossible to give even an order-of-magnitude estimate of Γ_v in a sample in which E_V spans almost two orders of magnitude. Let us however note that calculations done for a particular model of interface roughness (a single monoatomic step) gave valley relaxation times $\tau_v = 1/\Gamma_v > 1$ ms for $E_V < 100$ μeV [12]. Since at $x_{sv} = 50$ nm we have $E_V(x_{sv}) \approx 35$ μeV, it seems that it is rather improbable that valley relaxation time at this x_{sv} could be 9 orders of magnitude larger than the one calculated in [12] for any possible realization of atomic disorder at the interface.

Finally, let us note that the presence of multiple frequencies of singlet return probability oscillations in Figs. 3e-g strongly suggest that excited valley states in both the stationary and the shuttled dot are appreciably occupied during the whole shuttling time that reaches ~ 1 μs. This clearly shows that sub-microsecond valley relaxation times are not expected anywhere along the shuttling channel. Having $\tau_v \sim 1$ ns at $x = x_{sv}$, so that the spin relaxation at this point becomes fast enough to bring a large change in singlet return probability signal when the neighbourhood of x_{sv} is visited for $\Delta t \sim 1$ ns, is thus highly improbable.

This leaves us with the mechanism of diabatic-to-adiabatic change of character of electron spin and valley dynamics during shuttling though the spin-valley resonance, as an explanation of suppression of singlet-triplet coherence when v_S drops below ~ 0.1 m/s. As discussed in Sec. SUPPLEMENTARY NOTE VIII B, adiabatic passage through the resonance converts a spin superposition into a valley superposition, and exposes the coherence of the superposition to valley splitting fluctuations.

In Sec. SUPPLEMENTARY NOTE VIII B we have discussed the dephasing for shuttling to $d > x_{sv}$ and back for adiabatic dynamics thorugh the resonance: the spin qubit was converted into a valley qubit at x_{sv}, propagated as such to d and back to x_{sv}, and then was adiabatically converted back to a spin qubit. In order to capture the behavior shown in Fig. 3c,f of the main text we have to account for the degree to which the spin-valley dynamics is adiabatic at given v_S. This means that the state of the spin qubit approaching the spin-valley resonance for the first time transforms upon the passage as in Eq. (S19) where the amplitudes a_{svf} and b_{svf} are obtained by solving the Landau-Zener model from Eq. (S16) for given value of Δ_{sv} and valley splitting sweep rate v_δ that is related to the real space shuttling velocity by $v_\delta \equiv (\mathrm{d}E_{\mathrm{VS}}/\mathrm{d}x) \cdot v_S$. The state after the passage experiences dephasing due to spin splitting and valley splitting fluctuations, and it undergoes the second Landau-Zener evolution during the backwards shuttling through the spin-valley resonance. The full theory of spin-valley dynamics and dephasing should thus account for possibility of Landau-Zener-Stueckelberg-Majorana (LZSM) interference due to two coherent "beam splitting" events, with partial dephasing of superposition of three relevant states happening between them. In order to simplify the model to capture the key effect expected when dephasing of valley superposition is much stronger than that of spin superpositions, we assume that any intervalley coherence is simply lost during shuttling from x_{sv} to d and back to x_{sv}. This means that after the full shuttling cycle, the oscillations of singlet return probability are due to events in which a diabatic path was taken through the anticrossing on way both "there" and "back" (which happen with probability $|b_{svf}|^4$), with the dephasing of these oscillations caused by Zeeman splitting fluctuations only. All the other paths (adiabatic on way "there", diabatic on way "back", etc) result in either an arrival of a polarized tripled, or an arrival of completely dephased superposition of a singlet an unpolarized triplet. Consequently, the oscillating part of the

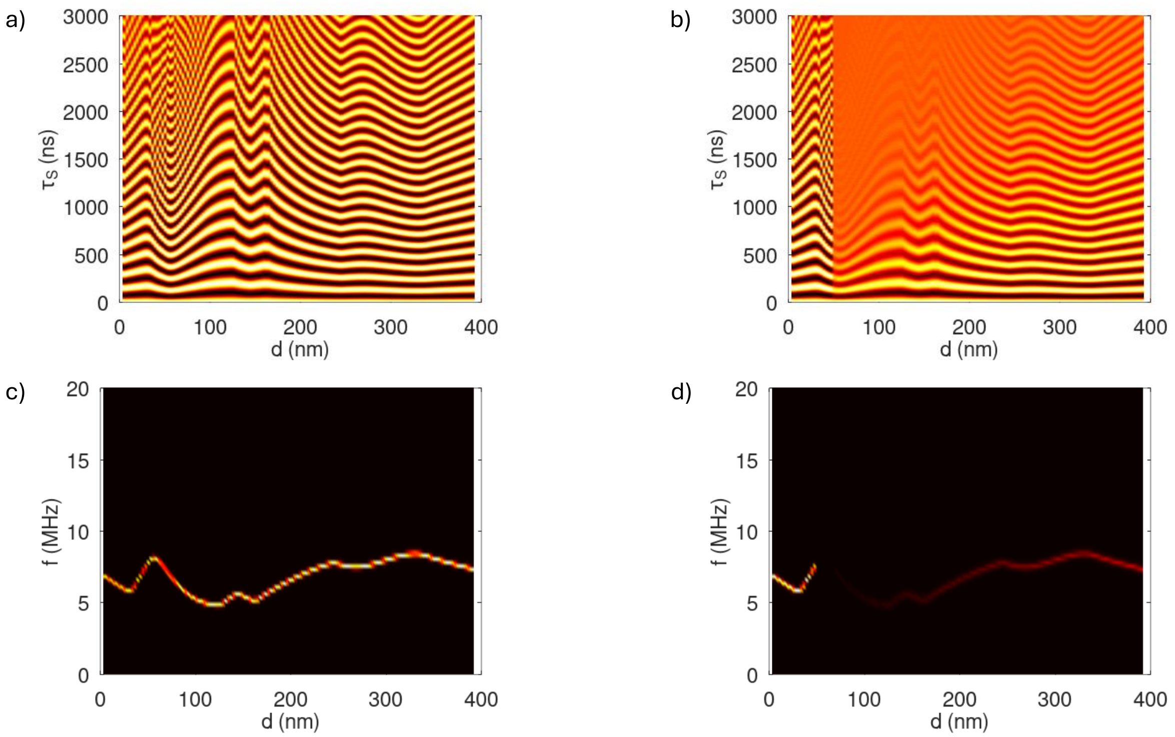

Supplementary Figure 15. (a) Simulation of $P_S(d, \tau_S)$ signal at $B = 0.3$ T using Eqs. (S24) and (S23), with $\delta g(x)$ data extracted from experiments, and $T_2^*(d)$ time following from assuming static single spin qubit $T_2^* = 4.66$ μs in each of the dots (due to hyperfine coupling to nuclei and g-factor fluctuations due to low-frequency electric field noise). (b) An analogous simulation, but taking into account the effect of passage through spin-valley resonance at $x_{sv} = 50$ nm, assuming $\Delta_{sv} = 300$ neV and very fast dephasing of valley superpositions. (c,d) Corresponding Fourier transforms with respect to τ_S. Note the similarity between panels (b) and d here and corresponding panels in Fig. 3 in the main text. Note that the second frequency visible in the signal in Fig. 3f is not included in the model used here, as we focus on effects of passage through a spin-valley resonance for any of the singlets that are occupied in the experiment.

singlet return probability is given by

$$P_S(\tau_S) \propto |b_{svf}|^4 \cos\left[(\bar{\omega}(d)\tau_S\right] \ \exp\left[-\left(\frac{\tau_S}{T_2^*(d)}\right)^2\right] \tag{S31}$$

with $\bar{\omega}(d)$ and $T_2^*(d)$ given in Sec. SUPPLEMENTARY NOTE VIII A, and

$$|b_{svf}|^2 = \exp\left(-2\pi\Delta_{sv}^2/\hbar v_\delta\right) \ . \tag{S32}$$

In Figure 15b we plot the signal given by the above formula assuming $\Delta_{sv} = 300$ neV, and $|\mathrm{d}E_{\mathrm{VS}}/\mathrm{d}x| = 10$ μeV/nm. In Fig. 15d we show the Fourier transform of this result with respect to τ_S, and we see a clear qualitative similarity to results shown in Fig. 3f in the main text.

SUPPLEMENTARY NOTE X: DISCUSSION OF EXPERIMENTAL RESULTS: MULTIPLE PASSAGES THROUGH VALLEY SPLITTING MINIMUM AND SPIN-VALLEY RESONANCES

Let us discuss now the decoherence caused by shuttling the qubit n times over distance λ and back, while passing through a valley splitting minimum and/or a spin-valley resonance. The data from Fig. 4a and Table I of the main paper show that when no valley splitting minima with $E_{\mathrm{VS}}^{\mathrm{min}} \lesssim 10$ μeV, and no spin-valley resonances are encountered on the shuttling path, the spin dephasing follows Gaussian decay with no significant v_S dependence of $T_2^*(\lambda)$ time, as previously reported [13]. This is the expected result when dephasing is due to quasi-static fluctuations of spin splitting, see [13] and Sec. SUPPLEMENTARY NOTE VIII A above. On the other hand, as the other panels of Fig. 4

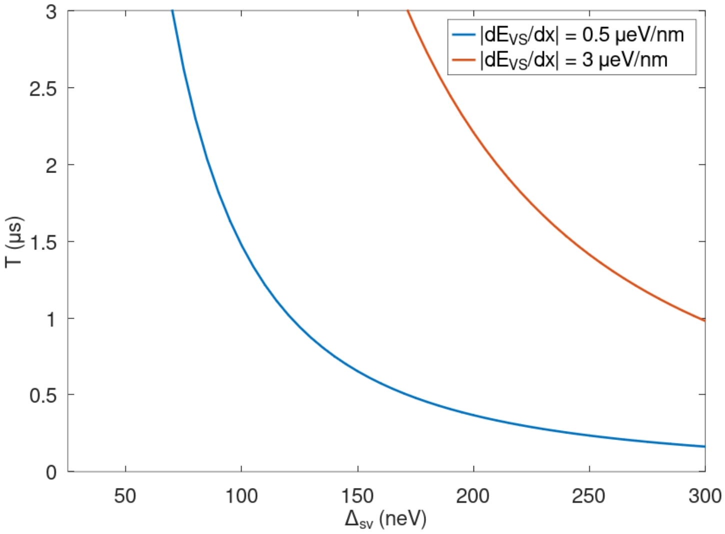

Supplementary Figure 16. Decoherence time T from Eq. (S37) due to multiple passages through a spin-valley resonance, plotted as a function of the spin-valley coupling Δ_{sv} for two values of valley splitting gradient at the resonance.

and Table I from the main paper show, shuttling through two valley splitting minima with $E_{\mathrm{VS}}^{\mathrm{min}} \lesssim 10$ μeV at $d \approx 232$ and 245 nm (and through two spin-valley resonances at $B = 40$ mT) leads to coherence decay that is better fit by an exponential, i.e. having the envelope of the oscillations damped as $e^{-\tau/T}$, with the decoherence time T decreasing with shuttling velocity v_S and magnetic field B increasing. Let us analyze now how multiple passages through the valley splitting mimum and/or the spin-valley resonance can lead to exponential decay of coherence, and what coherence times can be expected taking into account the typical parameters in the measured samples.

A: Passages through a spin-valley resonance

First, we generalize the model from Sec. SUPPLEMENTARY NOTE VIII B describing qubit dephasing due to shuttling across the spin-valley resonance, to the case of multiple there-and-back passages. We begin by noting that for $v_S \geq 5.6$ m/s used in there-and-back shuttling experiments, the probability of spin-valley flip-flop, P_{svf} given by Eq. (S17), is $\ll 1$ for $\Delta_{sv} \leq 300$ neV, and for $|\mathrm{d}E_{\mathrm{VS}}/\mathrm{d}x| > 0.5$ μeV/nm. As the average of $|\mathrm{d}E_{\mathrm{VS}}/\mathrm{d}x|$ shown in Fig. 3a of the main text is ≈ 3 μeV/nm, we can expect that shuttling across the spin-valley resonance with v_S above a few m/s results in mostly diabatic spin-valley dynamics, with events of spin-valley flip-flop occuring with $P_{svf} \propto 1/v_S \ll$. More specifically, for the two spin-valley resonances encountered at $B = 40$ mT and $d \in [230, 240]$ (see Fig. 4e of the main text), we have $|\mathrm{d}E_{\mathrm{VS}}/\mathrm{d}x| \approx 3$ and 0.5 μeV/nm, so the assumption of $P_{svf} \ll 1$ almost certainly holds for these resonances.

As above we assume that each event of spin-valley flip-flop leads to complete loss of coherence of the qubit. The decay of the envelope of the oscillating part of the singlet return probability signal, for n there-and-back passages is then given by

$$W_n = (1 - P_{svf})^{2n} = \exp\left(2n \ln[1 - P_{svf}]\right) \approx e^{-2nP_{svf}} \, , \tag{S33}$$

where we have used $\ln(1-x) \approx -x + O(x^2)$ for $x \ll 1$. Rewriting W_n as a function of total shuttling time $\tau = n\tau_S$, where $\tau_S = 2\lambda/v_S$ is the time of shuttling to λ and back, we obtain

$$W(\tau) = e^{-\tau/T} \, , \tag{S34}$$

where the decoherence time is

$$T = \frac{\tau_S}{2P_{svf}} = \frac{\lambda}{v_S P_{svf}} \, , \tag{S35}$$

and after using

$$P_{svf} \approx \frac{2\pi\Delta_{sv}^2}{\hbar v_S |\mathrm{d}E_{\mathrm{VS}}/\mathrm{d}x|} \, , \tag{S36}$$

we finally arrive as

$$T \approx \frac{\hbar\lambda|\mathrm{d}E_{\mathrm{VS}}/\mathrm{d}x|}{2\pi\Delta_{sv}^2} \ . \tag{S37}$$

The key things to note is the lack of dependence of this T on v_S, and a weak dependence of B field, as long as the resonance is actually present (i.e. $2\mu_B B$ is larger than E_{VS}^{min}). The first result follows from applicability of Landau-Zener model to the description of the spin-valley flip-flop, resulting in $P_{svf} \sim 1/v_S$, and consequently an approximate cancellation of v_S dependence from the denominator of Eq. (S35). As for the second, the implicit dependence of T on B comes from dependence of $|\mathrm{d}E_{\mathrm{VS}}/\mathrm{d}x|$ and Δ_{sv} on the value of valley splitting at which the spin-valley resonance, occurs, $E_{\mathrm{VS}} = 2\mu_B B$.

In Fig. 16, we show the coherence times T due to this mechanism plotted as function of Δ_{sv} for two values of the E_{VS} gradient, corresponding to the two resonances that occur at $d \approx 232$ nm when $B = 40$ mT. For the resonance at which the gradient is smaller, we obtain $T < 1$ μs for $\Delta_{sv} > 100$ neV. While this shows that the spin-valley flip-flop could explain the observed decrease of coherence time of the shuttled dot from > 4 μs to ≈ 2.6 μs at the B field is raised from 10 to 40 mT at $v_S = 5.6$ m/s, the fact that T at 40 mT exhibits visible v_S dependence, suggestes that the spin-valley flip-flop is not the process that dominates the observed decoherence. The fact that the T fitted to the experimental results also visibly vary between $B = 10$ and 20 mT (i.e. in the field range in which there is no spin-valley resonance, and no contribution to T from the above-discussed mechanism), additionally suggests that the spin-valley related mechanism, while in principle relevant in range of Δ_{sv}, v_S, and $|\mathrm{d}E_{\mathrm{VS}}/\mathrm{d}x|$ encountered in experiments, does not explain the magnetic field and shuttling velocity dependence of the observed T.

B: Passages through a valley splitting minimum

Let us thus move on to the mechanism associated with intervalley excitation - and the resulting change of g-factor of the shuttled spin - that occurs when a valley splitting minimum is traversed with large enough v_S.

As discussed in Sec. SUPPLEMENTARY NOTE VI, shuttling through a minimum of $E_{\mathrm{VS}}(x)$ results in finite probability Q_v of valley excitation. Example calculations from that Section have shown that when $E_{\mathrm{VS}}^{min} < 25$ μeV, we can expect $Q_v \sim 0.01 - 0.1$ for $v_S \sim 10$ m/s. Furthermore, when the shape of $E_{\mathrm{VS}}(x)$ near the minimum at x_m is symmetric, and the dependence of $E_{\mathrm{VS}}(x)$ is close to a linear one in wide range of x not too close to x_m, then we can count that with sizable probability the Landau-Zener model calculation to give a good approximation to Q_v (especially at lowest values of E_{VS}^{min}, say below 10 μeV). Note however that is observation, based on simulations of ~ 10 trajectories of $\Delta(x)$, should be taken with a grain of salt, as it is known that trajectories of Δ can lead shapes of $E_{\mathrm{VS}}(x)$ in perfect agreement with the L-Z model, while giving results for $Q_v(v_S)$ that are in qualitative disagreement with this model [4]. However we can set this issue aside when focusing on data shown in Fig. 4 of the main text, as the measured $E_{\mathrm{VS}}(x)$ for $x \in [220, 250]$ nm shown there exhibits two minima separated by ≈ 15 nm (i.e. a distance similar to the autocorrelation length of valley coupling). As illustrated in the fifth row from of Fig. 12, such a shape of $E_{\mathrm{VS}}(x) = 2|\Delta(x)|$ is most probably caused by a trajectory of $\Delta(x)$ in complex plane that makes a loop, or at least a sizable fraction of a loop, around zero. As shown in that figure, this can result in $Q_v(v_S)$ dependence that shows qualitative deviations (e.g. plateaus, possibly even oscillations) from the Landau-Zener result for $Q_v(v_S)$. More investigations into the statistical properties of $Q_v(v_S)$ curves for many realizations of valley coupling disorder would be necessary to discuss more quantitatively the probabilities that a given feature in $E_{\mathrm{VS}}(x)$ corresponds to a particular $Q_v(v_S)$ dependence. Here we will take a "black box" approach to the valley excitation probability at a double minimum of $E_{\mathrm{VS}}(x)$: we will do a model calculation of the spin dephasing due to multiple passages through a region in which the valley dynamics is expected to be partially nonadiabatic, and we will check if we can reproduce the observed decoherence time that changes from more that 4 μs at $B = 10$ to ≈ 2.6 μs at 40 mT when $v_S = 5.6$ m/s, and then comment on the suppression of this time down to ≈ 1.5 μs when v_S is raised to 22.4 m/s.

We consider repeated shuttling n_{rep} times over distance λ and back, with time of one there-and-back shuttle given by $\tau_S = 2\lambda/v_S$, and total shuttling time $\tau \equiv n_{rep}\tau_S$. When the shuttled qubit is in valley ν (with $\nu = \pm 1$, and $\nu = -1$ denoting the ground valley state), the spin phase picked up during one cycle of shuttling is given by Eq. (S21) with valley-specific $\bar{\omega}_\nu(\lambda)$, i.e. this phase is $\Delta\phi_\nu = \bar{\omega}_\nu(\lambda)\tau_S$. We focus now on a single minimum of $E_{\mathrm{VS}}(d)$ at $d = d_e$, at which a change of valley state that results in change of precession frequency of the shuttled qubit, occurs with probability Q_v. We assume that $Q_v \ll 1$, so that after a valley state change the qubit spends typically $\ll 1$ cycles in the new state. In this limit we can ignore the fact that valley flipping events happen at discrete times (of passage through d_e either on the way to λ, or on the way back), and set up a model in which the valley flipping occurs with rate

$$\gamma \equiv \frac{2Q_v}{\tau_S} = \frac{Q_v v_S}{\lambda} \ . \tag{S38}$$

Let us write the valley-dependent shuttling frequency $\bar{\omega}_\nu$ as

$$\bar{\omega}_\nu = \bar{\omega}_0 + \delta\bar{\omega}_\nu \ , \tag{S39}$$

and observe that the phase acquired during one realization of the initialization-shuttling-readout experiment can be approximated by

$$\phi(\tau) = (\bar{\omega}_0 + \bar{\omega}_{av})\tau + \delta\bar{\omega} \int_0^\tau \xi(t)\mathrm{d}t \ , \tag{S40}$$

where

$$\bar{\omega}_{av} = \frac{1}{2}(\delta\bar{\omega}_+ + \delta\bar{\omega}_-) \ , \tag{S41}$$

$$\delta\bar{\omega} = \frac{1}{2}(\delta\bar{\omega}_+ - \delta\bar{\omega}_-) \equiv \overline{\delta g}\mu_B B \ , \tag{S42}$$

where $\overline{\delta g}$ is is the difference of g-factors in two valleys averaged over the distance λ, and $\xi(t)$ is a realization of Random Telegraph Noise process, with ξ switching between $\xi = 1$ and $\xi = -1$ with rate γ. Averaging of this phase over realizations of the stochastic process $\xi(t)$ is thus equivalent to calculation of dephasing of the qubit the splitting of which is affected by random telegraphic noise (RTN). This problem has a known solution [20–22] that reads

$$W(\tau) \equiv \langle e^{-i\phi(\tau)} \rangle = e^{-i(\bar{\omega}_0 + \bar{\omega}_{av})\tau} e^{-\gamma\tau} \left(\cosh\gamma\mu\tau + \frac{\gamma - ip\delta\bar{\omega}}{\gamma\mu} \sinh\gamma\mu\tau \right) \tag{S43}$$

where $\langle ... \rangle$ denotes the average over the realizations of noise ξ, $p = -1$ $(+1)$ if the evolution starts with the shuttled qubit in $-$ $(+)$ valley state, and $\mu \equiv \sqrt{1 - \delta\bar{\omega}^2/\gamma^2}$.

The key features of the above expression for dephasing factor $W(\tau)$ are the following: When $\gamma \ll \delta\bar{\omega}$ we are in the so-called strong coupling regime, in which the typical random phase acquired after a single valley-flipping event is $\gg 1$, and its variance is of the same order of magnitude. The characteristic decay timescale is then simply γ^{-1}: the envelope of decay of is approximately exponential, $|W(\tau)| \approx \exp(-\tau/T_S)$, with the strong-coupling decay time $T_S = \gamma^{-1}$. Note that this was the limit in which the valley-excitation-induced dephasing of the shuttled spin was considered in [12]. On the other hand, when $\gamma \gg \delta\bar{\omega}$ we are in the so-called weak coupling regime, in which multiple valley flips occur before dephasing becomes significant, each random phase contribution is $\ll 1$, and the signs of these contributions are random, so that the total random phase evolves with τ as a random walk. We are then in the motional narrowing regime: the above expression for $W(\tau)$ is then approximated as

$$W_{weak}(\tau) \approx e^{-i(\bar{\omega}_0 + \bar{\omega}_{av})\tau} e^{-\tau/T_W} \ , \tag{S44}$$

in which the weak-coupling timescale of exponential decay is

$$T_W = \frac{2\gamma}{\delta\bar{\omega}^2} \ . \tag{S45}$$

For fixed $\delta\bar{\omega}$ (i.e. fixed B field and maximum shuttling distance λ), the characteristic decay timescale T of qubit's phase is thus a non-monotonic function of $\gamma = Q_v v_S/\lambda$, with the minimum dephasing time given by

$$T_{min} \approx \frac{2}{\delta\bar{\omega}} \ , \tag{S46}$$

which is obtained when $\gamma \approx \delta\bar{\omega}$.

The measurement of T as function of B and v_S for shuttling channel at $y = 0$ are shown in Fig. 4 of the main text, and fitted T timescales of exponential decay fits are given in Table I there. The two visible trends are: (1) decrease of T with increasing B at $v_S = 5.6$ m/s, and (2) further decrease of T with increasing v_S at $B = 40$ mT. In Fig. 17, we show a plot of $T(\gamma)$ (defined by $|W(T)| = 1/e$) for three values of magnetic field. The average g-factor intervalley difference used in the calculation is chosen to quantitatively reproduce the result (1) when $\gamma \approx 0.5$ μs^{-1}, corresponding to $Q_v \approx 0.015$, while allowing for further decrease of T when $\gamma = Q_v v_S/\lambda$ is increased. In Fig. 18 we show the results for the dephasing factor $|W(\tau)|$ calculated with these parameters for $B = 10$, 20, 40 mT. Note that at higher values of B, when we enter the strong coupling regime, the envelope of the coherence signal exhibits plateaus at $\tau \approx k\pi/\delta\bar{\omega}$ (with integer k) characteristic for dephasing due to RTN.

The last experimental result to be be qualitativel reproduced is the decrease of T at $B = 40$ mT as v_S is increased from 5.6 to 22.4 m/s. In order to model this we need to know the shuttling velocity dependence of $Q_v(v_S)$, in order

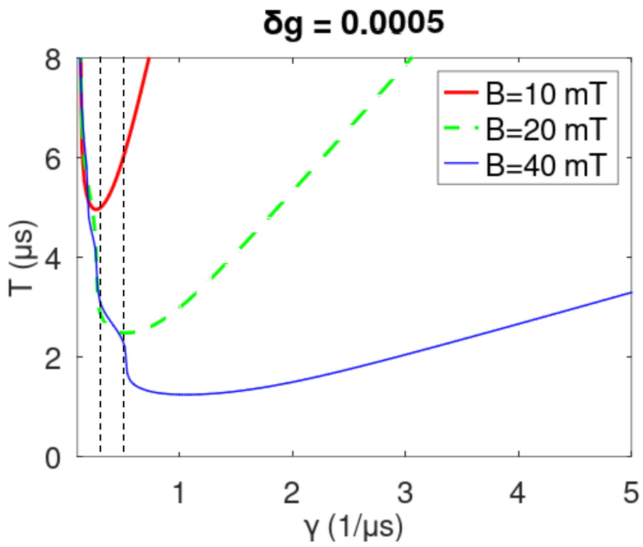

Supplementary Figure 17. A plot of approximate dephasing time $T(\gamma)$ due to multiple passages through $E_{\rm VS}$ minimum resulting in valley state changes occuring with probability Q_v per passage. δg is the difference of valley-dependent g-factors averaged over the shuttling length λ. The valley flip rate is $\gamma = Q_v v_S/\lambda$. For γ in the range between the two vertical dashed lines, increasing B from 10 to 20 mT leads to a suppression of T from > 4 μs to ≈ 2 μs, while the subsequent increase of B to 40 mT leads to a much smaller decrease of T. This reproduces the measured magnetic field dependence of T for $v_S = 5.6$ m/s, see Table I of the main text.

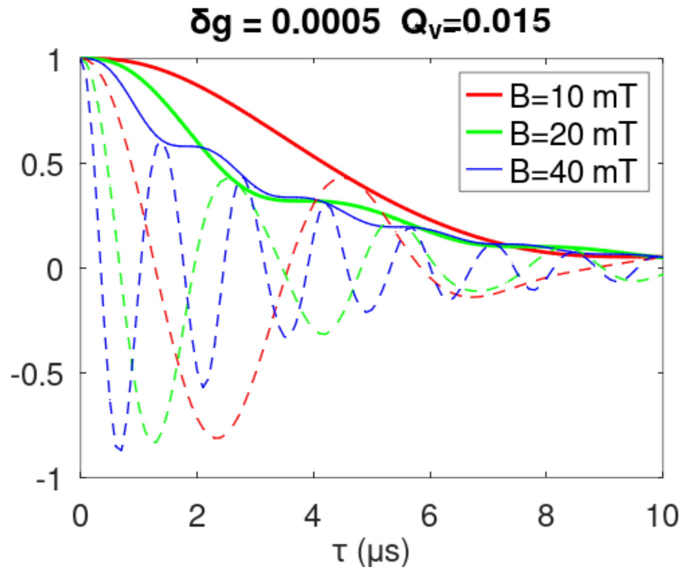

Supplementary Figure 18. Coherence decay from Eq. (S43) calculated for $\delta\bar{\omega}$ used in Fig. 17, with $v_S = 5.6$ m/s and $Q_v = 0.015$. Solid lines: envelope of coherence function, $|W(\tau)|$; dashed lines are Re$W(\tau)$ with average g-factor determining the magnetic field dependence of $\bar{\omega}_0 + \bar{\omega}_{av}$ chosen to reproduce the measured oscillation frequency dependence on the magnetic field.

to relate γ to v_S. Clearly, if Q_v strongly increases with v_S, as in the Landau-Zener model in the low-velocity regime, in which $Q_v \ll 1$, even a two-fold increase of v_S will push $\gamma \propto v_S Q_v(v_S)$ to value deep in the weak coupling regime, resulting in **increase** of coherence time. On the other hand, at v_S high enough that the dynamics is approximately diabatic, we expect Q_v to approach a constant $Q_{v,diab}$. It is crucial to take now into account two things.

First, for trajectories of $\Delta(x)$ that correspond to local minima of $E_{\rm VS}(x)$ (see Section SUPPLEMENTARY NOTE VI), we **do not** expect $Q_{v,diab} = 1$, as it should be given by overlap squared of the initial lower (higher) energy valley eigenstatate and the final higher (lower) energy eigenstate. For $\Delta(x)$ trajectories shown in Fig. 12 these overlaps, and

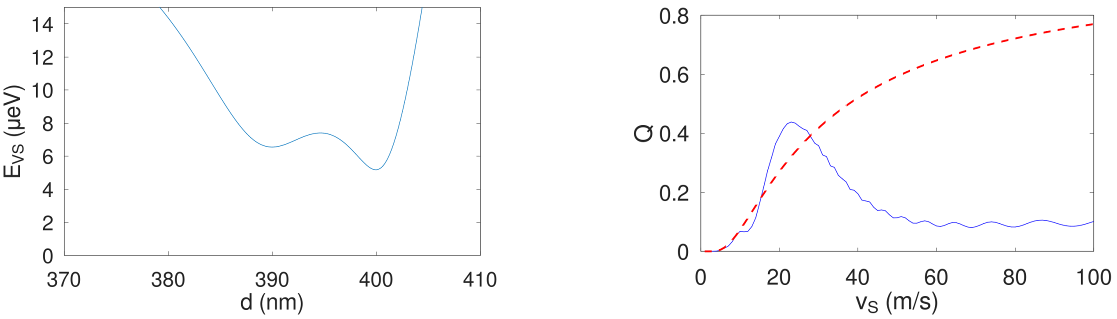

Supplementary Figure 19. Using $\Delta(x)$ from the fifth row of Fig. 12 downscaled by a factor of 2 we obtain the $E_{\rm VS}(d)$ dependence similar shown in the left panel, which has not only the qualitatively similar double-minmum shape as the one encountered in the experiment, but it also has the minimal $E_{\rm VS}$ of about 5 μeV. The valley flip probability Q_v for shuttling through such a double minimum of valley splitting is shown in the right panel, in which the solid blue line is the numerical calculation, and the red dashed line is the Landau-Zener approximation obtained in the way described in Sec. SUPPLEMENTARY NOTE VI.

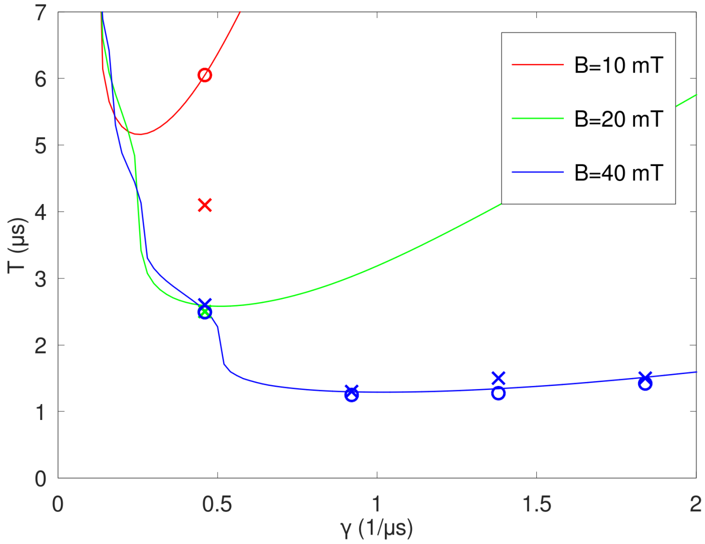

Supplementary Figure 20. Calculated $T(\gamma)$ for $B = 10$, 20, 40 mT with $Q_v = 0.023$ and $\delta\bar{\omega} = 4.8 \cdot 10^{-4} \mu_B B$. Measured values at corresponding v_S (determining $\gamma = Q_v v_S/\lambda$) are marked with crosses, with colors marking the B field. The circles are the theoretical values.

thus the values of $Q_{v,diab}$ are not equal to 1; for example for the fifth trajectory shown there that corresponds to a double minimum of $E_{\rm VS}(x)$, Q_v saturates at ≈ 0.1 for $v_S > 100$ m/s. After dividing the value of valley coupling for the fifth trajectory of Δ, so that we obtain the minimum values of $E_{\rm VS}$ closer to the ones measured at $d_e = 230 - 240$ nm, we obtain the $Q_v(v_S)$ dependence shown in Fig. 19

Second, the above discussed model of dephasing still applies, when $Q_{v,diab} \approx 1$. In this regime it is the lack of valley flip that is a rare event that stochastically perturbs the deterministic evolution, only now this evolution involves valley state change at every passage through d_e. After changing Q_v to $1 - Q_v$, and appropriately redefining $\delta\bar{\omega}$, all the above formulas and discussions apply to this case of almost-diabatic evolution.

These two observations, together with the previously made observation (see Sec. SUPPLEMENTARY NOTE VI) that $Q_v(v_S)$ can exhibit plateaus as a function of v_S when shuttling through a double minimum of $E_{\rm VS}(x)$, show that the assumption that Q_v is approximately velocity-independent is not unreasonable when dealing with shuttling through a valley splitting minimum with $E_{\rm VS}^{min} \leq 5$ μeV.

Coming back to the results shown in Fig. 17: with Q_v that does not depend on v_S (at least in the considered range of velocities), or, even better, one that is decreasing with v_S in such a way that γ increases sub-linearly with v_S, we can obtain T decreasing down to the minimal value of ≈ 1 μs. The model in which we assume $\overline{\delta g} \approx 5 \cdot 10^{-4}$ and assume that $Q_v \approx 0.015 - 0.025$ is v_S-independent, is thus in semi-quantitative agreement with the observations.

In order to quantify the degree of agreement more precisely, we have performed fits of the T times following from the above formulas for $W(\tau)$, using $\overline{\delta g}$ and Q_v as fitting parameters, and assuming that Q_v does not depend on the shuttling velocity. We have not used the measured T for $B = 10$ mT in the fitting, as we suspect that this value is in fact determined by the B- and v_S-independent dephasing of the stationary spin. The best fit was obtained for $Q_v = 0.023 \pm 0.003$ and $\overline{\delta g} = (4.8 \pm 0.3) \cdot 10^{-4}$, and the resulting calculated values of T (including the $B = 10$ mT point, for which the fitted value is larger than the measured one, consistently with our assumption that dephasing time of the shuttled dot at this field is > 4 μs) are shown in Fig. 20.

---

[1] Cywinski, Ł. *et al.*. Singlet-triplet oscillations in multivalley Si quantum dots (2026). In preparation.

[2] Volmer, M. *et al.* Mapping of valley splitting by conveyor-mode spin-coherent electron shuttling. *npj Quantum Inf.* **10**, 61 (2024).

[3] Losert, M. P. *et al.* Strategies for enhancing spin-shuttling fidelities in Si/SiGe quantum wells with random-alloy disorder. *PRX Quantum* **5**, 040322 (2024).

[4] Lima, J. R. F. & Burkard, G. Partial landau-zener transitions and applications to qubit shuttling. *Phys. Rev. B* **111**, 235439 (2025).

[5] Ruskov, R., Veldhorst, M., Dzurak, A. S. & Tahan, C. Electron g-factor of valley states in realistic silicon quantum dots. *Phys. Rev. B* **98**, 245424 (2018).

[6] Woods, B. D., Losert, M. P., Joynt, R. & Friesen, M. g-factor theory of Si/SiGe quantum dots: spin-valley and giant renormalization effects. *arXiv:2412.19795* (2024). Preprint at https://arxiv.org/abs/2412.19795.

[7] Yang, C. H. *et al.* Spin-valley lifetimes in a silicon quantum dot with tunable valley splitting. *Nat. Commun.* **4**, 2069 (2013).

[8] Hwang, J. C. C. *et al.* Impact of g-factors and valleys on spin qubits in a silicon double quantum dot. *Phys. Rev. B* **96**, 045302 (2017).

[9] Paquelet Wuetz, B. *et al.* Atomic fluctuations lifting the energy degeneracy in Si/SiGe quantum dots. *Nat. Commun.* **13**, 7730 (2022).

[10] Losert, M. P. *et al.* Practical strategies for enhancing the valley splitting in Si/SiGe quantum wells. *Phys. Rev. B* **108**, 125405 (2023).

[11] Lima, J. R. F. & Burkard, G. Interface and electromagnetic effects in the valley splitting of Si quantum dots. *Mater. Quantum Technol.* **3**, 025004 (2023).

[12] Langrock, V. *et al.* Blueprint of a scalable spin qubit shuttle device for coherent mid-range qubit transfer in disordered Si/SiGe/SiO_2. *PRX Quantum* **4**, 020305 (2023).

[13] Struck, T. *et al.* Spin-EPR-pair separation by conveyor-mode single electron shuttling in Si/SiGe. *Nat. Commun.* **15**, 1325 (2024).

[14] Yoneda, J. *et al.* Noise-correlation spectrum for a pair of spin qubits in silicon. *Nat. Phys.* **19**, 1793 (2023).

[15] Cai, X., Connors, E. J., Edge, L. F. & Nichol, J. M. Coherent spin–valley oscillations in silicon. *Nat. Phys.* **19**, 386 (2023).

[16] Jock, R. M. *et al.* A silicon singlet–triplet qubit driven by spin-valley coupling. *Nat. Commun.* **13**, 641 (2022).

[17] Huang, P. & Hu, X. Spin relaxation in a si quantum dot due to spin-valley mixing. *Phys. Rev. B* **90**, 235315 (2014).

[18] Hollmann, A. *et al.* Large, tunable valley splitting and single-spin relaxation mechanisms in a Si/Si_xGe_{1-x} quantum dot. *Phys. Rev. Appl.* **13**, 034068 (2020).

[19] Friesen, M. & Coppersmith, S. N. Theory of valley-orbit coupling in a Si/SiGe quantum dot. *Phys. Rev. B* **81**, 115324 (2010).

[20] Paladino, E., Galperin, Y. M., Falci, G. & Altshuler, B. L. $1/f$ noise: Implications for solid-state quantum information. *Rev. Mod. Phys.* **86**, 361 (2014).

[21] Ramon, G. Dynamical decoupling of a singlet-triplet qubit afflicted by a charge fluctuator. *Phys. Rev. B* **86**, 125317 (2012).

[22] Szańkowski, P. Introduction to the theory of open quantum systems. *SciPost Phys. Lect. Notes* **68**, 1 (2023).

Reviewer #2
(Remarks to the Author)

I appreciate the opportunity to review the article "Reduction of the impact of local valley splitting on the coherence of conveyor-belt spin shuttling in 28Si/SiGe" by authors L. Volmer *et al.* for publication in Nature Communications. The article presents a detailed experimental study of the interplay between shuttling parameters (rate, distance, path, time) and the fidelity of a spin state over the shuttling operation. The work draws on the experimental method previously developed by this team (Ref 43) to corelate features in the shuttling fidelity directly with the local structure of the valley splitting and Zeeman energies. Shuttling protocols which preserve spin coherence are of significant importance in the semiconductor spin-qubit community. Further, this work presents a novel experimental characterization of a key material parameter, namely valley splitting, which will be of interest to the wider silicon quantum technology community. In particular, the precise knowledge of valley hot-spots can be useful in order to avoid them to preserve coherence or as a go-to operation point where fast relaxation rates are desired for initialisation into a spin ground state. As such, I support publication of this manuscript in nature communications provided the authors are able to satisfactorily address the following concerns. Below are several major comments which the authors must address before I can support publication, and some minor comments to improve readability.

Major Comments:

1- The authors should provide a clearer explanation of initialization of the (3,1) state from the (4,0) configuration. At present the authors claim that after initializing (3,1) the "*system can be described by a spin-singlet (S) state with potentially mixed valley-state, S_i, where i is an element of {|+->,|+->,|++>, |-,+>}*". The authors support this statement with two references, Refs 55 and 43. The initialization of the (3,1) spin-orbit-valley state is fundamental to the subsequent analysis, which involves interpreting resonances of the initialized state with the polarized triplets (based on ref 43). If I cannot comprehend what (3,1) state is initialized it is difficult to comprehend the meaning of the subsequent analysis.

   Firstly, the inclusion of the word "potentially" is vague. Are the authors implying they are creating a equal mix of the 8 singlet-triplet-valley states, or simply that this could happen, or do they expect only one valley-state (ie |+->) is initialized but they don't know which one. Secondly, the details of the initialization are primarily supported by Ref 55, which is identified as 'In preparation' in the reference list. This means I have no way to independently verify the scientific content of statement (perhaps the authors should wait until Ref 55 is peer reviewed, or at the very least on a preprint server). Finally, the authors also cite ref 43 for their state initialization. However, this work claims that one specific valley-orbit state is initialized, rather than a potential mix of all 4. I would appreciate a plot of the eigenenergy's of the relevant energies to support the understanding of the experiment.

   Fundamentally I trust that the method the authors use to identify valley splitting is independent of the valley-orbit state that is initialized, as I naively expect that there is a symmetry in the behavior of the polarized triplets with respect to the 4 valley-orbit configurations. However, I would expect the authors of the paper to supply necessary figures/equations/assumptions to support this within this manuscript or supplement.

2- Please provide an explicit statement of the fitting method used to quantify the valley-splitting from figure 1d in the main text. I found it confusing to determine exactly how the valley-splitting is quantified using just this manuscript alone. The authors should include a clear direct statement of how the dashed line in figure 1e) is determined in the caption or main text. Reading the supplement of this manuscript I was left more confused regarding the dashed line in figure 1e. In some sections of the supplement (e.g. Supp. Note II) the authors refer to "manual guides to the eye for the found valley splitting curve", however there is no explanation on how the curve

is fitted to the experimental data. Then in the caption they are referred to as "offset copies of spline fits" (such as in supplement figures 2-8). To understand how the dashed line in Figure 1e was assigned I had to look in the supplementary material of Ref 43, where I now understand the dashed line is determined by eye/hand based on discontinuities in the normalized singlet return probability. I have no issue with by eye/hand assignment of the valley-splitting, however it should be clearly stated in the caption.

3- In figure 2d the features in the valley profile appear much sharper along the y-axis than along the x-axis. In particular, in the region of x from 100nm to 220nm there are several undulations in valley splitting with a spatial width of about 50nm in x direction. However, the same undulations rise and fall over less than 10nm in the y direction.

   Can the authors comment on this feature? Is this perhaps an underestimation of the conversion of voltage to y-displacement, are there physical reasons why the x and y valley profile would be different (crystallographic?), or is this just the case for this particular region?

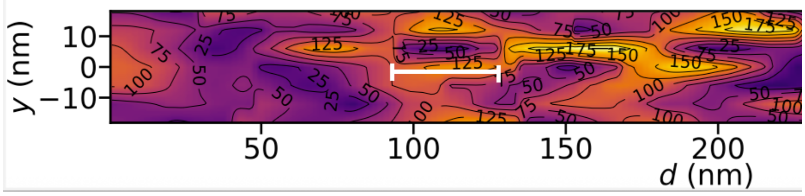

4- The discussion of the features in figures 3b-d) seems quite qualitative and the language of 'spin coherence' leaves some open questions. In Figure 3b-d) the authors show plots of singlet return probability as a function of shuttle distance and total shuttle time. The understanding is that for a fixed distance 'd' a vertical line cut (varying t_s) represents shuttling to the same location at different speeds. To me the 'coherence' would represent the time decay of the state as a function of time. It is not clear if the authors are considering this vertical line cut (t_s) to represent coherence time (ie the P_s decay in the oscillations with respect to the total shuttling time). My understanding is this is not necessarily what the authors are directly interested in, instead they are talking about the maximum probability of returning the initial state, which to me would be more like the 'shuttling fidelity'.

   Within this analysis the authors identify distances that produce a "*sudden reduction in the visibility of the oscillations*." At this point the language of 'coherence' becomes confusing as a reduction in coherence would suggest a quicker decay envelope. Instead, I think the authors are talking about the overall suppression in the amplitude of the oscillations which occurs. The total overall suppression of the oscillations beyond d=220nm, however this may not necessarily be a shortening of the coherence envelope. Instead, an overall suppression in oscillation amplitude could represent spreading the initial state across more possible quantum states, resulting in more complex dynamics (which does seem to happen based on the FFT's in Fig 3e-g). This comment could be addressed by more clearly defining the terms.

Reviewer #2
(Remarks to the Author)

Minor Comments:

1- The title of the manuscript does not accurately reflect the nature of the work. As the authors state in their introduction, they “explore the interplay of spin coherence and conveyor-mode shuttling” in the context of regions of low spin-valley splitting and resonances between valley splitting and Zeeman energy. I agree that the insights provided by this manuscript *could in the future* be used to further reduce the impact of valley splitting on shuttling operations. Additionally, I agree that the authors show that some parameters do show improved/reduced shuttling coherence. However, the current title implies they have demonstrated a systematic reduction in the impact of valley-splitting, which I don’t find accurate. I would suggest a more appropriate title to be along the lines of ‘*A study of the interplay…*’ etc.

2- Figure 1, (particularly section c,d,e) are difficult to read and follow in the present form. Section c was confusing as it doesn’t follow right-to-left and only some transition arrows are included. I would recommend figure 1 be full page wide.

3- In Figure 3c) the arrow labelled 2.8m/s interrupts the visibility of the oscillations. This should be resolved as the text specifically compares the oscillation amplitudes around this region. It is also difficult to judge how much the oscillations are varying by as the additional lines also interrupt the figure. It may be helpful to include an un-edited version of figures 3 b-d in the supplement

4- The section “Spin decoherence during shuttling" is missing an article. “We start with […], since the Zeeman energy of the moving QD […]”.

5- Could the authors use line number for journal proofs to allow more accurate addressing of sections. This will also help the reviewers follow any changes you may add.

6- Relate to major comment 1, the authors refer to ‘valley-orbit’ states. Are you able to comment on the relative energies of the orbital spacing, in order to justify ‘valley-orbit’ states rather than just ‘valley’ states.

7- The acronym 1DEC is defined only in the caption of Fig 1 and the methods section. It would be helpful to define it in the main text.
